# Tumor Microenvironment Subtypes and Immune-Related Signatures for the Prognosis of Breast Cancer

**DOI:** 10.1155/2021/6650107

**Published:** 2021-06-01

**Authors:** Yiqun Han, Jiayu Wang, Binghe Xu

**Affiliations:** Department of Medical Oncology, National Cancer Center/National Clinical Research Center for Cancer/Cancer Hospital, Chinese Academy of Medical Sciences and Peking Union Medical College, No. 17, Panjiayuan Nanli, Chaoyang District, Beijing 100021, China

## Abstract

**Objective:**

To better understand the immune-related heterogeneity of tumor microenvironment (TME) and establish a prognostic model for breast cancer in clinical practice.

**Methods:**

For the 2620 breast cancer cases obtained from The Cancer Genome Atlas and the Molecular Taxonomy of Breast Cancer International Consortium, the CIBERSORT algorithm was performed to identify the immunological pattern, which underwent consensus clustering to curate TME subtypes, and biological profiles were explored by enrichment analysis. Random forest analysis, least absolute shrinkage, and selection operator analysis, in addition to uni- and multivariate COX regression analyses, were successively employed to precisely select the significant genes with prediction values for the introduction of the prognostic model.

**Results:**

Three TME subtypes with distinct molecular and clinical features were identified by an unsupervised clustering approach, of which the molecular heterogeneity could be the result of cell cycle dysfunction and the variation of cytotoxic T lymphocyte activity. A total of 15 significant genes were proposed to construct the prognostic immune-related score system, and a predictive model was established in combination with clinicopathological characteristics for the survival of breast cancer patients. For immunological signatures, proactivity of CD8 T lymphocytes and hyperangiogenesis could be attributed to heterogeneous survival profiles.

**Conclusions:**

We developed and validated a prognostic model based on immune-related signatures for breast cancer. This promising model is justified for validation and optimized in future clinical practice.

## 1. Introduction

Currently, the landscape of the tumor microenvironment (TME) has been generally portrayed, of which the components are considered an essential composition of cancer immunity, with counterpart activities across the “immunoediting” process [1]. In this tumor-related contexture, the density, activity, and organization of immunological infiltration is crucial and regarded as a promising indicator for both clinical response and prognosis of cancer patients [2]. In the advancing era of immunotherapy, several malignancies have been rendered with unprecedented benefits and durable response for the specific tailing effect of novel mechanisms [3]. However, the clinical response provided by immunotherapy was inconsistent among populations or even in the changing stage of an individual, which could be the result of heterogeneities existing in this complicated interactive contexture.

Breast cancer is a worldwide leading newly diagnosed cancer in the female and a heterogeneous disease with the possibility of distinct clinical outcomes [4]. Recent studies using bioinformatics tools have provided insight into the deep mining on the dissection of the TME [5]. However, to further achieve the prediction for the prognosis of patients from clinical practice, taking clinical characteristics into consideration is essential to this implementation. Accordingly, it is critical to integrate comprehensive factors including both data on multiomics and clinical parameters to create a precise system for breast cancer. In this study, we used the transcriptome mixture and clinicopathological information of 2620 individuals which were publicly retrieved on databases, analyzed the potential immune-related mechanisms for divergent TME phenotypes, and constructed a prognostic model with good performance for patients with breast cancer.

## 2. Materials and Methods

### 2.1. Breast Cancer Datasets and Preprocessing

The breast cancer gene expression dataset, as a training cohort, was searched on The Cancer Genome Atlas (TCGA), and the RNA-seq by Expectation-Maximization (RSEM) counts with full clinical information of breast cancer patients (*N* = 1095) was obtained from the University of California, Santa Cruz (UCSC) Xena browser (http://xena.ucsc.edu/). For a supplement, gene expression profile and curated clinical data (*N* = 1525), as the validation dataset, were retrieved from the Molecular Taxonomy of Breast Cancer International Consortium (METABRIC) database which was downloaded from cBioPortal for the Cancer Genomics database (https://cbioportal.org). The process of cohort selection was presented in Supplementary Figure 1, in which the detailed information of included datasets was provided in Supplementary Table 1.

### 2.2. Infiltrating Abundances and TME Subtypes

On the basis of gene expression mixture, we applied the Cell Type Identification by Estimation Relative Subsets of RNA Transcripts (CIBERSORT) approach to infer the relative proportions of infiltrating components, and the algorithms were performed using LM22 signatures with 1000 permutations (http://cibersort.standford.edu/) [6]. The number of TME subtypes was successively determined by the consensus clustering algorithm on the basis of hierarchical agglomerative clustering methods, using ConsensusClusterPlus package of R software [7], based on the quantitatively immunological infiltrating patterns of cases from TCGA-BRCA and METABRIC, respectively, and validated by survival analysis by the Kaplan-Meier (KM) method using the log-rank test.

Identification for differentially expressed genes (DEGs) among TME clusters was accomplished with limma R package [8], in which the absolute of fold change (FC) more than 1 and *P* value adjusted by the Benjamini-Hochberg method less than 0.05 were considered the criteria for significant DEGs with annotations searched from the GeneCardsSuite database (http://genecards.org/). Enrichment analyses, including Gene Ontology (GO) and Kyoto Encyclopedia of Genes and Genomes (KEGG), were conducted to describe the molecular function and biological profiles of DEGs, while Gene Set Enrichment Analysis (GSEA) was performed to successively explore the potential mechanisms by virtue of the gene sets of h: hallmarks (h.all.v7.1.symbols) and c7: immunologic signatures (c7.all.v7.1.symbols) rendered by the Molecular Signatures Database (http://gsea-msigdb.org/) using the ClusterProfiler R package [9, 10]. Through the STRING database (http://string.db.org/), the functional analysis of protein-protein interaction (PPI) networks was established and further visualized by Cytoscape software (version 3.8.0) [11].

### 2.3. Prognostic Immune-Related Score (pIRS) and Prognostic Model

Survival analyses concerning the expression of each DEG for overall survival (OS) were performed using the KM method by survival and survminer R packages [12], and variables with statistical significance were determined with random forest analysis and least absolute shrinkage and selection operator (LASSO) analysis, using the randomForest package and glmnet package of R software [13], for dimensional reduction and identification of the overlapping DEGs significant for prognosis. Next, a univariate COX proportional hazard model was utilized to differentiate the foremost groups of DEGs, and the prognostic immune-related score (pIRS) was defined as

(1) $$\begin{array}{c} \text{piRS}=\overset{n}{\underset{k=1}{\sum }}{\text{exprs}}_{k}{\text{coef}}_{k}+\overset{n}{\underset{i=1}{\sum }}{\text{exprs}}_{i}{\text{coef}}_{i}, \end{array}$$

where *n* is the number of genes. exprs_*k*_ and coef_*k*_ were the gene expression and regression coefficient for DEGs of which the hazard ratio (HR) is more than 1, while exprs_*i*_ and coef_*i*_ were for the genes of which HR is less than 1. The pIRS of each patient was calculated and recorded for the following analysis.

In combination with clinicopathological characteristics, pIRS was included in the multivariate COX regression analysis to construct a nomogram with the rms package of R software, of which predictive power for prognosis in terms of sensitivity and specificity was assessed by the time-dependent receiver characteristic curve (ROC) using timeROC R package [14]. The discriminative power of the prognostic model was quantitatively measured by Harrell's concordance index (C-index). The flow diagram of data processing and analysis is presented in Supplementary Figure 2.

### 2.4. Statistical Analysis

In this study, correlation analysis was carried out and demonstrated with Spearman's coefficients and the corresponding *P* values. Paired comparative analysis was performed for continuous variables with the independent *t*-test for normal distribution and the Mann-Whitney *U* test for abnormal distribution, respectively, which was visualized using the ggplot2 R package. A *P* value less than 0.05 was regarded as statistically significant. The missing data were excluded against analyses to weaken heterogeneity. All the statistical analyses were 2-sided and conducted by R software (version 3.6.4).

## 3. Results

### 3.1. Identification of TME Subtypes

**Figure 1:**
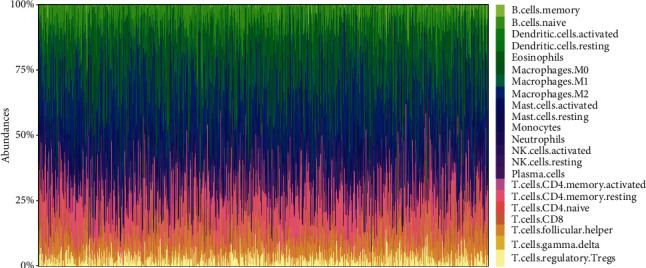
The landscape of immunologic infiltrations in the TME of breast cancer. The relative proportions of 22 lymphocytes infiltrating in the TME of TCGA-BRCA patients were portrayed, which demonstrated an evident heterogeneity among individuals.

**Figure 2:**
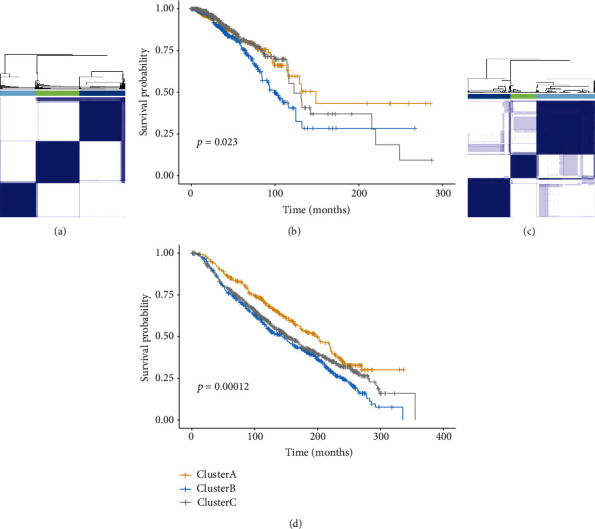
The curated TME subtypes with distinct prognostic outcomes. Three TME phenotypes of TCGA-BRCA patients (*N* = 1095) were retrieved by a consensus clustering method (a) with a significant difference in overall survival (*P* = 0.023) (b). Consistent results were obtained in patients from the METABRIC database (*N* = 1525) (c) with distinction in prognosis (*P* = 0.00012) (d). The survival analyses were performed with the Kaplan-Meier method using the log-rank test.

The infiltration patterns were analyzed with CIBERSORT deconvolution algorithms by quantifying the fractions of 22 immune cell types in TME of 1095 TCGA-BRCA patients. The landscape of immunological infiltration is exhibited in Figure 1, Supplementary Figure 3, and Supplementary Table 2. Data on the estimated proportions of infiltrating cells indicated an evident heterogeneity, considering that an unsupervised clustering analysis was performed to determine the potential TME subtypes. On the basis of the consensus clustering method, three robust TME clusters were retrieved, and the prognosis of patients from these curated subtypes was different with statistical significance (*P* = 0.023) (Figures 2(a) and 2(b)). With the aim of validation, the bulk tissue gene expression profiles of 1525 patients from the METABRIC database were also estimated for immune infiltrations and underwent a clustering analysis (Supplementary Table 3), from which three clusters were obtained with a statistical difference in OS (*P* < 0.001) (Figures 2(c) and 2(d)), indicating that this partition was stable for TME subtypes in terms of immunological infiltrations.

### 3.2. Signatures of TME Phenotypes

**Figure 3:**
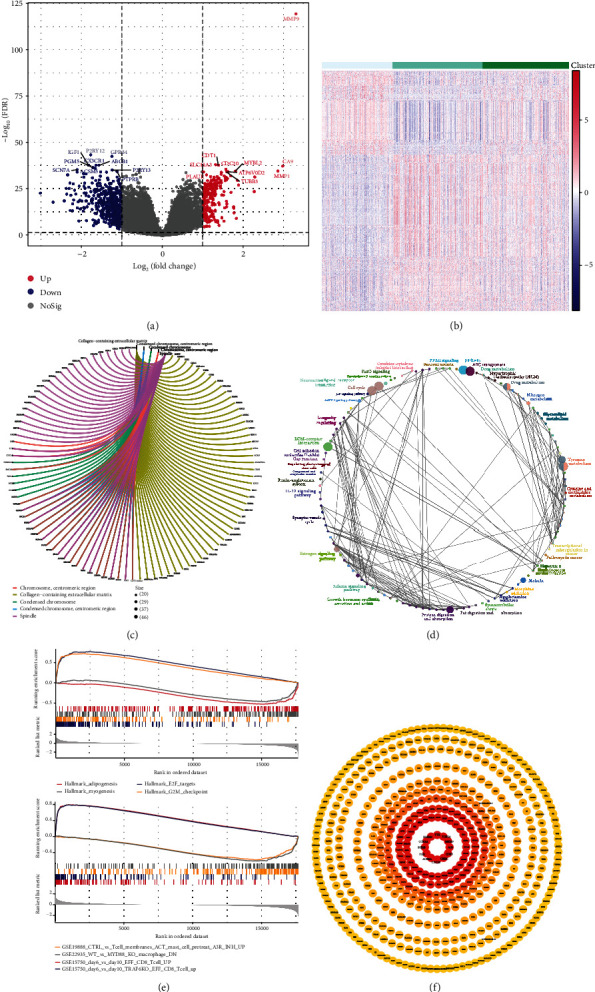
Contributing mechanisms of differential TME phenotypes. A total of 664 significant differentially expressed genes (DEGs) identified through differential analysis with 233 genes upregulated (red) and 431 genes downregulated (blue), respectively (a). DEGs were distributed evenly among three TME subtypes (b). GO analysis (c), KEGG pathways analysis (d), and GSEA (e) were successively carried out, and PPI networks were constructed to explore the interactions (f).

To explore the contributing mechanisms for versatile TME phenotypes, we systematically conducted the differential analysis to identify the DEGs and enrichment analyses to elucidate the biological profiles. A total of 20530 DEGs were identified through differential analysis, of which 664 genes significantly expressed heterogeneity among three immune-related TME subtypes, with 233 genes upregulated and 431 genes downregulated (Figures 3(a) and 3(b), Supplementary Table 4). Among the identified DEGs, MMP9, SLC16A3, CDT1, CA9, and CDC20 were revealed to be the leading upregulated, while P2RY12, GPR34, ABCB1, IGF1, and CX3CR1 were estimated to express downregulation with the foremost significance.

Enrichment analyses, including GO, KEGG, and GSEA, were carried out to portray the landscape of biological profiles of DEGs and illustrate the mechanism contributing to the differential phenotypes. GO analysis demonstrated that the significant DEGs intensively mapped to the GO terms concerning cellular localizations include the collagen-containing extracellular matrix, condensed chromosome, centromeric region, and spindle (Figure 3(c)). Downstream analysis from KEGG suggested that DEGs were significantly involved in the enriched pathways comprising neuroactive ligand-receptor interaction, PI3K-Akt signaling pathways, and cell cycle (Figure 3(d)). GSEA results showed that the DEGs, in terms of cancer hallmarks, were an ensemble of cell cycle dysfunction, including E2F targets and G2M checkpoints, which were significantly muted in metabolisms such as adipogenesis and myogenesis. Regarding immune-related signatures, the improved effectiveness of CD8 T lymphocyte was expected to facilitate the genesis of TME phenotypes (Figure 3(e)). Built on cluster analysis, PPI networks were built to clarify the interactive functions of significant DEGs, and the corresponding relationships were shown with a ranked degree (Figure 3(f)).

### 3.3. Prognostic Value of Signature Genes

**Figure 4:**
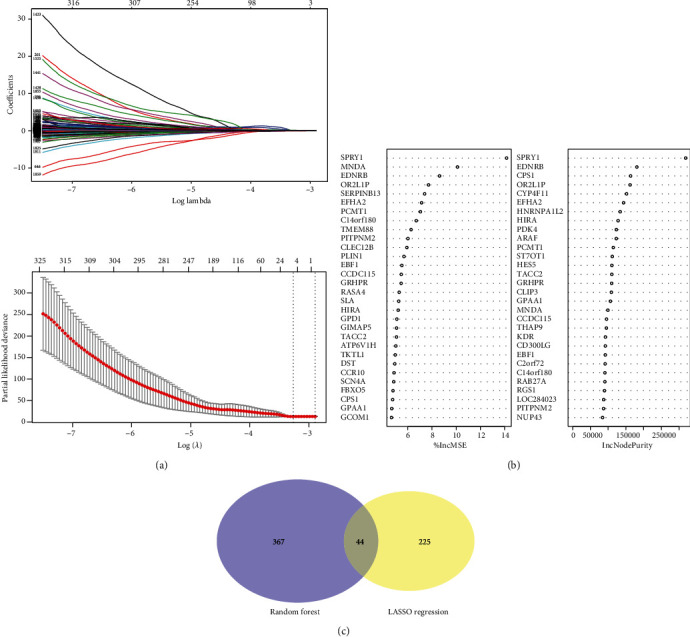
Identification of genes mostly significant for prognosis. LASSO analysis (a) and random forest analysis (b) were synchronously performed for the dimensional reduction and selection of variables, with 411 and 269 genes identified, respectively. The intersected proportion of 44 genes underwent the following analysis (c).

**Figure 5:**
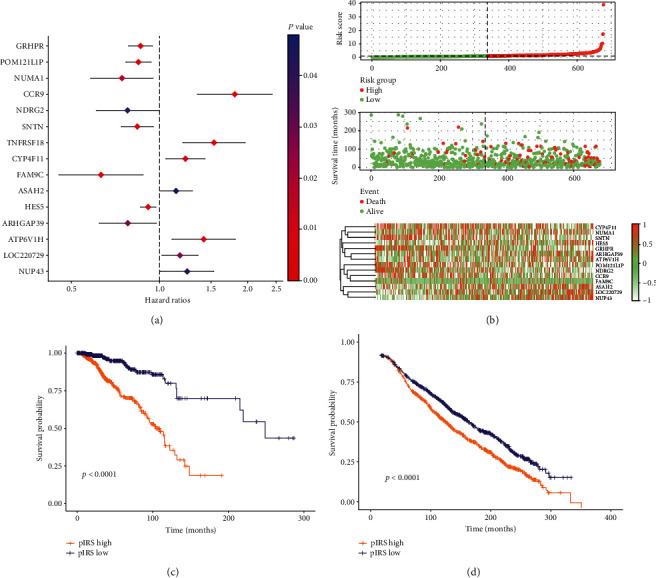
Construction and validation of the prognostic panel. The univariate COX regression analysis was carried out to recognize the variables with the utmost statistical significance with 15 genes determined (a). The correlations between gene expression and prognostic outcomes were demonstrated (b). The partition system based on pIRS was validated with survival analysis using the Kaplan-Meier method, which revealed a significant difference in survival of patients from both TCGA (*P* < 0.0001) (c) and METABRIC database (*P* < 0.0001) (d).

Survival analysis was carried out using the KM method to identify the significant genes with predictive values for survival. The whole 20530 DEGs were successively assessed based on the corresponding expression from each patient, and a total of 2274 genes exhibited a statistical significance for OS. Then, random forest analysis and LASSO analysis were synchronously performed to further recognize the significant genes for prognosis, which discriminated 411 and 269 genes, respectively, and the intersected 44 genes were prepared for the following analysis (Figures 4(a)–4(c)). Next, we carried out univariate COX regression analysis for identification of the most significant variables for survival, and the panel with a total of 15 genes was finally determined (Figures 5(a) and 5(b), Supplementary Table 5).

Subsequently, the pIRS was constructed based on the expression of 15 genes in combination with the corresponding coefficient for each patient, and all cases were stratified into the high pIRS group and the low pIRS group. Survival analysis suggested that patients from these two groups exhibited a divergent clinical prognosis with statistical significance (*P* < 0.0001), which was in accordance with the result of the validation cohort (*P* < 0.0001) (Figures 5(c) and 5(d)).

**Figure 6:**
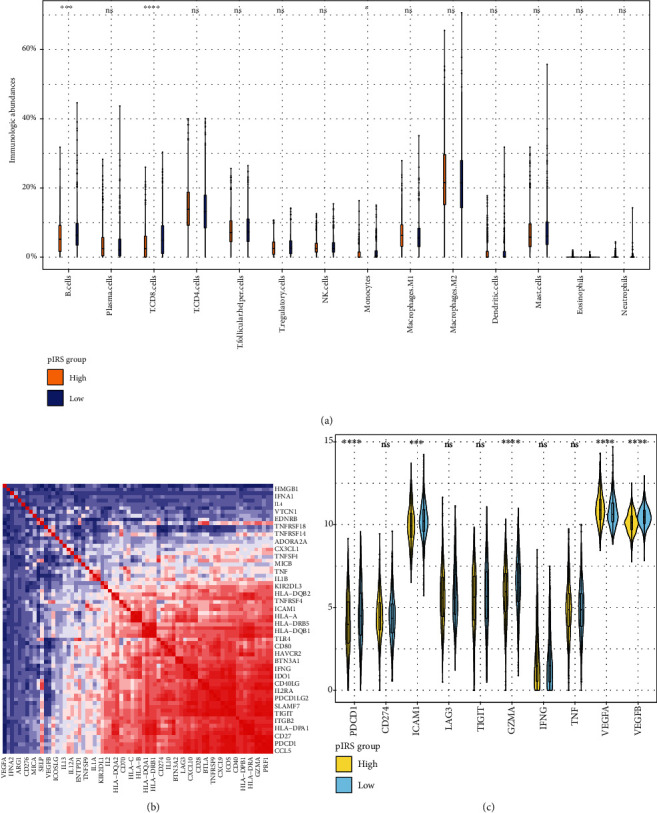
Immunologic profiles of the pIRS groups. Comparative analysis of the abundances of infiltrating immunologic cells from the high pIRS group and the low pIRS group (a). The correlations among immune-related modulators in breast cancer tissue (b) and the difference in immune checkpoint between two groups (c).

Comparative analysis for immunological phenotypes was performed, of which the results indicated that the abundance of B lymphocytes and CD8 T cells was notably higher in the low pIRS group (Figure 6(a)). Distributions of leukocyte infiltration among groups with diverse clinicopathological factors are shown in Supplementary Figure 4. The expression levels of immunologic modulators were also evaluated between two groups with a total of 73 signature genes included [15] (Supplementary Figure 5). Interactive correlations are shown in Figure 6(b), and the genes, in particular, associated with the immune checkpoint underwent comparative analyses (Figure 6(c)). Results from paired analyses showed that the expression of PDCD1 (PD-1) and ICAM1 was significantly higher in the low pIRS group, while VEGFA presented an increasing trend of expression in the high pIRS group.

### 3.4. Prediction Model for Prognosis of Breast Cancer

**Figure 7:**
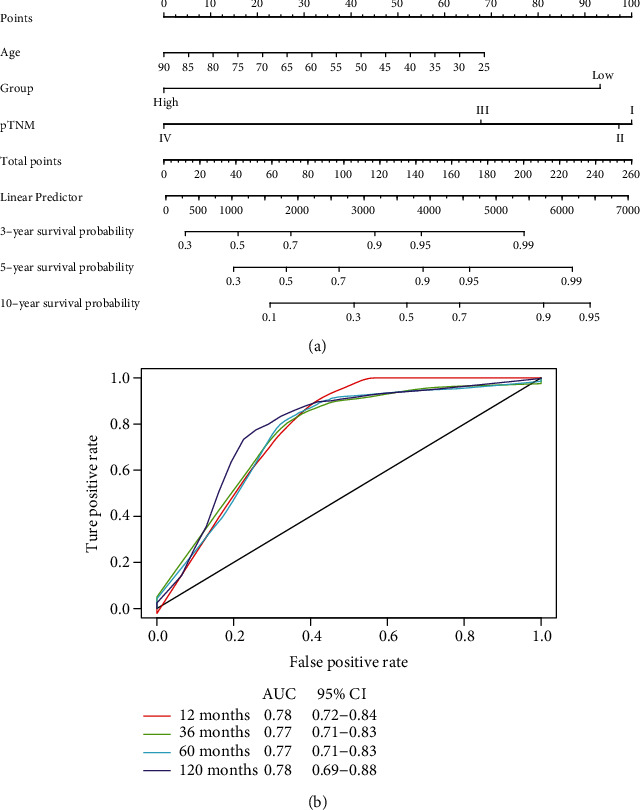
Prognostic model for survival probability of breast cancer. Nomogram constructed for the prediction of 3-year, 5-year, and 10-year survival probability of patients in the training cohort (a). The time-dependent ROC validated the performance of this prognostic model (b).

A total of 677 breast cancer patients with complete clinicopathological characteristics including age, pathological TNM stage (pTNM), molecular features, PAM50 subtypes, and pIRS group were adopted into the COX proportional model for quantitative estimation of survival. Results from multivariate regression analysis demonstrated that the age, pTNM, and pIRS group were independent factors for the prognosis of breast cancer, which were utilized to construct a nomogram for the prediction of 3-year, 5-year, and 10-year survival probability (Figure 7(a), Supplementary Table 6). Time-dependent ROC suggested that the time-dependent under curve area was ranging from 0.77 to 0.78, indicating that the curated prognostic model was well performed (Figure 7(b)), and the quantified C-index was 0.823 obtained from the training cohort and 0.776 from the validation cohort, respectively, which were generally higher than those computed from the TNM staging system, revealing the robustness in addition to better accuracy of this prediction model (Supplementary Table 7).

## 4. Discussion

In this study, we performed an overall analysis of TME immunological profiles based on transcriptome data of breast cancer, discussing the heterogeneity of the stromal contexture in terms of the immune-related subtypes and potential contributing mechanisms, in addition to giving a quantitative estimation of the associations between immune-related parameters and the prognosis of breast cancer.

To quantitatively ascertain the stromal infiltration of TME, the CIBERSORT approach was adopted for computational proportions of immunological cell types of interest [6]. The landscape of immunologic infiltration exhibited a varying tendency among patients, prompting us to perform an analysis for the potential subtypes of TME. Three TME clusters were determined based on consensus clustering algorithms and proven to be stable in the validation cohort. Moreover, results from survival analysis, as the exploratory mining, demonstrated that an apparent difference of survival was detected among the patients from corresponding TME subtypes, indicating that the immunologic heterogeneity of TME could be a promising predictor for the survival of breast cancer. Previous studies have managed to correlate molecular features to the prognosis of breast cancer patients. However, multiomic angles have to be focused which primarily included genomics, epigenetics, and transcriptomic profiles. On the basis of the dataset, Shen and colleagues established an lncRNA panel associated with immunological signatures to stably predict the prognosis of breast cancer [16], which is consistent with several studies [17, 18], and supported that the phenotypes could be the results of molecular heterogeneity. From the immunologic perspective, we indicated that inherent heterogeneity could lead to divergent prognosis and curated three robust TME subtypes for breast cancer, of which the potential mechanisms leading to this kind of differentiation remained to be explored.

Focusing on the illustration of this mechanism, then, we performed systematic analyses for the differential genetic expressions, of which the versatile phenotypes tended to be the product. Differential analysis among three TME clusters recognized the genes with divergent transcriptome profiles, while the enrichment analyses demonstrated that mapping molecular positions lied in the extracellular matrix and nuclear components, and the ensemble pathways were significantly enriched in the cellular signaling transduction involved in cancer proliferation, migration, and invasion. Results from enrichment analysis with specific genetic references suggested that the differential phenotypes could be the concerted result of cell cycle dysfunction, the variety in CD8 T lymphocyte activity, and the repression of metabolite homeostasis, which could be a potential target for novel therapeutics toward the breast cancer population. PPI analysis identified the core protein consisting of TOP2A, MKI67, and CDK1, which was supposed to function at the process for immunological heterogeneity. Based on the tissue-based research, Zheng et al. suggested that TOP2A expression could independently predict the survival of triple-negative breast cancer [19], which was further confirmed by Xu and colleagues [20]. MKI67 has been regarded as a contributing component for molecular heterogeneity of breast cancer validated by previous studies [21, 22]. As an essential regulator for the cell cycle, the activity of CDK1 has long been considered a potential target, which is associated with cell cycle dysfunction and corresponding distinct molecular profiles of breast cancer [23]. These findings were in accordance with the current researches of TTK [24], CDC20 [25], PLK1 [26], and AURKA [27], which were considered the leading contributors for molecular heterogeneity genesis in the current study.

The construction of a prognostic panel experienced systematic analyses with gene shrinkage and selections. Survival analysis using the KM method was initially carried out to identify the significant genes with prognostic values for survival. As the criteria of the group established, the median of gene expression was adopted to partition patients into two groups, and a total of 20530 DEGs successively underwent assessment for statistical significance. Then, the combination of random forest analysis and LASSO analysis was performed for variable shrinkage and selection based on the recognized genes, and the intersection proportion was retrieved for precision. The expression of this group of selected ones, as the continuous variable, was further consecutively adopted into a univariate COX proportional model and evaluated the corresponding significance to survival, and the genetic panel, comprising 15 genes, was finally determined. In combination with regression coefficients, gene expression was quantified as an indicator for OS of breast cancer, and the pIRS system was accordingly constructed as the product. Subsequently, individuals were classified into two pIRS groups based on the immunological contexture. The clinicopathological characteristics and the pIRS group of eligible patients were synchronously assessed by the application of multivariate COX regression analysis to organize a prognostic model, and the independent variables were utilized to create the nomogram validated with good performance.

We also carried out comparative analyses to portray the immune-related features contained in these patients from two groups. Results of tumor-infiltrating cells showed that the abundance of CD8 T lymphocyte, B cells, and monocytes was greatly higher in the low IRS group, revealing that these sorts of immunologic cells could presage improved survival of breast cancer. Currently, the relation between the densities of CD8 effector T cells and prognosis was reportedly controversial [28–31]. These findings confirmed that the improved survival was in positive associations with an enriched abundance of CD8 T cells. On top of that, recent researches have revealed that the spatially distinct distribution of CD8 T cells constitutes the leading reference to classifying the patterns of TME in the breast cancer subgroup [32]. This kind of association between the heterogeneous patterns of CD8 T cells and the clinical outcomes could be potentially correlated to the heterogeneity of TME in breast cancer as well. To further elucidate the inherent correlations between immunologic phenotypes and distinct prognostic outcomes, the curated gene list of immune modulators was adopted [15], of which the correlogram was suggestive of evident collections consisting of these immune-related components. With the in-depth illustration of immunoediting theory and evolution of immunotherapy, immune checkpoints have been considered with cruciality in cancer immunity response, and the developed inhibitors have been dramatically improving the prognosis of several types of malignancies [33–35]. Herein, immune checkpoints were selected to be evaluated between these two pIRS groups. This part of results revealed that PDCD1 [36], ICAM1 [37], and GZMA [38], which were associated with proactivity of cytotoxic T lymphocytes and cancer immunity, were predictive of prolonged prognosis in the low pIRS group, and VEGFA, as the leading molecule of tumor-induced angiogenesis for tumor invasion [39], exhibited an increased expression in breast cancer patients with limited survival of patients from the high pIRS group.

Immune infiltration remains an intriguing focus in the field of oncology research, which has been considered not only an indicator for therapeutic effectiveness but also a promising basis, as the essential step for “immunomonitoring,” to drive treatment evolution [40]. The developing techniques have been applied to dissect tumor-related contexture which has shed novel light on the understanding of tumor heterogeneity and cancer management. However, current research could not fully interpret some clinical phenomenon that was inconsistent with experimental results, for instance, insufficient biomarkers with predictive values for response and prognosis. It seems that the intricate interactions among tumor-related complex systems, including tumor cells, immune cells, and TME, should be elucidated by integrating technologies and curate increasing findings for cancer therapy followed by survival benefits for patients [41]. Indeed, several genetic panels were constructed for the precise prediction of survival in breast cancer cohorts, such as the Oncotype DX assay [42] and MammaPrint panel [43], and have been extensively validated in clinical practice. However, the current panel tends to be developed and designed for a specified cohort and relatively limited in the general utilization for the entire cohort. From this perspective, the curated panel introduced by our research was promising for future practice and promising benefits to breast cancer patients.

Some inevitable limitations should be stated here. Firstly, the sample size and clinicopathological characteristics, for instance, sequential treatment details and concomitant diseases, stored in the publicly available database were limited, which could attribute to the potential bias and weaken the power of the prediction model. Secondly, the partition method for the pIRS groups adopted the median as a cutoff value, which might lead to the inappropriate division of the cohort and the limited unearthing of promising findings. Under this circumstance, the standard for population selection should be optimized in the upcoming research. Last, the focus of TME phenotypes lay in the differential analysis for mechanism; however, the absence of deep mining on the increasing factors that induced intratumor heterogeneity or divergences from populations was obvious.

## 5. Conclusions

In conclusion, we systematically derived the three molecularly and clinically distinct TME subtypes based on immunologic infiltration and explored the potential mechanisms promoting this kind of divergence. A predictive score system was built for population selection, and a prognostic model was built with the combinations with clinicopathological characteristics of cohorts for prediction of survival. Future studies are warranted to absorb increasing factors and optimize this model in favor of clinical practice in the perspective.

## Data Availability

The datasets and/or analysis results used during the current study are available from the corresponding authors upon reasonable request.

## Supplementary Materials

Supplementary Materials Supplementary Figure 1: flow diagram of patient selection and cohort identification. Supplementary Figure 2: the overview and flow diagram of this study. Supplementary Figure 3: correlations among tumor-infiltrating immunologic cell types. Supplementary Figure 4: distributions of leukocyte infiltrations related to clinicopathological characteristics. Supplementary Figure 5: the gene expression of immunologic modulators in the cohort. Supplementary Table 1: basic information of the included datasets. Supplementary Table 2: the immunologic infiltrations of TCGA-BRCA patients quantitively evaluated by CIBERSORT algorithms. Supplementary Table 3: the immunologic infiltrations of METABRIC patients quantitively evaluated by CIBERSORT algorithms. Supplementary Table 4: the significant differentially expressed genes among three TME subtypes. Supplementary Table 5: the identified 15 genes with the foremost significance for prognosis. Supplementary Table 6: clinicopathological characteristics at baseline of training cohort (*N* = 677). Supplementary Table 7: the computational C-index of cohorts.
