# Supplementary material for: Tumor Microenvironment Subtypes and Immune-Related Signatures for the Prognosis of Breast Cancer
Source: Biomed Res Int. 2021 Jun 1;2021:6650107. doi: 10.1155/2021/6650107 (PMC8189770; doi:10.1155/2021/6650107)
Supplement: Supplementary Materials — Supplementary Figure 1: flow diagram of patient selection and cohort identification. Supplementary Figure 2: the overview and flow diagram of this study. Supplementary Figure 3: correlations among tumor-infiltrating immunologic cell types. Supplementary Figure 4: distributions of leukocyte infiltrations related to clinicopathological characteristics. Supplementary Figure 5: the gene expression of immunologic modulators in the cohort. Supplementary Table 1: basic information of the included datasets. Supplementary Table 2: the immunologic infiltrations of TCGA-BRCA patients quantitively evaluated by CIBERSORT algorithms. Supplementary Table 3: the immunologic infiltrations of METABRIC patients quantitively evaluated by CIBERSORT algorithms. Supplementary Table 4: the significant differentially expressed genes among three TME subtypes. Supplementary Table 5: the identified 15 genes with the foremost significance for prognosis. Supplementary Table 6: clinicopathological characteristics at baseline of training cohort (N = 677). Supplementary Table 7: the computational C-index of cohorts. [file 6650107.f1.docx]

**Supplementary Materials**

**Supplementary Figure 1.** Flow diagram of patient selection and cohort identification.

**
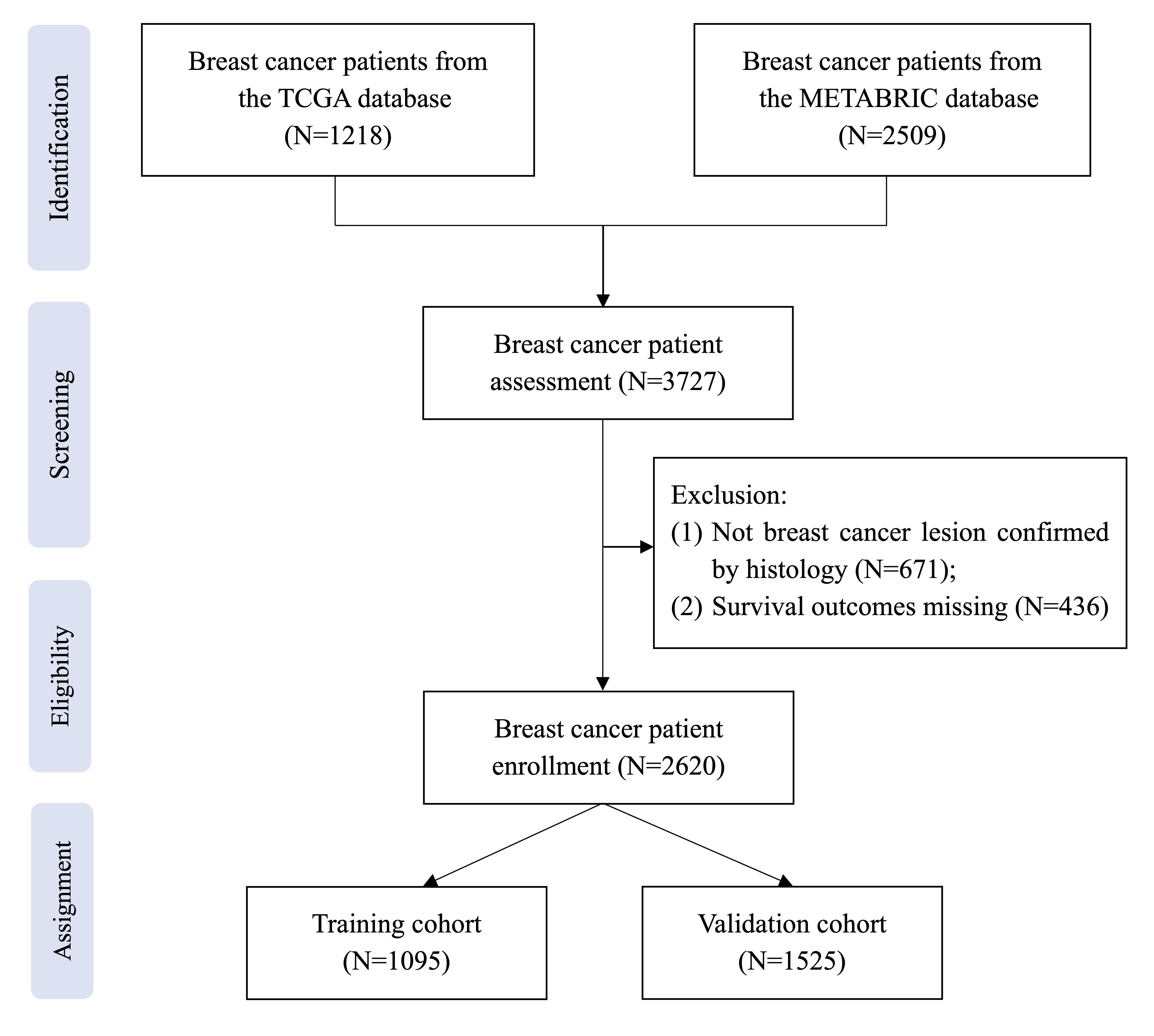
**

**
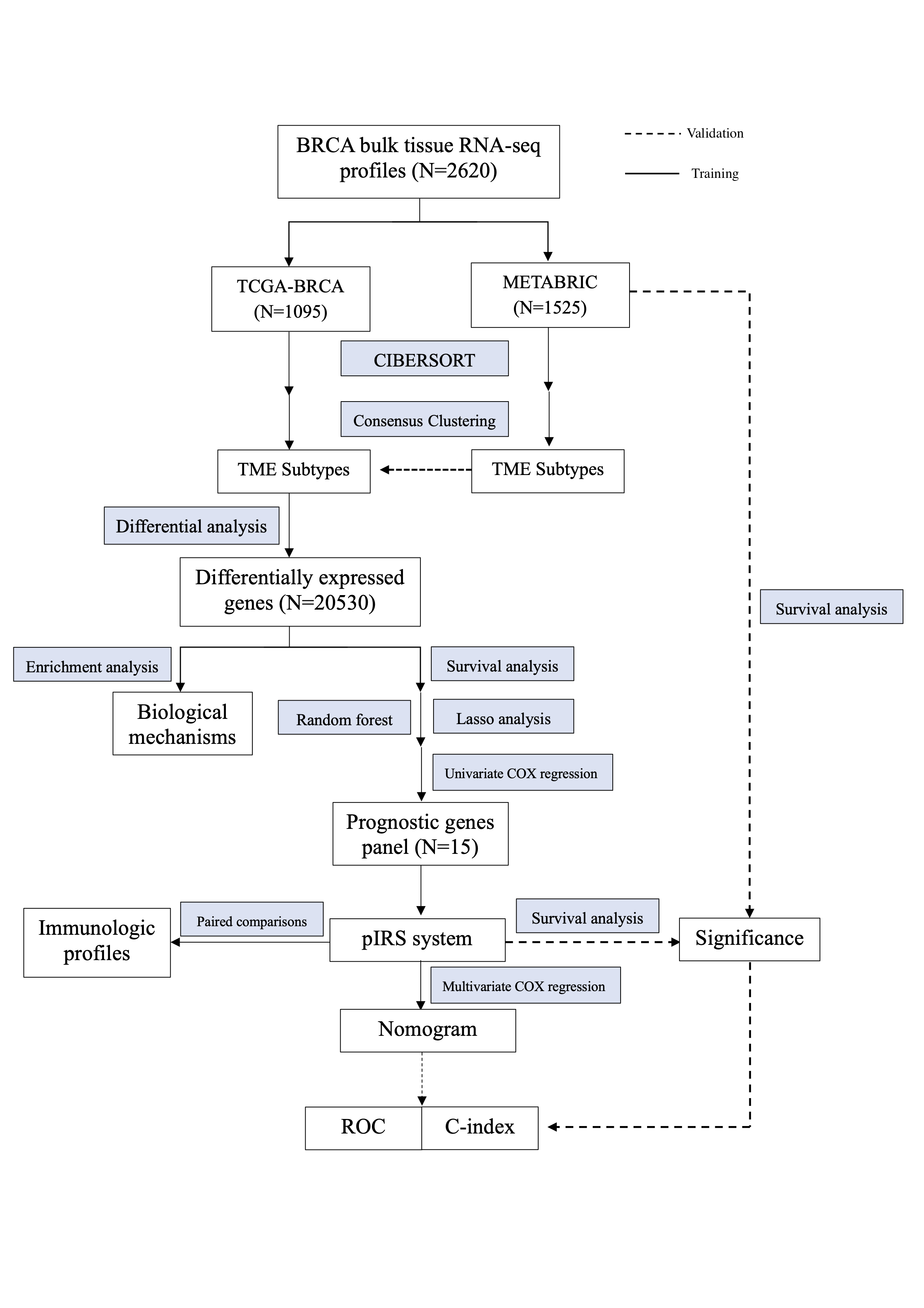
Supplementary Figure 2.** The overview and flow diagram of this study.

**Supplementary Figure 3.** Correlations among tumor infiltrating immunologic cell types.

**
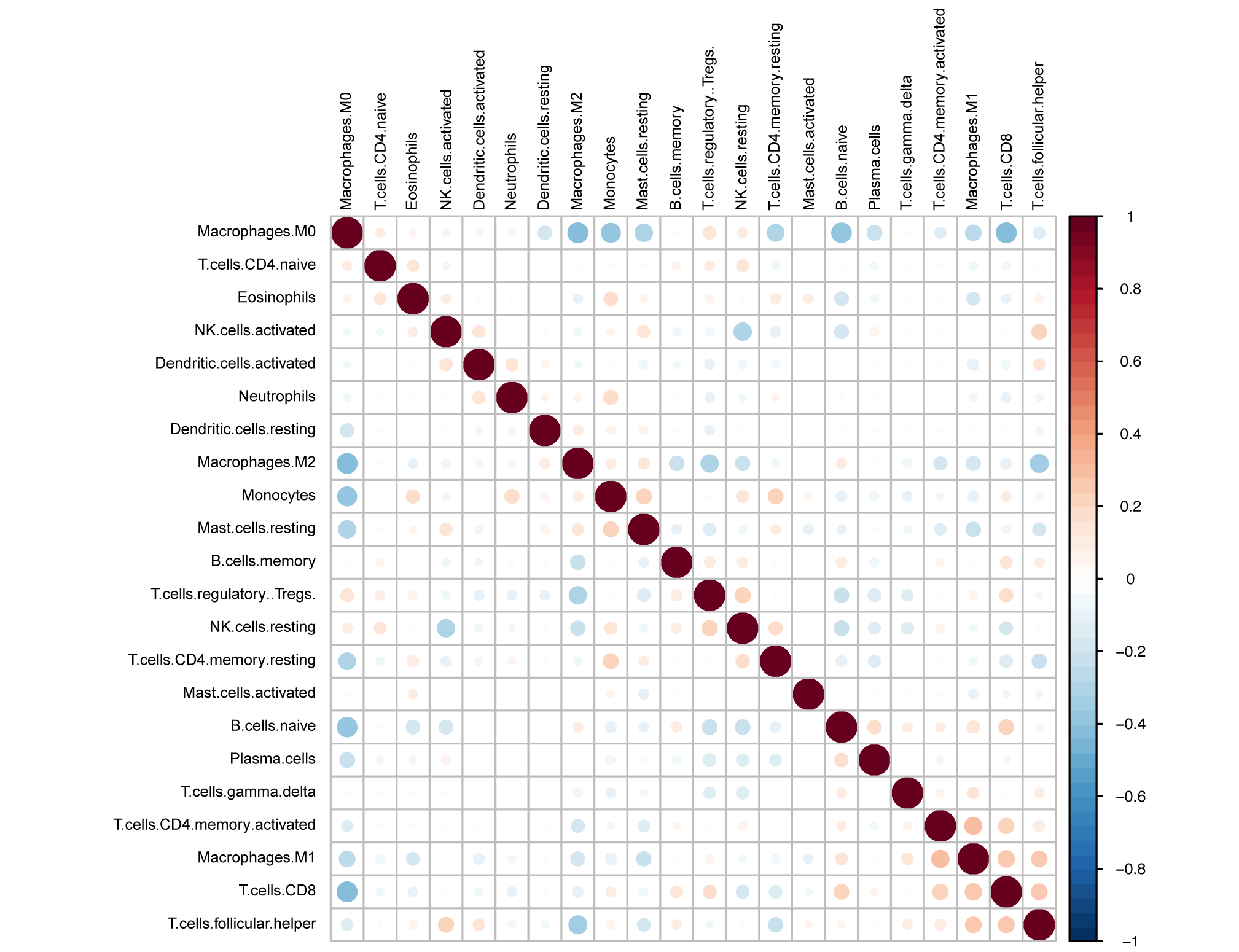
**

**
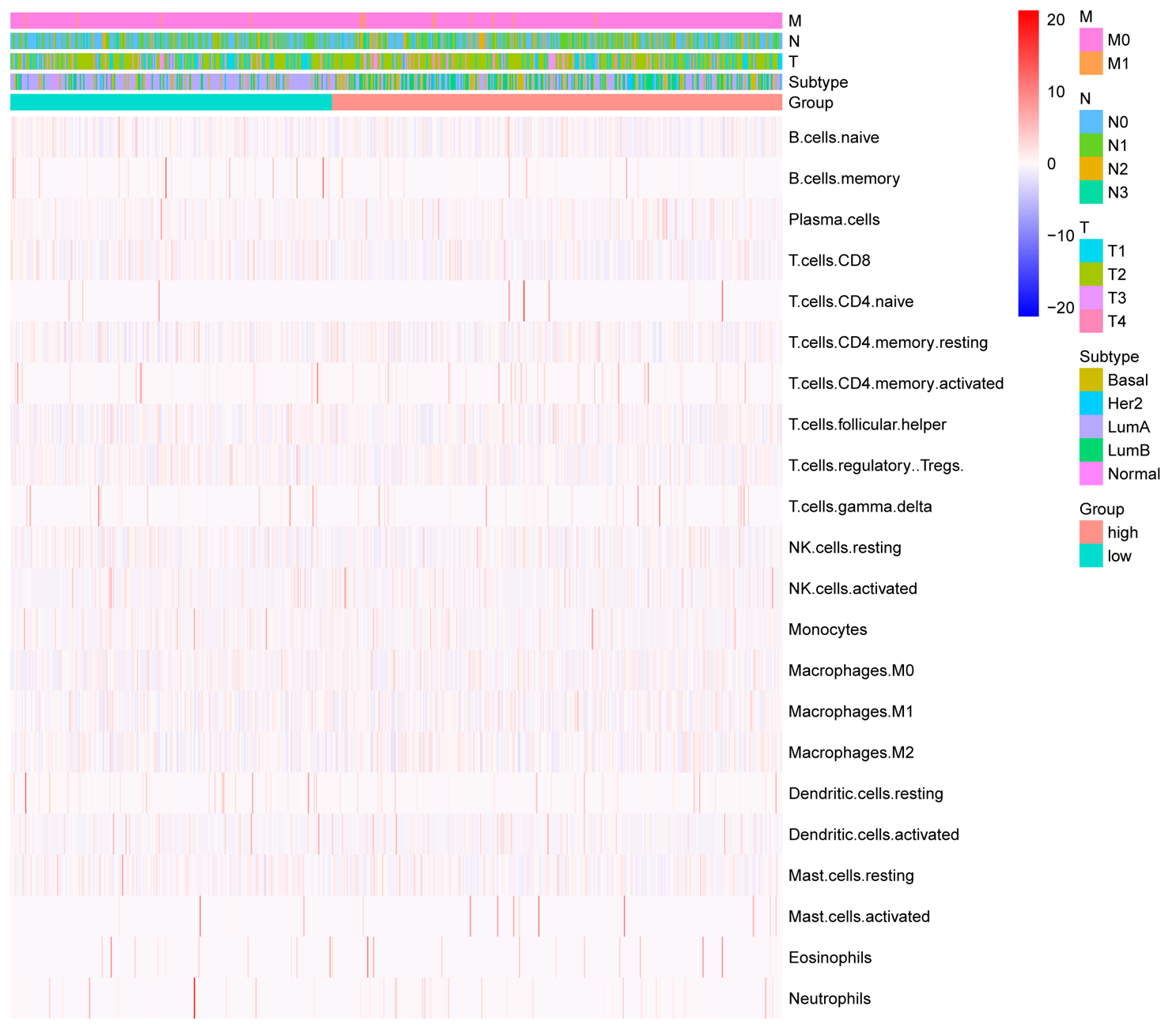
Supplementary Figure 4.** Distributions of leukocyte infiltrations related to clinicopathological characteristics.

**
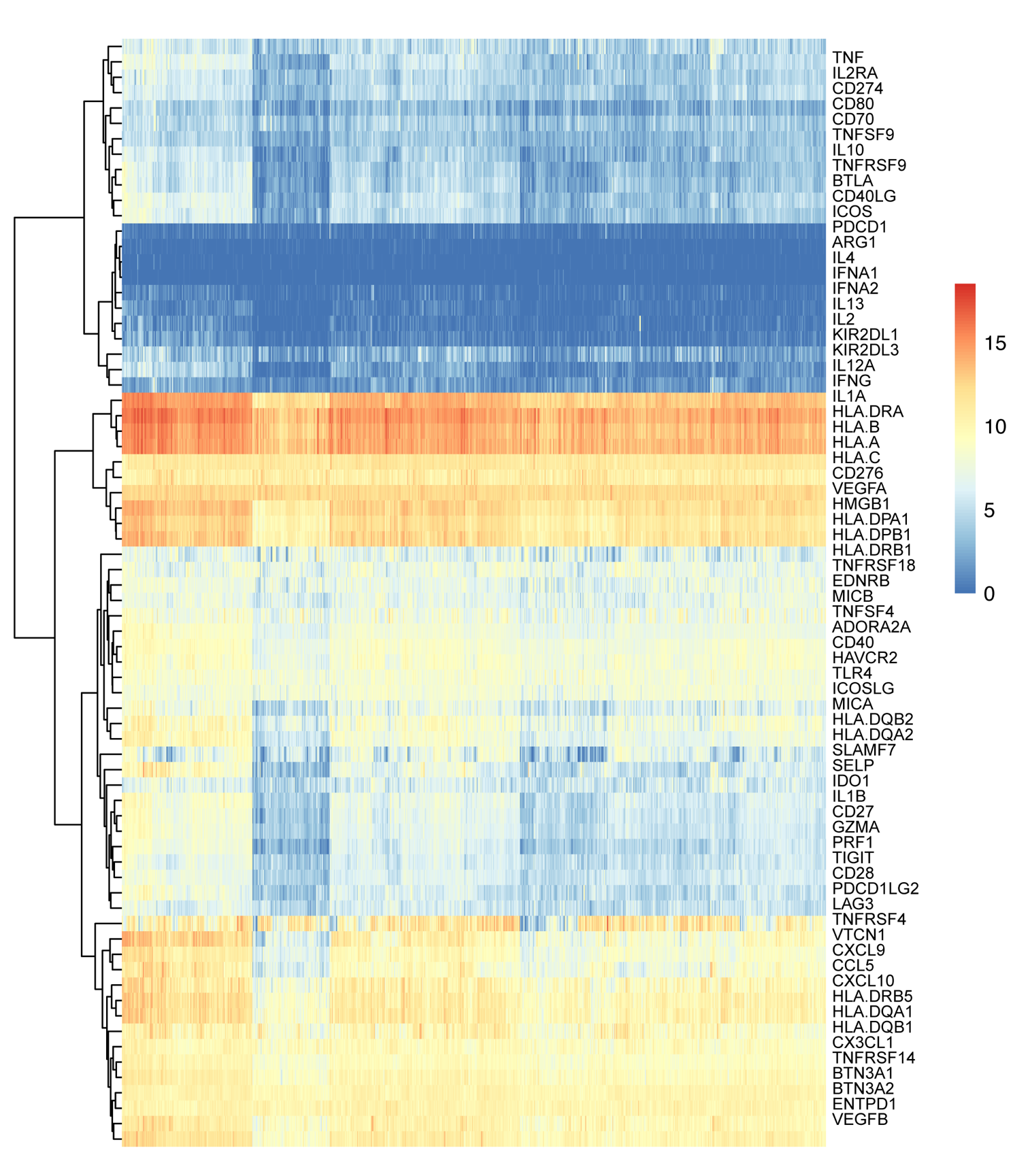
** **Supplementary Figure 5.** The gene expression of immunologic modulators in cohort.

**Supplementary Table 1.** Basic information of the included datasets

| **Dataset** | **No. of patients** | **Cohort** | **Platform** |
| --- | --- | --- | --- |
| TCGA-BRCA | 1095 | training cohort | GPL9052 |
| METABRIC | 1525 | validation cohort | GPL6947 |

**Supplementary Table 2.** The immunologic infiltrations of TCGA-BRCA patients quantitively evaluated by CIBERSORT algorithms

| **Sample** | **B cells naive** | **B cells memory** | **Plasma cells** | **T cells CD8** | **T cells CD4 naive** | **T cells CD4 memory resting** | **T cells CD4 memory activated** | **T cells follicular helper** | **T cells regulatory Tregs** | **T cells gamma delta** | **NK cells resting** | **NK cells activated** | **Monocytes** | **Macrophages M0** | **Macrophages M1** | **Macrophages M2** | **Dendritic cells resting** | **Dendritic cells activated** | **Mast cells resting** | **Mast cells activated** | **Eosinophils** | **Neutrophils** |
| --- | --- | --- | --- | --- | --- | --- | --- | --- | --- | --- | --- | --- | --- | --- | --- | --- | --- | --- | --- | --- | --- | --- |
| TCGA-3C-AAAU-01 | 0.0610 | 0.0035 | 0.0762 | 0.0294 | 0.0046 | 0.0169 | 0.0000 | 0.0533 | 0.1009 | 0.0000 | 0.0620 | 0.0000 | 0.0151 | 0.1563 | 0.0314 | 0.3669 | 0.0000 | 0.0000 | 0.0225 | 0.0000 | 0.0000 | 0.0000 |
| TCGA-3C-AALI-01 | 0.0349 | 0.0000 | 0.0000 | 0.1419 | 0.0000 | 0.0000 | 0.0000 | 0.1118 | 0.1399 | 0.0000 | 0.0171 | 0.0144 | 0.0000 | 0.3036 | 0.1019 | 0.0643 | 0.0000 | 0.0159 | 0.0518 | 0.0000 | 0.0000 | 0.0024 |
| TCGA-3C-AALJ-01 | 0.0429 | 0.0000 | 0.0278 | 0.0254 | 0.0000 | 0.0201 | 0.0000 | 0.1158 | 0.0805 | 0.0000 | 0.0403 | 0.0000 | 0.0000 | 0.4057 | 0.0689 | 0.1281 | 0.0000 | 0.0000 | 0.0445 | 0.0000 | 0.0000 | 0.0000 |
| TCGA-3C-AALK-01 | 0.0569 | 0.0000 | 0.1407 | 0.0757 | 0.0000 | 0.1939 | 0.0000 | 0.0530 | 0.0454 | 0.0000 | 0.0159 | 0.0013 | 0.0140 | 0.0000 | 0.0777 | 0.2224 | 0.0030 | 0.0000 | 0.1002 | 0.0000 | 0.0000 | 0.0000 |
| TCGA-4H-AAAK-01 | 0.0346 | 0.0000 | 0.0159 | 0.0495 | 0.0000 | 0.1150 | 0.0000 | 0.0791 | 0.0486 | 0.0000 | 0.0270 | 0.0000 | 0.0151 | 0.1196 | 0.0415 | 0.2919 | 0.0000 | 0.0000 | 0.1623 | 0.0000 | 0.0000 | 0.0000 |
| TCGA-5L-AAT0-01 | 0.0765 | 0.0000 | 0.0383 | 0.1131 | 0.0000 | 0.1264 | 0.0000 | 0.1110 | 0.0714 | 0.0000 | 0.0347 | 0.0000 | 0.0046 | 0.0720 | 0.0790 | 0.2167 | 0.0000 | 0.0000 | 0.0561 | 0.0000 | 0.0000 | 0.0000 |
| TCGA-5L-AAT1-01 | 0.0406 | 0.0000 | 0.0161 | 0.2177 | 0.0000 | 0.1044 | 0.0000 | 0.0781 | 0.0720 | 0.0000 | 0.0163 | 0.0007 | 0.0137 | 0.0000 | 0.1144 | 0.2647 | 0.0048 | 0.0000 | 0.0565 | 0.0000 | 0.0000 | 0.0000 |
| TCGA-5T-A9QA-01 | 0.0000 | 0.0083 | 0.0000 | 0.0000 | 0.0000 | 0.2927 | 0.0000 | 0.0187 | 0.0764 | 0.0000 | 0.0644 | 0.0000 | 0.0000 | 0.2569 | 0.0012 | 0.2493 | 0.0000 | 0.0000 | 0.0232 | 0.0000 | 0.0091 | 0.0000 |
| TCGA-A1-A0SB-01 | 0.0121 | 0.0000 | 0.0000 | 0.0000 | 0.0000 | 0.2883 | 0.0000 | 0.1632 | 0.0000 | 0.0000 | 0.0000 | 0.0472 | 0.0383 | 0.0000 | 0.0000 | 0.2578 | 0.0000 | 0.1084 | 0.0805 | 0.0000 | 0.0042 | 0.0000 |
| TCGA-A1-A0SD-01 | 0.0656 | 0.0000 | 0.0409 | 0.0000 | 0.0000 | 0.1868 | 0.0000 | 0.0663 | 0.0223 | 0.0000 | 0.0172 | 0.0079 | 0.0010 | 0.0960 | 0.1528 | 0.2407 | 0.0000 | 0.0000 | 0.1027 | 0.0000 | 0.0000 | 0.0000 |
| TCGA-A1-A0SE-01 | 0.0930 | 0.0000 | 0.0660 | 0.1127 | 0.0000 | 0.2077 | 0.0000 | 0.0022 | 0.0484 | 0.0000 | 0.0269 | 0.0000 | 0.0134 | 0.0000 | 0.0643 | 0.2103 | 0.0266 | 0.0000 | 0.1286 | 0.0000 | 0.0000 | 0.0000 |
| TCGA-A1-A0SF-01 | 0.0853 | 0.0000 | 0.1143 | 0.1833 | 0.0000 | 0.2052 | 0.0000 | 0.0537 | 0.0585 | 0.0000 | 0.0000 | 0.0000 | 0.0000 | 0.0000 | 0.0558 | 0.1449 | 0.0130 | 0.0166 | 0.0693 | 0.0000 | 0.0000 | 0.0000 |
| TCGA-A1-A0SG-01 | 0.1982 | 0.0965 | 0.0435 | 0.0463 | 0.0000 | 0.1502 | 0.0000 | 0.0489 | 0.0000 | 0.0000 | 0.0039 | 0.0000 | 0.0003 | 0.0077 | 0.0511 | 0.2483 | 0.0168 | 0.0005 | 0.0879 | 0.0000 | 0.0000 | 0.0000 |
| TCGA-A1-A0SH-01 | 0.0000 | 0.0000 | 0.0200 | 0.0000 | 0.0000 | 0.3122 | 0.0000 | 0.0142 | 0.0049 | 0.0000 | 0.0055 | 0.0000 | 0.0241 | 0.2312 | 0.0017 | 0.1902 | 0.0000 | 0.0000 | 0.1959 | 0.0000 | 0.0000 | 0.0000 |
| TCGA-A1-A0SI-01 | 0.0188 | 0.0000 | 0.0360 | 0.0114 | 0.0000 | 0.1563 | 0.0000 | 0.1445 | 0.0183 | 0.0000 | 0.0059 | 0.0415 | 0.0229 | 0.2483 | 0.1152 | 0.0668 | 0.0000 | 0.0000 | 0.1140 | 0.0000 | 0.0000 | 0.0000 |
| TCGA-A1-A0SJ-01 | 0.1407 | 0.0000 | 0.1567 | 0.0241 | 0.0000 | 0.1996 | 0.0000 | 0.0125 | 0.0123 | 0.0000 | 0.0233 | 0.0000 | 0.0000 | 0.0980 | 0.0672 | 0.1705 | 0.0523 | 0.0000 | 0.0419 | 0.0000 | 0.0000 | 0.0008 |
| TCGA-A1-A0SK-01 | 0.0000 | 0.0112 | 0.0241 | 0.0382 | 0.0000 | 0.3591 | 0.0000 | 0.2312 | 0.0080 | 0.0000 | 0.0000 | 0.0000 | 0.0000 | 0.2942 | 0.0087 | 0.0092 | 0.0000 | 0.0000 | 0.0161 | 0.0000 | 0.0000 | 0.0000 |
| TCGA-A1-A0SM-01 | 0.0004 | 0.0085 | 0.0002 | 0.0000 | 0.0000 | 0.2925 | 0.0000 | 0.0667 | 0.0271 | 0.0000 | 0.0114 | 0.0223 | 0.0226 | 0.1124 | 0.0148 | 0.3272 | 0.0000 | 0.0000 | 0.0931 | 0.0000 | 0.0009 | 0.0000 |
| TCGA-A1-A0SN-01 | 0.0668 | 0.0000 | 0.0174 | 0.1021 | 0.0000 | 0.2627 | 0.0057 | 0.0483 | 0.0453 | 0.0000 | 0.0374 | 0.0000 | 0.0111 | 0.0916 | 0.0651 | 0.2054 | 0.0000 | 0.0000 | 0.0411 | 0.0000 | 0.0000 | 0.0000 |
| TCGA-A1-A0SO-01 | 0.1055 | 0.0000 | 0.1225 | 0.0240 | 0.0000 | 0.1284 | 0.0059 | 0.0665 | 0.0282 | 0.0000 | 0.0018 | 0.0000 | 0.0000 | 0.2073 | 0.0617 | 0.2465 | 0.0000 | 0.0000 | 0.0016 | 0.0000 | 0.0000 | 0.0000 |
| TCGA-A1-A0SP-01 | 0.0503 | 0.0000 | 0.0299 | 0.0246 | 0.0000 | 0.1723 | 0.0000 | 0.0435 | 0.0391 | 0.0000 | 0.0000 | 0.0347 | 0.0271 | 0.2531 | 0.0562 | 0.1741 | 0.0393 | 0.0219 | 0.0339 | 0.0000 | 0.0000 | 0.0000 |
| TCGA-A1-A0SQ-01 | 0.1488 | 0.0000 | 0.0826 | 0.0686 | 0.0000 | 0.1591 | 0.0000 | 0.0531 | 0.0000 | 0.0000 | 0.0003 | 0.0078 | 0.0349 | 0.0000 | 0.0366 | 0.3198 | 0.0000 | 0.0000 | 0.0883 | 0.0000 | 0.0000 | 0.0000 |
| TCGA-A2-A04N-01 | 0.0651 | 0.0397 | 0.0337 | 0.0087 | 0.0000 | 0.0634 | 0.0000 | 0.1103 | 0.0000 | 0.0000 | 0.0403 | 0.0000 | 0.0056 | 0.1311 | 0.0452 | 0.3138 | 0.0000 | 0.0000 | 0.1431 | 0.0000 | 0.0000 | 0.0000 |
| TCGA-A2-A04P-01 | 0.0968 | 0.0479 | 0.0508 | 0.0591 | 0.0000 | 0.1090 | 0.0000 | 0.1689 | 0.0256 | 0.0000 | 0.0007 | 0.0000 | 0.0041 | 0.0000 | 0.1439 | 0.1782 | 0.0000 | 0.0824 | 0.0326 | 0.0000 | 0.0000 | 0.0000 |
| TCGA-A2-A04Q-01 | 0.0139 | 0.0478 | 0.0464 | 0.0921 | 0.0000 | 0.1195 | 0.0253 | 0.1197 | 0.0704 | 0.0000 | 0.0559 | 0.0000 | 0.0142 | 0.1340 | 0.1211 | 0.1131 | 0.0015 | 0.0017 | 0.0235 | 0.0000 | 0.0000 | 0.0000 |
| TCGA-A2-A04R-01 | 0.1151 | 0.0000 | 0.0784 | 0.0113 | 0.0000 | 0.0390 | 0.0000 | 0.0130 | 0.0124 | 0.0000 | 0.0000 | 0.0067 | 0.0000 | 0.4596 | 0.0379 | 0.1245 | 0.0000 | 0.0000 | 0.1022 | 0.0000 | 0.0000 | 0.0000 |
| TCGA-A2-A04T-01 | 0.0763 | 0.0000 | 0.0000 | 0.0534 | 0.0000 | 0.1416 | 0.0779 | 0.1365 | 0.0297 | 0.0000 | 0.0153 | 0.0000 | 0.0000 | 0.1467 | 0.0785 | 0.2118 | 0.0017 | 0.0138 | 0.0169 | 0.0000 | 0.0000 | 0.0000 |
| TCGA-A2-A04U-01 | 0.1077 | 0.0000 | 0.1221 | 0.0391 | 0.0000 | 0.0000 | 0.0000 | 0.0486 | 0.0343 | 0.0000 | 0.0129 | 0.0000 | 0.0000 | 0.3279 | 0.0446 | 0.2435 | 0.0000 | 0.0000 | 0.0192 | 0.0000 | 0.0000 | 0.0000 |
| TCGA-A2-A04V-01 | 0.0061 | 0.0058 | 0.0000 | 0.0000 | 0.0000 | 0.2461 | 0.0000 | 0.0175 | 0.0317 | 0.0000 | 0.0000 | 0.1155 | 0.0000 | 0.3242 | 0.0259 | 0.0796 | 0.0114 | 0.0000 | 0.1362 | 0.0000 | 0.0000 | 0.0000 |
| TCGA-A2-A04W-01 | 0.0793 | 0.0000 | 0.0839 | 0.0262 | 0.0000 | 0.0645 | 0.0000 | 0.0102 | 0.0115 | 0.0000 | 0.0183 | 0.0000 | 0.0000 | 0.4330 | 0.0320 | 0.1845 | 0.0000 | 0.0000 | 0.0565 | 0.0000 | 0.0000 | 0.0000 |
| TCGA-A2-A04X-01 | 0.0624 | 0.0000 | 0.0700 | 0.1116 | 0.0000 | 0.1404 | 0.0000 | 0.0818 | 0.0608 | 0.0000 | 0.0325 | 0.0000 | 0.0078 | 0.0695 | 0.0872 | 0.1932 | 0.0000 | 0.0427 | 0.0401 | 0.0000 | 0.0000 | 0.0000 |
| TCGA-A2-A04Y-01 | 0.0709 | 0.0000 | 0.0187 | 0.0373 | 0.0000 | 0.1593 | 0.0000 | 0.0681 | 0.0457 | 0.0000 | 0.0255 | 0.0000 | 0.0000 | 0.0280 | 0.0862 | 0.4098 | 0.0000 | 0.0000 | 0.0505 | 0.0000 | 0.0000 | 0.0000 |
| TCGA-A2-A0CK-01 | 0.0655 | 0.0000 | 0.0219 | 0.1373 | 0.0000 | 0.2526 | 0.0000 | 0.0392 | 0.0199 | 0.0000 | 0.0141 | 0.0000 | 0.0145 | 0.0007 | 0.0559 | 0.3326 | 0.0000 | 0.0000 | 0.0460 | 0.0000 | 0.0000 | 0.0000 |
| TCGA-A2-A0CL-01 | 0.1344 | 0.0000 | 0.0000 | 0.1045 | 0.0000 | 0.2395 | 0.0328 | 0.0779 | 0.0192 | 0.0000 | 0.0564 | 0.0000 | 0.0171 | 0.0145 | 0.1316 | 0.1401 | 0.0000 | 0.0132 | 0.0191 | 0.0000 | 0.0000 | 0.0000 |
| TCGA-A2-A0CM-01 | 0.0104 | 0.0000 | 0.0154 | 0.0719 | 0.0000 | 0.1193 | 0.0032 | 0.1194 | 0.0438 | 0.0000 | 0.0113 | 0.0460 | 0.0310 | 0.2475 | 0.1759 | 0.0546 | 0.0006 | 0.0000 | 0.0496 | 0.0000 | 0.0000 | 0.0000 |
| TCGA-A2-A0CO-01 | 0.1419 | 0.0000 | 0.0000 | 0.1250 | 0.0000 | 0.1981 | 0.0000 | 0.1530 | 0.0776 | 0.0000 | 0.0151 | 0.0000 | 0.0077 | 0.0309 | 0.0896 | 0.1554 | 0.0000 | 0.0026 | 0.0011 | 0.0020 | 0.0000 | 0.0000 |
| TCGA-A2-A0CP-01 | 0.0899 | 0.0000 | 0.0938 | 0.0765 | 0.0000 | 0.0985 | 0.0000 | 0.0505 | 0.0000 | 0.0000 | 0.0000 | 0.0110 | 0.0000 | 0.0000 | 0.0752 | 0.2825 | 0.0000 | 0.0000 | 0.2221 | 0.0000 | 0.0000 | 0.0000 |
| TCGA-A2-A0CQ-01 | 0.0738 | 0.0000 | 0.0000 | 0.0383 | 0.0000 | 0.1295 | 0.0000 | 0.0763 | 0.0167 | 0.0000 | 0.0011 | 0.0061 | 0.1225 | 0.0000 | 0.0287 | 0.4656 | 0.0000 | 0.0000 | 0.0413 | 0.0000 | 0.0000 | 0.0000 |
| TCGA-A2-A0CR-01 | 0.0662 | 0.0087 | 0.0096 | 0.1972 | 0.0000 | 0.1778 | 0.0000 | 0.1020 | 0.0674 | 0.0000 | 0.0000 | 0.0316 | 0.0154 | 0.1140 | 0.0664 | 0.1157 | 0.0000 | 0.0000 | 0.0280 | 0.0000 | 0.0000 | 0.0000 |
| TCGA-A2-A0CS-01 | 0.0418 | 0.0000 | 0.0005 | 0.0329 | 0.0000 | 0.0833 | 0.0000 | 0.0542 | 0.0352 | 0.0000 | 0.0185 | 0.0000 | 0.0089 | 0.0000 | 0.0012 | 0.2590 | 0.2391 | 0.0789 | 0.1403 | 0.0000 | 0.0000 | 0.0063 |
| TCGA-A2-A0CT-01 | 0.0448 | 0.0000 | 0.0666 | 0.0000 | 0.0000 | 0.1356 | 0.0000 | 0.0507 | 0.0007 | 0.0000 | 0.0000 | 0.0284 | 0.0000 | 0.0110 | 0.0232 | 0.4270 | 0.0000 | 0.0000 | 0.2122 | 0.0000 | 0.0000 | 0.0000 |
| TCGA-A2-A0CU-01 | 0.1077 | 0.0000 | 0.0539 | 0.0360 | 0.0000 | 0.1006 | 0.0000 | 0.1036 | 0.0361 | 0.0000 | 0.0340 | 0.0171 | 0.0384 | 0.0000 | 0.0696 | 0.2864 | 0.0000 | 0.0000 | 0.1166 | 0.0000 | 0.0000 | 0.0000 |
| TCGA-A2-A0CV-01 | 0.1138 | 0.0000 | 0.0494 | 0.0866 | 0.0000 | 0.1498 | 0.0000 | 0.0961 | 0.0117 | 0.0228 | 0.0000 | 0.0000 | 0.0194 | 0.0000 | 0.0762 | 0.3346 | 0.0003 | 0.0102 | 0.0291 | 0.0000 | 0.0000 | 0.0000 |
| TCGA-A2-A0CW-01 | 0.0760 | 0.0000 | 0.0184 | 0.0294 | 0.0000 | 0.1700 | 0.0000 | 0.1114 | 0.0390 | 0.0000 | 0.0370 | 0.0000 | 0.0031 | 0.1420 | 0.0866 | 0.1910 | 0.0000 | 0.0290 | 0.0670 | 0.0000 | 0.0000 | 0.0000 |
| TCGA-A2-A0CX-01 | 0.0302 | 0.0000 | 0.0175 | 0.0000 | 0.0000 | 0.1881 | 0.0000 | 0.0767 | 0.0294 | 0.0000 | 0.0032 | 0.0081 | 0.0000 | 0.2662 | 0.0733 | 0.2506 | 0.0000 | 0.0000 | 0.0566 | 0.0000 | 0.0000 | 0.0000 |
| TCGA-A2-A0CY-01 | 0.1157 | 0.0000 | 0.0083 | 0.0584 | 0.0000 | 0.0000 | 0.0000 | 0.1448 | 0.0080 | 0.0000 | 0.0134 | 0.0073 | 0.0000 | 0.5152 | 0.0000 | 0.1159 | 0.0000 | 0.0000 | 0.0129 | 0.0000 | 0.0000 | 0.0000 |
| TCGA-A2-A0CZ-01 | 0.0113 | 0.0000 | 0.0392 | 0.0000 | 0.0000 | 0.1497 | 0.0000 | 0.0548 | 0.0448 | 0.0000 | 0.0043 | 0.0121 | 0.0000 | 0.3939 | 0.0693 | 0.0962 | 0.0000 | 0.0000 | 0.1244 | 0.0000 | 0.0000 | 0.0000 |
| TCGA-A2-A0D0-01 | 0.0354 | 0.0000 | 0.0000 | 0.0683 | 0.0000 | 0.1344 | 0.0000 | 0.1103 | 0.0276 | 0.0000 | 0.0547 | 0.0021 | 0.0000 | 0.2849 | 0.1001 | 0.1627 | 0.0015 | 0.0000 | 0.0179 | 0.0000 | 0.0000 | 0.0000 |
| TCGA-A2-A0D1-01 | 0.0673 | 0.0000 | 0.0354 | 0.0832 | 0.0000 | 0.0802 | 0.0000 | 0.0638 | 0.0460 | 0.0000 | 0.0197 | 0.0000 | 0.0000 | 0.4188 | 0.0577 | 0.1268 | 0.0000 | 0.0000 | 0.0012 | 0.0000 | 0.0000 | 0.0000 |
| TCGA-A2-A0D2-01 | 0.0652 | 0.0000 | 0.0258 | 0.0801 | 0.0000 | 0.0797 | 0.0284 | 0.1094 | 0.0151 | 0.0000 | 0.0098 | 0.0051 | 0.0200 | 0.2605 | 0.0917 | 0.1599 | 0.0000 | 0.0111 | 0.0382 | 0.0000 | 0.0000 | 0.0000 |
| TCGA-A2-A0D3-01 | 0.0847 | 0.0051 | 0.0872 | 0.0901 | 0.0000 | 0.1931 | 0.0000 | 0.1439 | 0.0000 | 0.0473 | 0.0000 | 0.0040 | 0.0000 | 0.0000 | 0.0799 | 0.2306 | 0.0000 | 0.0092 | 0.0248 | 0.0000 | 0.0000 | 0.0000 |
| TCGA-A2-A0D4-01 | 0.1312 | 0.0000 | 0.0613 | 0.0000 | 0.0000 | 0.1048 | 0.0000 | 0.0637 | 0.0159 | 0.0000 | 0.0000 | 0.0413 | 0.0052 | 0.1195 | 0.0256 | 0.2620 | 0.0000 | 0.0000 | 0.1696 | 0.0000 | 0.0000 | 0.0000 |
| TCGA-A2-A0EM-01 | 0.0015 | 0.0097 | 0.0091 | 0.0000 | 0.0000 | 0.2486 | 0.0000 | 0.0525 | 0.0529 | 0.0000 | 0.0727 | 0.0000 | 0.0319 | 0.1054 | 0.0000 | 0.2130 | 0.0029 | 0.0112 | 0.1883 | 0.0000 | 0.0000 | 0.0000 |
| TCGA-A2-A0EN-01 | 0.0890 | 0.0000 | 0.0000 | 0.0931 | 0.0000 | 0.2007 | 0.0000 | 0.1064 | 0.0619 | 0.0000 | 0.0365 | 0.0000 | 0.0127 | 0.0206 | 0.0788 | 0.2287 | 0.0180 | 0.0000 | 0.0535 | 0.0000 | 0.0000 | 0.0000 |
| TCGA-A2-A0EO-01 | 0.0642 | 0.0000 | 0.0668 | 0.0595 | 0.0000 | 0.2783 | 0.0000 | 0.0207 | 0.0022 | 0.0000 | 0.0177 | 0.0000 | 0.0095 | 0.0858 | 0.0676 | 0.2456 | 0.0041 | 0.0000 | 0.0779 | 0.0000 | 0.0000 | 0.0000 |
| TCGA-A2-A0EP-01 | 0.0616 | 0.0000 | 0.0000 | 0.0238 | 0.0000 | 0.1442 | 0.0044 | 0.0868 | 0.0741 | 0.0000 | 0.0272 | 0.0000 | 0.0286 | 0.3196 | 0.0271 | 0.1702 | 0.0138 | 0.0000 | 0.0187 | 0.0000 | 0.0000 | 0.0000 |
| TCGA-A2-A0EQ-01 | 0.0893 | 0.0000 | 0.0000 | 0.0966 | 0.0000 | 0.1588 | 0.0000 | 0.0750 | 0.0778 | 0.0000 | 0.0103 | 0.0207 | 0.0000 | 0.2174 | 0.1238 | 0.0971 | 0.0000 | 0.0000 | 0.0333 | 0.0000 | 0.0000 | 0.0000 |
| TCGA-A2-A0ER-01 | 0.0202 | 0.0000 | 0.0900 | 0.0000 | 0.0000 | 0.2313 | 0.0000 | 0.0467 | 0.0008 | 0.0000 | 0.0097 | 0.0116 | 0.0047 | 0.0481 | 0.0479 | 0.2612 | 0.0000 | 0.0130 | 0.2142 | 0.0000 | 0.0000 | 0.0005 |
| TCGA-A2-A0ES-01 | 0.0799 | 0.0000 | 0.0509 | 0.0597 | 0.0000 | 0.2147 | 0.0000 | 0.0142 | 0.0000 | 0.0000 | 0.0226 | 0.0000 | 0.0162 | 0.0000 | 0.0661 | 0.3703 | 0.0000 | 0.0305 | 0.0747 | 0.0000 | 0.0000 | 0.0000 |
| TCGA-A2-A0ET-01 | 0.1150 | 0.0000 | 0.0037 | 0.0883 | 0.0000 | 0.1967 | 0.0000 | 0.0510 | 0.0244 | 0.0000 | 0.0278 | 0.0000 | 0.0517 | 0.0000 | 0.0363 | 0.3441 | 0.0452 | 0.0140 | 0.0000 | 0.0018 | 0.0000 | 0.0000 |
| TCGA-A2-A0EU-01 | 0.0914 | 0.0000 | 0.0000 | 0.0103 | 0.0000 | 0.1498 | 0.0000 | 0.0916 | 0.0000 | 0.0181 | 0.0000 | 0.0038 | 0.0000 | 0.1426 | 0.0702 | 0.1807 | 0.0000 | 0.0421 | 0.1928 | 0.0000 | 0.0000 | 0.0066 |
| TCGA-A2-A0EV-01 | 0.0607 | 0.0000 | 0.0298 | 0.0407 | 0.0000 | 0.1569 | 0.0046 | 0.0334 | 0.0233 | 0.0000 | 0.0310 | 0.0000 | 0.0000 | 0.3736 | 0.0275 | 0.1767 | 0.0000 | 0.0056 | 0.0361 | 0.0000 | 0.0000 | 0.0000 |
| TCGA-A2-A0EW-01 | 0.0067 | 0.0000 | 0.0153 | 0.2071 | 0.0000 | 0.0834 | 0.0000 | 0.1455 | 0.0000 | 0.0000 | 0.0000 | 0.0224 | 0.0416 | 0.0655 | 0.0124 | 0.2266 | 0.0000 | 0.0148 | 0.1589 | 0.0000 | 0.0000 | 0.0000 |
| TCGA-A2-A0EX-01 | 0.1161 | 0.0000 | 0.0396 | 0.0000 | 0.0000 | 0.1451 | 0.0000 | 0.0666 | 0.0432 | 0.0000 | 0.0313 | 0.0000 | 0.0106 | 0.2208 | 0.0435 | 0.1703 | 0.0000 | 0.0000 | 0.1131 | 0.0000 | 0.0000 | 0.0000 |
| TCGA-A2-A0EY-01 | 0.1103 | 0.0000 | 0.0186 | 0.0810 | 0.0000 | 0.1844 | 0.0000 | 0.1263 | 0.0000 | 0.0000 | 0.0289 | 0.0000 | 0.0139 | 0.0078 | 0.0737 | 0.2966 | 0.0062 | 0.0000 | 0.0522 | 0.0000 | 0.0000 | 0.0000 |
| TCGA-A2-A0ST-01 | 0.0488 | 0.0578 | 0.0399 | 0.1270 | 0.0000 | 0.1346 | 0.0008 | 0.1009 | 0.0543 | 0.0000 | 0.0187 | 0.0000 | 0.0128 | 0.1043 | 0.1681 | 0.0556 | 0.0338 | 0.0130 | 0.0295 | 0.0000 | 0.0000 | 0.0000 |
| TCGA-A2-A0SU-01 | 0.1064 | 0.0000 | 0.0712 | 0.0604 | 0.0000 | 0.1301 | 0.0000 | 0.0343 | 0.0565 | 0.0000 | 0.0145 | 0.0000 | 0.0070 | 0.1446 | 0.0386 | 0.2294 | 0.0082 | 0.0057 | 0.0930 | 0.0000 | 0.0000 | 0.0000 |
| TCGA-A2-A0SV-01 | 0.0283 | 0.0000 | 0.0000 | 0.0000 | 0.0000 | 0.0904 | 0.0000 | 0.0650 | 0.0650 | 0.0000 | 0.0360 | 0.0000 | 0.0000 | 0.2340 | 0.0523 | 0.3951 | 0.0018 | 0.0000 | 0.0250 | 0.0000 | 0.0000 | 0.0072 |
| TCGA-A2-A0SW-01 | 0.0834 | 0.0000 | 0.0898 | 0.1317 | 0.0000 | 0.0813 | 0.0047 | 0.1080 | 0.0252 | 0.0000 | 0.0202 | 0.0069 | 0.0162 | 0.1562 | 0.0736 | 0.1203 | 0.0000 | 0.0000 | 0.0826 | 0.0000 | 0.0000 | 0.0000 |
| TCGA-A2-A0SX-01 | 0.0225 | 0.0177 | 0.0319 | 0.0245 | 0.0000 | 0.1265 | 0.0000 | 0.1057 | 0.0640 | 0.0000 | 0.0234 | 0.0000 | 0.0000 | 0.3720 | 0.0873 | 0.0789 | 0.0000 | 0.0000 | 0.0456 | 0.0000 | 0.0000 | 0.0000 |
| TCGA-A2-A0SY-01 | 0.1694 | 0.0000 | 0.0700 | 0.0627 | 0.0000 | 0.1947 | 0.0000 | 0.0740 | 0.0108 | 0.0000 | 0.0000 | 0.0000 | 0.0162 | 0.0093 | 0.0689 | 0.2279 | 0.0109 | 0.0000 | 0.0851 | 0.0000 | 0.0000 | 0.0000 |
| TCGA-A2-A0T0-01 | 0.0735 | 0.0000 | 0.0140 | 0.0415 | 0.0000 | 0.1734 | 0.0000 | 0.0548 | 0.0299 | 0.0000 | 0.0504 | 0.0000 | 0.0000 | 0.2510 | 0.1027 | 0.2017 | 0.0000 | 0.0000 | 0.0000 | 0.0073 | 0.0000 | 0.0000 |
| TCGA-A2-A0T1-01 | 0.0297 | 0.0000 | 0.0278 | 0.0848 | 0.0000 | 0.1826 | 0.0000 | 0.0572 | 0.0788 | 0.0000 | 0.0562 | 0.0000 | 0.0000 | 0.2154 | 0.0831 | 0.1739 | 0.0000 | 0.0000 | 0.0102 | 0.0000 | 0.0000 | 0.0000 |
| TCGA-A2-A0T2-01 | 0.0666 | 0.0000 | 0.1353 | 0.0900 | 0.0000 | 0.1049 | 0.0000 | 0.1040 | 0.0142 | 0.0000 | 0.0000 | 0.0236 | 0.0061 | 0.0933 | 0.0887 | 0.2517 | 0.0000 | 0.0000 | 0.0167 | 0.0000 | 0.0000 | 0.0048 |
| TCGA-A2-A0T3-01 | 0.1014 | 0.0000 | 0.0170 | 0.0398 | 0.0000 | 0.0536 | 0.0000 | 0.0845 | 0.0310 | 0.0000 | 0.0292 | 0.0000 | 0.0000 | 0.3197 | 0.0648 | 0.2421 | 0.0000 | 0.0000 | 0.0167 | 0.0000 | 0.0000 | 0.0000 |
| TCGA-A2-A0T4-01 | 0.0553 | 0.0000 | 0.0185 | 0.0582 | 0.0000 | 0.0813 | 0.0000 | 0.0534 | 0.0296 | 0.0000 | 0.0000 | 0.0147 | 0.0000 | 0.2184 | 0.0624 | 0.2256 | 0.0000 | 0.0000 | 0.1827 | 0.0000 | 0.0000 | 0.0000 |
| TCGA-A2-A0T5-01 | 0.0864 | 0.0000 | 0.0362 | 0.0000 | 0.0000 | 0.0356 | 0.0000 | 0.1142 | 0.0531 | 0.0000 | 0.0599 | 0.0000 | 0.0056 | 0.2067 | 0.0272 | 0.2866 | 0.0000 | 0.0487 | 0.0399 | 0.0000 | 0.0000 | 0.0000 |
| TCGA-A2-A0T6-01 | 0.1431 | 0.0000 | 0.0040 | 0.0453 | 0.0000 | 0.2083 | 0.0000 | 0.0785 | 0.0155 | 0.0000 | 0.0037 | 0.0000 | 0.0004 | 0.0144 | 0.0587 | 0.2571 | 0.0000 | 0.0000 | 0.1706 | 0.0000 | 0.0000 | 0.0004 |
| TCGA-A2-A0T7-01 | 0.0716 | 0.0000 | 0.0000 | 0.0510 | 0.0000 | 0.2009 | 0.0000 | 0.0324 | 0.0429 | 0.0000 | 0.0225 | 0.0000 | 0.0000 | 0.1055 | 0.0336 | 0.3645 | 0.0000 | 0.0345 | 0.0406 | 0.0000 | 0.0000 | 0.0000 |
| TCGA-A2-A0YC-01 | 0.1088 | 0.0000 | 0.0228 | 0.0564 | 0.0000 | 0.0849 | 0.0000 | 0.0367 | 0.0246 | 0.0000 | 0.0272 | 0.0000 | 0.0051 | 0.0823 | 0.0382 | 0.2838 | 0.0720 | 0.0005 | 0.1348 | 0.0000 | 0.0000 | 0.0218 |
| TCGA-A2-A0YD-01 | 0.0540 | 0.0000 | 0.0130 | 0.0409 | 0.0000 | 0.1263 | 0.0000 | 0.0756 | 0.0558 | 0.0000 | 0.0272 | 0.0000 | 0.0000 | 0.1341 | 0.0976 | 0.2878 | 0.0002 | 0.0000 | 0.0871 | 0.0000 | 0.0000 | 0.0004 |
| TCGA-A2-A0YE-01 | 0.0817 | 0.0000 | 0.2227 | 0.0080 | 0.0000 | 0.0808 | 0.0000 | 0.0799 | 0.0397 | 0.0000 | 0.0058 | 0.0259 | 0.0000 | 0.1426 | 0.0576 | 0.1595 | 0.0000 | 0.0668 | 0.0289 | 0.0000 | 0.0000 | 0.0000 |
| TCGA-A2-A0YF-01 | 0.0004 | 0.0000 | 0.0140 | 0.0000 | 0.0000 | 0.2027 | 0.0000 | 0.0269 | 0.0344 | 0.0000 | 0.0000 | 0.0267 | 0.0000 | 0.3520 | 0.0176 | 0.2306 | 0.0026 | 0.0000 | 0.0713 | 0.0000 | 0.0210 | 0.0000 |
| TCGA-A2-A0YG-01 | 0.0439 | 0.0000 | 0.0181 | 0.0000 | 0.0000 | 0.1991 | 0.0000 | 0.0605 | 0.0409 | 0.0000 | 0.0168 | 0.0000 | 0.0000 | 0.2296 | 0.0897 | 0.2464 | 0.0000 | 0.0000 | 0.0552 | 0.0000 | 0.0000 | 0.0000 |
| TCGA-A2-A0YH-01 | 0.0209 | 0.0000 | 0.0564 | 0.0188 | 0.0000 | 0.1908 | 0.0048 | 0.0582 | 0.0095 | 0.0000 | 0.0263 | 0.0000 | 0.0186 | 0.0862 | 0.0573 | 0.2897 | 0.0000 | 0.0283 | 0.1262 | 0.0000 | 0.0000 | 0.0078 |
| TCGA-A2-A0YI-01 | 0.0901 | 0.0000 | 0.0471 | 0.0160 | 0.0000 | 0.1971 | 0.0000 | 0.0262 | 0.0306 | 0.0000 | 0.0358 | 0.0000 | 0.0000 | 0.1520 | 0.0675 | 0.3132 | 0.0000 | 0.0000 | 0.0245 | 0.0000 | 0.0000 | 0.0000 |
| TCGA-A2-A0YJ-01 | 0.0000 | 0.0032 | 0.0151 | 0.0000 | 0.0000 | 0.2656 | 0.0000 | 0.0700 | 0.0109 | 0.0000 | 0.0533 | 0.0000 | 0.0000 | 0.5428 | 0.0028 | 0.0214 | 0.0000 | 0.0000 | 0.0116 | 0.0000 | 0.0000 | 0.0032 |
| TCGA-A2-A0YK-01 | 0.1886 | 0.0000 | 0.0216 | 0.1433 | 0.0000 | 0.1591 | 0.0051 | 0.1383 | 0.0255 | 0.0007 | 0.0025 | 0.0000 | 0.0067 | 0.0044 | 0.1026 | 0.1692 | 0.0000 | 0.0000 | 0.0325 | 0.0000 | 0.0000 | 0.0000 |
| TCGA-A2-A0YL-01 | 0.1325 | 0.0000 | 0.0535 | 0.1604 | 0.0000 | 0.1040 | 0.0000 | 0.0451 | 0.0072 | 0.0000 | 0.0219 | 0.0000 | 0.0209 | 0.0052 | 0.0956 | 0.2502 | 0.0112 | 0.0000 | 0.0923 | 0.0000 | 0.0000 | 0.0000 |
| TCGA-A2-A0YM-01 | 0.0276 | 0.0000 | 0.0000 | 0.0093 | 0.0000 | 0.0958 | 0.0000 | 0.0406 | 0.0294 | 0.0000 | 0.0143 | 0.0000 | 0.0022 | 0.5962 | 0.0801 | 0.0487 | 0.0000 | 0.0558 | 0.0000 | 0.0000 | 0.0000 | 0.0000 |
| TCGA-A2-A0YT-01 | 0.0015 | 0.0000 | 0.0436 | 0.0291 | 0.0000 | 0.2023 | 0.0000 | 0.0221 | 0.0095 | 0.0000 | 0.0076 | 0.0152 | 0.0797 | 0.0164 | 0.0000 | 0.2682 | 0.0000 | 0.1204 | 0.1721 | 0.0000 | 0.0122 | 0.0000 |
| TCGA-A2-A1FV-01 | 0.1547 | 0.0000 | 0.0000 | 0.0000 | 0.0000 | 0.1899 | 0.0000 | 0.0627 | 0.0000 | 0.0000 | 0.0000 | 0.0253 | 0.0000 | 0.0000 | 0.0142 | 0.3015 | 0.0000 | 0.0044 | 0.2472 | 0.0000 | 0.0000 | 0.0000 |
| TCGA-A2-A1FW-01 | 0.1439 | 0.0000 | 0.0777 | 0.0233 | 0.0000 | 0.2880 | 0.0000 | 0.0175 | 0.0000 | 0.0000 | 0.0000 | 0.0000 | 0.0000 | 0.0142 | 0.0648 | 0.2979 | 0.0000 | 0.0000 | 0.0728 | 0.0000 | 0.0000 | 0.0000 |
| TCGA-A2-A1FX-01 | 0.0587 | 0.0000 | 0.0021 | 0.0697 | 0.0000 | 0.1276 | 0.0000 | 0.0367 | 0.0070 | 0.0000 | 0.0000 | 0.0000 | 0.0535 | 0.0000 | 0.0629 | 0.4225 | 0.0000 | 0.0000 | 0.1578 | 0.0000 | 0.0000 | 0.0014 |
| TCGA-A2-A1FZ-01 | 0.0791 | 0.0000 | 0.0000 | 0.0264 | 0.0000 | 0.1811 | 0.0000 | 0.0375 | 0.0000 | 0.0000 | 0.0099 | 0.0000 | 0.0368 | 0.0139 | 0.0592 | 0.4460 | 0.0319 | 0.0000 | 0.0748 | 0.0000 | 0.0000 | 0.0033 |
| TCGA-A2-A1G0-01 | 0.0182 | 0.0000 | 0.1617 | 0.0454 | 0.0000 | 0.1903 | 0.0000 | 0.0780 | 0.0032 | 0.0000 | 0.0775 | 0.0000 | 0.0559 | 0.0325 | 0.0484 | 0.1703 | 0.0181 | 0.0000 | 0.1005 | 0.0000 | 0.0000 | 0.0000 |
| TCGA-A2-A1G1-01 | 0.0117 | 0.0000 | 0.0554 | 0.0000 | 0.0000 | 0.1277 | 0.0185 | 0.1247 | 0.0000 | 0.0373 | 0.0000 | 0.0120 | 0.0000 | 0.1193 | 0.2310 | 0.1815 | 0.0007 | 0.0000 | 0.0801 | 0.0000 | 0.0000 | 0.0000 |
| TCGA-A2-A1G4-01 | 0.0977 | 0.0326 | 0.1011 | 0.0508 | 0.0000 | 0.0924 | 0.0000 | 0.1148 | 0.0000 | 0.0000 | 0.0000 | 0.0014 | 0.0000 | 0.0352 | 0.0680 | 0.3396 | 0.0000 | 0.0000 | 0.0620 | 0.0000 | 0.0000 | 0.0043 |
| TCGA-A2-A1G6-01 | 0.0923 | 0.0000 | 0.0749 | 0.0721 | 0.0000 | 0.0905 | 0.0000 | 0.0515 | 0.0254 | 0.0000 | 0.0106 | 0.0000 | 0.0180 | 0.0000 | 0.0024 | 0.3923 | 0.0444 | 0.0027 | 0.1230 | 0.0000 | 0.0000 | 0.0000 |
| TCGA-A2-A259-01 | 0.1247 | 0.0000 | 0.0000 | 0.0139 | 0.0000 | 0.2003 | 0.0000 | 0.0208 | 0.0382 | 0.0000 | 0.0279 | 0.0000 | 0.0365 | 0.0261 | 0.0462 | 0.1464 | 0.0004 | 0.0464 | 0.2706 | 0.0000 | 0.0000 | 0.0016 |
| TCGA-A2-A25A-01 | 0.1087 | 0.0000 | 0.0184 | 0.0900 | 0.0000 | 0.1815 | 0.0000 | 0.0303 | 0.0270 | 0.0000 | 0.0136 | 0.0000 | 0.0043 | 0.0000 | 0.0991 | 0.2969 | 0.0084 | 0.0000 | 0.1218 | 0.0000 | 0.0000 | 0.0000 |
| TCGA-A2-A25B-01 | 0.0795 | 0.0000 | 0.0176 | 0.0362 | 0.0000 | 0.2366 | 0.0000 | 0.0413 | 0.0100 | 0.0000 | 0.0163 | 0.0000 | 0.0000 | 0.0589 | 0.0880 | 0.3403 | 0.0000 | 0.0000 | 0.0625 | 0.0000 | 0.0000 | 0.0128 |
| TCGA-A2-A25C-01 | 0.1502 | 0.0000 | 0.0394 | 0.0000 | 0.0000 | 0.1903 | 0.0000 | 0.0590 | 0.0000 | 0.0168 | 0.0071 | 0.0000 | 0.0062 | 0.0464 | 0.0425 | 0.2816 | 0.0000 | 0.0773 | 0.0833 | 0.0000 | 0.0000 | 0.0000 |
| TCGA-A2-A25D-01 | 0.0947 | 0.0000 | 0.0226 | 0.0356 | 0.0000 | 0.1397 | 0.0125 | 0.1162 | 0.0501 | 0.0000 | 0.0985 | 0.0000 | 0.0264 | 0.0000 | 0.1363 | 0.1889 | 0.0000 | 0.0000 | 0.0786 | 0.0000 | 0.0000 | 0.0000 |
| TCGA-A2-A25E-01 | 0.2049 | 0.0000 | 0.0280 | 0.0000 | 0.0000 | 0.1620 | 0.0012 | 0.0852 | 0.0186 | 0.0000 | 0.0000 | 0.0008 | 0.0000 | 0.0370 | 0.0844 | 0.2957 | 0.0079 | 0.0000 | 0.0742 | 0.0000 | 0.0000 | 0.0000 |
| TCGA-A2-A25F-01 | 0.0378 | 0.0219 | 0.0000 | 0.0378 | 0.0000 | 0.2819 | 0.0471 | 0.0530 | 0.0072 | 0.0219 | 0.0187 | 0.0000 | 0.0000 | 0.1947 | 0.1109 | 0.1358 | 0.0000 | 0.0041 | 0.0274 | 0.0000 | 0.0000 | 0.0000 |
| TCGA-A2-A3KC-01 | 0.1168 | 0.0000 | 0.0719 | 0.0195 | 0.0000 | 0.1463 | 0.0000 | 0.0376 | 0.0208 | 0.0000 | 0.0259 | 0.0000 | 0.0000 | 0.1015 | 0.0462 | 0.3452 | 0.0000 | 0.0014 | 0.0668 | 0.0000 | 0.0000 | 0.0000 |
| TCGA-A2-A3KD-01 | 0.0003 | 0.0000 | 0.0664 | 0.0000 | 0.0000 | 0.2796 | 0.0000 | 0.0658 | 0.0237 | 0.0000 | 0.1181 | 0.0000 | 0.0584 | 0.0883 | 0.0000 | 0.1993 | 0.0000 | 0.0000 | 0.0961 | 0.0000 | 0.0039 | 0.0000 |
| TCGA-A2-A3XS-01 | 0.1297 | 0.0000 | 0.0000 | 0.0921 | 0.0000 | 0.1239 | 0.0000 | 0.1373 | 0.0847 | 0.0000 | 0.0268 | 0.0000 | 0.0390 | 0.0882 | 0.0952 | 0.1358 | 0.0000 | 0.0135 | 0.0338 | 0.0000 | 0.0000 | 0.0000 |
| TCGA-A2-A3XT-01 | 0.0000 | 0.0024 | 0.0000 | 0.0330 | 0.0000 | 0.1179 | 0.0026 | 0.0210 | 0.0120 | 0.0000 | 0.0207 | 0.0000 | 0.0004 | 0.5823 | 0.0553 | 0.1238 | 0.0000 | 0.0000 | 0.0286 | 0.0000 | 0.0000 | 0.0000 |
| TCGA-A2-A3XU-01 | 0.1067 | 0.0000 | 0.1303 | 0.0480 | 0.0000 | 0.0000 | 0.0000 | 0.1700 | 0.0157 | 0.0000 | 0.0000 | 0.0087 | 0.0000 | 0.2810 | 0.0006 | 0.2348 | 0.0000 | 0.0000 | 0.0042 | 0.0000 | 0.0000 | 0.0000 |
| TCGA-A2-A3XV-01 | 0.0401 | 0.0000 | 0.0067 | 0.0295 | 0.0000 | 0.1129 | 0.0000 | 0.0487 | 0.0144 | 0.0000 | 0.0060 | 0.0107 | 0.0000 | 0.2183 | 0.0344 | 0.4635 | 0.0000 | 0.0000 | 0.0148 | 0.0000 | 0.0000 | 0.0000 |
| TCGA-A2-A3XW-01 | 0.0241 | 0.0000 | 0.0000 | 0.0918 | 0.0000 | 0.1516 | 0.0000 | 0.0053 | 0.0633 | 0.0000 | 0.0267 | 0.0162 | 0.0219 | 0.2268 | 0.0085 | 0.1909 | 0.0552 | 0.0185 | 0.0991 | 0.0000 | 0.0000 | 0.0000 |
| TCGA-A2-A3XX-01 | 0.0744 | 0.0000 | 0.0607 | 0.0512 | 0.0000 | 0.0492 | 0.0000 | 0.1132 | 0.0254 | 0.0000 | 0.0348 | 0.0000 | 0.0000 | 0.3805 | 0.0725 | 0.1221 | 0.0000 | 0.0000 | 0.0161 | 0.0000 | 0.0000 | 0.0000 |
| TCGA-A2-A3XY-01 | 0.0236 | 0.0016 | 0.0000 | 0.0078 | 0.0000 | 0.1031 | 0.0000 | 0.1120 | 0.0405 | 0.0000 | 0.0359 | 0.0009 | 0.0000 | 0.3884 | 0.1506 | 0.1010 | 0.0000 | 0.0000 | 0.0347 | 0.0000 | 0.0000 | 0.0000 |
| TCGA-A2-A3XZ-01 | 0.1244 | 0.1291 | 0.0000 | 0.0562 | 0.0000 | 0.1785 | 0.0000 | 0.0969 | 0.0849 | 0.0000 | 0.0176 | 0.0000 | 0.0000 | 0.0408 | 0.0588 | 0.1769 | 0.0072 | 0.0112 | 0.0174 | 0.0000 | 0.0000 | 0.0000 |
| TCGA-A2-A3Y0-01 | 0.0045 | 0.0137 | 0.0078 | 0.1485 | 0.0000 | 0.1339 | 0.0000 | 0.1578 | 0.0747 | 0.0000 | 0.0008 | 0.0475 | 0.0040 | 0.1631 | 0.0997 | 0.1173 | 0.0000 | 0.0017 | 0.0250 | 0.0000 | 0.0000 | 0.0000 |
| TCGA-A2-A4RW-01 | 0.0907 | 0.0000 | 0.0245 | 0.1144 | 0.0000 | 0.0872 | 0.0000 | 0.0508 | 0.0443 | 0.0000 | 0.0185 | 0.0096 | 0.0130 | 0.0000 | 0.0550 | 0.2286 | 0.0000 | 0.0000 | 0.2635 | 0.0000 | 0.0000 | 0.0000 |
| TCGA-A2-A4RX-01 | 0.2414 | 0.0000 | 0.0052 | 0.0217 | 0.0000 | 0.1792 | 0.0000 | 0.1223 | 0.0117 | 0.0000 | 0.0176 | 0.0000 | 0.0000 | 0.1932 | 0.0557 | 0.1198 | 0.0000 | 0.0110 | 0.0213 | 0.0000 | 0.0000 | 0.0000 |
| TCGA-A2-A4RY-01 | 0.2032 | 0.0000 | 0.0320 | 0.0747 | 0.0000 | 0.0880 | 0.0000 | 0.1709 | 0.0000 | 0.0000 | 0.0196 | 0.0000 | 0.0286 | 0.0247 | 0.0446 | 0.2079 | 0.0036 | 0.0120 | 0.0903 | 0.0000 | 0.0000 | 0.0000 |
| TCGA-A2-A4S0-01 | 0.0000 | 0.0327 | 0.0499 | 0.0000 | 0.0000 | 0.4012 | 0.0000 | 0.0607 | 0.0355 | 0.0000 | 0.0337 | 0.0000 | 0.0684 | 0.0781 | 0.0000 | 0.0716 | 0.0071 | 0.0000 | 0.1602 | 0.0000 | 0.0008 | 0.0000 |
| TCGA-A2-A4S1-01 | 0.0000 | 0.0032 | 0.0000 | 0.0349 | 0.0000 | 0.0347 | 0.0000 | 0.0112 | 0.0044 | 0.0000 | 0.0275 | 0.0000 | 0.0000 | 0.1669 | 0.0201 | 0.6555 | 0.0000 | 0.0000 | 0.0408 | 0.0000 | 0.0000 | 0.0008 |
| TCGA-A2-A4S2-01 | 0.0736 | 0.0000 | 0.0039 | 0.0799 | 0.0000 | 0.1571 | 0.0000 | 0.0716 | 0.0440 | 0.0000 | 0.0474 | 0.0047 | 0.0014 | 0.2698 | 0.0584 | 0.1279 | 0.0000 | 0.0112 | 0.0492 | 0.0000 | 0.0000 | 0.0000 |
| TCGA-A2-A4S3-01 | 0.0181 | 0.0028 | 0.0009 | 0.0439 | 0.0000 | 0.0095 | 0.0000 | 0.1050 | 0.1060 | 0.0000 | 0.0329 | 0.0342 | 0.0243 | 0.3684 | 0.0896 | 0.1093 | 0.0000 | 0.0000 | 0.0552 | 0.0000 | 0.0000 | 0.0000 |
| TCGA-A7-A0CD-01 | 0.0820 | 0.0000 | 0.0611 | 0.0169 | 0.0000 | 0.0671 | 0.0000 | 0.0191 | 0.0329 | 0.0000 | 0.0331 | 0.0000 | 0.0000 | 0.0000 | 0.0543 | 0.3091 | 0.0099 | 0.0000 | 0.3146 | 0.0000 | 0.0000 | 0.0000 |
| TCGA-A7-A0CE-01 | 0.1226 | 0.0000 | 0.0981 | 0.0000 | 0.0000 | 0.0167 | 0.0000 | 0.0568 | 0.0483 | 0.0000 | 0.0210 | 0.0030 | 0.0000 | 0.2744 | 0.1264 | 0.1955 | 0.0000 | 0.0000 | 0.0372 | 0.0000 | 0.0000 | 0.0000 |
| TCGA-A7-A0CG-01 | 0.0728 | 0.0000 | 0.0422 | 0.0514 | 0.0000 | 0.2832 | 0.0000 | 0.0718 | 0.0029 | 0.0000 | 0.0090 | 0.0000 | 0.0178 | 0.0341 | 0.0780 | 0.1989 | 0.0078 | 0.0000 | 0.1301 | 0.0000 | 0.0000 | 0.0000 |
| TCGA-A7-A0CH-01 | 0.0028 | 0.0000 | 0.0429 | 0.0024 | 0.0000 | 0.0717 | 0.0000 | 0.1287 | 0.0000 | 0.0000 | 0.0000 | 0.0444 | 0.0152 | 0.1612 | 0.0217 | 0.3177 | 0.0000 | 0.0342 | 0.1571 | 0.0000 | 0.0000 | 0.0000 |
| TCGA-A7-A0CJ-01 | 0.0884 | 0.0000 | 0.0000 | 0.0396 | 0.0000 | 0.1407 | 0.0131 | 0.0799 | 0.0284 | 0.0000 | 0.0477 | 0.0000 | 0.0000 | 0.3021 | 0.0691 | 0.1607 | 0.0061 | 0.0000 | 0.0218 | 0.0000 | 0.0000 | 0.0026 |
| TCGA-A7-A0D9-01 | 0.0024 | 0.0000 | 0.1009 | 0.0000 | 0.0000 | 0.3118 | 0.0000 | 0.0440 | 0.0640 | 0.0000 | 0.0546 | 0.0117 | 0.0000 | 0.3475 | 0.0000 | 0.0032 | 0.0000 | 0.0231 | 0.0368 | 0.0000 | 0.0000 | 0.0000 |
| TCGA-A7-A0DA-01 | 0.0647 | 0.0000 | 0.0280 | 0.0000 | 0.0000 | 0.0879 | 0.0000 | 0.0148 | 0.0122 | 0.0000 | 0.0361 | 0.0000 | 0.0000 | 0.4479 | 0.0688 | 0.2282 | 0.0000 | 0.0113 | 0.0000 | 0.0002 | 0.0000 | 0.0000 |
| TCGA-A7-A0DB-01 | 0.0000 | 0.0069 | 0.0000 | 0.0000 | 0.0000 | 0.1564 | 0.0000 | 0.0051 | 0.0248 | 0.0000 | 0.0310 | 0.0020 | 0.0157 | 0.4883 | 0.0093 | 0.1580 | 0.0000 | 0.0000 | 0.1025 | 0.0000 | 0.0000 | 0.0000 |
| TCGA-A7-A0DC-01 | 0.0017 | 0.0000 | 0.0843 | 0.0000 | 0.0000 | 0.1836 | 0.0000 | 0.1111 | 0.0075 | 0.0000 | 0.0348 | 0.0440 | 0.0000 | 0.3529 | 0.0023 | 0.0721 | 0.0000 | 0.0000 | 0.1058 | 0.0000 | 0.0000 | 0.0000 |
| TCGA-A7-A13D-01 | 0.0554 | 0.0000 | 0.0411 | 0.0024 | 0.0000 | 0.0000 | 0.0000 | 0.0762 | 0.0067 | 0.0093 | 0.0000 | 0.0000 | 0.0000 | 0.7935 | 0.0057 | 0.0006 | 0.0000 | 0.0089 | 0.0000 | 0.0000 | 0.0000 | 0.0000 |
| TCGA-A7-A13E-01 | 0.0017 | 0.0065 | 0.0062 | 0.0000 | 0.0000 | 0.1892 | 0.0000 | 0.0885 | 0.0470 | 0.0000 | 0.0535 | 0.0000 | 0.0000 | 0.4854 | 0.0000 | 0.0754 | 0.0000 | 0.0000 | 0.0466 | 0.0000 | 0.0000 | 0.0000 |
| TCGA-A7-A13F-01 | 0.0829 | 0.0000 | 0.0429 | 0.0099 | 0.0000 | 0.0438 | 0.0000 | 0.0552 | 0.0000 | 0.0000 | 0.0120 | 0.0000 | 0.0000 | 0.0333 | 0.0180 | 0.5197 | 0.0733 | 0.0211 | 0.0706 | 0.0000 | 0.0000 | 0.0173 |
| TCGA-A7-A13G-01 | 0.0349 | 0.0000 | 0.0224 | 0.0037 | 0.0000 | 0.0400 | 0.0000 | 0.1342 | 0.0111 | 0.0000 | 0.0000 | 0.0609 | 0.0166 | 0.0481 | 0.0115 | 0.3247 | 0.1940 | 0.0087 | 0.0893 | 0.0000 | 0.0000 | 0.0000 |
| TCGA-A7-A13H-01 | 0.0331 | 0.0000 | 0.0113 | 0.0379 | 0.0000 | 0.0806 | 0.0000 | 0.0286 | 0.0311 | 0.0000 | 0.0154 | 0.0000 | 0.0000 | 0.5603 | 0.0540 | 0.0934 | 0.0000 | 0.0000 | 0.0545 | 0.0000 | 0.0000 | 0.0000 |
| TCGA-A7-A26E-01 | 0.0869 | 0.0000 | 0.0052 | 0.0209 | 0.0000 | 0.1725 | 0.0000 | 0.0369 | 0.0000 | 0.0000 | 0.0199 | 0.0000 | 0.1325 | 0.0000 | 0.0714 | 0.3533 | 0.0000 | 0.0000 | 0.0962 | 0.0000 | 0.0000 | 0.0043 |
| TCGA-A7-A26F-01 | 0.0317 | 0.0000 | 0.0317 | 0.0130 | 0.0000 | 0.2185 | 0.0163 | 0.0217 | 0.0000 | 0.0000 | 0.0696 | 0.0000 | 0.0047 | 0.1313 | 0.0701 | 0.1728 | 0.0000 | 0.1719 | 0.0228 | 0.0000 | 0.0000 | 0.0238 |
| TCGA-A7-A26G-01 | 0.0649 | 0.0000 | 0.0679 | 0.0238 | 0.0000 | 0.1761 | 0.0000 | 0.0621 | 0.0021 | 0.0033 | 0.0000 | 0.0000 | 0.0000 | 0.1080 | 0.1101 | 0.2490 | 0.0000 | 0.0321 | 0.1004 | 0.0000 | 0.0000 | 0.0000 |
| TCGA-A7-A26H-01 | 0.0876 | 0.0000 | 0.0146 | 0.0162 | 0.0000 | 0.1625 | 0.0000 | 0.0583 | 0.0167 | 0.0000 | 0.0311 | 0.0000 | 0.0000 | 0.2336 | 0.0598 | 0.2872 | 0.0000 | 0.0000 | 0.0325 | 0.0000 | 0.0000 | 0.0000 |
| TCGA-A7-A26I-01 | 0.0000 | 0.0037 | 0.0188 | 0.0000 | 0.0000 | 0.3214 | 0.0000 | 0.0490 | 0.0547 | 0.0000 | 0.0285 | 0.0158 | 0.0136 | 0.3949 | 0.0007 | 0.0309 | 0.0000 | 0.0000 | 0.0680 | 0.0000 | 0.0000 | 0.0000 |
| TCGA-A7-A26J-01 | 0.0726 | 0.0000 | 0.0142 | 0.0000 | 0.0000 | 0.1919 | 0.0000 | 0.0530 | 0.0090 | 0.0000 | 0.0188 | 0.0000 | 0.0000 | 0.2133 | 0.0497 | 0.2935 | 0.0000 | 0.0000 | 0.0759 | 0.0000 | 0.0000 | 0.0080 |
| TCGA-A7-A2KD-01 | 0.0763 | 0.0000 | 0.0148 | 0.0277 | 0.0000 | 0.2247 | 0.0066 | 0.0583 | 0.0292 | 0.0000 | 0.0444 | 0.0000 | 0.0000 | 0.1011 | 0.0883 | 0.2982 | 0.0000 | 0.0000 | 0.0303 | 0.0000 | 0.0000 | 0.0000 |
| TCGA-A7-A3IY-01 | 0.0068 | 0.0000 | 0.0000 | 0.0000 | 0.0000 | 0.0070 | 0.0000 | 0.0676 | 0.0437 | 0.0000 | 0.0129 | 0.0046 | 0.0000 | 0.5397 | 0.0159 | 0.2475 | 0.0000 | 0.0000 | 0.0000 | 0.0532 | 0.0012 | 0.0000 |
| TCGA-A7-A3IZ-01 | 0.0983 | 0.0000 | 0.0203 | 0.0183 | 0.0000 | 0.0709 | 0.0000 | 0.0141 | 0.0015 | 0.0000 | 0.0000 | 0.0105 | 0.0000 | 0.1314 | 0.0188 | 0.3213 | 0.0000 | 0.0000 | 0.2945 | 0.0000 | 0.0000 | 0.0000 |
| TCGA-A7-A3J0-01 | 0.0858 | 0.0000 | 0.0000 | 0.0879 | 0.0000 | 0.0255 | 0.0000 | 0.1408 | 0.0717 | 0.0000 | 0.0364 | 0.0000 | 0.0156 | 0.2381 | 0.1266 | 0.1345 | 0.0000 | 0.0000 | 0.0370 | 0.0000 | 0.0000 | 0.0000 |
| TCGA-A7-A3J1-01 | 0.1304 | 0.0000 | 0.0123 | 0.0973 | 0.0000 | 0.2186 | 0.0077 | 0.0774 | 0.0282 | 0.0000 | 0.0507 | 0.0000 | 0.0040 | 0.0000 | 0.0686 | 0.2412 | 0.0000 | 0.0258 | 0.0377 | 0.0000 | 0.0000 | 0.0000 |
| TCGA-A7-A3RF-01 | 0.0518 | 0.0000 | 0.0328 | 0.0000 | 0.0000 | 0.0299 | 0.0000 | 0.0930 | 0.0328 | 0.0000 | 0.0229 | 0.0000 | 0.0000 | 0.3415 | 0.0181 | 0.2340 | 0.0000 | 0.0000 | 0.1430 | 0.0000 | 0.0000 | 0.0000 |
| TCGA-A7-A425-01 | 0.0530 | 0.0000 | 0.0000 | 0.0457 | 0.0000 | 0.1205 | 0.0000 | 0.0837 | 0.0000 | 0.0000 | 0.0435 | 0.0000 | 0.0831 | 0.0000 | 0.0176 | 0.4623 | 0.0000 | 0.0000 | 0.0907 | 0.0000 | 0.0000 | 0.0000 |
| TCGA-A7-A426-01 | 0.0982 | 0.0000 | 0.0217 | 0.1677 | 0.0000 | 0.0502 | 0.0000 | 0.1222 | 0.0000 | 0.0000 | 0.0000 | 0.0397 | 0.0149 | 0.0000 | 0.0234 | 0.1872 | 0.0000 | 0.0000 | 0.2748 | 0.0000 | 0.0000 | 0.0000 |
| TCGA-A7-A4SA-01 | 0.1137 | 0.0000 | 0.0045 | 0.1434 | 0.0000 | 0.2299 | 0.0101 | 0.0593 | 0.0572 | 0.0000 | 0.0238 | 0.0000 | 0.0091 | 0.0000 | 0.1245 | 0.1652 | 0.0097 | 0.0000 | 0.0496 | 0.0000 | 0.0000 | 0.0000 |
| TCGA-A7-A4SB-01 | 0.1200 | 0.0000 | 0.0597 | 0.0954 | 0.0000 | 0.0849 | 0.0000 | 0.1123 | 0.0000 | 0.0000 | 0.0010 | 0.0086 | 0.0016 | 0.0000 | 0.0540 | 0.3056 | 0.0000 | 0.0000 | 0.1570 | 0.0000 | 0.0000 | 0.0000 |
| TCGA-A7-A4SC-01 | 0.1064 | 0.0000 | 0.0090 | 0.1096 | 0.0000 | 0.1740 | 0.0000 | 0.0675 | 0.0228 | 0.0000 | 0.0303 | 0.0000 | 0.0185 | 0.0000 | 0.0630 | 0.3021 | 0.0000 | 0.0000 | 0.0967 | 0.0000 | 0.0000 | 0.0000 |
| TCGA-A7-A4SD-01 | 0.0547 | 0.0000 | 0.0920 | 0.0783 | 0.0000 | 0.0657 | 0.0051 | 0.1418 | 0.0395 | 0.0000 | 0.0315 | 0.0000 | 0.0039 | 0.2006 | 0.1669 | 0.0743 | 0.0000 | 0.0168 | 0.0287 | 0.0000 | 0.0000 | 0.0000 |
| TCGA-A7-A4SE-01 | 0.0112 | 0.0000 | 0.0000 | 0.0000 | 0.0000 | 0.1107 | 0.0000 | 0.1502 | 0.0075 | 0.0000 | 0.0081 | 0.0215 | 0.0000 | 0.4388 | 0.1076 | 0.1151 | 0.0000 | 0.0000 | 0.0293 | 0.0000 | 0.0000 | 0.0000 |
| TCGA-A7-A4SF-01 | 0.1060 | 0.0000 | 0.0165 | 0.0432 | 0.0000 | 0.0939 | 0.0000 | 0.1113 | 0.0635 | 0.0000 | 0.0201 | 0.0000 | 0.0000 | 0.2503 | 0.0941 | 0.1219 | 0.0000 | 0.0000 | 0.0793 | 0.0000 | 0.0000 | 0.0000 |
| TCGA-A7-A56D-01 | 0.0007 | 0.0000 | 0.0442 | 0.0265 | 0.0000 | 0.0000 | 0.0000 | 0.0579 | 0.0609 | 0.0114 | 0.0000 | 0.0228 | 0.0155 | 0.4203 | 0.0000 | 0.2456 | 0.0000 | 0.0460 | 0.0483 | 0.0000 | 0.0000 | 0.0000 |
| TCGA-A7-A5ZV-01 | 0.0416 | 0.0000 | 0.0188 | 0.0313 | 0.0000 | 0.0491 | 0.0000 | 0.0399 | 0.0100 | 0.0000 | 0.0000 | 0.0262 | 0.0000 | 0.3425 | 0.0073 | 0.3088 | 0.0010 | 0.0742 | 0.0438 | 0.0000 | 0.0000 | 0.0054 |
| TCGA-A7-A5ZW-01 | 0.0490 | 0.0000 | 0.0200 | 0.0251 | 0.0000 | 0.1548 | 0.0000 | 0.0454 | 0.0164 | 0.0000 | 0.0157 | 0.0000 | 0.0000 | 0.1744 | 0.0493 | 0.2365 | 0.0223 | 0.0000 | 0.1910 | 0.0000 | 0.0000 | 0.0000 |
| TCGA-A7-A5ZX-01 | 0.0000 | 0.0000 | 0.0593 | 0.0000 | 0.0000 | 0.2370 | 0.0000 | 0.0905 | 0.0052 | 0.0000 | 0.0334 | 0.0126 | 0.0275 | 0.1423 | 0.0000 | 0.0628 | 0.0071 | 0.0000 | 0.3222 | 0.0000 | 0.0000 | 0.0000 |
| TCGA-A7-A6VV-01 | 0.0034 | 0.0032 | 0.0092 | 0.0000 | 0.0000 | 0.0992 | 0.0000 | 0.0706 | 0.0349 | 0.0000 | 0.0275 | 0.0084 | 0.0000 | 0.5294 | 0.0489 | 0.1283 | 0.0000 | 0.0000 | 0.0371 | 0.0000 | 0.0000 | 0.0000 |
| TCGA-A7-A6VW-01 | 0.0504 | 0.0000 | 0.0408 | 0.0164 | 0.0000 | 0.0558 | 0.0000 | 0.0653 | 0.0342 | 0.0000 | 0.0000 | 0.0483 | 0.0189 | 0.2178 | 0.0000 | 0.2350 | 0.0000 | 0.1808 | 0.0144 | 0.0000 | 0.0000 | 0.0220 |
| TCGA-A7-A6VX-01 | 0.0003 | 0.0071 | 0.0000 | 0.0000 | 0.0000 | 0.2364 | 0.0000 | 0.0675 | 0.0729 | 0.0000 | 0.0533 | 0.0059 | 0.0137 | 0.4061 | 0.0112 | 0.0302 | 0.0000 | 0.0000 | 0.0955 | 0.0000 | 0.0000 | 0.0000 |
| TCGA-A7-A6VY-01 | 0.0000 | 0.0111 | 0.0834 | 0.0000 | 0.0000 | 0.0708 | 0.0159 | 0.1239 | 0.0179 | 0.0000 | 0.0537 | 0.0000 | 0.0000 | 0.3280 | 0.1495 | 0.1434 | 0.0000 | 0.0000 | 0.0022 | 0.0000 | 0.0000 | 0.0000 |
| TCGA-A8-A06N-01 | 0.0196 | 0.0000 | 0.1376 | 0.0000 | 0.0000 | 0.1513 | 0.0000 | 0.0738 | 0.0025 | 0.0000 | 0.0000 | 0.0387 | 0.0000 | 0.0343 | 0.0273 | 0.4240 | 0.0000 | 0.0000 | 0.0909 | 0.0000 | 0.0000 | 0.0000 |
| TCGA-A8-A06O-01 | 0.0898 | 0.0000 | 0.0252 | 0.0534 | 0.0000 | 0.1104 | 0.0000 | 0.0480 | 0.0337 | 0.0000 | 0.0000 | 0.0083 | 0.0029 | 0.1247 | 0.0603 | 0.3954 | 0.0113 | 0.0000 | 0.0366 | 0.0000 | 0.0000 | 0.0000 |
| TCGA-A8-A06P-01 | 0.0000 | 0.0283 | 0.0325 | 0.0000 | 0.0000 | 0.2164 | 0.0000 | 0.0314 | 0.0446 | 0.0000 | 0.0112 | 0.0000 | 0.0219 | 0.1940 | 0.0000 | 0.2864 | 0.0001 | 0.0000 | 0.1331 | 0.0000 | 0.0000 | 0.0000 |
| TCGA-A8-A06Q-01 | 0.1290 | 0.0000 | 0.0552 | 0.0022 | 0.0000 | 0.1013 | 0.0000 | 0.0584 | 0.0000 | 0.0000 | 0.0091 | 0.0009 | 0.0000 | 0.0000 | 0.0620 | 0.4491 | 0.0000 | 0.0000 | 0.1326 | 0.0000 | 0.0000 | 0.0000 |
| TCGA-A8-A06R-01 | 0.0384 | 0.0000 | 0.0668 | 0.0000 | 0.0000 | 0.1352 | 0.0000 | 0.0677 | 0.0555 | 0.0000 | 0.0284 | 0.0000 | 0.0083 | 0.3051 | 0.1106 | 0.1336 | 0.0017 | 0.0000 | 0.0488 | 0.0000 | 0.0000 | 0.0000 |
| TCGA-A8-A06T-01 | 0.1109 | 0.0000 | 0.0596 | 0.0688 | 0.0000 | 0.1545 | 0.0122 | 0.1395 | 0.0186 | 0.0032 | 0.0150 | 0.0000 | 0.0003 | 0.0617 | 0.0650 | 0.2514 | 0.0000 | 0.0244 | 0.0148 | 0.0000 | 0.0000 | 0.0000 |
| TCGA-A8-A06U-01 | 0.0700 | 0.0000 | 0.0427 | 0.0825 | 0.0000 | 0.0682 | 0.0000 | 0.0936 | 0.0368 | 0.0000 | 0.0126 | 0.0081 | 0.0116 | 0.0450 | 0.1311 | 0.3197 | 0.0000 | 0.0000 | 0.0781 | 0.0000 | 0.0000 | 0.0000 |
| TCGA-A8-A06X-01 | 0.0376 | 0.0000 | 0.0325 | 0.0533 | 0.0000 | 0.0665 | 0.0000 | 0.0912 | 0.0448 | 0.0000 | 0.0000 | 0.0377 | 0.0000 | 0.3179 | 0.0629 | 0.2343 | 0.0000 | 0.0000 | 0.0214 | 0.0000 | 0.0000 | 0.0000 |
| TCGA-A8-A06Y-01 | 0.1685 | 0.0000 | 0.0784 | 0.0000 | 0.0000 | 0.0000 | 0.0000 | 0.1019 | 0.0235 | 0.0000 | 0.0050 | 0.0126 | 0.0000 | 0.2172 | 0.0617 | 0.1955 | 0.0000 | 0.0737 | 0.0620 | 0.0000 | 0.0000 | 0.0000 |
| TCGA-A8-A06Z-01 | 0.0020 | 0.0044 | 0.0000 | 0.0000 | 0.0000 | 0.3786 | 0.0000 | 0.0065 | 0.0919 | 0.0000 | 0.0777 | 0.0000 | 0.0000 | 0.2155 | 0.0151 | 0.1625 | 0.0129 | 0.0000 | 0.0329 | 0.0000 | 0.0000 | 0.0000 |
| TCGA-A8-A075-01 | 0.0449 | 0.0000 | 0.1058 | 0.0626 | 0.0000 | 0.1205 | 0.0000 | 0.0943 | 0.0165 | 0.0000 | 0.0455 | 0.0000 | 0.0030 | 0.1657 | 0.0830 | 0.2064 | 0.0086 | 0.0000 | 0.0433 | 0.0000 | 0.0000 | 0.0000 |
| TCGA-A8-A076-01 | 0.0508 | 0.0000 | 0.0383 | 0.0000 | 0.0000 | 0.1004 | 0.0000 | 0.0489 | 0.0299 | 0.0000 | 0.0273 | 0.0000 | 0.0000 | 0.2265 | 0.0761 | 0.2988 | 0.0000 | 0.0000 | 0.0999 | 0.0000 | 0.0000 | 0.0032 |
| TCGA-A8-A079-01 | 0.0746 | 0.0000 | 0.1000 | 0.0395 | 0.0000 | 0.0610 | 0.0000 | 0.0222 | 0.0364 | 0.0000 | 0.0307 | 0.0000 | 0.0000 | 0.2126 | 0.0395 | 0.3592 | 0.0000 | 0.0000 | 0.0202 | 0.0000 | 0.0000 | 0.0040 |
| TCGA-A8-A07B-01 | 0.0000 | 0.0026 | 0.0003 | 0.0000 | 0.0000 | 0.2556 | 0.0000 | 0.0781 | 0.0151 | 0.0000 | 0.0294 | 0.0230 | 0.0431 | 0.0892 | 0.0177 | 0.1710 | 0.0000 | 0.0408 | 0.2344 | 0.0000 | 0.0000 | 0.0000 |
| TCGA-A8-A07C-01 | 0.0059 | 0.0000 | 0.0552 | 0.0161 | 0.0000 | 0.1482 | 0.0000 | 0.1229 | 0.0046 | 0.0000 | 0.0139 | 0.0337 | 0.0260 | 0.0548 | 0.0909 | 0.2837 | 0.0000 | 0.0108 | 0.1333 | 0.0000 | 0.0000 | 0.0000 |
| TCGA-A8-A07E-01 | 0.0589 | 0.0000 | 0.0205 | 0.0934 | 0.0000 | 0.1621 | 0.0000 | 0.0529 | 0.0194 | 0.0000 | 0.0192 | 0.0000 | 0.0119 | 0.0000 | 0.1131 | 0.2558 | 0.0106 | 0.0000 | 0.1821 | 0.0000 | 0.0000 | 0.0000 |
| TCGA-A8-A07F-01 | 0.0724 | 0.0000 | 0.0000 | 0.0351 | 0.0000 | 0.2476 | 0.0000 | 0.0286 | 0.0127 | 0.0000 | 0.0063 | 0.0000 | 0.0000 | 0.0817 | 0.0802 | 0.3071 | 0.0211 | 0.0000 | 0.1073 | 0.0000 | 0.0000 | 0.0000 |
| TCGA-A8-A07G-01 | 0.1218 | 0.0000 | 0.0428 | 0.0629 | 0.0000 | 0.1050 | 0.0000 | 0.0873 | 0.0138 | 0.0000 | 0.0234 | 0.0000 | 0.0000 | 0.1496 | 0.0524 | 0.2901 | 0.0011 | 0.0012 | 0.0487 | 0.0000 | 0.0000 | 0.0000 |
| TCGA-A8-A07I-01 | 0.0062 | 0.0000 | 0.0096 | 0.0000 | 0.0000 | 0.0726 | 0.0000 | 0.0550 | 0.0553 | 0.0000 | 0.0164 | 0.0000 | 0.0000 | 0.3766 | 0.0929 | 0.2458 | 0.0000 | 0.0000 | 0.0698 | 0.0000 | 0.0000 | 0.0000 |
| TCGA-A8-A07J-01 | 0.0544 | 0.0000 | 0.0097 | 0.0176 | 0.0000 | 0.0938 | 0.0000 | 0.0074 | 0.0158 | 0.0000 | 0.0155 | 0.0000 | 0.0024 | 0.2346 | 0.0290 | 0.3902 | 0.0438 | 0.0000 | 0.0832 | 0.0000 | 0.0000 | 0.0024 |
| TCGA-A8-A07L-01 | 0.0904 | 0.0000 | 0.0406 | 0.0356 | 0.0000 | 0.0739 | 0.0000 | 0.0836 | 0.0227 | 0.0000 | 0.0030 | 0.0038 | 0.0000 | 0.1714 | 0.0485 | 0.3632 | 0.0000 | 0.0000 | 0.0619 | 0.0000 | 0.0000 | 0.0015 |
| TCGA-A8-A07O-01 | 0.0086 | 0.0000 | 0.0000 | 0.0295 | 0.0000 | 0.0985 | 0.0037 | 0.0445 | 0.0217 | 0.0000 | 0.0075 | 0.0027 | 0.0000 | 0.6995 | 0.0179 | 0.0425 | 0.0000 | 0.0233 | 0.0000 | 0.0000 | 0.0000 | 0.0000 |
| TCGA-A8-A07P-01 | 0.0747 | 0.0000 | 0.0204 | 0.0386 | 0.0000 | 0.1070 | 0.0051 | 0.0072 | 0.0000 | 0.0000 | 0.0000 | 0.0065 | 0.0382 | 0.0091 | 0.0400 | 0.3445 | 0.0000 | 0.0103 | 0.2985 | 0.0000 | 0.0000 | 0.0000 |
| TCGA-A8-A07R-01 | 0.1479 | 0.0000 | 0.2232 | 0.0029 | 0.0000 | 0.0511 | 0.0000 | 0.0312 | 0.0256 | 0.0000 | 0.0000 | 0.0158 | 0.0000 | 0.1958 | 0.0794 | 0.1230 | 0.0088 | 0.0452 | 0.0312 | 0.0000 | 0.0000 | 0.0189 |
| TCGA-A8-A07S-01 | 0.0067 | 0.0000 | 0.0000 | 0.0000 | 0.0216 | 0.2374 | 0.0000 | 0.0775 | 0.0655 | 0.0000 | 0.0533 | 0.0000 | 0.0006 | 0.2083 | 0.0138 | 0.2341 | 0.0000 | 0.0007 | 0.0806 | 0.0000 | 0.0000 | 0.0000 |
| TCGA-A8-A07U-01 | 0.0501 | 0.0000 | 0.0484 | 0.0624 | 0.0000 | 0.1076 | 0.0318 | 0.1238 | 0.0563 | 0.0000 | 0.0250 | 0.0072 | 0.0157 | 0.0972 | 0.2285 | 0.0848 | 0.0000 | 0.0127 | 0.0484 | 0.0000 | 0.0000 | 0.0000 |
| TCGA-A8-A07W-01 | 0.0166 | 0.0000 | 0.0263 | 0.0000 | 0.0000 | 0.1102 | 0.0000 | 0.0379 | 0.0303 | 0.0000 | 0.0203 | 0.0000 | 0.0000 | 0.3784 | 0.0751 | 0.2590 | 0.0000 | 0.0000 | 0.0442 | 0.0000 | 0.0000 | 0.0019 |
| TCGA-A8-A07Z-01 | 0.0231 | 0.0000 | 0.0000 | 0.0667 | 0.0000 | 0.0075 | 0.0000 | 0.1190 | 0.0794 | 0.0000 | 0.0000 | 0.0718 | 0.0586 | 0.0000 | 0.0000 | 0.3481 | 0.0000 | 0.1481 | 0.0776 | 0.0000 | 0.0000 | 0.0000 |
| TCGA-A8-A081-01 | 0.0297 | 0.0000 | 0.0130 | 0.1821 | 0.0000 | 0.0000 | 0.0014 | 0.0519 | 0.0476 | 0.0000 | 0.0110 | 0.0119 | 0.0191 | 0.2479 | 0.1340 | 0.1299 | 0.0000 | 0.0000 | 0.1207 | 0.0000 | 0.0000 | 0.0000 |
| TCGA-A8-A082-01 | 0.0000 | 0.0000 | 0.0203 | 0.0000 | 0.0000 | 0.1432 | 0.0000 | 0.0039 | 0.0391 | 0.0000 | 0.1027 | 0.0000 | 0.0133 | 0.0921 | 0.0000 | 0.5021 | 0.0192 | 0.0000 | 0.0640 | 0.0000 | 0.0000 | 0.0000 |
| TCGA-A8-A083-01 | 0.0065 | 0.0000 | 0.0296 | 0.0000 | 0.0000 | 0.0950 | 0.0000 | 0.2233 | 0.0000 | 0.0000 | 0.0233 | 0.0698 | 0.0390 | 0.0685 | 0.0384 | 0.3178 | 0.0000 | 0.0035 | 0.0852 | 0.0000 | 0.0000 | 0.0000 |
| TCGA-A8-A084-01 | 0.1058 | 0.0000 | 0.0000 | 0.0000 | 0.0000 | 0.0533 | 0.0000 | 0.0315 | 0.0132 | 0.0000 | 0.0000 | 0.0090 | 0.0000 | 0.4808 | 0.0896 | 0.1517 | 0.0000 | 0.0000 | 0.0406 | 0.0000 | 0.0000 | 0.0246 |
| TCGA-A8-A085-01 | 0.1293 | 0.0000 | 0.0325 | 0.0000 | 0.0000 | 0.0000 | 0.0000 | 0.0233 | 0.0505 | 0.0000 | 0.0283 | 0.0000 | 0.0000 | 0.3313 | 0.0034 | 0.3227 | 0.0000 | 0.0000 | 0.0786 | 0.0000 | 0.0000 | 0.0000 |
| TCGA-A8-A086-01 | 0.0013 | 0.0030 | 0.0038 | 0.0000 | 0.0000 | 0.2102 | 0.0000 | 0.0284 | 0.0573 | 0.0000 | 0.0583 | 0.0014 | 0.0027 | 0.3491 | 0.0007 | 0.1996 | 0.0000 | 0.0035 | 0.0808 | 0.0000 | 0.0000 | 0.0000 |
| TCGA-A8-A08A-01 | 0.0562 | 0.0000 | 0.0029 | 0.0307 | 0.0000 | 0.0723 | 0.0000 | 0.0911 | 0.0331 | 0.0000 | 0.0100 | 0.0103 | 0.0000 | 0.2721 | 0.0503 | 0.3510 | 0.0000 | 0.0000 | 0.0200 | 0.0000 | 0.0000 | 0.0000 |
| TCGA-A8-A08B-01 | 0.0460 | 0.0000 | 0.0569 | 0.0312 | 0.0000 | 0.2294 | 0.0000 | 0.1126 | 0.0252 | 0.0000 | 0.0758 | 0.0000 | 0.0044 | 0.1024 | 0.1116 | 0.1202 | 0.0000 | 0.0193 | 0.0650 | 0.0000 | 0.0000 | 0.0000 |
| TCGA-A8-A08C-01 | 0.1104 | 0.0000 | 0.0110 | 0.0453 | 0.0000 | 0.1336 | 0.0000 | 0.0645 | 0.0529 | 0.0000 | 0.0121 | 0.0015 | 0.0000 | 0.0000 | 0.0535 | 0.3854 | 0.0075 | 0.0781 | 0.0404 | 0.0000 | 0.0000 | 0.0038 |
| TCGA-A8-A08F-01 | 0.0058 | 0.0000 | 0.0000 | 0.0000 | 0.0000 | 0.1489 | 0.0000 | 0.0064 | 0.0048 | 0.0000 | 0.0000 | 0.0267 | 0.0151 | 0.2413 | 0.0975 | 0.3521 | 0.0000 | 0.0000 | 0.1014 | 0.0000 | 0.0000 | 0.0000 |
| TCGA-A8-A08G-01 | 0.0545 | 0.0000 | 0.0062 | 0.0000 | 0.0000 | 0.1300 | 0.0000 | 0.0867 | 0.0465 | 0.0000 | 0.0414 | 0.0000 | 0.0000 | 0.1751 | 0.0961 | 0.2980 | 0.0000 | 0.0000 | 0.0656 | 0.0000 | 0.0000 | 0.0000 |
| TCGA-A8-A08H-01 | 0.0076 | 0.0000 | 0.0000 | 0.0115 | 0.0000 | 0.1268 | 0.0000 | 0.0040 | 0.0051 | 0.0000 | 0.0000 | 0.0092 | 0.0659 | 0.0886 | 0.0227 | 0.5390 | 0.0002 | 0.0000 | 0.1065 | 0.0000 | 0.0000 | 0.0129 |
| TCGA-A8-A08I-01 | 0.0052 | 0.0000 | 0.0000 | 0.1174 | 0.0000 | 0.0000 | 0.0042 | 0.0327 | 0.0598 | 0.0000 | 0.0000 | 0.0145 | 0.0000 | 0.4942 | 0.1141 | 0.1051 | 0.0000 | 0.0000 | 0.0529 | 0.0000 | 0.0000 | 0.0000 |
| TCGA-A8-A08J-01 | 0.0646 | 0.0000 | 0.1454 | 0.0161 | 0.0000 | 0.0269 | 0.0000 | 0.0203 | 0.0209 | 0.0000 | 0.0083 | 0.0000 | 0.0000 | 0.2733 | 0.0562 | 0.3258 | 0.0000 | 0.0000 | 0.0425 | 0.0000 | 0.0000 | 0.0000 |
| TCGA-A8-A08L-01 | 0.0650 | 0.0000 | 0.0118 | 0.0783 | 0.0000 | 0.2093 | 0.0073 | 0.0812 | 0.0635 | 0.0000 | 0.0405 | 0.0000 | 0.0321 | 0.1069 | 0.1021 | 0.1902 | 0.0000 | 0.0000 | 0.0119 | 0.0000 | 0.0000 | 0.0000 |
| TCGA-A8-A08O-01 | 0.0571 | 0.0000 | 0.0648 | 0.0010 | 0.0000 | 0.1997 | 0.0000 | 0.0645 | 0.0414 | 0.0000 | 0.0000 | 0.0586 | 0.0185 | 0.0454 | 0.0369 | 0.2770 | 0.0000 | 0.0000 | 0.1351 | 0.0000 | 0.0000 | 0.0000 |
| TCGA-A8-A08P-01 | 0.0445 | 0.0000 | 0.0000 | 0.0000 | 0.0000 | 0.2386 | 0.0024 | 0.0650 | 0.0379 | 0.0000 | 0.0094 | 0.0057 | 0.0000 | 0.1592 | 0.1560 | 0.1771 | 0.0051 | 0.0000 | 0.0954 | 0.0000 | 0.0000 | 0.0036 |
| TCGA-A8-A08R-01 | 0.0392 | 0.0000 | 0.0325 | 0.0108 | 0.0000 | 0.0879 | 0.0000 | 0.0724 | 0.0515 | 0.0000 | 0.0185 | 0.0064 | 0.0000 | 0.4061 | 0.1376 | 0.1030 | 0.0000 | 0.0000 | 0.0341 | 0.0000 | 0.0000 | 0.0000 |
| TCGA-A8-A08S-01 | 0.0036 | 0.0000 | 0.0004 | 0.0000 | 0.0000 | 0.0909 | 0.0000 | 0.1107 | 0.0761 | 0.0000 | 0.0399 | 0.0000 | 0.0000 | 0.4679 | 0.0148 | 0.1106 | 0.0000 | 0.0022 | 0.0830 | 0.0000 | 0.0000 | 0.0000 |
| TCGA-A8-A08T-01 | 0.0489 | 0.0000 | 0.0018 | 0.0385 | 0.0000 | 0.1036 | 0.0000 | 0.0376 | 0.0191 | 0.0000 | 0.0167 | 0.0000 | 0.0000 | 0.3275 | 0.0209 | 0.2872 | 0.0000 | 0.0000 | 0.0982 | 0.0000 | 0.0000 | 0.0000 |
| TCGA-A8-A08X-01 | 0.0379 | 0.0000 | 0.2829 | 0.0240 | 0.0000 | 0.1354 | 0.0000 | 0.0463 | 0.0437 | 0.0000 | 0.0396 | 0.0000 | 0.0081 | 0.1315 | 0.0497 | 0.1670 | 0.0000 | 0.0058 | 0.0282 | 0.0000 | 0.0000 | 0.0000 |
| TCGA-A8-A08Z-01 | 0.0351 | 0.0000 | 0.0000 | 0.0159 | 0.0000 | 0.1420 | 0.0000 | 0.0152 | 0.0161 | 0.0000 | 0.0205 | 0.0000 | 0.0112 | 0.2440 | 0.0382 | 0.3559 | 0.0000 | 0.0000 | 0.1057 | 0.0000 | 0.0000 | 0.0000 |
| TCGA-A8-A090-01 | 0.0000 | 0.0000 | 0.0055 | 0.0000 | 0.0000 | 0.0569 | 0.0000 | 0.0544 | 0.0682 | 0.0025 | 0.0035 | 0.0000 | 0.0000 | 0.4242 | 0.0617 | 0.2549 | 0.0000 | 0.0000 | 0.0681 | 0.0000 | 0.0000 | 0.0000 |
| TCGA-A8-A091-01 | 0.0618 | 0.0000 | 0.0257 | 0.0659 | 0.0000 | 0.1771 | 0.0000 | 0.0264 | 0.0066 | 0.0000 | 0.0286 | 0.0000 | 0.0000 | 0.0571 | 0.0345 | 0.4359 | 0.0000 | 0.0000 | 0.0803 | 0.0000 | 0.0000 | 0.0000 |
| TCGA-A8-A092-01 | 0.0017 | 0.0000 | 0.0045 | 0.0000 | 0.0000 | 0.2500 | 0.0000 | 0.0713 | 0.0572 | 0.0000 | 0.0300 | 0.0246 | 0.0359 | 0.0642 | 0.0231 | 0.2722 | 0.0000 | 0.0078 | 0.1519 | 0.0000 | 0.0055 | 0.0000 |
| TCGA-A8-A093-01 | 0.0000 | 0.0038 | 0.0347 | 0.0000 | 0.0000 | 0.2611 | 0.0000 | 0.0209 | 0.0584 | 0.0000 | 0.1034 | 0.0000 | 0.0204 | 0.2094 | 0.0059 | 0.0542 | 0.0277 | 0.0000 | 0.1997 | 0.0000 | 0.0000 | 0.0004 |
| TCGA-A8-A094-01 | 0.0106 | 0.0000 | 0.0170 | 0.0674 | 0.0000 | 0.1064 | 0.0000 | 0.1027 | 0.0094 | 0.0000 | 0.0000 | 0.0441 | 0.0000 | 0.1220 | 0.1645 | 0.2450 | 0.0013 | 0.0000 | 0.1096 | 0.0000 | 0.0000 | 0.0000 |
| TCGA-A8-A095-01 | 0.0945 | 0.0000 | 0.0000 | 0.0452 | 0.0000 | 0.1240 | 0.0000 | 0.0987 | 0.0202 | 0.0000 | 0.0050 | 0.0000 | 0.0000 | 0.0976 | 0.0449 | 0.3374 | 0.0082 | 0.0093 | 0.1148 | 0.0000 | 0.0000 | 0.0000 |
| TCGA-A8-A096-01 | 0.0033 | 0.0000 | 0.0047 | 0.0533 | 0.0000 | 0.2418 | 0.0014 | 0.0670 | 0.0388 | 0.0000 | 0.0025 | 0.0181 | 0.0182 | 0.0650 | 0.0730 | 0.2676 | 0.0243 | 0.0000 | 0.1211 | 0.0000 | 0.0000 | 0.0000 |
| TCGA-A8-A097-01 | 0.0850 | 0.0000 | 0.0032 | 0.0000 | 0.0000 | 0.1407 | 0.0000 | 0.0358 | 0.0251 | 0.0000 | 0.0092 | 0.0002 | 0.0000 | 0.3257 | 0.0772 | 0.2441 | 0.0000 | 0.0000 | 0.0538 | 0.0000 | 0.0000 | 0.0000 |
| TCGA-A8-A099-01 | 0.0538 | 0.0000 | 0.0488 | 0.0000 | 0.0000 | 0.1954 | 0.0000 | 0.0385 | 0.0453 | 0.0000 | 0.0117 | 0.0044 | 0.0073 | 0.0038 | 0.0527 | 0.4466 | 0.0109 | 0.0000 | 0.0808 | 0.0000 | 0.0000 | 0.0000 |
| TCGA-A8-A09A-01 | 0.1136 | 0.0000 | 0.0475 | 0.0641 | 0.0000 | 0.2007 | 0.0014 | 0.0481 | 0.0392 | 0.0000 | 0.0182 | 0.0000 | 0.0065 | 0.0526 | 0.1033 | 0.2231 | 0.0000 | 0.0000 | 0.0816 | 0.0000 | 0.0000 | 0.0000 |
| TCGA-A8-A09B-01 | 0.1334 | 0.0000 | 0.0000 | 0.0750 | 0.0000 | 0.1514 | 0.0000 | 0.0375 | 0.0249 | 0.0000 | 0.0390 | 0.0000 | 0.0502 | 0.0000 | 0.0627 | 0.3494 | 0.0000 | 0.0000 | 0.0718 | 0.0000 | 0.0000 | 0.0048 |
| TCGA-A8-A09C-01 | 0.1090 | 0.0000 | 0.0351 | 0.0000 | 0.0000 | 0.0985 | 0.0000 | 0.0627 | 0.0100 | 0.0000 | 0.0000 | 0.0184 | 0.0003 | 0.1248 | 0.0649 | 0.3683 | 0.0000 | 0.0092 | 0.0986 | 0.0000 | 0.0000 | 0.0000 |
| TCGA-A8-A09D-01 | 0.0535 | 0.0000 | 0.0070 | 0.0094 | 0.0000 | 0.1221 | 0.0000 | 0.0158 | 0.0261 | 0.0000 | 0.0251 | 0.0000 | 0.0000 | 0.2774 | 0.0482 | 0.3738 | 0.0000 | 0.0000 | 0.0386 | 0.0000 | 0.0000 | 0.0031 |
| TCGA-A8-A09E-01 | 0.1272 | 0.0000 | 0.0039 | 0.0080 | 0.0000 | 0.1191 | 0.0000 | 0.0912 | 0.0123 | 0.0000 | 0.0024 | 0.0000 | 0.0000 | 0.0787 | 0.1023 | 0.4010 | 0.0000 | 0.0099 | 0.0440 | 0.0000 | 0.0000 | 0.0000 |
| TCGA-A8-A09G-01 | 0.0753 | 0.0000 | 0.0248 | 0.0864 | 0.0000 | 0.2384 | 0.0000 | 0.1205 | 0.0346 | 0.0000 | 0.0186 | 0.0152 | 0.0267 | 0.0000 | 0.0956 | 0.2006 | 0.0026 | 0.0000 | 0.0606 | 0.0000 | 0.0000 | 0.0000 |
| TCGA-A8-A09I-01 | 0.0746 | 0.0000 | 0.0368 | 0.0387 | 0.0000 | 0.1026 | 0.0408 | 0.0828 | 0.0173 | 0.0000 | 0.0351 | 0.0000 | 0.0595 | 0.0685 | 0.0770 | 0.2537 | 0.0171 | 0.0489 | 0.0270 | 0.0000 | 0.0000 | 0.0195 |
| TCGA-A8-A09K-01 | 0.0517 | 0.0000 | 0.0546 | 0.1813 | 0.0000 | 0.0802 | 0.0259 | 0.1086 | 0.0448 | 0.0099 | 0.0000 | 0.0032 | 0.0000 | 0.0715 | 0.0515 | 0.1575 | 0.0000 | 0.0000 | 0.1592 | 0.0000 | 0.0000 | 0.0000 |
| TCGA-A8-A09M-01 | 0.1204 | 0.0000 | 0.0596 | 0.0654 | 0.0000 | 0.1358 | 0.0000 | 0.0976 | 0.0580 | 0.0000 | 0.0279 | 0.0000 | 0.0055 | 0.0242 | 0.1010 | 0.2666 | 0.0034 | 0.0000 | 0.0346 | 0.0000 | 0.0000 | 0.0000 |
| TCGA-A8-A09N-01 | 0.0917 | 0.0000 | 0.0228 | 0.1297 | 0.0000 | 0.1583 | 0.0000 | 0.0610 | 0.0464 | 0.0000 | 0.0073 | 0.0046 | 0.0082 | 0.0000 | 0.0911 | 0.2868 | 0.0000 | 0.0000 | 0.0920 | 0.0000 | 0.0000 | 0.0000 |
| TCGA-A8-A09Q-01 | 0.0376 | 0.0048 | 0.0000 | 0.0753 | 0.0000 | 0.1366 | 0.0028 | 0.0868 | 0.0073 | 0.0000 | 0.0311 | 0.0000 | 0.0214 | 0.1359 | 0.0510 | 0.2919 | 0.0000 | 0.0000 | 0.1176 | 0.0000 | 0.0000 | 0.0000 |
| TCGA-A8-A09R-01 | 0.0014 | 0.0000 | 0.0048 | 0.0000 | 0.0000 | 0.1148 | 0.0000 | 0.0747 | 0.0461 | 0.0000 | 0.0000 | 0.0339 | 0.0062 | 0.3228 | 0.1319 | 0.2011 | 0.0044 | 0.0000 | 0.0581 | 0.0000 | 0.0000 | 0.0000 |
| TCGA-A8-A09T-01 | 0.0052 | 0.0000 | 0.0000 | 0.0849 | 0.0000 | 0.2036 | 0.0000 | 0.0406 | 0.0107 | 0.0000 | 0.0000 | 0.0239 | 0.0335 | 0.0557 | 0.0080 | 0.4237 | 0.0000 | 0.0000 | 0.1095 | 0.0000 | 0.0006 | 0.0000 |
| TCGA-A8-A09V-01 | 0.0685 | 0.0000 | 0.0583 | 0.0000 | 0.0187 | 0.1001 | 0.0000 | 0.0371 | 0.0024 | 0.0000 | 0.0650 | 0.0000 | 0.0584 | 0.0000 | 0.0093 | 0.3744 | 0.0000 | 0.0000 | 0.2055 | 0.0000 | 0.0000 | 0.0023 |
| TCGA-A8-A09W-01 | 0.0906 | 0.0000 | 0.0447 | 0.0164 | 0.0000 | 0.1810 | 0.0000 | 0.0308 | 0.0218 | 0.0000 | 0.0331 | 0.0000 | 0.0000 | 0.0000 | 0.0632 | 0.3909 | 0.0000 | 0.0000 | 0.1272 | 0.0000 | 0.0000 | 0.0003 |
| TCGA-A8-A09X-01 | 0.0444 | 0.0000 | 0.0297 | 0.1424 | 0.0000 | 0.1387 | 0.0000 | 0.0770 | 0.0753 | 0.0000 | 0.0104 | 0.0064 | 0.0136 | 0.0406 | 0.0520 | 0.2838 | 0.0000 | 0.0162 | 0.0690 | 0.0000 | 0.0000 | 0.0005 |
| TCGA-A8-A09Z-01 | 0.0041 | 0.0000 | 0.0078 | 0.0598 | 0.0000 | 0.2597 | 0.0038 | 0.0934 | 0.0623 | 0.0000 | 0.0693 | 0.0081 | 0.0119 | 0.0537 | 0.1019 | 0.1519 | 0.0011 | 0.0000 | 0.1063 | 0.0000 | 0.0049 | 0.0000 |
| TCGA-A8-A0A1-01 | 0.0423 | 0.0000 | 0.0000 | 0.0181 | 0.0000 | 0.1169 | 0.0008 | 0.0603 | 0.0398 | 0.0000 | 0.0130 | 0.0000 | 0.0000 | 0.4748 | 0.0696 | 0.1220 | 0.0000 | 0.0000 | 0.0425 | 0.0000 | 0.0000 | 0.0000 |
| TCGA-A8-A0A2-01 | 0.0825 | 0.0000 | 0.1107 | 0.0740 | 0.0000 | 0.2101 | 0.0000 | 0.0421 | 0.0261 | 0.0000 | 0.0205 | 0.0000 | 0.0503 | 0.0000 | 0.0495 | 0.2633 | 0.0000 | 0.0000 | 0.0710 | 0.0000 | 0.0000 | 0.0000 |
| TCGA-A8-A0A4-01 | 0.1510 | 0.0000 | 0.0438 | 0.0701 | 0.0000 | 0.1001 | 0.0000 | 0.0694 | 0.0380 | 0.0000 | 0.0258 | 0.0037 | 0.0039 | 0.0365 | 0.0779 | 0.2959 | 0.0000 | 0.0000 | 0.0840 | 0.0000 | 0.0000 | 0.0000 |
| TCGA-A8-A0A6-01 | 0.0207 | 0.0214 | 0.0594 | 0.1031 | 0.0000 | 0.1757 | 0.0000 | 0.1167 | 0.0379 | 0.0016 | 0.0108 | 0.0000 | 0.0000 | 0.0394 | 0.1108 | 0.2239 | 0.0046 | 0.0136 | 0.0604 | 0.0000 | 0.0000 | 0.0000 |
| TCGA-A8-A0A7-01 | 0.0885 | 0.0000 | 0.0363 | 0.1494 | 0.0000 | 0.1997 | 0.0110 | 0.0406 | 0.0563 | 0.0000 | 0.0185 | 0.0000 | 0.0051 | 0.1208 | 0.1045 | 0.1307 | 0.0000 | 0.0000 | 0.0385 | 0.0000 | 0.0000 | 0.0000 |
| TCGA-A8-A0A9-01 | 0.0486 | 0.0000 | 0.0000 | 0.0349 | 0.0000 | 0.1775 | 0.0000 | 0.0600 | 0.0209 | 0.0000 | 0.0251 | 0.0000 | 0.0000 | 0.2679 | 0.0990 | 0.2456 | 0.0000 | 0.0000 | 0.0206 | 0.0000 | 0.0000 | 0.0000 |
| TCGA-A8-A0AB-01 | 0.1239 | 0.0000 | 0.0825 | 0.0000 | 0.0000 | 0.0377 | 0.0000 | 0.0531 | 0.0009 | 0.0000 | 0.0000 | 0.0153 | 0.0000 | 0.1031 | 0.0000 | 0.3746 | 0.0045 | 0.0272 | 0.1772 | 0.0000 | 0.0000 | 0.0000 |
| TCGA-A8-A0AD-01 | 0.0000 | 0.0055 | 0.0026 | 0.0000 | 0.0000 | 0.2415 | 0.0000 | 0.0178 | 0.0038 | 0.0000 | 0.0539 | 0.0219 | 0.0391 | 0.0319 | 0.0000 | 0.3988 | 0.0000 | 0.0000 | 0.1833 | 0.0000 | 0.0000 | 0.0000 |
| TCGA-AC-A23C-01 | 0.0931 | 0.0000 | 0.0842 | 0.0356 | 0.0000 | 0.2249 | 0.0016 | 0.0561 | 0.0199 | 0.0000 | 0.0243 | 0.0000 | 0.0000 | 0.0082 | 0.0810 | 0.3043 | 0.0000 | 0.0246 | 0.0357 | 0.0000 | 0.0000 | 0.0064 |
| TCGA-AC-A23E-01 | 0.0149 | 0.0000 | 0.0001 | 0.0000 | 0.0000 | 0.0575 | 0.0000 | 0.0589 | 0.0016 | 0.0000 | 0.0098 | 0.0021 | 0.0000 | 0.1838 | 0.0109 | 0.5398 | 0.0000 | 0.0000 | 0.1112 | 0.0000 | 0.0000 | 0.0094 |
| TCGA-AC-A23G-01 | 0.0367 | 0.0000 | 0.0000 | 0.0370 | 0.0000 | 0.0881 | 0.0000 | 0.0050 | 0.0094 | 0.0000 | 0.0061 | 0.0000 | 0.0000 | 0.5923 | 0.0194 | 0.1663 | 0.0000 | 0.0000 | 0.0396 | 0.0000 | 0.0000 | 0.0000 |
| TCGA-AC-A23H-01 | 0.0011 | 0.0042 | 0.0018 | 0.0000 | 0.0000 | 0.2757 | 0.0000 | 0.0390 | 0.0845 | 0.0000 | 0.0537 | 0.0197 | 0.0000 | 0.3788 | 0.0031 | 0.0695 | 0.0000 | 0.0000 | 0.0689 | 0.0000 | 0.0000 | 0.0000 |
| TCGA-AC-A2B8-01 | 0.0835 | 0.0000 | 0.0000 | 0.0685 | 0.0000 | 0.2306 | 0.0007 | 0.0303 | 0.0075 | 0.0000 | 0.0092 | 0.0000 | 0.0452 | 0.0501 | 0.0660 | 0.3244 | 0.0000 | 0.0040 | 0.0769 | 0.0000 | 0.0000 | 0.0031 |
| TCGA-AC-A2BK-01 | 0.0220 | 0.0000 | 0.0748 | 0.2601 | 0.0000 | 0.1067 | 0.0000 | 0.1971 | 0.0081 | 0.0000 | 0.0000 | 0.0355 | 0.0494 | 0.1034 | 0.0367 | 0.0000 | 0.0000 | 0.0000 | 0.1041 | 0.0000 | 0.0021 | 0.0000 |
| TCGA-AC-A2BM-01 | 0.0058 | 0.0000 | 0.0467 | 0.0080 | 0.0000 | 0.2281 | 0.0000 | 0.0971 | 0.0037 | 0.0000 | 0.0418 | 0.0000 | 0.0000 | 0.1193 | 0.0534 | 0.2576 | 0.0000 | 0.0230 | 0.1155 | 0.0000 | 0.0000 | 0.0000 |
| TCGA-AC-A2FB-01 | 0.1448 | 0.0000 | 0.0004 | 0.1080 | 0.0000 | 0.2765 | 0.0528 | 0.0438 | 0.0000 | 0.0000 | 0.0000 | 0.0000 | 0.0105 | 0.0060 | 0.1210 | 0.1296 | 0.0000 | 0.0222 | 0.0843 | 0.0000 | 0.0000 | 0.0000 |
| TCGA-AC-A2FE-01 | 0.0280 | 0.0000 | 0.0106 | 0.2181 | 0.0000 | 0.1081 | 0.0000 | 0.0877 | 0.0939 | 0.0000 | 0.0572 | 0.0137 | 0.0588 | 0.0313 | 0.1200 | 0.1477 | 0.0043 | 0.0000 | 0.0207 | 0.0000 | 0.0000 | 0.0000 |
| TCGA-AC-A2FF-01 | 0.1105 | 0.0000 | 0.0885 | 0.1015 | 0.0000 | 0.1540 | 0.0000 | 0.0545 | 0.0254 | 0.0000 | 0.0037 | 0.0000 | 0.0091 | 0.0011 | 0.0651 | 0.2615 | 0.0000 | 0.0102 | 0.1150 | 0.0000 | 0.0000 | 0.0000 |
| TCGA-AC-A2FG-01 | 0.0749 | 0.0000 | 0.0981 | 0.0465 | 0.0000 | 0.1370 | 0.0000 | 0.0309 | 0.0028 | 0.0000 | 0.0192 | 0.0000 | 0.0059 | 0.0943 | 0.0225 | 0.2303 | 0.0339 | 0.0000 | 0.2038 | 0.0000 | 0.0000 | 0.0000 |
| TCGA-AC-A2FK-01 | 0.0387 | 0.0000 | 0.0572 | 0.0822 | 0.0000 | 0.1108 | 0.0000 | 0.1125 | 0.0000 | 0.0000 | 0.0316 | 0.0025 | 0.0315 | 0.2041 | 0.0459 | 0.1836 | 0.0010 | 0.0000 | 0.0984 | 0.0000 | 0.0000 | 0.0000 |
| TCGA-AC-A2FM-01 | 0.0621 | 0.0000 | 0.0584 | 0.0284 | 0.0000 | 0.1573 | 0.0000 | 0.0554 | 0.0327 | 0.0000 | 0.0293 | 0.0134 | 0.0245 | 0.0000 | 0.0464 | 0.3173 | 0.0084 | 0.0000 | 0.1663 | 0.0000 | 0.0000 | 0.0000 |
| TCGA-AC-A2FO-01 | 0.1737 | 0.0000 | 0.0537 | 0.0782 | 0.0000 | 0.2491 | 0.0127 | 0.0374 | 0.0240 | 0.0027 | 0.0000 | 0.0000 | 0.0124 | 0.0000 | 0.0612 | 0.2312 | 0.0127 | 0.0000 | 0.0509 | 0.0000 | 0.0000 | 0.0000 |
| TCGA-AC-A2QH-01 | 0.0020 | 0.0105 | 0.0018 | 0.0000 | 0.0000 | 0.1471 | 0.0025 | 0.0283 | 0.0370 | 0.0000 | 0.0818 | 0.0000 | 0.0000 | 0.5317 | 0.0035 | 0.1519 | 0.0000 | 0.0000 | 0.0000 | 0.0020 | 0.0000 | 0.0000 |
| TCGA-AC-A2QI-01 | 0.1085 | 0.0000 | 0.0032 | 0.0370 | 0.0000 | 0.1551 | 0.0000 | 0.0977 | 0.0294 | 0.0000 | 0.0265 | 0.0000 | 0.0000 | 0.1548 | 0.0795 | 0.2817 | 0.0000 | 0.0000 | 0.0266 | 0.0000 | 0.0000 | 0.0000 |
| TCGA-AC-A2QJ-01 | 0.0058 | 0.0000 | 0.0000 | 0.0194 | 0.0000 | 0.0000 | 0.0000 | 0.0772 | 0.0290 | 0.0000 | 0.0000 | 0.0000 | 0.0133 | 0.3545 | 0.0118 | 0.4495 | 0.0000 | 0.0000 | 0.0395 | 0.0000 | 0.0000 | 0.0000 |
| TCGA-AC-A3BB-01 | 0.0708 | 0.0038 | 0.0185 | 0.1653 | 0.0000 | 0.1824 | 0.0000 | 0.1064 | 0.0395 | 0.0000 | 0.0180 | 0.0000 | 0.0084 | 0.0549 | 0.0507 | 0.2215 | 0.0000 | 0.0160 | 0.0438 | 0.0000 | 0.0000 | 0.0000 |
| TCGA-AC-A3EH-01 | 0.0464 | 0.0000 | 0.0211 | 0.0428 | 0.0000 | 0.1201 | 0.0000 | 0.0166 | 0.0202 | 0.0000 | 0.0164 | 0.0000 | 0.0000 | 0.4666 | 0.0422 | 0.1700 | 0.0000 | 0.0000 | 0.0377 | 0.0000 | 0.0000 | 0.0000 |
| TCGA-AC-A3HN-01 | 0.0429 | 0.0000 | 0.0270 | 0.0497 | 0.0000 | 0.0487 | 0.0000 | 0.0777 | 0.0384 | 0.0000 | 0.0174 | 0.0046 | 0.0000 | 0.2833 | 0.0493 | 0.2400 | 0.0000 | 0.0000 | 0.1210 | 0.0000 | 0.0000 | 0.0000 |
| TCGA-AC-A3OD-01 | 0.0482 | 0.0000 | 0.0109 | 0.1210 | 0.0000 | 0.1321 | 0.0000 | 0.0585 | 0.0369 | 0.0000 | 0.0282 | 0.0000 | 0.0000 | 0.2193 | 0.0535 | 0.2146 | 0.0070 | 0.0000 | 0.0698 | 0.0000 | 0.0000 | 0.0000 |
| TCGA-AC-A3QP-01 | 0.0204 | 0.0000 | 0.0534 | 0.1544 | 0.0000 | 0.0277 | 0.0000 | 0.1354 | 0.0042 | 0.0000 | 0.0000 | 0.0266 | 0.0257 | 0.0000 | 0.0225 | 0.3890 | 0.0000 | 0.0000 | 0.1407 | 0.0000 | 0.0000 | 0.0000 |
| TCGA-AC-A3QQ-01 | 0.0850 | 0.0000 | 0.0911 | 0.1352 | 0.0000 | 0.0451 | 0.0000 | 0.0614 | 0.0554 | 0.0000 | 0.0000 | 0.0432 | 0.0142 | 0.1221 | 0.0393 | 0.2348 | 0.0000 | 0.0000 | 0.0734 | 0.0000 | 0.0000 | 0.0000 |
| TCGA-AC-A3TM-01 | 0.0209 | 0.0000 | 0.0501 | 0.1881 | 0.0000 | 0.0842 | 0.0000 | 0.1065 | 0.0378 | 0.0000 | 0.0000 | 0.0231 | 0.0000 | 0.0915 | 0.0350 | 0.2925 | 0.0000 | 0.0000 | 0.0705 | 0.0000 | 0.0000 | 0.0000 |
| TCGA-AC-A3TN-01 | 0.0645 | 0.0000 | 0.0498 | 0.0601 | 0.0000 | 0.1658 | 0.0000 | 0.0540 | 0.0093 | 0.0000 | 0.0148 | 0.0000 | 0.0000 | 0.1719 | 0.0401 | 0.2814 | 0.0000 | 0.0051 | 0.0832 | 0.0000 | 0.0000 | 0.0000 |
| TCGA-AC-A3W5-01 | 0.0349 | 0.0000 | 0.0108 | 0.0782 | 0.0000 | 0.1333 | 0.0000 | 0.0616 | 0.0475 | 0.0000 | 0.0209 | 0.0000 | 0.0000 | 0.3098 | 0.0718 | 0.1755 | 0.0000 | 0.0000 | 0.0560 | 0.0000 | 0.0000 | 0.0000 |
| TCGA-AC-A3W6-01 | 0.0153 | 0.0000 | 0.0041 | 0.1540 | 0.0000 | 0.1467 | 0.0000 | 0.0584 | 0.0920 | 0.0000 | 0.0000 | 0.0120 | 0.0306 | 0.0176 | 0.1415 | 0.2007 | 0.0165 | 0.0000 | 0.1106 | 0.0000 | 0.0000 | 0.0000 |
| TCGA-AC-A3W7-01 | 0.0830 | 0.0000 | 0.0000 | 0.1151 | 0.0000 | 0.0939 | 0.0000 | 0.1629 | 0.0186 | 0.0000 | 0.0235 | 0.0010 | 0.0056 | 0.0494 | 0.0538 | 0.2613 | 0.0000 | 0.0084 | 0.1234 | 0.0000 | 0.0000 | 0.0000 |
| TCGA-AC-A3YI-01 | 0.0616 | 0.0000 | 0.0000 | 0.0260 | 0.0000 | 0.0595 | 0.0000 | 0.0278 | 0.0209 | 0.0000 | 0.0176 | 0.0000 | 0.0000 | 0.4521 | 0.0294 | 0.1675 | 0.0000 | 0.0243 | 0.1027 | 0.0106 | 0.0000 | 0.0000 |
| TCGA-AC-A3YJ-01 | 0.0350 | 0.0000 | 0.0000 | 0.0368 | 0.0000 | 0.0305 | 0.0000 | 0.0528 | 0.0098 | 0.0000 | 0.0114 | 0.0000 | 0.0260 | 0.0000 | 0.0200 | 0.7065 | 0.0033 | 0.0000 | 0.0680 | 0.0000 | 0.0000 | 0.0000 |
| TCGA-AC-A4ZE-01 | 0.1000 | 0.0000 | 0.0000 | 0.1013 | 0.0000 | 0.1723 | 0.0000 | 0.1319 | 0.0047 | 0.0069 | 0.0000 | 0.0000 | 0.0070 | 0.0000 | 0.0431 | 0.3020 | 0.0000 | 0.0000 | 0.1308 | 0.0000 | 0.0000 | 0.0000 |
| TCGA-AC-A5EH-01 | 0.0000 | 0.0000 | 0.0002 | 0.0000 | 0.0000 | 0.0216 | 0.0000 | 0.1241 | 0.0468 | 0.0000 | 0.0000 | 0.0217 | 0.0000 | 0.4577 | 0.0513 | 0.2108 | 0.0000 | 0.0000 | 0.0658 | 0.0000 | 0.0000 | 0.0000 |
| TCGA-AC-A5XS-01 | 0.0604 | 0.0000 | 0.0598 | 0.0832 | 0.0000 | 0.2254 | 0.0000 | 0.0850 | 0.0137 | 0.0000 | 0.0000 | 0.0000 | 0.0041 | 0.0000 | 0.0979 | 0.3297 | 0.0000 | 0.0030 | 0.0151 | 0.0000 | 0.0000 | 0.0227 |
| TCGA-AC-A5XU-01 | 0.0191 | 0.0000 | 0.0941 | 0.0512 | 0.0000 | 0.0854 | 0.0000 | 0.0544 | 0.0310 | 0.0000 | 0.0018 | 0.0179 | 0.0000 | 0.3166 | 0.0436 | 0.2066 | 0.0000 | 0.0000 | 0.0782 | 0.0000 | 0.0000 | 0.0000 |
| TCGA-AC-A62V-01 | 0.0021 | 0.0000 | 0.0340 | 0.0000 | 0.0049 | 0.0440 | 0.0000 | 0.0199 | 0.0381 | 0.0000 | 0.0669 | 0.0000 | 0.0031 | 0.3133 | 0.0000 | 0.3525 | 0.0134 | 0.0000 | 0.1077 | 0.0000 | 0.0000 | 0.0000 |
| TCGA-AC-A62X-01 | 0.0108 | 0.0000 | 0.0000 | 0.0192 | 0.0000 | 0.0000 | 0.0000 | 0.1810 | 0.0159 | 0.0000 | 0.0000 | 0.0526 | 0.0596 | 0.3736 | 0.0380 | 0.0000 | 0.0000 | 0.1659 | 0.0391 | 0.0000 | 0.0000 | 0.0442 |
| TCGA-AC-A62Y-01 | 0.0385 | 0.0000 | 0.0000 | 0.0195 | 0.0000 | 0.0773 | 0.0000 | 0.0295 | 0.0000 | 0.0000 | 0.0141 | 0.0000 | 0.0000 | 0.5070 | 0.0379 | 0.2569 | 0.0000 | 0.0000 | 0.0193 | 0.0000 | 0.0000 | 0.0000 |
| TCGA-AC-A6IV-01 | 0.0609 | 0.0000 | 0.0025 | 0.0753 | 0.0000 | 0.1661 | 0.0000 | 0.0414 | 0.0403 | 0.0000 | 0.0348 | 0.0000 | 0.0066 | 0.0555 | 0.0712 | 0.3417 | 0.0000 | 0.0000 | 0.1030 | 0.0000 | 0.0000 | 0.0006 |
| TCGA-AC-A6IW-01 | 0.0102 | 0.0063 | 0.0000 | 0.0000 | 0.0000 | 0.0797 | 0.0000 | 0.2019 | 0.0037 | 0.0000 | 0.0000 | 0.0398 | 0.0063 | 0.0531 | 0.1795 | 0.2789 | 0.0694 | 0.0000 | 0.0713 | 0.0000 | 0.0000 | 0.0000 |
| TCGA-AC-A6IX-01 | 0.1064 | 0.0000 | 0.0784 | 0.0671 | 0.0000 | 0.1710 | 0.0000 | 0.0262 | 0.0263 | 0.0000 | 0.0293 | 0.0000 | 0.0138 | 0.0000 | 0.0511 | 0.3292 | 0.0000 | 0.0223 | 0.0789 | 0.0000 | 0.0000 | 0.0000 |
| TCGA-AC-A6NO-01 | 0.0867 | 0.0000 | 0.0175 | 0.0742 | 0.0000 | 0.0000 | 0.0000 | 0.2472 | 0.0213 | 0.0000 | 0.0602 | 0.0000 | 0.0232 | 0.2132 | 0.0654 | 0.1432 | 0.0000 | 0.0000 | 0.0478 | 0.0000 | 0.0000 | 0.0000 |
| TCGA-AC-A7VB-01 | 0.1254 | 0.0000 | 0.1114 | 0.0491 | 0.0000 | 0.0000 | 0.0000 | 0.0412 | 0.0768 | 0.0000 | 0.0293 | 0.0000 | 0.0119 | 0.2485 | 0.0169 | 0.2279 | 0.0000 | 0.0005 | 0.0611 | 0.0000 | 0.0000 | 0.0000 |
| TCGA-AC-A7VC-01 | 0.0000 | 0.0259 | 0.0224 | 0.0000 | 0.0000 | 0.0584 | 0.0209 | 0.1094 | 0.0240 | 0.0000 | 0.0869 | 0.0010 | 0.0000 | 0.5351 | 0.0000 | 0.0772 | 0.0192 | 0.0000 | 0.0137 | 0.0059 | 0.0000 | 0.0000 |
| TCGA-AC-A8OP-01 | 0.1532 | 0.0000 | 0.0000 | 0.0501 | 0.0000 | 0.1036 | 0.0000 | 0.0990 | 0.0028 | 0.0000 | 0.0291 | 0.0000 | 0.0000 | 0.1720 | 0.0723 | 0.2463 | 0.0000 | 0.0000 | 0.0668 | 0.0048 | 0.0000 | 0.0000 |
| TCGA-AC-A8OQ-01 | 0.0585 | 0.0000 | 0.1649 | 0.0287 | 0.0000 | 0.0431 | 0.0000 | 0.0812 | 0.1071 | 0.0000 | 0.0539 | 0.0000 | 0.0248 | 0.2321 | 0.0813 | 0.0731 | 0.0000 | 0.0000 | 0.0513 | 0.0000 | 0.0000 | 0.0000 |
| TCGA-AC-A8OR-01 | 0.0023 | 0.0034 | 0.0093 | 0.0000 | 0.0015 | 0.1092 | 0.0000 | 0.0110 | 0.0747 | 0.0000 | 0.0858 | 0.0000 | 0.0150 | 0.3845 | 0.0094 | 0.2319 | 0.0000 | 0.0000 | 0.0620 | 0.0000 | 0.0000 | 0.0000 |
| TCGA-AC-A8OS-01 | 0.0609 | 0.0000 | 0.0172 | 0.1431 | 0.0000 | 0.1086 | 0.0000 | 0.1076 | 0.0165 | 0.0000 | 0.0394 | 0.0000 | 0.0344 | 0.0095 | 0.0559 | 0.1851 | 0.0000 | 0.0000 | 0.2218 | 0.0000 | 0.0000 | 0.0000 |
| TCGA-AN-A03X-01 | 0.0625 | 0.0000 | 0.0000 | 0.0289 | 0.0000 | 0.0893 | 0.0000 | 0.0435 | 0.0000 | 0.0000 | 0.0086 | 0.0039 | 0.0325 | 0.0000 | 0.0419 | 0.4651 | 0.0000 | 0.0234 | 0.1588 | 0.0000 | 0.0000 | 0.0416 |
| TCGA-AN-A03Y-01 | 0.0535 | 0.0000 | 0.0103 | 0.1961 | 0.0000 | 0.0874 | 0.0000 | 0.1423 | 0.0503 | 0.0000 | 0.0190 | 0.0000 | 0.0017 | 0.0260 | 0.0934 | 0.2416 | 0.0005 | 0.0000 | 0.0779 | 0.0000 | 0.0000 | 0.0000 |
| TCGA-AN-A041-01 | 0.0400 | 0.0000 | 0.0731 | 0.0000 | 0.0000 | 0.0860 | 0.0000 | 0.0451 | 0.0246 | 0.0155 | 0.0000 | 0.0174 | 0.0000 | 0.3606 | 0.0568 | 0.2332 | 0.0000 | 0.0000 | 0.0477 | 0.0000 | 0.0000 | 0.0000 |
| TCGA-AN-A046-01 | 0.0472 | 0.0000 | 0.0099 | 0.1212 | 0.0000 | 0.0590 | 0.0053 | 0.1358 | 0.0357 | 0.0000 | 0.0740 | 0.0000 | 0.0314 | 0.0793 | 0.1176 | 0.1679 | 0.0169 | 0.0188 | 0.0798 | 0.0000 | 0.0000 | 0.0000 |
| TCGA-AN-A049-01 | 0.0896 | 0.0000 | 0.0354 | 0.0282 | 0.0000 | 0.1463 | 0.0034 | 0.0368 | 0.0187 | 0.0000 | 0.0241 | 0.0000 | 0.0000 | 0.1823 | 0.0656 | 0.3258 | 0.0000 | 0.0000 | 0.0400 | 0.0000 | 0.0000 | 0.0039 |
| TCGA-AN-A04A-01 | 0.1220 | 0.0000 | 0.0483 | 0.0136 | 0.0000 | 0.1190 | 0.0000 | 0.0175 | 0.0279 | 0.0000 | 0.0265 | 0.0000 | 0.0000 | 0.2751 | 0.0730 | 0.2293 | 0.0128 | 0.0000 | 0.0350 | 0.0000 | 0.0000 | 0.0000 |
| TCGA-AN-A04C-01 | 0.0754 | 0.0000 | 0.0604 | 0.0438 | 0.0000 | 0.1395 | 0.0000 | 0.1305 | 0.0221 | 0.0000 | 0.0023 | 0.0076 | 0.0022 | 0.0493 | 0.0840 | 0.3254 | 0.0109 | 0.0256 | 0.0210 | 0.0000 | 0.0000 | 0.0000 |
| TCGA-AN-A04D-01 | 0.0310 | 0.0000 | 0.0000 | 0.0874 | 0.0000 | 0.0246 | 0.0000 | 0.2353 | 0.0000 | 0.0000 | 0.0247 | 0.0349 | 0.0384 | 0.0813 | 0.0264 | 0.2723 | 0.0045 | 0.0501 | 0.0890 | 0.0000 | 0.0000 | 0.0000 |
| TCGA-AN-A0AJ-01 | 0.0285 | 0.0000 | 0.0114 | 0.0796 | 0.0000 | 0.1133 | 0.0193 | 0.1003 | 0.0328 | 0.0000 | 0.0179 | 0.0000 | 0.0000 | 0.2527 | 0.0928 | 0.2027 | 0.0000 | 0.0008 | 0.0479 | 0.0000 | 0.0000 | 0.0000 |
| TCGA-AN-A0AK-01 | 0.0539 | 0.0000 | 0.0549 | 0.0572 | 0.0000 | 0.1135 | 0.0000 | 0.0628 | 0.0281 | 0.0000 | 0.0000 | 0.0020 | 0.0000 | 0.2449 | 0.0750 | 0.2907 | 0.0000 | 0.0000 | 0.0170 | 0.0000 | 0.0000 | 0.0000 |
| TCGA-AN-A0AL-01 | 0.1507 | 0.0000 | 0.0616 | 0.0069 | 0.0000 | 0.1576 | 0.0070 | 0.0876 | 0.0120 | 0.0000 | 0.0264 | 0.0000 | 0.0000 | 0.1814 | 0.0416 | 0.1627 | 0.0000 | 0.0677 | 0.0366 | 0.0000 | 0.0000 | 0.0000 |
| TCGA-AN-A0AM-01 | 0.0915 | 0.0000 | 0.0534 | 0.0885 | 0.0000 | 0.1064 | 0.0028 | 0.1115 | 0.0637 | 0.0000 | 0.0473 | 0.0000 | 0.0043 | 0.0732 | 0.1377 | 0.1885 | 0.0000 | 0.0118 | 0.0194 | 0.0000 | 0.0000 | 0.0000 |
| TCGA-AN-A0AR-01 | 0.0759 | 0.0000 | 0.0628 | 0.0334 | 0.0000 | 0.1319 | 0.0000 | 0.0962 | 0.0398 | 0.0000 | 0.0000 | 0.0204 | 0.0045 | 0.1262 | 0.0340 | 0.3010 | 0.0542 | 0.0000 | 0.0196 | 0.0000 | 0.0000 | 0.0000 |
| TCGA-AN-A0AS-01 | 0.0020 | 0.0000 | 0.0308 | 0.0000 | 0.0000 | 0.1078 | 0.0047 | 0.0411 | 0.0663 | 0.0000 | 0.0396 | 0.0000 | 0.0000 | 0.5866 | 0.0000 | 0.0595 | 0.0000 | 0.0000 | 0.0599 | 0.0000 | 0.0000 | 0.0019 |
| TCGA-AN-A0AT-01 | 0.0899 | 0.0000 | 0.0017 | 0.0000 | 0.0000 | 0.0984 | 0.0000 | 0.1302 | 0.0043 | 0.0000 | 0.0328 | 0.0000 | 0.0000 | 0.3906 | 0.0173 | 0.2115 | 0.0000 | 0.0000 | 0.0000 | 0.0232 | 0.0000 | 0.0000 |
| TCGA-AN-A0FD-01 | 0.0018 | 0.0000 | 0.0509 | 0.0207 | 0.0000 | 0.0680 | 0.0000 | 0.0793 | 0.0826 | 0.0000 | 0.0000 | 0.0326 | 0.0000 | 0.3435 | 0.1308 | 0.1286 | 0.0000 | 0.0000 | 0.0612 | 0.0000 | 0.0000 | 0.0000 |
| TCGA-AN-A0FF-01 | 0.0614 | 0.0000 | 0.0206 | 0.0324 | 0.0000 | 0.1209 | 0.0000 | 0.0535 | 0.0112 | 0.0000 | 0.0192 | 0.0000 | 0.0000 | 0.3542 | 0.0432 | 0.2446 | 0.0000 | 0.0000 | 0.0388 | 0.0000 | 0.0000 | 0.0000 |
| TCGA-AN-A0FJ-01 | 0.0409 | 0.0000 | 0.0879 | 0.0000 | 0.0000 | 0.1465 | 0.0000 | 0.1108 | 0.0000 | 0.0000 | 0.0390 | 0.0045 | 0.0000 | 0.3433 | 0.1022 | 0.1214 | 0.0000 | 0.0000 | 0.0036 | 0.0000 | 0.0000 | 0.0000 |
| TCGA-AN-A0FK-01 | 0.1153 | 0.0000 | 0.0866 | 0.0438 | 0.0000 | 0.0231 | 0.0000 | 0.0159 | 0.0279 | 0.0000 | 0.0000 | 0.0038 | 0.0000 | 0.3653 | 0.0695 | 0.2048 | 0.0000 | 0.0000 | 0.0438 | 0.0000 | 0.0000 | 0.0000 |
| TCGA-AN-A0FL-01 | 0.0601 | 0.0000 | 0.0228 | 0.0132 | 0.0000 | 0.1018 | 0.0000 | 0.0325 | 0.0087 | 0.0000 | 0.0165 | 0.0000 | 0.0000 | 0.3896 | 0.0209 | 0.2547 | 0.0000 | 0.0465 | 0.0327 | 0.0000 | 0.0000 | 0.0000 |
| TCGA-AN-A0FN-01 | 0.0895 | 0.0107 | 0.0199 | 0.0404 | 0.0000 | 0.2691 | 0.0018 | 0.0983 | 0.0008 | 0.0687 | 0.0000 | 0.0000 | 0.0000 | 0.0424 | 0.0922 | 0.1950 | 0.0000 | 0.0027 | 0.0684 | 0.0000 | 0.0000 | 0.0000 |
| TCGA-AN-A0FS-01 | 0.0003 | 0.0000 | 0.0313 | 0.0000 | 0.0000 | 0.3563 | 0.0000 | 0.0807 | 0.0004 | 0.0000 | 0.0000 | 0.0301 | 0.0463 | 0.0477 | 0.0022 | 0.1239 | 0.0011 | 0.0000 | 0.2797 | 0.0000 | 0.0000 | 0.0000 |
| TCGA-AN-A0FT-01 | 0.0520 | 0.0000 | 0.0074 | 0.0951 | 0.0000 | 0.1483 | 0.0000 | 0.0350 | 0.0000 | 0.0000 | 0.0000 | 0.0330 | 0.0056 | 0.0207 | 0.0696 | 0.4868 | 0.0000 | 0.0000 | 0.0465 | 0.0000 | 0.0000 | 0.0000 |
| TCGA-AN-A0FV-01 | 0.0000 | 0.0000 | 0.0313 | 0.0000 | 0.0000 | 0.0284 | 0.0000 | 0.1048 | 0.0081 | 0.0171 | 0.0000 | 0.0226 | 0.0000 | 0.4559 | 0.0212 | 0.2012 | 0.0000 | 0.0000 | 0.1092 | 0.0000 | 0.0000 | 0.0000 |
| TCGA-AN-A0FW-01 | 0.1084 | 0.0000 | 0.0503 | 0.1557 | 0.0000 | 0.1623 | 0.0020 | 0.0281 | 0.0135 | 0.0000 | 0.0000 | 0.0000 | 0.0110 | 0.0000 | 0.0843 | 0.3114 | 0.0217 | 0.0000 | 0.0511 | 0.0000 | 0.0000 | 0.0000 |
| TCGA-AN-A0FX-01 | 0.1429 | 0.0000 | 0.0836 | 0.0309 | 0.0000 | 0.0120 | 0.0000 | 0.0702 | 0.0766 | 0.0000 | 0.0145 | 0.0081 | 0.0000 | 0.3125 | 0.0947 | 0.1541 | 0.0000 | 0.0000 | 0.0000 | 0.0000 | 0.0000 | 0.0000 |
| TCGA-AN-A0FY-01 | 0.0139 | 0.0000 | 0.0000 | 0.0000 | 0.0000 | 0.2330 | 0.0000 | 0.0842 | 0.0595 | 0.0000 | 0.0289 | 0.0000 | 0.0201 | 0.2790 | 0.0222 | 0.2149 | 0.0000 | 0.0000 | 0.0444 | 0.0000 | 0.0000 | 0.0000 |
| TCGA-AN-A0FZ-01 | 0.0000 | 0.0100 | 0.0290 | 0.0000 | 0.0000 | 0.2387 | 0.0000 | 0.0421 | 0.0318 | 0.0000 | 0.1164 | 0.0000 | 0.0000 | 0.3231 | 0.0140 | 0.1780 | 0.0100 | 0.0000 | 0.0069 | 0.0000 | 0.0000 | 0.0000 |
| TCGA-AN-A0G0-01 | 0.0356 | 0.0000 | 0.0388 | 0.0085 | 0.0000 | 0.1084 | 0.0000 | 0.0818 | 0.0405 | 0.0000 | 0.0636 | 0.0000 | 0.0011 | 0.2841 | 0.0741 | 0.2441 | 0.0000 | 0.0000 | 0.0193 | 0.0000 | 0.0000 | 0.0000 |
| TCGA-AN-A0XL-01 | 0.0000 | 0.0004 | 0.0108 | 0.0000 | 0.0000 | 0.2423 | 0.0000 | 0.1191 | 0.0080 | 0.0000 | 0.0709 | 0.0000 | 0.0500 | 0.1541 | 0.0000 | 0.2719 | 0.0000 | 0.0000 | 0.0670 | 0.0000 | 0.0055 | 0.0000 |
| TCGA-AN-A0XN-01 | 0.0015 | 0.0033 | 0.0006 | 0.0000 | 0.0000 | 0.0663 | 0.0000 | 0.0282 | 0.0445 | 0.0000 | 0.0000 | 0.0324 | 0.0000 | 0.4953 | 0.0020 | 0.2580 | 0.0000 | 0.0121 | 0.0558 | 0.0000 | 0.0000 | 0.0000 |
| TCGA-AN-A0XO-01 | 0.0023 | 0.0004 | 0.0267 | 0.0000 | 0.0000 | 0.1744 | 0.0000 | 0.0165 | 0.0362 | 0.0000 | 0.0665 | 0.0030 | 0.0000 | 0.5527 | 0.0000 | 0.0026 | 0.0000 | 0.0932 | 0.0256 | 0.0000 | 0.0000 | 0.0000 |
| TCGA-AN-A0XP-01 | 0.0932 | 0.0000 | 0.0210 | 0.0060 | 0.0000 | 0.0645 | 0.0000 | 0.0511 | 0.0242 | 0.0000 | 0.0134 | 0.0014 | 0.0000 | 0.0974 | 0.0561 | 0.4536 | 0.0000 | 0.0304 | 0.0675 | 0.0000 | 0.0000 | 0.0201 |
| TCGA-AN-A0XR-01 | 0.0126 | 0.0000 | 0.0000 | 0.0000 | 0.0000 | 0.2147 | 0.0000 | 0.0244 | 0.0785 | 0.0000 | 0.0626 | 0.0000 | 0.0000 | 0.4592 | 0.0000 | 0.0389 | 0.0000 | 0.0236 | 0.0856 | 0.0000 | 0.0000 | 0.0000 |
| TCGA-AN-A0XS-01 | 0.0506 | 0.0058 | 0.1271 | 0.1004 | 0.0000 | 0.1312 | 0.0000 | 0.0999 | 0.0611 | 0.0000 | 0.0272 | 0.0000 | 0.0118 | 0.1144 | 0.0807 | 0.1502 | 0.0000 | 0.0000 | 0.0395 | 0.0000 | 0.0000 | 0.0000 |
| TCGA-AN-A0XT-01 | 0.0969 | 0.0000 | 0.0193 | 0.0560 | 0.0000 | 0.1304 | 0.0000 | 0.0302 | 0.0364 | 0.0000 | 0.0212 | 0.0000 | 0.0034 | 0.0798 | 0.0871 | 0.2382 | 0.0000 | 0.0000 | 0.2011 | 0.0000 | 0.0000 | 0.0000 |
| TCGA-AN-A0XU-01 | 0.0495 | 0.0000 | 0.0503 | 0.0000 | 0.0000 | 0.0922 | 0.0000 | 0.1284 | 0.0003 | 0.0000 | 0.0128 | 0.0495 | 0.0000 | 0.2832 | 0.1657 | 0.1376 | 0.0000 | 0.0000 | 0.0307 | 0.0000 | 0.0000 | 0.0000 |
| TCGA-AN-A0XV-01 | 0.1460 | 0.0000 | 0.0466 | 0.0547 | 0.0000 | 0.1705 | 0.0000 | 0.1177 | 0.0053 | 0.0484 | 0.0000 | 0.0000 | 0.0000 | 0.0918 | 0.0992 | 0.1546 | 0.0000 | 0.0000 | 0.0653 | 0.0000 | 0.0000 | 0.0000 |
| TCGA-AN-A0XW-01 | 0.0239 | 0.0000 | 0.0212 | 0.0184 | 0.0000 | 0.1792 | 0.0000 | 0.1669 | 0.0100 | 0.0000 | 0.0611 | 0.0053 | 0.0120 | 0.0260 | 0.1549 | 0.1796 | 0.0054 | 0.0000 | 0.1361 | 0.0000 | 0.0000 | 0.0000 |
| TCGA-AO-A03L-01 | 0.0881 | 0.0000 | 0.0928 | 0.0836 | 0.0000 | 0.2415 | 0.0000 | 0.0696 | 0.0156 | 0.0000 | 0.0255 | 0.0000 | 0.0339 | 0.0000 | 0.0723 | 0.2152 | 0.0000 | 0.0222 | 0.0398 | 0.0000 | 0.0000 | 0.0000 |
| TCGA-AO-A03M-01 | 0.0857 | 0.0000 | 0.0189 | 0.1082 | 0.0000 | 0.1156 | 0.0000 | 0.1283 | 0.0051 | 0.0000 | 0.0356 | 0.0199 | 0.0294 | 0.0932 | 0.1174 | 0.1426 | 0.0018 | 0.0000 | 0.0984 | 0.0000 | 0.0000 | 0.0000 |
| TCGA-AO-A03N-01 | 0.0020 | 0.0000 | 0.0101 | 0.0000 | 0.0000 | 0.0992 | 0.0000 | 0.0427 | 0.0373 | 0.0000 | 0.0000 | 0.0079 | 0.0000 | 0.5921 | 0.0000 | 0.1097 | 0.0000 | 0.0000 | 0.0991 | 0.0000 | 0.0000 | 0.0000 |
| TCGA-AO-A03O-01 | 0.0122 | 0.0000 | 0.0178 | 0.0000 | 0.0000 | 0.1790 | 0.0000 | 0.1149 | 0.0619 | 0.0000 | 0.0442 | 0.0000 | 0.0058 | 0.1608 | 0.1287 | 0.2032 | 0.0000 | 0.0000 | 0.0715 | 0.0000 | 0.0000 | 0.0000 |
| TCGA-AO-A03P-01 | 0.0823 | 0.0000 | 0.0360 | 0.0162 | 0.0000 | 0.0501 | 0.0000 | 0.0651 | 0.0521 | 0.0000 | 0.0081 | 0.0000 | 0.0000 | 0.2815 | 0.0452 | 0.3104 | 0.0000 | 0.0042 | 0.0488 | 0.0000 | 0.0000 | 0.0000 |
| TCGA-AO-A03R-01 | 0.0396 | 0.0000 | 0.0321 | 0.1246 | 0.0000 | 0.0028 | 0.0000 | 0.1858 | 0.0869 | 0.0000 | 0.0000 | 0.0000 | 0.0384 | 0.0529 | 0.1695 | 0.1838 | 0.0113 | 0.0000 | 0.0725 | 0.0000 | 0.0000 | 0.0000 |
| TCGA-AO-A03T-01 | 0.0863 | 0.0000 | 0.0824 | 0.0602 | 0.0000 | 0.1135 | 0.0000 | 0.0744 | 0.0234 | 0.0000 | 0.0000 | 0.0384 | 0.0000 | 0.2225 | 0.1251 | 0.1379 | 0.0000 | 0.0000 | 0.0358 | 0.0000 | 0.0000 | 0.0000 |
| TCGA-AO-A03U-01 | 0.0000 | 0.0018 | 0.0122 | 0.0396 | 0.0000 | 0.0964 | 0.0000 | 0.0139 | 0.0000 | 0.0000 | 0.0748 | 0.0656 | 0.0537 | 0.0000 | 0.0061 | 0.4921 | 0.0267 | 0.0163 | 0.1010 | 0.0000 | 0.0000 | 0.0000 |
| TCGA-AO-A03V-01 | 0.0445 | 0.0000 | 0.1102 | 0.0365 | 0.0000 | 0.1163 | 0.0000 | 0.0860 | 0.0544 | 0.0000 | 0.0204 | 0.0213 | 0.0163 | 0.1013 | 0.1191 | 0.2310 | 0.0000 | 0.0000 | 0.0427 | 0.0000 | 0.0000 | 0.0000 |
| TCGA-AO-A0J2-01 | 0.0055 | 0.0000 | 0.0126 | 0.0260 | 0.0000 | 0.0752 | 0.0000 | 0.2286 | 0.0359 | 0.0000 | 0.0091 | 0.0795 | 0.0363 | 0.1605 | 0.1127 | 0.0692 | 0.0038 | 0.0007 | 0.1283 | 0.0000 | 0.0161 | 0.0000 |
| TCGA-AO-A0J3-01 | 0.0686 | 0.0000 | 0.0263 | 0.0585 | 0.0000 | 0.1440 | 0.0000 | 0.0409 | 0.0242 | 0.0000 | 0.0163 | 0.0222 | 0.0041 | 0.0808 | 0.0506 | 0.3648 | 0.0000 | 0.0000 | 0.0988 | 0.0000 | 0.0000 | 0.0000 |
| TCGA-AO-A0J4-01 | 0.1031 | 0.0119 | 0.0000 | 0.0089 | 0.0000 | 0.1529 | 0.0000 | 0.1557 | 0.0245 | 0.0000 | 0.0678 | 0.0000 | 0.0000 | 0.1943 | 0.1836 | 0.0759 | 0.0000 | 0.0000 | 0.0215 | 0.0000 | 0.0000 | 0.0000 |
| TCGA-AO-A0J5-01 | 0.0000 | 0.0000 | 0.0346 | 0.0482 | 0.0000 | 0.1931 | 0.0000 | 0.0637 | 0.0021 | 0.0000 | 0.0218 | 0.0000 | 0.0315 | 0.1830 | 0.0000 | 0.2191 | 0.0000 | 0.0000 | 0.2028 | 0.0000 | 0.0000 | 0.0000 |
| TCGA-AO-A0J6-01 | 0.0125 | 0.0000 | 0.0312 | 0.0740 | 0.0000 | 0.0832 | 0.0000 | 0.1658 | 0.0183 | 0.0000 | 0.0651 | 0.0469 | 0.0087 | 0.0893 | 0.1300 | 0.0947 | 0.0000 | 0.1656 | 0.0148 | 0.0000 | 0.0000 | 0.0000 |
| TCGA-AO-A0J7-01 | 0.0688 | 0.0000 | 0.0494 | 0.0011 | 0.0000 | 0.0955 | 0.0000 | 0.0629 | 0.0290 | 0.0014 | 0.0000 | 0.0023 | 0.0000 | 0.2624 | 0.0558 | 0.2847 | 0.0237 | 0.0000 | 0.0631 | 0.0000 | 0.0000 | 0.0000 |
| TCGA-AO-A0J8-01 | 0.0574 | 0.0000 | 0.0000 | 0.0364 | 0.0000 | 0.1234 | 0.0000 | 0.0402 | 0.0429 | 0.0000 | 0.0102 | 0.0000 | 0.0000 | 0.5002 | 0.0304 | 0.1001 | 0.0000 | 0.0004 | 0.0585 | 0.0000 | 0.0000 | 0.0000 |
| TCGA-AO-A0J9-01 | 0.0389 | 0.0000 | 0.0071 | 0.0882 | 0.0000 | 0.2120 | 0.0000 | 0.0263 | 0.0584 | 0.0000 | 0.0085 | 0.0000 | 0.0102 | 0.1464 | 0.0566 | 0.2852 | 0.0107 | 0.0000 | 0.0514 | 0.0000 | 0.0000 | 0.0000 |
| TCGA-AO-A0JA-01 | 0.0914 | 0.0000 | 0.0584 | 0.0258 | 0.0000 | 0.2183 | 0.0000 | 0.0133 | 0.0301 | 0.0000 | 0.0172 | 0.0000 | 0.0000 | 0.0000 | 0.0688 | 0.3922 | 0.0000 | 0.0192 | 0.0566 | 0.0000 | 0.0000 | 0.0087 |
| TCGA-AO-A0JB-01 | 0.0891 | 0.0000 | 0.0517 | 0.1042 | 0.0000 | 0.0982 | 0.0000 | 0.1155 | 0.0403 | 0.0000 | 0.0000 | 0.0208 | 0.0095 | 0.0388 | 0.0320 | 0.3389 | 0.0000 | 0.0331 | 0.0280 | 0.0000 | 0.0000 | 0.0000 |
| TCGA-AO-A0JC-01 | 0.3401 | 0.0323 | 0.0000 | 0.1805 | 0.0000 | 0.0599 | 0.0273 | 0.0794 | 0.0665 | 0.0097 | 0.0215 | 0.0000 | 0.0000 | 0.0324 | 0.0474 | 0.0990 | 0.0000 | 0.0000 | 0.0000 | 0.0038 | 0.0000 | 0.0000 |
| TCGA-AO-A0JD-01 | 0.0934 | 0.0000 | 0.0088 | 0.0670 | 0.0000 | 0.1319 | 0.0000 | 0.1277 | 0.0274 | 0.0000 | 0.0120 | 0.0095 | 0.0111 | 0.0985 | 0.1060 | 0.2002 | 0.0359 | 0.0000 | 0.0706 | 0.0000 | 0.0000 | 0.0000 |
| TCGA-AO-A0JE-01 | 0.0672 | 0.0000 | 0.0459 | 0.0838 | 0.0000 | 0.1449 | 0.0081 | 0.1117 | 0.0750 | 0.0000 | 0.0109 | 0.0000 | 0.0049 | 0.0021 | 0.0884 | 0.2602 | 0.0007 | 0.0283 | 0.0655 | 0.0000 | 0.0000 | 0.0024 |
| TCGA-AO-A0JF-01 | 0.0452 | 0.0000 | 0.0210 | 0.0712 | 0.0000 | 0.1883 | 0.0000 | 0.0193 | 0.0072 | 0.0000 | 0.0118 | 0.0000 | 0.0148 | 0.0014 | 0.0392 | 0.3643 | 0.0402 | 0.0065 | 0.1695 | 0.0000 | 0.0000 | 0.0000 |
| TCGA-AO-A0JG-01 | 0.0872 | 0.0000 | 0.0139 | 0.1534 | 0.0000 | 0.1635 | 0.0000 | 0.1439 | 0.0480 | 0.0135 | 0.0139 | 0.0000 | 0.0000 | 0.0592 | 0.0829 | 0.1673 | 0.0032 | 0.0000 | 0.0502 | 0.0000 | 0.0000 | 0.0000 |
| TCGA-AO-A0JI-01 | 0.1134 | 0.0000 | 0.0353 | 0.0346 | 0.0000 | 0.0946 | 0.0000 | 0.0267 | 0.0128 | 0.0000 | 0.0000 | 0.0169 | 0.0000 | 0.0003 | 0.0378 | 0.0700 | 0.0000 | 0.0000 | 0.5575 | 0.0000 | 0.0000 | 0.0000 |
| TCGA-AO-A0JJ-01 | 0.1182 | 0.0000 | 0.0855 | 0.0737 | 0.0000 | 0.2056 | 0.0000 | 0.0671 | 0.0306 | 0.0000 | 0.0023 | 0.0000 | 0.0298 | 0.0000 | 0.0467 | 0.2750 | 0.0026 | 0.0010 | 0.0618 | 0.0000 | 0.0000 | 0.0000 |
| TCGA-AO-A0JL-01 | 0.1210 | 0.0000 | 0.1271 | 0.0019 | 0.0000 | 0.0253 | 0.0000 | 0.0869 | 0.0265 | 0.0000 | 0.0000 | 0.0210 | 0.0000 | 0.3320 | 0.0600 | 0.1533 | 0.0000 | 0.0000 | 0.0449 | 0.0000 | 0.0000 | 0.0000 |
| TCGA-AO-A0JM-01 | 0.0697 | 0.0000 | 0.0218 | 0.0164 | 0.0000 | 0.1161 | 0.0000 | 0.0514 | 0.0478 | 0.0000 | 0.0138 | 0.0000 | 0.0000 | 0.4223 | 0.0480 | 0.1654 | 0.0000 | 0.0000 | 0.0272 | 0.0000 | 0.0000 | 0.0000 |
| TCGA-AO-A124-01 | 0.1699 | 0.0000 | 0.0054 | 0.0000 | 0.0000 | 0.1465 | 0.0000 | 0.1283 | 0.0300 | 0.0000 | 0.0401 | 0.0000 | 0.0000 | 0.2874 | 0.0109 | 0.0672 | 0.0000 | 0.0933 | 0.0000 | 0.0210 | 0.0000 | 0.0000 |
| TCGA-AO-A125-01 | 0.0743 | 0.0000 | 0.0194 | 0.0000 | 0.0000 | 0.0044 | 0.0000 | 0.1990 | 0.0000 | 0.0000 | 0.0000 | 0.0534 | 0.0069 | 0.2824 | 0.0000 | 0.1923 | 0.0000 | 0.1164 | 0.0514 | 0.0000 | 0.0000 | 0.0000 |
| TCGA-AO-A126-01 | 0.0894 | 0.0000 | 0.0618 | 0.0618 | 0.0000 | 0.1447 | 0.0000 | 0.0665 | 0.0340 | 0.0000 | 0.0000 | 0.0113 | 0.0046 | 0.0000 | 0.0513 | 0.3300 | 0.0000 | 0.0000 | 0.1445 | 0.0000 | 0.0000 | 0.0000 |
| TCGA-AO-A128-01 | 0.0414 | 0.0000 | 0.0000 | 0.1079 | 0.0000 | 0.0967 | 0.0649 | 0.1046 | 0.0314 | 0.0000 | 0.0288 | 0.0074 | 0.0000 | 0.0649 | 0.2376 | 0.1454 | 0.0000 | 0.0000 | 0.0689 | 0.0000 | 0.0000 | 0.0000 |
| TCGA-AO-A129-01 | 0.0650 | 0.0362 | 0.0368 | 0.0891 | 0.0000 | 0.1172 | 0.0071 | 0.1390 | 0.0194 | 0.0000 | 0.0831 | 0.0000 | 0.0040 | 0.1571 | 0.1353 | 0.1067 | 0.0000 | 0.0039 | 0.0000 | 0.0000 | 0.0000 | 0.0000 |
| TCGA-AO-A12A-01 | 0.0671 | 0.0000 | 0.0139 | 0.1805 | 0.0000 | 0.0095 | 0.0000 | 0.1208 | 0.0739 | 0.0000 | 0.0244 | 0.0084 | 0.0177 | 0.0042 | 0.0550 | 0.2664 | 0.0000 | 0.0834 | 0.0748 | 0.0000 | 0.0000 | 0.0000 |
| TCGA-AO-A12B-01 | 0.0568 | 0.0000 | 0.0895 | 0.0000 | 0.0000 | 0.0830 | 0.0000 | 0.0497 | 0.0280 | 0.0000 | 0.0433 | 0.0000 | 0.0287 | 0.0502 | 0.0538 | 0.4271 | 0.0000 | 0.0000 | 0.0900 | 0.0000 | 0.0000 | 0.0000 |
| TCGA-AO-A12C-01 | 0.0679 | 0.0000 | 0.0495 | 0.0000 | 0.0000 | 0.0485 | 0.0000 | 0.0251 | 0.0292 | 0.0000 | 0.0174 | 0.0000 | 0.0000 | 0.4366 | 0.0269 | 0.2311 | 0.0000 | 0.0000 | 0.0678 | 0.0000 | 0.0000 | 0.0000 |
| TCGA-AO-A12D-01 | 0.0076 | 0.0000 | 0.0964 | 0.0610 | 0.0000 | 0.2206 | 0.0000 | 0.0880 | 0.0667 | 0.0000 | 0.0128 | 0.0016 | 0.0109 | 0.0883 | 0.0946 | 0.1844 | 0.0000 | 0.0168 | 0.0502 | 0.0000 | 0.0000 | 0.0000 |
| TCGA-AO-A12E-01 | 0.1266 | 0.0000 | 0.0627 | 0.0008 | 0.0000 | 0.0902 | 0.0000 | 0.0314 | 0.0000 | 0.0000 | 0.0127 | 0.0000 | 0.0510 | 0.0225 | 0.0237 | 0.3617 | 0.0000 | 0.0052 | 0.2114 | 0.0000 | 0.0000 | 0.0000 |
| TCGA-AO-A12F-01 | 0.0698 | 0.0000 | 0.0792 | 0.0002 | 0.0000 | 0.0962 | 0.0000 | 0.0819 | 0.0000 | 0.0000 | 0.0308 | 0.0132 | 0.0192 | 0.1057 | 0.0000 | 0.3348 | 0.0000 | 0.0748 | 0.0908 | 0.0000 | 0.0000 | 0.0033 |
| TCGA-AO-A12G-01 | 0.1196 | 0.0000 | 0.0505 | 0.0623 | 0.0000 | 0.1662 | 0.0000 | 0.0353 | 0.0433 | 0.0000 | 0.0296 | 0.0000 | 0.0159 | 0.0000 | 0.0801 | 0.2926 | 0.0008 | 0.0000 | 0.1039 | 0.0000 | 0.0000 | 0.0001 |
| TCGA-AO-A12H-01 | 0.0008 | 0.0040 | 0.0030 | 0.0000 | 0.0000 | 0.2180 | 0.0000 | 0.0695 | 0.0534 | 0.0000 | 0.0688 | 0.0000 | 0.0371 | 0.2373 | 0.0000 | 0.2491 | 0.0000 | 0.0000 | 0.0526 | 0.0000 | 0.0063 | 0.0000 |
| TCGA-AO-A1KO-01 | 0.0968 | 0.0000 | 0.1669 | 0.0364 | 0.0000 | 0.3033 | 0.0000 | 0.0000 | 0.0000 | 0.0000 | 0.0619 | 0.0000 | 0.0273 | 0.0727 | 0.0000 | 0.1487 | 0.0000 | 0.0601 | 0.0259 | 0.0000 | 0.0000 | 0.0000 |
| TCGA-AO-A1KP-01 | 0.0765 | 0.0000 | 0.0078 | 0.0000 | 0.0000 | 0.1858 | 0.0000 | 0.2300 | 0.0000 | 0.0000 | 0.0292 | 0.0054 | 0.0085 | 0.0555 | 0.0897 | 0.1398 | 0.0121 | 0.0700 | 0.0898 | 0.0000 | 0.0000 | 0.0000 |
| TCGA-AO-A1KQ-01 | 0.0373 | 0.0000 | 0.0216 | 0.2022 | 0.0000 | 0.0000 | 0.0000 | 0.1512 | 0.0760 | 0.0000 | 0.0231 | 0.0000 | 0.0164 | 0.2434 | 0.0780 | 0.1254 | 0.0000 | 0.0000 | 0.0253 | 0.0000 | 0.0000 | 0.0000 |
| TCGA-AO-A1KR-01 | 0.0503 | 0.0000 | 0.0589 | 0.1780 | 0.0000 | 0.1509 | 0.0000 | 0.1943 | 0.0032 | 0.0000 | 0.0435 | 0.0601 | 0.0256 | 0.0308 | 0.0716 | 0.0543 | 0.0000 | 0.0324 | 0.0462 | 0.0000 | 0.0000 | 0.0000 |
| TCGA-AO-A1KS-01 | 0.1589 | 0.0000 | 0.0498 | 0.0002 | 0.0000 | 0.2469 | 0.0150 | 0.0616 | 0.0066 | 0.0104 | 0.0000 | 0.0000 | 0.0000 | 0.0848 | 0.0376 | 0.2425 | 0.0000 | 0.0328 | 0.0528 | 0.0000 | 0.0000 | 0.0000 |
| TCGA-AO-A1KT-01 | 0.0338 | 0.0000 | 0.0168 | 0.0080 | 0.0000 | 0.1329 | 0.0000 | 0.0494 | 0.0049 | 0.0000 | 0.0296 | 0.0000 | 0.0000 | 0.2054 | 0.0212 | 0.3882 | 0.0000 | 0.0326 | 0.0705 | 0.0000 | 0.0000 | 0.0068 |
| TCGA-AQ-A04H-01 | 0.0207 | 0.0000 | 0.0011 | 0.0000 | 0.0423 | 0.1417 | 0.0000 | 0.0532 | 0.0730 | 0.0000 | 0.0615 | 0.0000 | 0.0000 | 0.3290 | 0.0580 | 0.1768 | 0.0000 | 0.0000 | 0.0428 | 0.0000 | 0.0000 | 0.0000 |
| TCGA-AQ-A04J-01 | 0.2798 | 0.0000 | 0.0249 | 0.0924 | 0.0007 | 0.0454 | 0.0356 | 0.1207 | 0.0099 | 0.0000 | 0.0065 | 0.0000 | 0.0058 | 0.1143 | 0.0430 | 0.1649 | 0.0000 | 0.0332 | 0.0227 | 0.0000 | 0.0000 | 0.0000 |
| TCGA-AQ-A04L-01 | 0.0952 | 0.0000 | 0.0392 | 0.0012 | 0.0000 | 0.0000 | 0.0000 | 0.1220 | 0.0117 | 0.0000 | 0.0042 | 0.0000 | 0.0000 | 0.3912 | 0.0490 | 0.2633 | 0.0000 | 0.0000 | 0.0231 | 0.0000 | 0.0000 | 0.0000 |
| TCGA-AQ-A0Y5-01 | 0.0009 | 0.0000 | 0.0296 | 0.0000 | 0.0000 | 0.1441 | 0.0000 | 0.0073 | 0.0188 | 0.0000 | 0.0206 | 0.0066 | 0.0000 | 0.3316 | 0.0528 | 0.3242 | 0.0000 | 0.0000 | 0.0633 | 0.0000 | 0.0000 | 0.0002 |
| TCGA-AQ-A1H2-01 | 0.0620 | 0.0000 | 0.0046 | 0.0627 | 0.0000 | 0.0706 | 0.0000 | 0.0507 | 0.0082 | 0.0000 | 0.0226 | 0.0000 | 0.1505 | 0.0000 | 0.0440 | 0.4614 | 0.0000 | 0.0000 | 0.0627 | 0.0000 | 0.0000 | 0.0000 |
| TCGA-AQ-A1H3-01 | 0.1298 | 0.0000 | 0.0199 | 0.0438 | 0.0000 | 0.2712 | 0.0000 | 0.0656 | 0.0120 | 0.0000 | 0.0198 | 0.0000 | 0.0030 | 0.0216 | 0.0811 | 0.1665 | 0.0832 | 0.0212 | 0.0614 | 0.0000 | 0.0000 | 0.0000 |
| TCGA-AQ-A54N-01 | 0.0128 | 0.0000 | 0.1836 | 0.1241 | 0.0000 | 0.0691 | 0.0000 | 0.1654 | 0.0000 | 0.0000 | 0.0016 | 0.0191 | 0.0073 | 0.0000 | 0.0618 | 0.3264 | 0.0000 | 0.0154 | 0.0133 | 0.0000 | 0.0000 | 0.0000 |
| TCGA-AQ-A54O-01 | 0.0545 | 0.0000 | 0.0006 | 0.1125 | 0.0000 | 0.0000 | 0.0000 | 0.1980 | 0.0212 | 0.0000 | 0.0000 | 0.0074 | 0.0000 | 0.3524 | 0.0441 | 0.1962 | 0.0000 | 0.0000 | 0.0133 | 0.0000 | 0.0000 | 0.0000 |
| TCGA-AQ-A7U7-01 | 0.1033 | 0.0000 | 0.0366 | 0.1081 | 0.0000 | 0.2343 | 0.0055 | 0.1332 | 0.0217 | 0.0000 | 0.0094 | 0.0000 | 0.0042 | 0.0314 | 0.1124 | 0.1544 | 0.0000 | 0.0000 | 0.0454 | 0.0000 | 0.0000 | 0.0000 |
| TCGA-AR-A0TP-01 | 0.0000 | 0.0000 | 0.0623 | 0.0000 | 0.0000 | 0.0893 | 0.0000 | 0.0459 | 0.0338 | 0.0000 | 0.0466 | 0.0000 | 0.0000 | 0.6820 | 0.0000 | 0.0340 | 0.0000 | 0.0000 | 0.0060 | 0.0000 | 0.0000 | 0.0000 |
| TCGA-AR-A0TQ-01 | 0.0331 | 0.0000 | 0.0259 | 0.0757 | 0.0000 | 0.1438 | 0.0000 | 0.0478 | 0.0494 | 0.0000 | 0.0539 | 0.0000 | 0.0000 | 0.3116 | 0.0723 | 0.1442 | 0.0000 | 0.0000 | 0.0423 | 0.0000 | 0.0000 | 0.0000 |
| TCGA-AR-A0TR-01 | 0.2546 | 0.0000 | 0.0382 | 0.0965 | 0.0000 | 0.0000 | 0.0000 | 0.1169 | 0.0618 | 0.0000 | 0.0012 | 0.0000 | 0.0004 | 0.0000 | 0.0684 | 0.2684 | 0.0000 | 0.0232 | 0.0704 | 0.0000 | 0.0000 | 0.0000 |
| TCGA-AR-A0TS-01 | 0.2509 | 0.0669 | 0.0000 | 0.0406 | 0.0447 | 0.0876 | 0.0015 | 0.0777 | 0.0467 | 0.0000 | 0.0376 | 0.0000 | 0.0151 | 0.0606 | 0.1195 | 0.1466 | 0.0000 | 0.0000 | 0.0039 | 0.0000 | 0.0000 | 0.0000 |
| TCGA-AR-A0TT-01 | 0.0680 | 0.0000 | 0.0000 | 0.0199 | 0.0000 | 0.1722 | 0.0075 | 0.0717 | 0.0694 | 0.0000 | 0.0460 | 0.0000 | 0.0019 | 0.2245 | 0.0967 | 0.1848 | 0.0000 | 0.0000 | 0.0373 | 0.0000 | 0.0000 | 0.0000 |
| TCGA-AR-A0TU-01 | 0.0031 | 0.0000 | 0.0025 | 0.0656 | 0.0000 | 0.0993 | 0.0399 | 0.1332 | 0.0237 | 0.0000 | 0.0000 | 0.0200 | 0.0000 | 0.1049 | 0.1822 | 0.2019 | 0.0000 | 0.0929 | 0.0307 | 0.0000 | 0.0000 | 0.0000 |
| TCGA-AR-A0TV-01 | 0.0454 | 0.0000 | 0.0140 | 0.0829 | 0.0000 | 0.1313 | 0.0222 | 0.0985 | 0.0216 | 0.0000 | 0.0309 | 0.0000 | 0.0151 | 0.2651 | 0.0824 | 0.1807 | 0.0000 | 0.0000 | 0.0098 | 0.0000 | 0.0000 | 0.0000 |
| TCGA-AR-A0TW-01 | 0.0375 | 0.0000 | 0.0663 | 0.1247 | 0.0000 | 0.1173 | 0.0795 | 0.0807 | 0.0007 | 0.0000 | 0.0000 | 0.0166 | 0.0000 | 0.0411 | 0.1358 | 0.1969 | 0.0000 | 0.0000 | 0.1029 | 0.0000 | 0.0000 | 0.0000 |
| TCGA-AR-A0TX-01 | 0.0668 | 0.0000 | 0.0162 | 0.1044 | 0.0000 | 0.1230 | 0.0250 | 0.1063 | 0.0496 | 0.0000 | 0.0175 | 0.0000 | 0.0000 | 0.0420 | 0.0832 | 0.2795 | 0.0000 | 0.0304 | 0.0561 | 0.0000 | 0.0000 | 0.0000 |
| TCGA-AR-A0TY-01 | 0.0821 | 0.0000 | 0.0304 | 0.0006 | 0.0004 | 0.0249 | 0.0000 | 0.0231 | 0.0064 | 0.0000 | 0.0109 | 0.0000 | 0.0000 | 0.3201 | 0.0191 | 0.3769 | 0.0000 | 0.0000 | 0.1050 | 0.0000 | 0.0000 | 0.0000 |
| TCGA-AR-A0TZ-01 | 0.0683 | 0.0000 | 0.0000 | 0.0396 | 0.0000 | 0.1852 | 0.0000 | 0.0717 | 0.0000 | 0.0000 | 0.0053 | 0.0000 | 0.0020 | 0.0606 | 0.0593 | 0.4331 | 0.0076 | 0.0000 | 0.0486 | 0.0187 | 0.0000 | 0.0000 |
| TCGA-AR-A0U0-01 | 0.0402 | 0.0000 | 0.0568 | 0.0292 | 0.0000 | 0.1543 | 0.0205 | 0.1017 | 0.0153 | 0.0000 | 0.0848 | 0.0000 | 0.0000 | 0.1044 | 0.2128 | 0.1383 | 0.0000 | 0.0000 | 0.0416 | 0.0000 | 0.0000 | 0.0000 |
| TCGA-AR-A0U1-01 | 0.1392 | 0.0000 | 0.1108 | 0.1187 | 0.0000 | 0.0000 | 0.0000 | 0.1269 | 0.0553 | 0.0000 | 0.0088 | 0.0107 | 0.0144 | 0.0179 | 0.1166 | 0.1980 | 0.0357 | 0.0141 | 0.0330 | 0.0000 | 0.0000 | 0.0000 |
| TCGA-AR-A0U2-01 | 0.1077 | 0.0000 | 0.0937 | 0.0652 | 0.0000 | 0.1797 | 0.0142 | 0.0592 | 0.0081 | 0.0000 | 0.0323 | 0.0000 | 0.0000 | 0.1522 | 0.0633 | 0.1927 | 0.0000 | 0.0000 | 0.0318 | 0.0000 | 0.0000 | 0.0000 |
| TCGA-AR-A0U3-01 | 0.0864 | 0.0000 | 0.0000 | 0.0304 | 0.0000 | 0.1002 | 0.0026 | 0.0883 | 0.0309 | 0.0000 | 0.0104 | 0.0000 | 0.0000 | 0.3195 | 0.0632 | 0.2004 | 0.0000 | 0.0080 | 0.0597 | 0.0000 | 0.0000 | 0.0000 |
| TCGA-AR-A0U4-01 | 0.0000 | 0.0057 | 0.0000 | 0.0136 | 0.0000 | 0.0328 | 0.0000 | 0.0533 | 0.0184 | 0.0000 | 0.0231 | 0.0000 | 0.0244 | 0.6113 | 0.1391 | 0.0357 | 0.0000 | 0.0000 | 0.0382 | 0.0000 | 0.0000 | 0.0042 |
| TCGA-AR-A1AH-01 | 0.0299 | 0.0000 | 0.0044 | 0.0077 | 0.0000 | 0.3113 | 0.0000 | 0.2233 | 0.0000 | 0.0000 | 0.0000 | 0.0000 | 0.0482 | 0.3532 | 0.0052 | 0.0000 | 0.0000 | 0.0000 | 0.0000 | 0.0083 | 0.0085 | 0.0000 |
| TCGA-AR-A1AI-01 | 0.0646 | 0.0000 | 0.0655 | 0.0747 | 0.0000 | 0.1495 | 0.0255 | 0.0896 | 0.0204 | 0.0000 | 0.0345 | 0.0042 | 0.0246 | 0.1142 | 0.1060 | 0.1546 | 0.0004 | 0.0285 | 0.0432 | 0.0000 | 0.0000 | 0.0000 |
| TCGA-AR-A1AJ-01 | 0.0565 | 0.0000 | 0.0055 | 0.0000 | 0.0000 | 0.1888 | 0.0052 | 0.1453 | 0.0067 | 0.0000 | 0.0561 | 0.0000 | 0.0041 | 0.1045 | 0.1573 | 0.2254 | 0.0064 | 0.0102 | 0.0280 | 0.0000 | 0.0000 | 0.0000 |
| TCGA-AR-A1AK-01 | 0.0682 | 0.0000 | 0.0075 | 0.0781 | 0.0000 | 0.1498 | 0.0000 | 0.1027 | 0.0344 | 0.0000 | 0.0379 | 0.0000 | 0.0007 | 0.0000 | 0.1033 | 0.3460 | 0.0000 | 0.0000 | 0.0693 | 0.0000 | 0.0000 | 0.0020 |
| TCGA-AR-A1AL-01 | 0.0894 | 0.0000 | 0.0036 | 0.0820 | 0.0000 | 0.2382 | 0.0040 | 0.0000 | 0.0000 | 0.0000 | 0.0383 | 0.0000 | 0.0202 | 0.0000 | 0.0319 | 0.2810 | 0.0743 | 0.0103 | 0.1252 | 0.0000 | 0.0000 | 0.0015 |
| TCGA-AR-A1AM-01 | 0.1158 | 0.0000 | 0.0759 | 0.0910 | 0.0000 | 0.1113 | 0.0000 | 0.0524 | 0.0317 | 0.0000 | 0.0232 | 0.0000 | 0.0067 | 0.1228 | 0.0425 | 0.2471 | 0.0010 | 0.0000 | 0.0786 | 0.0000 | 0.0000 | 0.0000 |
| TCGA-AR-A1AN-01 | 0.0061 | 0.0123 | 0.0097 | 0.0000 | 0.0000 | 0.0000 | 0.0000 | 0.0922 | 0.0045 | 0.0239 | 0.0282 | 0.0000 | 0.0000 | 0.5555 | 0.0000 | 0.1696 | 0.0030 | 0.0000 | 0.0950 | 0.0000 | 0.0000 | 0.0000 |
| TCGA-AR-A1AO-01 | 0.0087 | 0.0151 | 0.0000 | 0.0071 | 0.0000 | 0.0584 | 0.0000 | 0.0362 | 0.0403 | 0.0000 | 0.0093 | 0.0000 | 0.0000 | 0.6028 | 0.0351 | 0.1359 | 0.0000 | 0.0000 | 0.0511 | 0.0000 | 0.0000 | 0.0000 |
| TCGA-AR-A1AP-01 | 0.0453 | 0.0000 | 0.0043 | 0.0000 | 0.0000 | 0.1085 | 0.0000 | 0.0800 | 0.0675 | 0.0000 | 0.0760 | 0.0000 | 0.0186 | 0.1271 | 0.0993 | 0.2648 | 0.0266 | 0.0000 | 0.0816 | 0.0000 | 0.0000 | 0.0005 |
| TCGA-AR-A1AQ-01 | 0.0381 | 0.0000 | 0.1176 | 0.0343 | 0.0000 | 0.1734 | 0.0350 | 0.1226 | 0.0222 | 0.0000 | 0.0372 | 0.0000 | 0.0018 | 0.1663 | 0.0815 | 0.1418 | 0.0060 | 0.0028 | 0.0193 | 0.0000 | 0.0000 | 0.0000 |
| TCGA-AR-A1AR-01 | 0.0302 | 0.0000 | 0.0000 | 0.0000 | 0.0000 | 0.1680 | 0.0100 | 0.0411 | 0.0179 | 0.0004 | 0.0539 | 0.0000 | 0.0000 | 0.3310 | 0.1212 | 0.2122 | 0.0000 | 0.0000 | 0.0142 | 0.0000 | 0.0000 | 0.0000 |
| TCGA-AR-A1AS-01 | 0.0000 | 0.0092 | 0.0010 | 0.0000 | 0.0821 | 0.0980 | 0.0000 | 0.0189 | 0.0642 | 0.0000 | 0.0747 | 0.0000 | 0.0000 | 0.4020 | 0.0000 | 0.2456 | 0.0000 | 0.0000 | 0.0042 | 0.0000 | 0.0000 | 0.0000 |
| TCGA-AR-A1AT-01 | 0.0595 | 0.0000 | 0.0148 | 0.0902 | 0.0000 | 0.1558 | 0.0000 | 0.0970 | 0.0456 | 0.0000 | 0.0249 | 0.0000 | 0.0042 | 0.1492 | 0.0891 | 0.2416 | 0.0000 | 0.0000 | 0.0283 | 0.0000 | 0.0000 | 0.0000 |
| TCGA-AR-A1AU-01 | 0.0433 | 0.0000 | 0.0858 | 0.0778 | 0.0000 | 0.1495 | 0.0000 | 0.0405 | 0.0000 | 0.0000 | 0.0027 | 0.0309 | 0.0425 | 0.0896 | 0.0489 | 0.2132 | 0.0000 | 0.0097 | 0.1656 | 0.0000 | 0.0000 | 0.0000 |
| TCGA-AR-A1AV-01 | 0.0004 | 0.0000 | 0.0323 | 0.0000 | 0.0000 | 0.2004 | 0.0000 | 0.0731 | 0.0476 | 0.0000 | 0.0584 | 0.0000 | 0.0195 | 0.3826 | 0.0000 | 0.1219 | 0.0000 | 0.0000 | 0.0637 | 0.0000 | 0.0000 | 0.0000 |
| TCGA-AR-A1AW-01 | 0.1115 | 0.0506 | 0.0448 | 0.0824 | 0.0000 | 0.0888 | 0.0278 | 0.0908 | 0.0266 | 0.0097 | 0.0357 | 0.0000 | 0.0000 | 0.1790 | 0.1038 | 0.1345 | 0.0000 | 0.0000 | 0.0141 | 0.0000 | 0.0000 | 0.0000 |
| TCGA-AR-A1AX-01 | 0.1267 | 0.0315 | 0.0000 | 0.0867 | 0.0000 | 0.1564 | 0.0035 | 0.1054 | 0.0316 | 0.0000 | 0.0164 | 0.0000 | 0.0108 | 0.0445 | 0.1063 | 0.1221 | 0.0000 | 0.0000 | 0.1581 | 0.0000 | 0.0000 | 0.0000 |
| TCGA-AR-A1AY-01 | 0.0002 | 0.0032 | 0.0112 | 0.0000 | 0.0000 | 0.1838 | 0.0000 | 0.0204 | 0.0420 | 0.0000 | 0.0430 | 0.0000 | 0.0000 | 0.5780 | 0.0135 | 0.0880 | 0.0000 | 0.0000 | 0.0168 | 0.0000 | 0.0000 | 0.0000 |
| TCGA-AR-A24H-01 | 0.1212 | 0.0000 | 0.0163 | 0.0897 | 0.0000 | 0.1076 | 0.0000 | 0.1190 | 0.0413 | 0.0000 | 0.0049 | 0.0000 | 0.0000 | 0.1519 | 0.0517 | 0.2629 | 0.0000 | 0.0000 | 0.0335 | 0.0000 | 0.0000 | 0.0000 |
| TCGA-AR-A24K-01 | 0.1500 | 0.0000 | 0.0812 | 0.0372 | 0.0000 | 0.1048 | 0.0000 | 0.0438 | 0.0017 | 0.0000 | 0.0165 | 0.0000 | 0.0101 | 0.0227 | 0.0391 | 0.4153 | 0.0067 | 0.0517 | 0.0177 | 0.0000 | 0.0000 | 0.0015 |
| TCGA-AR-A24L-01 | 0.1016 | 0.0000 | 0.0616 | 0.0000 | 0.0000 | 0.1071 | 0.0000 | 0.0168 | 0.0160 | 0.0001 | 0.0000 | 0.0162 | 0.0000 | 0.2434 | 0.0753 | 0.2853 | 0.0000 | 0.0000 | 0.0767 | 0.0000 | 0.0000 | 0.0000 |
| TCGA-AR-A24M-01 | 0.0760 | 0.0000 | 0.0381 | 0.0000 | 0.0000 | 0.0812 | 0.0000 | 0.0487 | 0.0077 | 0.0000 | 0.0207 | 0.0000 | 0.0000 | 0.1071 | 0.0179 | 0.4312 | 0.0000 | 0.0000 | 0.1588 | 0.0000 | 0.0000 | 0.0126 |
| TCGA-AR-A24N-01 | 0.0444 | 0.0000 | 0.1467 | 0.0000 | 0.0000 | 0.1779 | 0.0000 | 0.1081 | 0.0030 | 0.0000 | 0.0671 | 0.0000 | 0.0096 | 0.0466 | 0.0502 | 0.2614 | 0.0000 | 0.0290 | 0.0559 | 0.0000 | 0.0000 | 0.0000 |
| TCGA-AR-A24O-01 | 0.0954 | 0.0000 | 0.0273 | 0.0456 | 0.0000 | 0.2286 | 0.0000 | 0.0487 | 0.0007 | 0.0000 | 0.0183 | 0.0000 | 0.0104 | 0.0000 | 0.0788 | 0.3102 | 0.0007 | 0.0098 | 0.1256 | 0.0000 | 0.0000 | 0.0000 |
| TCGA-AR-A24P-01 | 0.0522 | 0.0000 | 0.0393 | 0.0000 | 0.0000 | 0.1764 | 0.0000 | 0.0787 | 0.0000 | 0.0000 | 0.0700 | 0.0000 | 0.0023 | 0.0733 | 0.0577 | 0.3957 | 0.0004 | 0.0130 | 0.0410 | 0.0000 | 0.0000 | 0.0000 |
| TCGA-AR-A24Q-01 | 0.0330 | 0.0000 | 0.0276 | 0.0259 | 0.0000 | 0.0412 | 0.0000 | 0.0790 | 0.0460 | 0.0082 | 0.0000 | 0.0106 | 0.0000 | 0.4191 | 0.0610 | 0.1728 | 0.0000 | 0.0000 | 0.0756 | 0.0000 | 0.0000 | 0.0000 |
| TCGA-AR-A24R-01 | 0.0665 | 0.0000 | 0.0210 | 0.0594 | 0.0000 | 0.1422 | 0.0000 | 0.0894 | 0.0451 | 0.0000 | 0.0168 | 0.0073 | 0.0159 | 0.0887 | 0.0811 | 0.2642 | 0.0118 | 0.0130 | 0.0777 | 0.0000 | 0.0000 | 0.0000 |
| TCGA-AR-A24S-01 | 0.0553 | 0.0000 | 0.0000 | 0.0000 | 0.0000 | 0.1383 | 0.0000 | 0.0270 | 0.0185 | 0.0000 | 0.0253 | 0.0000 | 0.0004 | 0.2334 | 0.0677 | 0.2697 | 0.0934 | 0.0000 | 0.0710 | 0.0000 | 0.0000 | 0.0000 |
| TCGA-AR-A24T-01 | 0.1366 | 0.0000 | 0.0218 | 0.0271 | 0.0000 | 0.1225 | 0.0000 | 0.0207 | 0.0110 | 0.0000 | 0.0205 | 0.0000 | 0.0000 | 0.0000 | 0.0489 | 0.4493 | 0.0000 | 0.0000 | 0.1417 | 0.0000 | 0.0000 | 0.0000 |
| TCGA-AR-A24U-01 | 0.0586 | 0.0171 | 0.0185 | 0.0270 | 0.0000 | 0.2638 | 0.0246 | 0.0484 | 0.0051 | 0.0000 | 0.0171 | 0.0000 | 0.0037 | 0.1363 | 0.0890 | 0.2082 | 0.0154 | 0.0099 | 0.0571 | 0.0000 | 0.0000 | 0.0000 |
| TCGA-AR-A24V-01 | 0.0018 | 0.0000 | 0.0107 | 0.0000 | 0.0000 | 0.1835 | 0.0000 | 0.0288 | 0.0690 | 0.0000 | 0.0521 | 0.0000 | 0.0000 | 0.2473 | 0.0368 | 0.2952 | 0.0000 | 0.0000 | 0.0474 | 0.0273 | 0.0000 | 0.0000 |
| TCGA-AR-A24W-01 | 0.0903 | 0.0000 | 0.1452 | 0.0180 | 0.0000 | 0.2550 | 0.0000 | 0.0030 | 0.0000 | 0.0000 | 0.0000 | 0.0048 | 0.0120 | 0.0000 | 0.0000 | 0.2397 | 0.0000 | 0.0419 | 0.1901 | 0.0000 | 0.0000 | 0.0000 |
| TCGA-AR-A24X-01 | 0.1126 | 0.0000 | 0.0139 | 0.0896 | 0.0000 | 0.2502 | 0.0000 | 0.2564 | 0.0000 | 0.0000 | 0.0627 | 0.0000 | 0.0667 | 0.0354 | 0.0601 | 0.0450 | 0.0004 | 0.0000 | 0.0069 | 0.0000 | 0.0000 | 0.0000 |
| TCGA-AR-A24Z-01 | 0.0027 | 0.0066 | 0.0000 | 0.0000 | 0.0000 | 0.2090 | 0.0000 | 0.0493 | 0.0682 | 0.0000 | 0.0620 | 0.0000 | 0.0000 | 0.3253 | 0.0049 | 0.2640 | 0.0000 | 0.0038 | 0.0042 | 0.0000 | 0.0000 | 0.0000 |
| TCGA-AR-A250-01 | 0.0240 | 0.0000 | 0.0266 | 0.0000 | 0.0000 | 0.1738 | 0.0000 | 0.0543 | 0.0346 | 0.0000 | 0.0108 | 0.0000 | 0.0000 | 0.3192 | 0.0521 | 0.2357 | 0.0000 | 0.0000 | 0.0690 | 0.0000 | 0.0000 | 0.0000 |
| TCGA-AR-A251-01 | 0.1574 | 0.0000 | 0.0603 | 0.0416 | 0.0000 | 0.1631 | 0.0099 | 0.0450 | 0.0000 | 0.0000 | 0.0191 | 0.0000 | 0.0125 | 0.0936 | 0.1733 | 0.1980 | 0.0000 | 0.0018 | 0.0245 | 0.0000 | 0.0000 | 0.0000 |
| TCGA-AR-A252-01 | 0.0478 | 0.0000 | 0.0232 | 0.1506 | 0.0000 | 0.2259 | 0.0329 | 0.0416 | 0.0372 | 0.0000 | 0.0199 | 0.0000 | 0.0322 | 0.0202 | 0.0871 | 0.2063 | 0.0171 | 0.0000 | 0.0579 | 0.0000 | 0.0000 | 0.0000 |
| TCGA-AR-A254-01 | 0.0059 | 0.0000 | 0.0429 | 0.0358 | 0.0000 | 0.0728 | 0.0054 | 0.0845 | 0.0231 | 0.0000 | 0.0000 | 0.0118 | 0.0085 | 0.1985 | 0.1057 | 0.2654 | 0.0009 | 0.0000 | 0.1388 | 0.0000 | 0.0000 | 0.0000 |
| TCGA-AR-A255-01 | 0.0550 | 0.0000 | 0.0088 | 0.0657 | 0.0000 | 0.0414 | 0.0000 | 0.0780 | 0.0427 | 0.0000 | 0.0187 | 0.0000 | 0.0043 | 0.1178 | 0.1198 | 0.2756 | 0.0077 | 0.0290 | 0.1356 | 0.0000 | 0.0000 | 0.0000 |
| TCGA-AR-A256-01 | 0.0216 | 0.0000 | 0.0000 | 0.0000 | 0.0000 | 0.1123 | 0.0000 | 0.0817 | 0.0193 | 0.0200 | 0.0000 | 0.0000 | 0.0000 | 0.4109 | 0.0318 | 0.1348 | 0.0000 | 0.1476 | 0.0201 | 0.0000 | 0.0000 | 0.0000 |
| TCGA-AR-A2LE-01 | 0.1403 | 0.0000 | 0.0356 | 0.0554 | 0.0000 | 0.1749 | 0.0000 | 0.0121 | 0.0024 | 0.0000 | 0.0135 | 0.0000 | 0.0018 | 0.0523 | 0.0506 | 0.2991 | 0.0577 | 0.0283 | 0.0760 | 0.0000 | 0.0000 | 0.0000 |
| TCGA-AR-A2LH-01 | 0.0749 | 0.0035 | 0.0238 | 0.0918 | 0.0000 | 0.2727 | 0.0000 | 0.0574 | 0.0305 | 0.0000 | 0.0417 | 0.0000 | 0.0424 | 0.0000 | 0.0904 | 0.1993 | 0.0020 | 0.0189 | 0.0509 | 0.0000 | 0.0000 | 0.0000 |
| TCGA-AR-A2LJ-01 | 0.0011 | 0.0045 | 0.0036 | 0.0635 | 0.0000 | 0.1404 | 0.0000 | 0.0082 | 0.0427 | 0.0000 | 0.0028 | 0.0055 | 0.0086 | 0.4132 | 0.0320 | 0.2254 | 0.0000 | 0.0000 | 0.0484 | 0.0000 | 0.0000 | 0.0000 |
| TCGA-AR-A2LK-01 | 0.0000 | 0.0058 | 0.0180 | 0.0000 | 0.0000 | 0.1920 | 0.0000 | 0.0246 | 0.0537 | 0.0000 | 0.0117 | 0.0316 | 0.0420 | 0.2258 | 0.0066 | 0.1720 | 0.0064 | 0.0000 | 0.2097 | 0.0000 | 0.0000 | 0.0000 |
| TCGA-AR-A2LL-01 | 0.0108 | 0.0013 | 0.0177 | 0.0000 | 0.0364 | 0.0787 | 0.0000 | 0.0276 | 0.0330 | 0.0000 | 0.0818 | 0.0000 | 0.0185 | 0.3146 | 0.0000 | 0.2443 | 0.0088 | 0.0000 | 0.1265 | 0.0000 | 0.0000 | 0.0000 |
| TCGA-AR-A2LM-01 | 0.1029 | 0.0000 | 0.0030 | 0.0885 | 0.0000 | 0.2212 | 0.0000 | 0.0841 | 0.0000 | 0.0000 | 0.0000 | 0.0000 | 0.0384 | 0.0000 | 0.0446 | 0.2455 | 0.0044 | 0.0023 | 0.1651 | 0.0000 | 0.0000 | 0.0000 |
| TCGA-AR-A2LN-01 | 0.0082 | 0.0000 | 0.0615 | 0.1670 | 0.0000 | 0.1395 | 0.0000 | 0.0714 | 0.0065 | 0.0000 | 0.0289 | 0.0000 | 0.0578 | 0.0032 | 0.0180 | 0.1772 | 0.0000 | 0.0446 | 0.2163 | 0.0000 | 0.0000 | 0.0000 |
| TCGA-AR-A2LO-01 | 0.1872 | 0.0000 | 0.0111 | 0.1489 | 0.0000 | 0.1448 | 0.0000 | 0.1564 | 0.0162 | 0.0000 | 0.0034 | 0.0000 | 0.0119 | 0.0000 | 0.0730 | 0.2198 | 0.0000 | 0.0099 | 0.0174 | 0.0000 | 0.0000 | 0.0000 |
| TCGA-AR-A2LQ-01 | 0.0000 | 0.0000 | 0.0764 | 0.0000 | 0.0000 | 0.1921 | 0.0000 | 0.0310 | 0.0287 | 0.0000 | 0.0148 | 0.0029 | 0.0194 | 0.3164 | 0.0000 | 0.2705 | 0.0000 | 0.0000 | 0.0477 | 0.0000 | 0.0000 | 0.0000 |
| TCGA-AR-A2LR-01 | 0.0055 | 0.0000 | 0.0065 | 0.0000 | 0.0000 | 0.3263 | 0.0000 | 0.1424 | 0.0058 | 0.0000 | 0.0019 | 0.0110 | 0.0606 | 0.1188 | 0.0157 | 0.2569 | 0.0000 | 0.0000 | 0.0451 | 0.0000 | 0.0032 | 0.0000 |
| TCGA-AR-A5QM-01 | 0.0788 | 0.0000 | 0.0381 | 0.0231 | 0.0000 | 0.1321 | 0.0000 | 0.0425 | 0.0000 | 0.0000 | 0.0109 | 0.0000 | 0.0000 | 0.3975 | 0.0223 | 0.1034 | 0.0000 | 0.0132 | 0.1382 | 0.0000 | 0.0000 | 0.0000 |
| TCGA-AR-A5QN-01 | 0.1289 | 0.0000 | 0.0216 | 0.0660 | 0.0000 | 0.2377 | 0.0000 | 0.0340 | 0.0160 | 0.0000 | 0.0184 | 0.0000 | 0.0052 | 0.0000 | 0.0959 | 0.2766 | 0.0000 | 0.0000 | 0.0996 | 0.0000 | 0.0000 | 0.0000 |
| TCGA-AR-A5QP-01 | 0.0812 | 0.0000 | 0.1713 | 0.0714 | 0.0000 | 0.1197 | 0.0000 | 0.0595 | 0.0000 | 0.0000 | 0.0188 | 0.0000 | 0.0074 | 0.0923 | 0.0360 | 0.2941 | 0.0000 | 0.0000 | 0.0483 | 0.0000 | 0.0000 | 0.0000 |
| TCGA-AR-A5QQ-01 | 0.0378 | 0.0226 | 0.0000 | 0.0117 | 0.0000 | 0.1033 | 0.0000 | 0.1112 | 0.0552 | 0.0000 | 0.0218 | 0.0123 | 0.0000 | 0.2542 | 0.0848 | 0.2733 | 0.0022 | 0.0000 | 0.0094 | 0.0000 | 0.0000 | 0.0000 |
| TCGA-B6-A0I1-01 | 0.0830 | 0.0000 | 0.0974 | 0.0064 | 0.0023 | 0.0000 | 0.0000 | 0.0787 | 0.0017 | 0.0000 | 0.0254 | 0.0000 | 0.0000 | 0.5842 | 0.0355 | 0.0449 | 0.0000 | 0.0174 | 0.0230 | 0.0000 | 0.0000 | 0.0000 |
| TCGA-B6-A0I2-01 | 0.0412 | 0.0000 | 0.0068 | 0.1500 | 0.0000 | 0.0596 | 0.0060 | 0.1042 | 0.0567 | 0.0000 | 0.0543 | 0.0389 | 0.0079 | 0.1464 | 0.2255 | 0.0391 | 0.0004 | 0.0000 | 0.0631 | 0.0000 | 0.0000 | 0.0000 |
| TCGA-B6-A0I5-01 | 0.0253 | 0.0160 | 0.0000 | 0.0733 | 0.0000 | 0.0042 | 0.0000 | 0.1840 | 0.0953 | 0.0000 | 0.0543 | 0.0000 | 0.0000 | 0.3500 | 0.0350 | 0.0702 | 0.0000 | 0.0000 | 0.0897 | 0.0000 | 0.0026 | 0.0000 |
| TCGA-B6-A0I6-01 | 0.0137 | 0.0000 | 0.0288 | 0.0000 | 0.0000 | 0.2715 | 0.0000 | 0.0961 | 0.0849 | 0.0000 | 0.0246 | 0.0000 | 0.0154 | 0.3018 | 0.0107 | 0.1030 | 0.0000 | 0.0000 | 0.0496 | 0.0000 | 0.0000 | 0.0000 |
| TCGA-B6-A0I8-01 | 0.1661 | 0.0000 | 0.1051 | 0.0246 | 0.0000 | 0.0003 | 0.0000 | 0.0161 | 0.0208 | 0.0000 | 0.0071 | 0.0002 | 0.0000 | 0.0833 | 0.0266 | 0.4517 | 0.0000 | 0.0203 | 0.0647 | 0.0000 | 0.0000 | 0.0130 |
| TCGA-B6-A0I9-01 | 0.1019 | 0.0000 | 0.0722 | 0.0295 | 0.0000 | 0.1403 | 0.0000 | 0.0503 | 0.0337 | 0.0000 | 0.0238 | 0.0000 | 0.0081 | 0.1982 | 0.0554 | 0.1945 | 0.0000 | 0.0659 | 0.0263 | 0.0000 | 0.0000 | 0.0000 |
| TCGA-B6-A0IA-01 | 0.0298 | 0.0000 | 0.0011 | 0.0000 | 0.0371 | 0.0000 | 0.0000 | 0.1848 | 0.0449 | 0.0000 | 0.0569 | 0.0000 | 0.0000 | 0.4797 | 0.0000 | 0.0708 | 0.0000 | 0.0756 | 0.0193 | 0.0000 | 0.0000 | 0.0000 |
| TCGA-B6-A0IB-01 | 0.0786 | 0.0000 | 0.0000 | 0.0757 | 0.0000 | 0.1366 | 0.0000 | 0.0010 | 0.0567 | 0.0000 | 0.0000 | 0.0311 | 0.0000 | 0.3478 | 0.0512 | 0.1544 | 0.0000 | 0.0000 | 0.0670 | 0.0000 | 0.0000 | 0.0000 |
| TCGA-B6-A0IC-01 | 0.0000 | 0.0000 | 0.0643 | 0.0000 | 0.0000 | 0.4237 | 0.0000 | 0.0751 | 0.0659 | 0.0000 | 0.0489 | 0.0249 | 0.0457 | 0.1508 | 0.0000 | 0.0647 | 0.0089 | 0.0000 | 0.0272 | 0.0000 | 0.0000 | 0.0000 |
| TCGA-B6-A0IE-01 | 0.0451 | 0.0000 | 0.4369 | 0.0752 | 0.0000 | 0.0607 | 0.0000 | 0.0123 | 0.0511 | 0.0000 | 0.0041 | 0.0047 | 0.0123 | 0.0000 | 0.0537 | 0.1925 | 0.0000 | 0.0000 | 0.0513 | 0.0000 | 0.0000 | 0.0000 |
| TCGA-B6-A0IG-01 | 0.0886 | 0.0000 | 0.0598 | 0.0442 | 0.0000 | 0.1409 | 0.0000 | 0.0305 | 0.0365 | 0.0000 | 0.0349 | 0.0000 | 0.0000 | 0.3451 | 0.0990 | 0.0838 | 0.0000 | 0.0103 | 0.0263 | 0.0000 | 0.0000 | 0.0000 |
| TCGA-B6-A0IH-01 | 0.1269 | 0.0000 | 0.1295 | 0.1016 | 0.0000 | 0.1663 | 0.0000 | 0.0747 | 0.0230 | 0.0000 | 0.0000 | 0.0181 | 0.0103 | 0.0000 | 0.0820 | 0.1530 | 0.0246 | 0.0078 | 0.0822 | 0.0000 | 0.0000 | 0.0000 |
| TCGA-B6-A0IJ-01 | 0.0043 | 0.0224 | 0.0276 | 0.0752 | 0.0000 | 0.1052 | 0.0000 | 0.0694 | 0.0662 | 0.0000 | 0.0074 | 0.0000 | 0.0150 | 0.2860 | 0.1251 | 0.1650 | 0.0011 | 0.0000 | 0.0301 | 0.0000 | 0.0000 | 0.0000 |
| TCGA-B6-A0IK-01 | 0.1036 | 0.0000 | 0.0882 | 0.0537 | 0.0000 | 0.1567 | 0.0000 | 0.0946 | 0.0427 | 0.0000 | 0.0014 | 0.0042 | 0.0049 | 0.0510 | 0.0625 | 0.2521 | 0.0000 | 0.0498 | 0.0347 | 0.0000 | 0.0000 | 0.0000 |
| TCGA-B6-A0IM-01 | 0.0021 | 0.0041 | 0.0090 | 0.0000 | 0.0000 | 0.2276 | 0.0000 | 0.0279 | 0.0344 | 0.0000 | 0.0577 | 0.0130 | 0.0000 | 0.4470 | 0.0151 | 0.0719 | 0.0086 | 0.0000 | 0.0815 | 0.0000 | 0.0000 | 0.0000 |
| TCGA-B6-A0IN-01 | 0.1394 | 0.0000 | 0.0400 | 0.0380 | 0.0000 | 0.2044 | 0.0000 | 0.0725 | 0.0420 | 0.0000 | 0.0210 | 0.0000 | 0.0105 | 0.1128 | 0.0476 | 0.1879 | 0.0569 | 0.0000 | 0.0270 | 0.0000 | 0.0000 | 0.0000 |
| TCGA-B6-A0IO-01 | 0.0495 | 0.0000 | 0.0137 | 0.0453 | 0.0000 | 0.1700 | 0.0000 | 0.0976 | 0.0580 | 0.0000 | 0.0319 | 0.0041 | 0.0230 | 0.1725 | 0.1033 | 0.1847 | 0.0000 | 0.0000 | 0.0464 | 0.0000 | 0.0000 | 0.0000 |
| TCGA-B6-A0IP-01 | 0.0334 | 0.1768 | 0.0000 | 0.1284 | 0.0000 | 0.0000 | 0.0000 | 0.1222 | 0.0657 | 0.0000 | 0.0623 | 0.0000 | 0.0278 | 0.1599 | 0.0801 | 0.0028 | 0.0090 | 0.0000 | 0.1297 | 0.0000 | 0.0020 | 0.0000 |
| TCGA-B6-A0IQ-01 | 0.0653 | 0.0000 | 0.0462 | 0.0465 | 0.0000 | 0.0412 | 0.0000 | 0.2299 | 0.0275 | 0.0000 | 0.0177 | 0.0135 | 0.0000 | 0.2364 | 0.1609 | 0.0679 | 0.0121 | 0.0000 | 0.0349 | 0.0000 | 0.0000 | 0.0000 |
| TCGA-B6-A0RE-01 | 0.0000 | 0.0085 | 0.0436 | 0.0384 | 0.0000 | 0.2145 | 0.0000 | 0.1922 | 0.0014 | 0.0000 | 0.0000 | 0.0000 | 0.0220 | 0.3940 | 0.0000 | 0.0000 | 0.0000 | 0.0331 | 0.0494 | 0.0000 | 0.0029 | 0.0000 |
| TCGA-B6-A0RG-01 | 0.0835 | 0.0000 | 0.0493 | 0.0335 | 0.0000 | 0.0479 | 0.0000 | 0.0197 | 0.0375 | 0.0000 | 0.0000 | 0.0274 | 0.0000 | 0.3607 | 0.0859 | 0.1621 | 0.0081 | 0.0000 | 0.0845 | 0.0000 | 0.0000 | 0.0000 |
| TCGA-B6-A0RH-01 | 0.0923 | 0.0000 | 0.0941 | 0.0782 | 0.0000 | 0.0533 | 0.0000 | 0.0707 | 0.0343 | 0.0000 | 0.0175 | 0.0154 | 0.0000 | 0.2179 | 0.0889 | 0.1357 | 0.0000 | 0.0401 | 0.0617 | 0.0000 | 0.0000 | 0.0000 |
| TCGA-B6-A0RI-01 | 0.0663 | 0.0000 | 0.0137 | 0.0040 | 0.0000 | 0.0991 | 0.0000 | 0.0277 | 0.0269 | 0.0000 | 0.0099 | 0.0000 | 0.0000 | 0.3223 | 0.0453 | 0.2871 | 0.0000 | 0.0000 | 0.0979 | 0.0000 | 0.0000 | 0.0000 |
| TCGA-B6-A0RL-01 | 0.0000 | 0.0160 | 0.0000 | 0.0000 | 0.0000 | 0.2487 | 0.0000 | 0.1988 | 0.0772 | 0.0000 | 0.0322 | 0.0165 | 0.0317 | 0.2429 | 0.0114 | 0.0798 | 0.0000 | 0.0000 | 0.0415 | 0.0000 | 0.0035 | 0.0000 |
| TCGA-B6-A0RM-01 | 0.0726 | 0.0000 | 0.0056 | 0.0505 | 0.0000 | 0.1844 | 0.0000 | 0.0488 | 0.0058 | 0.0000 | 0.0000 | 0.0073 | 0.0000 | 0.0000 | 0.0403 | 0.2610 | 0.0211 | 0.0000 | 0.3026 | 0.0000 | 0.0000 | 0.0000 |
| TCGA-B6-A0RN-01 | 0.1177 | 0.0000 | 0.0818 | 0.0253 | 0.0000 | 0.2111 | 0.0000 | 0.1079 | 0.0143 | 0.0000 | 0.0198 | 0.0054 | 0.0129 | 0.0000 | 0.0438 | 0.2407 | 0.0163 | 0.0000 | 0.1028 | 0.0000 | 0.0000 | 0.0000 |
| TCGA-B6-A0RO-01 | 0.1634 | 0.0000 | 0.0714 | 0.0339 | 0.0000 | 0.1212 | 0.0000 | 0.0422 | 0.0000 | 0.0000 | 0.0000 | 0.0171 | 0.0409 | 0.0000 | 0.0402 | 0.3874 | 0.0000 | 0.0513 | 0.0305 | 0.0000 | 0.0000 | 0.0003 |
| TCGA-B6-A0RP-01 | 0.0952 | 0.0000 | 0.0077 | 0.0956 | 0.0000 | 0.2420 | 0.0000 | 0.0416 | 0.0143 | 0.0000 | 0.0553 | 0.0000 | 0.0067 | 0.0613 | 0.0548 | 0.3070 | 0.0000 | 0.0000 | 0.0163 | 0.0024 | 0.0000 | 0.0000 |
| TCGA-B6-A0RQ-01 | 0.0039 | 0.0000 | 0.0183 | 0.0344 | 0.0000 | 0.2166 | 0.0000 | 0.1539 | 0.0016 | 0.0000 | 0.0000 | 0.0331 | 0.0428 | 0.1190 | 0.0202 | 0.0700 | 0.0082 | 0.0000 | 0.2780 | 0.0000 | 0.0000 | 0.0000 |
| TCGA-B6-A0RS-01 | 0.0828 | 0.0000 | 0.0244 | 0.0758 | 0.0000 | 0.2153 | 0.0074 | 0.0781 | 0.0092 | 0.0000 | 0.0207 | 0.0000 | 0.0000 | 0.0953 | 0.0589 | 0.2923 | 0.0000 | 0.0293 | 0.0104 | 0.0000 | 0.0000 | 0.0000 |
| TCGA-B6-A0RT-01 | 0.0706 | 0.0211 | 0.0000 | 0.2183 | 0.0000 | 0.0496 | 0.0143 | 0.1216 | 0.1092 | 0.0000 | 0.0361 | 0.0000 | 0.0189 | 0.0586 | 0.1641 | 0.0810 | 0.0000 | 0.0000 | 0.0365 | 0.0000 | 0.0000 | 0.0000 |
| TCGA-B6-A0RU-01 | 0.1207 | 0.0000 | 0.0430 | 0.0000 | 0.0000 | 0.1258 | 0.0000 | 0.0786 | 0.0489 | 0.0000 | 0.0000 | 0.0304 | 0.0045 | 0.1811 | 0.0606 | 0.2580 | 0.0000 | 0.0108 | 0.0377 | 0.0000 | 0.0000 | 0.0000 |
| TCGA-B6-A0RV-01 | 0.0593 | 0.0000 | 0.0432 | 0.1285 | 0.0000 | 0.1247 | 0.0000 | 0.0623 | 0.0678 | 0.0000 | 0.0202 | 0.0000 | 0.0000 | 0.1565 | 0.0683 | 0.2164 | 0.0000 | 0.0000 | 0.0528 | 0.0000 | 0.0000 | 0.0000 |
| TCGA-B6-A0WS-01 | 0.1077 | 0.0000 | 0.0264 | 0.0732 | 0.0000 | 0.1397 | 0.0000 | 0.0811 | 0.0653 | 0.0000 | 0.0209 | 0.0000 | 0.0000 | 0.2150 | 0.0888 | 0.1288 | 0.0000 | 0.0063 | 0.0467 | 0.0000 | 0.0000 | 0.0000 |
| TCGA-B6-A0WT-01 | 0.1233 | 0.0000 | 0.1144 | 0.0311 | 0.0000 | 0.0602 | 0.0000 | 0.0478 | 0.0346 | 0.0000 | 0.0000 | 0.0062 | 0.0000 | 0.1571 | 0.0313 | 0.2088 | 0.0000 | 0.0509 | 0.1344 | 0.0000 | 0.0000 | 0.0000 |
| TCGA-B6-A0WV-01 | 0.1671 | 0.0000 | 0.0935 | 0.0250 | 0.0000 | 0.1044 | 0.0000 | 0.0430 | 0.0506 | 0.0000 | 0.0000 | 0.0036 | 0.0000 | 0.1125 | 0.0403 | 0.2780 | 0.0000 | 0.0464 | 0.0356 | 0.0000 | 0.0000 | 0.0000 |
| TCGA-B6-A0WW-01 | 0.0911 | 0.0000 | 0.0399 | 0.0096 | 0.0000 | 0.1451 | 0.0000 | 0.0826 | 0.0347 | 0.0000 | 0.0309 | 0.0000 | 0.0000 | 0.1248 | 0.0961 | 0.2760 | 0.0000 | 0.0000 | 0.0692 | 0.0000 | 0.0000 | 0.0000 |
| TCGA-B6-A0WX-01 | 0.1963 | 0.0000 | 0.0639 | 0.0684 | 0.0000 | 0.1780 | 0.0000 | 0.1210 | 0.0381 | 0.0000 | 0.0114 | 0.0000 | 0.0071 | 0.0320 | 0.1096 | 0.1591 | 0.0000 | 0.0000 | 0.0150 | 0.0000 | 0.0000 | 0.0000 |
| TCGA-B6-A0WY-01 | 0.0962 | 0.0000 | 0.0320 | 0.0396 | 0.0000 | 0.0957 | 0.0000 | 0.0765 | 0.0487 | 0.0000 | 0.0049 | 0.0000 | 0.0000 | 0.1844 | 0.1062 | 0.2587 | 0.0000 | 0.0047 | 0.0524 | 0.0000 | 0.0000 | 0.0000 |
| TCGA-B6-A0WZ-01 | 0.0996 | 0.0000 | 0.0210 | 0.0185 | 0.0000 | 0.0851 | 0.0000 | 0.0393 | 0.0180 | 0.0000 | 0.0074 | 0.0048 | 0.0000 | 0.3337 | 0.0303 | 0.1961 | 0.0000 | 0.0362 | 0.1101 | 0.0000 | 0.0000 | 0.0000 |
| TCGA-B6-A0X0-01 | 0.0856 | 0.0000 | 0.0000 | 0.1347 | 0.0000 | 0.0000 | 0.0000 | 0.2478 | 0.0056 | 0.0000 | 0.0445 | 0.0000 | 0.0260 | 0.1367 | 0.0439 | 0.2304 | 0.0063 | 0.0000 | 0.0384 | 0.0000 | 0.0000 | 0.0000 |
| TCGA-B6-A0X1-01 | 0.0027 | 0.0000 | 0.1105 | 0.0359 | 0.0000 | 0.0480 | 0.0258 | 0.2177 | 0.0084 | 0.0000 | 0.0000 | 0.0810 | 0.0052 | 0.0621 | 0.1059 | 0.0429 | 0.0000 | 0.1069 | 0.1402 | 0.0000 | 0.0021 | 0.0047 |
| TCGA-B6-A0X4-01 | 0.1077 | 0.0000 | 0.0903 | 0.0000 | 0.0000 | 0.1049 | 0.0000 | 0.1089 | 0.0000 | 0.0000 | 0.0000 | 0.0579 | 0.0157 | 0.0937 | 0.0040 | 0.2578 | 0.0000 | 0.0000 | 0.1592 | 0.0000 | 0.0000 | 0.0000 |
| TCGA-B6-A0X5-01 | 0.0121 | 0.0000 | 0.0000 | 0.0208 | 0.0000 | 0.3610 | 0.0000 | 0.1425 | 0.0543 | 0.0000 | 0.0099 | 0.0053 | 0.0777 | 0.1128 | 0.0004 | 0.0055 | 0.0000 | 0.0000 | 0.1977 | 0.0000 | 0.0000 | 0.0000 |
| TCGA-B6-A0X7-01 | 0.1438 | 0.0000 | 0.0912 | 0.0636 | 0.0000 | 0.0860 | 0.0000 | 0.0625 | 0.0097 | 0.0000 | 0.0018 | 0.0172 | 0.0017 | 0.0083 | 0.0736 | 0.2721 | 0.0000 | 0.0046 | 0.1641 | 0.0000 | 0.0000 | 0.0000 |
| TCGA-B6-A1KC-01 | 0.0007 | 0.0000 | 0.0640 | 0.0000 | 0.0000 | 0.1877 | 0.0000 | 0.0356 | 0.0230 | 0.0000 | 0.0172 | 0.0052 | 0.0000 | 0.2239 | 0.0000 | 0.3542 | 0.0000 | 0.0147 | 0.0739 | 0.0000 | 0.0000 | 0.0000 |
| TCGA-B6-A1KF-01 | 0.0096 | 0.0000 | 0.0645 | 0.0000 | 0.0000 | 0.1200 | 0.0000 | 0.0455 | 0.0466 | 0.0000 | 0.0915 | 0.0000 | 0.0000 | 0.4874 | 0.0000 | 0.1234 | 0.0000 | 0.0000 | 0.0114 | 0.0000 | 0.0000 | 0.0000 |
| TCGA-B6-A1KI-01 | 0.0828 | 0.0000 | 0.0350 | 0.0000 | 0.0000 | 0.1041 | 0.0000 | 0.1111 | 0.0184 | 0.0072 | 0.0000 | 0.0448 | 0.0000 | 0.2972 | 0.0875 | 0.1299 | 0.0000 | 0.0000 | 0.0821 | 0.0000 | 0.0000 | 0.0000 |
| TCGA-B6-A1KN-01 | 0.1681 | 0.0000 | 0.0773 | 0.0685 | 0.0000 | 0.1648 | 0.0000 | 0.0892 | 0.0034 | 0.0000 | 0.0453 | 0.0000 | 0.0020 | 0.0010 | 0.0941 | 0.1835 | 0.0000 | 0.0710 | 0.0293 | 0.0000 | 0.0000 | 0.0025 |
| TCGA-B6-A2IU-01 | 0.1105 | 0.0000 | 0.0970 | 0.0729 | 0.0000 | 0.1140 | 0.0000 | 0.0660 | 0.0000 | 0.0000 | 0.0000 | 0.0064 | 0.0392 | 0.0000 | 0.0605 | 0.2761 | 0.0154 | 0.0000 | 0.1421 | 0.0000 | 0.0000 | 0.0000 |
| TCGA-B6-A3ZX-01 | 0.2147 | 0.1511 | 0.0000 | 0.1459 | 0.0154 | 0.0502 | 0.0000 | 0.1573 | 0.0466 | 0.0000 | 0.0149 | 0.0000 | 0.0000 | 0.0948 | 0.0351 | 0.0711 | 0.0000 | 0.0000 | 0.0028 | 0.0000 | 0.0000 | 0.0000 |
| TCGA-B6-A400-01 | 0.0051 | 0.0043 | 0.0000 | 0.0216 | 0.0000 | 0.0000 | 0.0000 | 0.0984 | 0.0721 | 0.0000 | 0.0034 | 0.0007 | 0.0000 | 0.4781 | 0.0000 | 0.1923 | 0.0000 | 0.1043 | 0.0000 | 0.0198 | 0.0000 | 0.0000 |
| TCGA-B6-A401-01 | 0.0313 | 0.0183 | 0.0042 | 0.0000 | 0.0000 | 0.0000 | 0.0000 | 0.2645 | 0.0148 | 0.0000 | 0.0571 | 0.0000 | 0.0000 | 0.5088 | 0.0000 | 0.0442 | 0.0000 | 0.0242 | 0.0327 | 0.0000 | 0.0000 | 0.0000 |
| TCGA-B6-A402-01 | 0.0014 | 0.0094 | 0.0026 | 0.0000 | 0.0000 | 0.1264 | 0.0000 | 0.1230 | 0.0605 | 0.0000 | 0.0725 | 0.0000 | 0.0000 | 0.5580 | 0.0021 | 0.0362 | 0.0000 | 0.0000 | 0.0000 | 0.0081 | 0.0000 | 0.0000 |
| TCGA-B6-A408-01 | 0.0683 | 0.0000 | 0.0456 | 0.0489 | 0.0000 | 0.1427 | 0.0000 | 0.1176 | 0.0522 | 0.0000 | 0.0300 | 0.0000 | 0.0000 | 0.1895 | 0.0489 | 0.2302 | 0.0000 | 0.0000 | 0.0261 | 0.0000 | 0.0000 | 0.0000 |
| TCGA-B6-A409-01 | 0.0638 | 0.0000 | 0.0572 | 0.0628 | 0.0000 | 0.2094 | 0.0000 | 0.0450 | 0.0409 | 0.0000 | 0.0049 | 0.0124 | 0.0039 | 0.1499 | 0.0873 | 0.2185 | 0.0000 | 0.0071 | 0.0368 | 0.0000 | 0.0000 | 0.0000 |
| TCGA-B6-A40B-01 | 0.0000 | 0.0068 | 0.0142 | 0.0000 | 0.0000 | 0.0529 | 0.0000 | 0.1349 | 0.0100 | 0.0000 | 0.0000 | 0.0244 | 0.0000 | 0.4112 | 0.0000 | 0.1453 | 0.0000 | 0.0374 | 0.1629 | 0.0000 | 0.0000 | 0.0000 |
| TCGA-B6-A40C-01 | 0.0865 | 0.0000 | 0.0586 | 0.0921 | 0.0000 | 0.1573 | 0.0000 | 0.0829 | 0.0595 | 0.0000 | 0.0471 | 0.0000 | 0.0714 | 0.0000 | 0.0474 | 0.2882 | 0.0000 | 0.0000 | 0.0090 | 0.0000 | 0.0000 | 0.0000 |
| TCGA-BH-A0AU-01 | 0.0941 | 0.0000 | 0.1316 | 0.0199 | 0.0000 | 0.1821 | 0.0022 | 0.0664 | 0.0352 | 0.0072 | 0.0000 | 0.0000 | 0.0000 | 0.1526 | 0.0617 | 0.1952 | 0.0000 | 0.0082 | 0.0435 | 0.0000 | 0.0000 | 0.0000 |
| TCGA-BH-A0AV-01 | 0.0010 | 0.0000 | 0.0132 | 0.0000 | 0.0000 | 0.0746 | 0.0000 | 0.0214 | 0.0364 | 0.0000 | 0.0000 | 0.0245 | 0.0000 | 0.6350 | 0.0109 | 0.1321 | 0.0000 | 0.0000 | 0.0508 | 0.0000 | 0.0000 | 0.0000 |
| TCGA-BH-A0AW-01 | 0.0524 | 0.0000 | 0.0000 | 0.0501 | 0.0000 | 0.2456 | 0.0045 | 0.0749 | 0.0760 | 0.0000 | 0.0024 | 0.0137 | 0.0166 | 0.1403 | 0.1029 | 0.1312 | 0.0032 | 0.0000 | 0.0862 | 0.0000 | 0.0000 | 0.0000 |
| TCGA-BH-A0AY-01 | 0.0568 | 0.0000 | 0.0000 | 0.0608 | 0.0000 | 0.2313 | 0.0064 | 0.0223 | 0.0111 | 0.0000 | 0.0039 | 0.0000 | 0.0000 | 0.0437 | 0.0536 | 0.3320 | 0.0109 | 0.0009 | 0.1664 | 0.0000 | 0.0000 | 0.0000 |
| TCGA-BH-A0AZ-01 | 0.0807 | 0.0000 | 0.0558 | 0.0070 | 0.0000 | 0.1387 | 0.0000 | 0.0347 | 0.0306 | 0.0000 | 0.0303 | 0.0000 | 0.0000 | 0.1993 | 0.0720 | 0.2799 | 0.0000 | 0.0000 | 0.0710 | 0.0000 | 0.0000 | 0.0000 |
| TCGA-BH-A0B0-01 | 0.0736 | 0.0000 | 0.0126 | 0.0740 | 0.0000 | 0.1222 | 0.0000 | 0.1060 | 0.0775 | 0.0000 | 0.0198 | 0.0000 | 0.0000 | 0.1911 | 0.0626 | 0.1323 | 0.0000 | 0.0369 | 0.0913 | 0.0000 | 0.0000 | 0.0000 |
| TCGA-BH-A0B1-01 | 0.0572 | 0.0000 | 0.0466 | 0.0000 | 0.0000 | 0.2479 | 0.0000 | 0.1172 | 0.0082 | 0.0000 | 0.0000 | 0.0376 | 0.0039 | 0.0625 | 0.0919 | 0.2125 | 0.0000 | 0.0157 | 0.0986 | 0.0000 | 0.0000 | 0.0000 |
| TCGA-BH-A0B3-01 | 0.0333 | 0.0000 | 0.1786 | 0.0595 | 0.0000 | 0.0769 | 0.0000 | 0.0820 | 0.0516 | 0.0000 | 0.0422 | 0.0000 | 0.0036 | 0.0966 | 0.1786 | 0.1755 | 0.0000 | 0.0000 | 0.0216 | 0.0000 | 0.0000 | 0.0000 |
| TCGA-BH-A0B4-01 | 0.0612 | 0.0000 | 0.0094 | 0.0350 | 0.0000 | 0.2458 | 0.0000 | 0.0484 | 0.0504 | 0.0000 | 0.0041 | 0.0116 | 0.0063 | 0.0880 | 0.1178 | 0.2547 | 0.0000 | 0.0000 | 0.0672 | 0.0000 | 0.0000 | 0.0000 |
| TCGA-BH-A0B5-01 | 0.0476 | 0.0000 | 0.0095 | 0.0000 | 0.0000 | 0.1617 | 0.0000 | 0.0725 | 0.0298 | 0.0000 | 0.0263 | 0.0000 | 0.0000 | 0.2109 | 0.0631 | 0.2132 | 0.0000 | 0.0000 | 0.1654 | 0.0000 | 0.0000 | 0.0000 |
| TCGA-BH-A0B6-01 | 0.0523 | 0.0138 | 0.0000 | 0.1861 | 0.0000 | 0.1316 | 0.0000 | 0.0661 | 0.1237 | 0.0000 | 0.0196 | 0.0336 | 0.0187 | 0.1142 | 0.1131 | 0.0816 | 0.0026 | 0.0000 | 0.0431 | 0.0000 | 0.0000 | 0.0000 |
| TCGA-BH-A0B7-01 | 0.1232 | 0.0000 | 0.0000 | 0.0341 | 0.0000 | 0.1768 | 0.0000 | 0.1098 | 0.0145 | 0.0174 | 0.0113 | 0.0000 | 0.0000 | 0.2675 | 0.0758 | 0.1429 | 0.0000 | 0.0000 | 0.0267 | 0.0000 | 0.0000 | 0.0000 |
| TCGA-BH-A0B8-01 | 0.0560 | 0.0000 | 0.0265 | 0.0368 | 0.0000 | 0.1781 | 0.0000 | 0.0579 | 0.0090 | 0.0000 | 0.0017 | 0.0150 | 0.0063 | 0.0030 | 0.0405 | 0.3539 | 0.0068 | 0.0184 | 0.1900 | 0.0000 | 0.0000 | 0.0000 |
| TCGA-BH-A0B9-01 | 0.0193 | 0.0178 | 0.0000 | 0.1291 | 0.0000 | 0.1389 | 0.0160 | 0.1389 | 0.0715 | 0.0000 | 0.0154 | 0.0000 | 0.0093 | 0.1175 | 0.1789 | 0.0957 | 0.0244 | 0.0000 | 0.0275 | 0.0000 | 0.0000 | 0.0000 |
| TCGA-BH-A0BA-01 | 0.1150 | 0.0000 | 0.1681 | 0.1017 | 0.0000 | 0.1939 | 0.0000 | 0.0705 | 0.0000 | 0.0000 | 0.0049 | 0.0000 | 0.0175 | 0.0000 | 0.0502 | 0.1417 | 0.0007 | 0.0000 | 0.1358 | 0.0000 | 0.0000 | 0.0000 |
| TCGA-BH-A0BC-01 | 0.0505 | 0.0000 | 0.0101 | 0.0635 | 0.0000 | 0.1089 | 0.0000 | 0.0347 | 0.0646 | 0.0000 | 0.0225 | 0.0000 | 0.0000 | 0.2996 | 0.0754 | 0.2446 | 0.0000 | 0.0000 | 0.0257 | 0.0000 | 0.0000 | 0.0000 |
| TCGA-BH-A0BD-01 | 0.0583 | 0.0000 | 0.0139 | 0.0831 | 0.0000 | 0.1316 | 0.0000 | 0.1066 | 0.0300 | 0.0000 | 0.0132 | 0.0000 | 0.0209 | 0.0577 | 0.0738 | 0.2236 | 0.0000 | 0.0823 | 0.1052 | 0.0000 | 0.0000 | 0.0000 |
| TCGA-BH-A0BF-01 | 0.0249 | 0.0000 | 0.0627 | 0.0980 | 0.0000 | 0.1541 | 0.0000 | 0.1347 | 0.0428 | 0.0000 | 0.0144 | 0.0000 | 0.0051 | 0.0425 | 0.0783 | 0.2325 | 0.0000 | 0.0000 | 0.1101 | 0.0000 | 0.0000 | 0.0000 |
| TCGA-BH-A0BG-01 | 0.0394 | 0.0159 | 0.0000 | 0.0305 | 0.0000 | 0.2285 | 0.0000 | 0.1402 | 0.0487 | 0.0000 | 0.0122 | 0.0360 | 0.0140 | 0.0937 | 0.1506 | 0.0748 | 0.0000 | 0.0147 | 0.1009 | 0.0000 | 0.0000 | 0.0000 |
| TCGA-BH-A0BJ-01 | 0.0666 | 0.0000 | 0.0606 | 0.0501 | 0.0000 | 0.1929 | 0.0000 | 0.0071 | 0.0247 | 0.0000 | 0.0282 | 0.0000 | 0.0000 | 0.2453 | 0.0441 | 0.2158 | 0.0000 | 0.0000 | 0.0645 | 0.0000 | 0.0000 | 0.0000 |
| TCGA-BH-A0BL-01 | 0.0675 | 0.0000 | 0.0045 | 0.0377 | 0.0000 | 0.1174 | 0.0003 | 0.1457 | 0.0037 | 0.0000 | 0.0204 | 0.0084 | 0.0143 | 0.0631 | 0.1945 | 0.2299 | 0.0289 | 0.0201 | 0.0437 | 0.0000 | 0.0000 | 0.0000 |
| TCGA-BH-A0BM-01 | 0.1145 | 0.0000 | 0.1182 | 0.0692 | 0.0000 | 0.1150 | 0.0000 | 0.1166 | 0.0277 | 0.0000 | 0.0049 | 0.0167 | 0.0152 | 0.0683 | 0.0718 | 0.1868 | 0.0000 | 0.0000 | 0.0751 | 0.0000 | 0.0000 | 0.0000 |
| TCGA-BH-A0BO-01 | 0.0000 | 0.0000 | 0.0265 | 0.0000 | 0.0000 | 0.3910 | 0.0000 | 0.0651 | 0.0000 | 0.0000 | 0.0126 | 0.0000 | 0.1459 | 0.0466 | 0.0000 | 0.0472 | 0.0000 | 0.0446 | 0.0779 | 0.0000 | 0.0000 | 0.1427 |
| TCGA-BH-A0BP-01 | 0.0035 | 0.0078 | 0.0043 | 0.0000 | 0.0000 | 0.1322 | 0.0000 | 0.0750 | 0.0390 | 0.0000 | 0.0317 | 0.0086 | 0.0000 | 0.4767 | 0.0061 | 0.1882 | 0.0000 | 0.0000 | 0.0271 | 0.0000 | 0.0000 | 0.0000 |
| TCGA-BH-A0BQ-01 | 0.0394 | 0.0000 | 0.0610 | 0.0648 | 0.0000 | 0.2028 | 0.0000 | 0.0401 | 0.0431 | 0.0000 | 0.0179 | 0.0000 | 0.0000 | 0.1838 | 0.0730 | 0.1994 | 0.0000 | 0.0044 | 0.0703 | 0.0000 | 0.0000 | 0.0000 |
| TCGA-BH-A0BR-01 | 0.0453 | 0.0000 | 0.0000 | 0.0240 | 0.0000 | 0.0730 | 0.0013 | 0.0504 | 0.0463 | 0.0000 | 0.0158 | 0.0000 | 0.0000 | 0.4100 | 0.0188 | 0.2568 | 0.0000 | 0.0196 | 0.0388 | 0.0000 | 0.0000 | 0.0000 |
| TCGA-BH-A0BS-01 | 0.0000 | 0.0000 | 0.0754 | 0.0000 | 0.0000 | 0.3867 | 0.0000 | 0.0627 | 0.0983 | 0.0000 | 0.0641 | 0.0000 | 0.0368 | 0.1836 | 0.0000 | 0.0470 | 0.0021 | 0.0000 | 0.0374 | 0.0000 | 0.0060 | 0.0000 |
| TCGA-BH-A0BT-01 | 0.0823 | 0.0000 | 0.0218 | 0.0792 | 0.0000 | 0.1184 | 0.0000 | 0.1451 | 0.0515 | 0.0000 | 0.0000 | 0.0312 | 0.0025 | 0.0944 | 0.0772 | 0.1943 | 0.0162 | 0.0136 | 0.0724 | 0.0000 | 0.0000 | 0.0000 |
| TCGA-BH-A0BV-01 | 0.0965 | 0.0000 | 0.0140 | 0.0378 | 0.0000 | 0.1703 | 0.0000 | 0.0334 | 0.0484 | 0.0000 | 0.0467 | 0.0000 | 0.0000 | 0.2484 | 0.0327 | 0.2446 | 0.0000 | 0.0000 | 0.0000 | 0.0272 | 0.0000 | 0.0000 |
| TCGA-BH-A0BW-01 | 0.0391 | 0.0000 | 0.0209 | 0.0000 | 0.0000 | 0.1848 | 0.0130 | 0.0858 | 0.0002 | 0.0000 | 0.0799 | 0.0000 | 0.0010 | 0.0789 | 0.2791 | 0.1565 | 0.0000 | 0.0000 | 0.0609 | 0.0000 | 0.0000 | 0.0000 |
| TCGA-BH-A0BZ-01 | 0.0272 | 0.0000 | 0.0168 | 0.1148 | 0.0000 | 0.1650 | 0.0033 | 0.0669 | 0.0555 | 0.0000 | 0.0000 | 0.0234 | 0.0158 | 0.1382 | 0.1498 | 0.1353 | 0.0000 | 0.0000 | 0.0879 | 0.0000 | 0.0000 | 0.0000 |
| TCGA-BH-A0C0-01 | 0.0383 | 0.0000 | 0.0000 | 0.1484 | 0.0000 | 0.0546 | 0.0300 | 0.1020 | 0.0724 | 0.0000 | 0.0225 | 0.0033 | 0.0214 | 0.1095 | 0.1652 | 0.1573 | 0.0014 | 0.0000 | 0.0737 | 0.0000 | 0.0000 | 0.0000 |
| TCGA-BH-A0C1-01 | 0.1029 | 0.0000 | 0.0386 | 0.1008 | 0.0000 | 0.3028 | 0.0000 | 0.0637 | 0.0007 | 0.0000 | 0.0105 | 0.0000 | 0.0066 | 0.0000 | 0.0722 | 0.2213 | 0.0000 | 0.0200 | 0.0598 | 0.0000 | 0.0000 | 0.0000 |
| TCGA-BH-A0C3-01 | 0.0676 | 0.0000 | 0.0169 | 0.0494 | 0.0000 | 0.1711 | 0.0000 | 0.0391 | 0.0162 | 0.0000 | 0.0145 | 0.0000 | 0.0458 | 0.0534 | 0.1282 | 0.2404 | 0.0126 | 0.0000 | 0.1448 | 0.0000 | 0.0000 | 0.0000 |
| TCGA-BH-A0C7-01 | 0.1188 | 0.0000 | 0.0424 | 0.0131 | 0.0000 | 0.2230 | 0.0025 | 0.0411 | 0.0000 | 0.0000 | 0.0215 | 0.0000 | 0.0000 | 0.1107 | 0.0790 | 0.2224 | 0.0000 | 0.0124 | 0.1131 | 0.0000 | 0.0000 | 0.0000 |
| TCGA-BH-A0DD-01 | 0.0792 | 0.0000 | 0.0000 | 0.0526 | 0.0000 | 0.0857 | 0.0000 | 0.1566 | 0.0164 | 0.0000 | 0.0023 | 0.0000 | 0.0000 | 0.2531 | 0.1208 | 0.1937 | 0.0000 | 0.0080 | 0.0318 | 0.0000 | 0.0000 | 0.0000 |
| TCGA-BH-A0DE-01 | 0.0631 | 0.0000 | 0.0124 | 0.0663 | 0.0000 | 0.1140 | 0.0000 | 0.0676 | 0.0192 | 0.0000 | 0.0338 | 0.0000 | 0.0019 | 0.0323 | 0.0934 | 0.4310 | 0.0040 | 0.0000 | 0.0610 | 0.0000 | 0.0000 | 0.0000 |
| TCGA-BH-A0DG-01 | 0.1257 | 0.0000 | 0.0393 | 0.0456 | 0.0000 | 0.1312 | 0.0141 | 0.0606 | 0.0266 | 0.0000 | 0.0501 | 0.0000 | 0.0000 | 0.1161 | 0.0708 | 0.2677 | 0.0000 | 0.0000 | 0.0522 | 0.0000 | 0.0000 | 0.0000 |
| TCGA-BH-A0DH-01 | 0.0417 | 0.0000 | 0.1727 | 0.0405 | 0.0000 | 0.0196 | 0.0000 | 0.0259 | 0.0572 | 0.0000 | 0.0069 | 0.0000 | 0.0000 | 0.2432 | 0.0419 | 0.3016 | 0.0000 | 0.0000 | 0.0487 | 0.0000 | 0.0000 | 0.0000 |
| TCGA-BH-A0DI-01 | 0.0861 | 0.0000 | 0.0334 | 0.0427 | 0.0000 | 0.1381 | 0.0000 | 0.0379 | 0.0017 | 0.0068 | 0.0000 | 0.0000 | 0.0000 | 0.3140 | 0.0559 | 0.2106 | 0.0000 | 0.0000 | 0.0729 | 0.0000 | 0.0000 | 0.0000 |
| TCGA-BH-A0DK-01 | 0.0872 | 0.0000 | 0.0380 | 0.1045 | 0.0000 | 0.2174 | 0.0000 | 0.0710 | 0.0515 | 0.0000 | 0.0170 | 0.0000 | 0.0000 | 0.1044 | 0.0986 | 0.1765 | 0.0000 | 0.0000 | 0.0339 | 0.0000 | 0.0000 | 0.0000 |
| TCGA-BH-A0DL-01 | 0.0033 | 0.0000 | 0.0222 | 0.0234 | 0.0000 | 0.0325 | 0.0168 | 0.1013 | 0.0500 | 0.0000 | 0.0129 | 0.0065 | 0.0004 | 0.2844 | 0.1633 | 0.2021 | 0.0508 | 0.0130 | 0.0172 | 0.0000 | 0.0000 | 0.0000 |
| TCGA-BH-A0DO-01 | 0.0000 | 0.0310 | 0.0630 | 0.0000 | 0.0000 | 0.1217 | 0.0000 | 0.1621 | 0.0000 | 0.0000 | 0.0250 | 0.0384 | 0.0000 | 0.4678 | 0.0000 | 0.0118 | 0.0000 | 0.0000 | 0.0793 | 0.0000 | 0.0000 | 0.0000 |
| TCGA-BH-A0DP-01 | 0.1200 | 0.0000 | 0.0423 | 0.0906 | 0.0000 | 0.1167 | 0.0000 | 0.1070 | 0.0000 | 0.0000 | 0.0000 | 0.0054 | 0.0000 | 0.0704 | 0.0567 | 0.3201 | 0.0000 | 0.0000 | 0.0709 | 0.0000 | 0.0000 | 0.0000 |
| TCGA-BH-A0DQ-01 | 0.0028 | 0.0050 | 0.0103 | 0.0000 | 0.0000 | 0.0945 | 0.0000 | 0.0279 | 0.0365 | 0.0000 | 0.0145 | 0.0086 | 0.0000 | 0.3953 | 0.0216 | 0.2285 | 0.0000 | 0.0000 | 0.1544 | 0.0000 | 0.0000 | 0.0000 |
| TCGA-BH-A0DS-01 | 0.0618 | 0.0000 | 0.0229 | 0.0923 | 0.0000 | 0.1085 | 0.0000 | 0.0471 | 0.0110 | 0.0000 | 0.0049 | 0.0000 | 0.0000 | 0.3073 | 0.0454 | 0.2752 | 0.0087 | 0.0000 | 0.0148 | 0.0000 | 0.0000 | 0.0000 |
| TCGA-BH-A0DT-01 | 0.1125 | 0.0000 | 0.0198 | 0.0727 | 0.0000 | 0.1609 | 0.0000 | 0.0978 | 0.0207 | 0.0000 | 0.0225 | 0.0000 | 0.0000 | 0.0836 | 0.1119 | 0.1790 | 0.0650 | 0.0102 | 0.0433 | 0.0000 | 0.0000 | 0.0000 |
| TCGA-BH-A0DV-01 | 0.0856 | 0.0000 | 0.0331 | 0.0245 | 0.0000 | 0.1506 | 0.0000 | 0.0499 | 0.0002 | 0.0000 | 0.0160 | 0.0057 | 0.0000 | 0.0995 | 0.0058 | 0.3510 | 0.0000 | 0.0055 | 0.1727 | 0.0000 | 0.0000 | 0.0000 |
| TCGA-BH-A0DX-01 | 0.0299 | 0.0000 | 0.0013 | 0.0111 | 0.0000 | 0.0825 | 0.0000 | 0.0075 | 0.0066 | 0.0000 | 0.0125 | 0.0000 | 0.0000 | 0.5630 | 0.0345 | 0.1859 | 0.0000 | 0.0000 | 0.0651 | 0.0000 | 0.0000 | 0.0000 |
| TCGA-BH-A0DZ-01 | 0.0432 | 0.0000 | 0.0541 | 0.0291 | 0.0000 | 0.1515 | 0.0129 | 0.0250 | 0.0023 | 0.0000 | 0.0162 | 0.0000 | 0.0000 | 0.1624 | 0.1013 | 0.3492 | 0.0000 | 0.0058 | 0.0438 | 0.0000 | 0.0000 | 0.0035 |
| TCGA-BH-A0E0-01 | 0.0128 | 0.0000 | 0.0189 | 0.0000 | 0.0000 | 0.2323 | 0.0000 | 0.1961 | 0.0185 | 0.0000 | 0.0000 | 0.0306 | 0.0300 | 0.0532 | 0.0000 | 0.1201 | 0.0844 | 0.0931 | 0.1037 | 0.0000 | 0.0063 | 0.0000 |
| TCGA-BH-A0E1-01 | 0.0830 | 0.0000 | 0.0276 | 0.0073 | 0.0000 | 0.0331 | 0.0000 | 0.0455 | 0.0096 | 0.0000 | 0.0354 | 0.0000 | 0.0000 | 0.4128 | 0.0141 | 0.2574 | 0.0000 | 0.0000 | 0.0742 | 0.0000 | 0.0000 | 0.0000 |
| TCGA-BH-A0E2-01 | 0.0576 | 0.0000 | 0.0130 | 0.0399 | 0.0000 | 0.1792 | 0.0000 | 0.0350 | 0.0486 | 0.0000 | 0.0143 | 0.0000 | 0.0000 | 0.3272 | 0.0734 | 0.1329 | 0.0000 | 0.0000 | 0.0788 | 0.0000 | 0.0000 | 0.0000 |
| TCGA-BH-A0E6-01 | 0.1338 | 0.0000 | 0.1311 | 0.1187 | 0.0000 | 0.0374 | 0.0000 | 0.0954 | 0.0551 | 0.0000 | 0.0031 | 0.0073 | 0.0000 | 0.2804 | 0.0559 | 0.0717 | 0.0000 | 0.0000 | 0.0100 | 0.0000 | 0.0000 | 0.0000 |
| TCGA-BH-A0E7-01 | 0.0111 | 0.0000 | 0.0206 | 0.0000 | 0.0000 | 0.1151 | 0.0000 | 0.1016 | 0.0094 | 0.0000 | 0.0101 | 0.0070 | 0.0090 | 0.0454 | 0.0211 | 0.4611 | 0.0000 | 0.0000 | 0.1883 | 0.0000 | 0.0000 | 0.0000 |
| TCGA-BH-A0E9-01 | 0.1028 | 0.0000 | 0.0366 | 0.1309 | 0.0000 | 0.2158 | 0.0000 | 0.0242 | 0.0000 | 0.0000 | 0.0084 | 0.0000 | 0.0134 | 0.0000 | 0.0289 | 0.2768 | 0.0369 | 0.0179 | 0.1072 | 0.0000 | 0.0000 | 0.0000 |
| TCGA-BH-A0EA-01 | 0.0568 | 0.0000 | 0.0058 | 0.0556 | 0.0000 | 0.0852 | 0.0000 | 0.0565 | 0.0190 | 0.0000 | 0.0145 | 0.0000 | 0.0000 | 0.2900 | 0.0370 | 0.2574 | 0.0750 | 0.0000 | 0.0471 | 0.0000 | 0.0000 | 0.0000 |
| TCGA-BH-A0EB-01 | 0.0582 | 0.0000 | 0.0611 | 0.0301 | 0.0000 | 0.1579 | 0.0007 | 0.0513 | 0.0218 | 0.0000 | 0.0316 | 0.0000 | 0.0633 | 0.0000 | 0.0832 | 0.3234 | 0.0627 | 0.0000 | 0.0545 | 0.0000 | 0.0000 | 0.0003 |
| TCGA-BH-A0EE-01 | 0.1020 | 0.0000 | 0.0395 | 0.0087 | 0.0000 | 0.1545 | 0.0000 | 0.0804 | 0.0496 | 0.0000 | 0.0702 | 0.0000 | 0.0000 | 0.0586 | 0.1834 | 0.1307 | 0.0000 | 0.0808 | 0.0414 | 0.0000 | 0.0000 | 0.0000 |
| TCGA-BH-A0EI-01 | 0.0276 | 0.0000 | 0.0153 | 0.0000 | 0.0000 | 0.1505 | 0.0000 | 0.0554 | 0.0189 | 0.0000 | 0.0140 | 0.0053 | 0.0000 | 0.1782 | 0.0080 | 0.3615 | 0.0000 | 0.0086 | 0.1568 | 0.0000 | 0.0000 | 0.0000 |
| TCGA-BH-A0GY-01 | 0.0232 | 0.0214 | 0.0000 | 0.0000 | 0.0000 | 0.1506 | 0.0000 | 0.0660 | 0.0521 | 0.0000 | 0.0212 | 0.0000 | 0.0000 | 0.4167 | 0.0969 | 0.0676 | 0.0000 | 0.0000 | 0.0720 | 0.0010 | 0.0000 | 0.0112 |
| TCGA-BH-A0GZ-01 | 0.1152 | 0.0000 | 0.0731 | 0.0600 | 0.0000 | 0.1107 | 0.0000 | 0.0638 | 0.0261 | 0.0000 | 0.0024 | 0.0133 | 0.0006 | 0.0800 | 0.0328 | 0.3061 | 0.0163 | 0.0005 | 0.0991 | 0.0000 | 0.0000 | 0.0000 |
| TCGA-BH-A0H0-01 | 0.1149 | 0.0000 | 0.0312 | 0.0412 | 0.0000 | 0.1228 | 0.0000 | 0.0393 | 0.0595 | 0.0000 | 0.0077 | 0.0028 | 0.0212 | 0.0000 | 0.0471 | 0.3389 | 0.0024 | 0.0000 | 0.1709 | 0.0000 | 0.0000 | 0.0000 |
| TCGA-BH-A0H3-01 | 0.0490 | 0.0000 | 0.0772 | 0.0673 | 0.0000 | 0.0946 | 0.0000 | 0.0570 | 0.0228 | 0.0000 | 0.0171 | 0.0000 | 0.0020 | 0.1264 | 0.0728 | 0.3337 | 0.0000 | 0.0000 | 0.0802 | 0.0000 | 0.0000 | 0.0000 |
| TCGA-BH-A0H5-01 | 0.0765 | 0.0630 | 0.0000 | 0.1818 | 0.0000 | 0.1124 | 0.0099 | 0.0710 | 0.0867 | 0.0000 | 0.0367 | 0.0000 | 0.0233 | 0.1162 | 0.1233 | 0.0950 | 0.0000 | 0.0000 | 0.0041 | 0.0000 | 0.0000 | 0.0000 |
| TCGA-BH-A0H6-01 | 0.0531 | 0.0000 | 0.0046 | 0.0132 | 0.0000 | 0.1567 | 0.0000 | 0.0246 | 0.0007 | 0.0000 | 0.0142 | 0.0000 | 0.0146 | 0.0885 | 0.0471 | 0.4915 | 0.0030 | 0.0000 | 0.0837 | 0.0000 | 0.0000 | 0.0044 |
| TCGA-BH-A0H7-01 | 0.1133 | 0.0000 | 0.0577 | 0.0356 | 0.0000 | 0.0994 | 0.0000 | 0.0447 | 0.0214 | 0.0000 | 0.0041 | 0.0000 | 0.0000 | 0.2864 | 0.0702 | 0.2091 | 0.0000 | 0.0000 | 0.0579 | 0.0000 | 0.0000 | 0.0000 |
| TCGA-BH-A0H9-01 | 0.0783 | 0.0000 | 0.0292 | 0.0122 | 0.0000 | 0.2022 | 0.0000 | 0.0413 | 0.0127 | 0.0000 | 0.0199 | 0.0000 | 0.0000 | 0.2348 | 0.0506 | 0.3005 | 0.0000 | 0.0000 | 0.0183 | 0.0000 | 0.0000 | 0.0000 |
| TCGA-BH-A0HA-01 | 0.0423 | 0.0000 | 0.0211 | 0.0479 | 0.0000 | 0.1517 | 0.0000 | 0.0771 | 0.0953 | 0.0000 | 0.0214 | 0.0090 | 0.0143 | 0.0482 | 0.1555 | 0.1520 | 0.0097 | 0.0000 | 0.1544 | 0.0000 | 0.0000 | 0.0000 |
| TCGA-BH-A0HB-01 | 0.0554 | 0.0000 | 0.0056 | 0.0364 | 0.0000 | 0.2437 | 0.0000 | 0.0608 | 0.0957 | 0.0000 | 0.0178 | 0.0000 | 0.0000 | 0.1798 | 0.0770 | 0.1835 | 0.0058 | 0.0000 | 0.0386 | 0.0000 | 0.0000 | 0.0000 |
| TCGA-BH-A0HF-01 | 0.0568 | 0.0000 | 0.0078 | 0.0273 | 0.0000 | 0.0978 | 0.0000 | 0.0251 | 0.0100 | 0.0000 | 0.0132 | 0.0000 | 0.0000 | 0.5107 | 0.0510 | 0.1607 | 0.0000 | 0.0000 | 0.0396 | 0.0000 | 0.0000 | 0.0000 |
| TCGA-BH-A0HI-01 | 0.1154 | 0.0000 | 0.0007 | 0.0115 | 0.0000 | 0.0847 | 0.0000 | 0.0625 | 0.0204 | 0.0000 | 0.0028 | 0.0000 | 0.0000 | 0.3161 | 0.0374 | 0.3179 | 0.0000 | 0.0000 | 0.0289 | 0.0017 | 0.0000 | 0.0000 |
| TCGA-BH-A0HK-01 | 0.1478 | 0.0000 | 0.1559 | 0.0235 | 0.0000 | 0.1790 | 0.0000 | 0.0359 | 0.0246 | 0.0000 | 0.0303 | 0.0000 | 0.0181 | 0.0000 | 0.0457 | 0.2696 | 0.0481 | 0.0044 | 0.0164 | 0.0000 | 0.0000 | 0.0007 |
| TCGA-BH-A0HL-01 | 0.0000 | 0.0000 | 0.0877 | 0.0000 | 0.0000 | 0.2005 | 0.0012 | 0.0212 | 0.0464 | 0.0000 | 0.0867 | 0.0000 | 0.0000 | 0.3603 | 0.0000 | 0.1366 | 0.0000 | 0.0326 | 0.0268 | 0.0000 | 0.0000 | 0.0000 |
| TCGA-BH-A0HN-01 | 0.0000 | 0.0104 | 0.0054 | 0.0000 | 0.0000 | 0.2300 | 0.0000 | 0.0238 | 0.1314 | 0.0000 | 0.1372 | 0.0000 | 0.0795 | 0.0000 | 0.0065 | 0.2810 | 0.0099 | 0.0000 | 0.0757 | 0.0000 | 0.0056 | 0.0036 |
| TCGA-BH-A0HO-01 | 0.0032 | 0.0035 | 0.0049 | 0.0000 | 0.0000 | 0.1344 | 0.0000 | 0.0746 | 0.0598 | 0.0000 | 0.0431 | 0.0000 | 0.0135 | 0.3809 | 0.0194 | 0.1892 | 0.0000 | 0.0062 | 0.0674 | 0.0000 | 0.0000 | 0.0000 |
| TCGA-BH-A0HP-01 | 0.1181 | 0.0000 | 0.0475 | 0.0176 | 0.0000 | 0.1841 | 0.0000 | 0.0428 | 0.0402 | 0.0000 | 0.0528 | 0.0000 | 0.0355 | 0.0000 | 0.0609 | 0.3616 | 0.0000 | 0.0000 | 0.0388 | 0.0000 | 0.0000 | 0.0000 |
| TCGA-BH-A0HQ-01 | 0.0010 | 0.0071 | 0.0159 | 0.0000 | 0.0000 | 0.1025 | 0.0000 | 0.0128 | 0.0318 | 0.0000 | 0.0426 | 0.0000 | 0.0000 | 0.5425 | 0.0000 | 0.0887 | 0.0000 | 0.0000 | 0.1551 | 0.0000 | 0.0000 | 0.0000 |
| TCGA-BH-A0HU-01 | 0.0896 | 0.0000 | 0.0188 | 0.0972 | 0.0000 | 0.1107 | 0.0000 | 0.0903 | 0.0485 | 0.0000 | 0.0290 | 0.0000 | 0.0034 | 0.1440 | 0.0508 | 0.2371 | 0.0000 | 0.0481 | 0.0325 | 0.0000 | 0.0000 | 0.0000 |
| TCGA-BH-A0HW-01 | 0.1258 | 0.0000 | 0.0126 | 0.0108 | 0.0000 | 0.0285 | 0.0000 | 0.0175 | 0.0364 | 0.0000 | 0.0056 | 0.0138 | 0.0000 | 0.2729 | 0.0000 | 0.2625 | 0.0000 | 0.0839 | 0.1299 | 0.0000 | 0.0000 | 0.0000 |
| TCGA-BH-A0HX-01 | 0.0708 | 0.0000 | 0.0097 | 0.0138 | 0.0000 | 0.1815 | 0.0006 | 0.0076 | 0.0251 | 0.0000 | 0.0335 | 0.0000 | 0.0000 | 0.3693 | 0.0587 | 0.2117 | 0.0000 | 0.0000 | 0.0178 | 0.0000 | 0.0000 | 0.0000 |
| TCGA-BH-A0HY-01 | 0.1131 | 0.0000 | 0.0446 | 0.0230 | 0.0000 | 0.1898 | 0.0000 | 0.0655 | 0.0068 | 0.0000 | 0.0295 | 0.0000 | 0.0000 | 0.1414 | 0.1141 | 0.2212 | 0.0000 | 0.0000 | 0.0509 | 0.0000 | 0.0000 | 0.0000 |
| TCGA-BH-A0RX-01 | 0.0405 | 0.0000 | 0.0242 | 0.0986 | 0.0000 | 0.1481 | 0.0079 | 0.1287 | 0.0185 | 0.0000 | 0.0026 | 0.0500 | 0.0021 | 0.1674 | 0.1350 | 0.1062 | 0.0000 | 0.0108 | 0.0592 | 0.0000 | 0.0000 | 0.0000 |
| TCGA-BH-A0W3-01 | 0.0665 | 0.0000 | 0.0276 | 0.0005 | 0.0000 | 0.1221 | 0.0000 | 0.0473 | 0.0022 | 0.0000 | 0.0155 | 0.0039 | 0.1632 | 0.0000 | 0.0468 | 0.4359 | 0.0000 | 0.0000 | 0.0684 | 0.0000 | 0.0000 | 0.0000 |
| TCGA-BH-A0W4-01 | 0.0028 | 0.0032 | 0.0089 | 0.0000 | 0.0000 | 0.1641 | 0.0000 | 0.0674 | 0.0334 | 0.0000 | 0.0596 | 0.0134 | 0.0000 | 0.4081 | 0.0038 | 0.1760 | 0.0000 | 0.0000 | 0.0593 | 0.0000 | 0.0000 | 0.0000 |
| TCGA-BH-A0W5-01 | 0.2469 | 0.0257 | 0.0000 | 0.0196 | 0.0000 | 0.2162 | 0.0040 | 0.1105 | 0.0315 | 0.0000 | 0.0131 | 0.0000 | 0.0000 | 0.1704 | 0.0360 | 0.0897 | 0.0000 | 0.0000 | 0.0363 | 0.0000 | 0.0000 | 0.0000 |
| TCGA-BH-A0W7-01 | 0.1201 | 0.0000 | 0.0403 | 0.0570 | 0.0000 | 0.0658 | 0.0073 | 0.0134 | 0.0829 | 0.0000 | 0.0405 | 0.0000 | 0.0114 | 0.2328 | 0.1064 | 0.1310 | 0.0000 | 0.0000 | 0.0911 | 0.0000 | 0.0000 | 0.0000 |
| TCGA-BH-A0WA-01 | 0.0368 | 0.0000 | 0.0047 | 0.0376 | 0.0000 | 0.1054 | 0.0000 | 0.0734 | 0.0164 | 0.0000 | 0.0196 | 0.0000 | 0.0000 | 0.4159 | 0.1185 | 0.1046 | 0.0000 | 0.0373 | 0.0298 | 0.0000 | 0.0000 | 0.0000 |
| TCGA-BH-A18F-01 | 0.1100 | 0.0000 | 0.0512 | 0.0554 | 0.0000 | 0.2006 | 0.0000 | 0.0649 | 0.0332 | 0.0000 | 0.0000 | 0.0000 | 0.0000 | 0.1035 | 0.0905 | 0.1973 | 0.0060 | 0.0000 | 0.0874 | 0.0000 | 0.0000 | 0.0000 |
| TCGA-BH-A18G-01 | 0.0000 | 0.0000 | 0.0092 | 0.2270 | 0.0000 | 0.0989 | 0.0000 | 0.0870 | 0.0188 | 0.0000 | 0.0211 | 0.0000 | 0.0345 | 0.1891 | 0.0024 | 0.1346 | 0.0001 | 0.0000 | 0.1719 | 0.0000 | 0.0054 | 0.0000 |
| TCGA-BH-A18H-01 | 0.1122 | 0.0000 | 0.0031 | 0.0242 | 0.0000 | 0.1666 | 0.0409 | 0.0773 | 0.0412 | 0.0000 | 0.0784 | 0.0000 | 0.0000 | 0.2627 | 0.1111 | 0.0628 | 0.0021 | 0.0000 | 0.0173 | 0.0000 | 0.0000 | 0.0000 |
| TCGA-BH-A18I-01 | 0.0741 | 0.0000 | 0.0253 | 0.0314 | 0.0000 | 0.1528 | 0.0000 | 0.0981 | 0.0470 | 0.0000 | 0.0217 | 0.0000 | 0.0037 | 0.0851 | 0.1272 | 0.1903 | 0.0000 | 0.0266 | 0.1167 | 0.0000 | 0.0000 | 0.0000 |
| TCGA-BH-A18J-01 | 0.0000 | 0.0055 | 0.0010 | 0.0000 | 0.0000 | 0.2592 | 0.0000 | 0.0327 | 0.0696 | 0.0000 | 0.0393 | 0.0186 | 0.0000 | 0.3330 | 0.0007 | 0.1866 | 0.0000 | 0.0000 | 0.0537 | 0.0000 | 0.0000 | 0.0000 |
| TCGA-BH-A18K-01 | 0.0432 | 0.0000 | 0.0062 | 0.0000 | 0.0000 | 0.0325 | 0.0000 | 0.1111 | 0.0395 | 0.0000 | 0.0257 | 0.0000 | 0.0000 | 0.3819 | 0.0571 | 0.2521 | 0.0000 | 0.0000 | 0.0507 | 0.0000 | 0.0000 | 0.0000 |
| TCGA-BH-A18L-01 | 0.0017 | 0.0000 | 0.0147 | 0.0000 | 0.0000 | 0.3425 | 0.0000 | 0.1136 | 0.0363 | 0.0000 | 0.0812 | 0.0000 | 0.0537 | 0.0169 | 0.0174 | 0.1656 | 0.0005 | 0.0000 | 0.1560 | 0.0000 | 0.0000 | 0.0000 |
| TCGA-BH-A18M-01 | 0.0519 | 0.0000 | 0.0467 | 0.0000 | 0.0000 | 0.2816 | 0.0000 | 0.0164 | 0.0000 | 0.0000 | 0.0450 | 0.0000 | 0.0268 | 0.0000 | 0.0625 | 0.2972 | 0.0000 | 0.0178 | 0.1542 | 0.0000 | 0.0000 | 0.0000 |
| TCGA-BH-A18N-01 | 0.0005 | 0.0064 | 0.0009 | 0.0000 | 0.0000 | 0.4002 | 0.0000 | 0.0076 | 0.0486 | 0.0000 | 0.1049 | 0.0000 | 0.0402 | 0.2292 | 0.0098 | 0.1364 | 0.0000 | 0.0000 | 0.0154 | 0.0000 | 0.0000 | 0.0000 |
| TCGA-BH-A18P-01 | 0.1084 | 0.0000 | 0.0146 | 0.0245 | 0.0000 | 0.1068 | 0.0023 | 0.0785 | 0.0294 | 0.0000 | 0.0281 | 0.0000 | 0.0000 | 0.2142 | 0.1081 | 0.2091 | 0.0000 | 0.0351 | 0.0409 | 0.0000 | 0.0000 | 0.0000 |
| TCGA-BH-A18Q-01 | 0.1195 | 0.0000 | 0.0445 | 0.0827 | 0.0000 | 0.0583 | 0.0000 | 0.1465 | 0.0101 | 0.0000 | 0.0208 | 0.0219 | 0.0207 | 0.0051 | 0.1496 | 0.1923 | 0.0000 | 0.0684 | 0.0596 | 0.0000 | 0.0000 | 0.0000 |
| TCGA-BH-A18R-01 | 0.1327 | 0.0000 | 0.1030 | 0.0245 | 0.0000 | 0.1607 | 0.0333 | 0.0628 | 0.0000 | 0.0000 | 0.0111 | 0.0000 | 0.0059 | 0.0082 | 0.0732 | 0.2429 | 0.0419 | 0.0151 | 0.0847 | 0.0000 | 0.0000 | 0.0000 |
| TCGA-BH-A18S-01 | 0.0003 | 0.0046 | 0.0033 | 0.0000 | 0.0000 | 0.2330 | 0.0000 | 0.0443 | 0.0373 | 0.0000 | 0.0587 | 0.0049 | 0.0297 | 0.0956 | 0.0000 | 0.2434 | 0.0000 | 0.0075 | 0.2303 | 0.0000 | 0.0071 | 0.0000 |
| TCGA-BH-A18T-01 | 0.0522 | 0.0000 | 0.0352 | 0.0000 | 0.0000 | 0.1395 | 0.0000 | 0.1076 | 0.0356 | 0.0000 | 0.0000 | 0.0346 | 0.0000 | 0.3534 | 0.0934 | 0.1140 | 0.0000 | 0.0000 | 0.0345 | 0.0000 | 0.0000 | 0.0000 |
| TCGA-BH-A18U-01 | 0.0311 | 0.0000 | 0.0505 | 0.0000 | 0.0000 | 0.1248 | 0.0000 | 0.1070 | 0.0029 | 0.0000 | 0.0117 | 0.0136 | 0.0000 | 0.1272 | 0.1559 | 0.2639 | 0.0114 | 0.0000 | 0.0962 | 0.0000 | 0.0000 | 0.0040 |
| TCGA-BH-A18V-01 | 0.0008 | 0.0025 | 0.0060 | 0.0000 | 0.0000 | 0.0844 | 0.0000 | 0.0801 | 0.0335 | 0.0000 | 0.0187 | 0.0077 | 0.0000 | 0.4793 | 0.1001 | 0.1581 | 0.0000 | 0.0000 | 0.0288 | 0.0000 | 0.0000 | 0.0000 |
| TCGA-BH-A1EN-01 | 0.0000 | 0.0000 | 0.0604 | 0.0000 | 0.0000 | 0.2827 | 0.0196 | 0.0150 | 0.0137 | 0.0000 | 0.0401 | 0.0058 | 0.0601 | 0.0032 | 0.0000 | 0.3304 | 0.0037 | 0.0000 | 0.1468 | 0.0000 | 0.0184 | 0.0000 |
| TCGA-BH-A1EO-01 | 0.0471 | 0.0000 | 0.0013 | 0.0000 | 0.0000 | 0.1870 | 0.0000 | 0.0271 | 0.0004 | 0.0111 | 0.0048 | 0.0000 | 0.0000 | 0.1932 | 0.0800 | 0.2874 | 0.0000 | 0.0000 | 0.1606 | 0.0000 | 0.0000 | 0.0000 |
| TCGA-BH-A1ES-01 | 0.0937 | 0.0000 | 0.0246 | 0.0000 | 0.0000 | 0.1026 | 0.0000 | 0.0763 | 0.0105 | 0.0000 | 0.0130 | 0.0199 | 0.0000 | 0.2766 | 0.0000 | 0.3033 | 0.0000 | 0.0305 | 0.0491 | 0.0000 | 0.0000 | 0.0000 |
| TCGA-BH-A1ET-01 | 0.1079 | 0.0000 | 0.1376 | 0.0938 | 0.0000 | 0.1380 | 0.0000 | 0.0325 | 0.0000 | 0.0000 | 0.0482 | 0.0000 | 0.0205 | 0.0000 | 0.0627 | 0.3199 | 0.0000 | 0.0000 | 0.0390 | 0.0000 | 0.0000 | 0.0000 |
| TCGA-BH-A1EU-01 | 0.1362 | 0.0332 | 0.0000 | 0.0227 | 0.0000 | 0.2299 | 0.0000 | 0.0702 | 0.0043 | 0.0000 | 0.0000 | 0.0000 | 0.0000 | 0.1003 | 0.0391 | 0.2592 | 0.0000 | 0.0179 | 0.0870 | 0.0000 | 0.0000 | 0.0000 |
| TCGA-BH-A1EV-01 | 0.0000 | 0.0038 | 0.0305 | 0.0000 | 0.0000 | 0.3156 | 0.0000 | 0.0445 | 0.0327 | 0.0000 | 0.0255 | 0.0205 | 0.0000 | 0.3068 | 0.0000 | 0.1733 | 0.0000 | 0.0025 | 0.0440 | 0.0000 | 0.0003 | 0.0000 |
| TCGA-BH-A1EW-01 | 0.1001 | 0.0000 | 0.0056 | 0.0364 | 0.0000 | 0.2239 | 0.0146 | 0.0361 | 0.0014 | 0.0000 | 0.0283 | 0.0000 | 0.0651 | 0.0005 | 0.0920 | 0.1965 | 0.0000 | 0.0285 | 0.1687 | 0.0000 | 0.0000 | 0.0022 |
| TCGA-BH-A1EX-01 | 0.0563 | 0.0000 | 0.0016 | 0.0251 | 0.0000 | 0.1435 | 0.0000 | 0.0797 | 0.0197 | 0.0000 | 0.0000 | 0.0000 | 0.0000 | 0.2173 | 0.0221 | 0.3960 | 0.0000 | 0.0283 | 0.0045 | 0.0000 | 0.0000 | 0.0061 |
| TCGA-BH-A1EY-01 | 0.0549 | 0.0000 | 0.0023 | 0.0000 | 0.0000 | 0.1940 | 0.0000 | 0.0825 | 0.0289 | 0.0000 | 0.0332 | 0.0000 | 0.0000 | 0.1525 | 0.0668 | 0.2657 | 0.0177 | 0.0054 | 0.0960 | 0.0000 | 0.0000 | 0.0000 |
| TCGA-BH-A1F0-01 | 0.0445 | 0.0173 | 0.0502 | 0.0360 | 0.0000 | 0.2051 | 0.0023 | 0.0831 | 0.0000 | 0.0000 | 0.0253 | 0.0000 | 0.0267 | 0.0681 | 0.1315 | 0.2844 | 0.0000 | 0.0025 | 0.0230 | 0.0000 | 0.0000 | 0.0000 |
| TCGA-BH-A1F2-01 | 0.0017 | 0.0044 | 0.0039 | 0.0000 | 0.0000 | 0.1916 | 0.0000 | 0.0431 | 0.0358 | 0.0000 | 0.0469 | 0.0197 | 0.0017 | 0.3734 | 0.0000 | 0.1844 | 0.0000 | 0.0000 | 0.0934 | 0.0000 | 0.0000 | 0.0000 |
| TCGA-BH-A1F5-01 | 0.0162 | 0.0000 | 0.0895 | 0.0000 | 0.0000 | 0.1130 | 0.0000 | 0.0961 | 0.0000 | 0.0000 | 0.0106 | 0.0139 | 0.0054 | 0.0000 | 0.0270 | 0.3628 | 0.0725 | 0.0131 | 0.1733 | 0.0067 | 0.0000 | 0.0000 |
| TCGA-BH-A1F6-01 | 0.0500 | 0.0000 | 0.0460 | 0.0274 | 0.0000 | 0.1337 | 0.0075 | 0.0468 | 0.0246 | 0.0000 | 0.0442 | 0.0000 | 0.0102 | 0.2946 | 0.0910 | 0.2096 | 0.0000 | 0.0000 | 0.0144 | 0.0000 | 0.0000 | 0.0000 |
| TCGA-BH-A1F8-01 | 0.1150 | 0.0000 | 0.0995 | 0.0478 | 0.0000 | 0.0548 | 0.0000 | 0.0599 | 0.0000 | 0.0172 | 0.0000 | 0.0247 | 0.0000 | 0.0711 | 0.0333 | 0.2867 | 0.0000 | 0.0943 | 0.0956 | 0.0000 | 0.0000 | 0.0000 |
| TCGA-BH-A1FB-01 | 0.1584 | 0.0000 | 0.0285 | 0.0641 | 0.0000 | 0.1676 | 0.0144 | 0.1127 | 0.0000 | 0.0000 | 0.0070 | 0.0000 | 0.0076 | 0.0000 | 0.0767 | 0.2178 | 0.0307 | 0.0014 | 0.1129 | 0.0000 | 0.0000 | 0.0000 |
| TCGA-BH-A1FC-01 | 0.0522 | 0.0000 | 0.1582 | 0.1106 | 0.0000 | 0.0189 | 0.0656 | 0.2154 | 0.0430 | 0.0000 | 0.0464 | 0.0000 | 0.0133 | 0.0459 | 0.1076 | 0.0750 | 0.0000 | 0.0073 | 0.0407 | 0.0000 | 0.0000 | 0.0000 |
| TCGA-BH-A1FD-01 | 0.0000 | 0.0000 | 0.0009 | 0.0000 | 0.0000 | 0.2797 | 0.0000 | 0.1149 | 0.0431 | 0.0000 | 0.0000 | 0.0000 | 0.0178 | 0.2432 | 0.0366 | 0.1950 | 0.0000 | 0.0072 | 0.0550 | 0.0000 | 0.0065 | 0.0000 |
| TCGA-BH-A1FE-01 | 0.1764 | 0.0000 | 0.0337 | 0.0000 | 0.0000 | 0.1651 | 0.0036 | 0.1142 | 0.0138 | 0.0000 | 0.0439 | 0.0000 | 0.0162 | 0.0332 | 0.1472 | 0.0821 | 0.0000 | 0.0116 | 0.1591 | 0.0000 | 0.0000 | 0.0000 |
| TCGA-BH-A1FG-01 | 0.0630 | 0.0000 | 0.0412 | 0.0413 | 0.0000 | 0.1471 | 0.0000 | 0.0536 | 0.0000 | 0.0000 | 0.0000 | 0.0000 | 0.0358 | 0.0000 | 0.0790 | 0.3490 | 0.0026 | 0.0000 | 0.1850 | 0.0000 | 0.0000 | 0.0024 |
| TCGA-BH-A1FH-01 | 0.0088 | 0.0000 | 0.0736 | 0.0264 | 0.0000 | 0.3231 | 0.0000 | 0.0933 | 0.0000 | 0.0000 | 0.0000 | 0.0000 | 0.0592 | 0.0304 | 0.0507 | 0.1409 | 0.0019 | 0.0000 | 0.1916 | 0.0000 | 0.0000 | 0.0000 |
| TCGA-BH-A1FJ-01 | 0.0481 | 0.0000 | 0.0125 | 0.0000 | 0.0000 | 0.0719 | 0.0000 | 0.0768 | 0.0136 | 0.0000 | 0.0000 | 0.0043 | 0.0000 | 0.1345 | 0.0000 | 0.3893 | 0.0000 | 0.1769 | 0.0715 | 0.0000 | 0.0000 | 0.0005 |
| TCGA-BH-A1FL-01 | 0.1142 | 0.0000 | 0.0328 | 0.0000 | 0.0000 | 0.1561 | 0.0000 | 0.1888 | 0.0395 | 0.0000 | 0.0722 | 0.0000 | 0.0430 | 0.0648 | 0.0377 | 0.0604 | 0.0000 | 0.0000 | 0.1906 | 0.0000 | 0.0000 | 0.0000 |
| TCGA-BH-A1FM-01 | 0.0029 | 0.0031 | 0.0061 | 0.0000 | 0.0000 | 0.0737 | 0.0000 | 0.0231 | 0.0437 | 0.0166 | 0.0213 | 0.0000 | 0.0000 | 0.3197 | 0.0022 | 0.3314 | 0.0000 | 0.0000 | 0.1310 | 0.0000 | 0.0000 | 0.0251 |
| TCGA-BH-A1FN-01 | 0.1115 | 0.0000 | 0.0416 | 0.0000 | 0.0000 | 0.0671 | 0.0000 | 0.0507 | 0.0405 | 0.0000 | 0.0211 | 0.0000 | 0.0000 | 0.4874 | 0.0550 | 0.1004 | 0.0000 | 0.0000 | 0.0000 | 0.0247 | 0.0000 | 0.0000 |
| TCGA-BH-A1FR-01 | 0.1143 | 0.0000 | 0.0138 | 0.0410 | 0.0000 | 0.1107 | 0.0000 | 0.0501 | 0.0223 | 0.0000 | 0.0119 | 0.0000 | 0.0000 | 0.2524 | 0.0620 | 0.3039 | 0.0000 | 0.0000 | 0.0175 | 0.0000 | 0.0000 | 0.0000 |
| TCGA-BH-A1FU-01 | 0.0121 | 0.0932 | 0.0000 | 0.0049 | 0.0000 | 0.1510 | 0.0000 | 0.0865 | 0.0242 | 0.0000 | 0.0343 | 0.0000 | 0.0092 | 0.2972 | 0.0825 | 0.1636 | 0.0000 | 0.0000 | 0.0413 | 0.0000 | 0.0000 | 0.0000 |
| TCGA-BH-A201-01 | 0.0007 | 0.0037 | 0.0070 | 0.0000 | 0.0000 | 0.2021 | 0.0000 | 0.0931 | 0.0409 | 0.0000 | 0.0388 | 0.0376 | 0.0000 | 0.4858 | 0.0132 | 0.0139 | 0.0000 | 0.0000 | 0.0632 | 0.0000 | 0.0000 | 0.0000 |
| TCGA-BH-A202-01 | 0.0796 | 0.0000 | 0.0384 | 0.0539 | 0.0000 | 0.1297 | 0.0267 | 0.0747 | 0.0320 | 0.0000 | 0.0278 | 0.0000 | 0.0000 | 0.1482 | 0.0808 | 0.2384 | 0.0121 | 0.0000 | 0.0577 | 0.0000 | 0.0000 | 0.0000 |
| TCGA-BH-A203-01 | 0.0395 | 0.0000 | 0.0792 | 0.0662 | 0.0000 | 0.0863 | 0.0037 | 0.1315 | 0.0332 | 0.0000 | 0.0068 | 0.0000 | 0.0085 | 0.1111 | 0.0858 | 0.2942 | 0.0000 | 0.0210 | 0.0330 | 0.0000 | 0.0000 | 0.0000 |
| TCGA-BH-A204-01 | 0.0000 | 0.0000 | 0.0619 | 0.0000 | 0.0000 | 0.3833 | 0.0000 | 0.0467 | 0.0162 | 0.0000 | 0.0699 | 0.0000 | 0.0392 | 0.0177 | 0.0000 | 0.2452 | 0.0000 | 0.0000 | 0.1201 | 0.0000 | 0.0000 | 0.0000 |
| TCGA-BH-A208-01 | 0.1030 | 0.0000 | 0.1648 | 0.0657 | 0.0000 | 0.1246 | 0.0000 | 0.0172 | 0.0078 | 0.0000 | 0.0067 | 0.0000 | 0.0000 | 0.0148 | 0.0062 | 0.3515 | 0.0000 | 0.0962 | 0.0415 | 0.0000 | 0.0000 | 0.0000 |
| TCGA-BH-A209-01 | 0.1125 | 0.0000 | 0.0089 | 0.1116 | 0.0000 | 0.1652 | 0.0543 | 0.0741 | 0.0233 | 0.0023 | 0.0280 | 0.0000 | 0.0000 | 0.0592 | 0.1302 | 0.1576 | 0.0000 | 0.0232 | 0.0497 | 0.0000 | 0.0000 | 0.0000 |
| TCGA-BH-A28O-01 | 0.0998 | 0.0000 | 0.1164 | 0.0852 | 0.0000 | 0.2669 | 0.0000 | 0.0579 | 0.0195 | 0.0000 | 0.0306 | 0.0000 | 0.0068 | 0.0000 | 0.0458 | 0.2290 | 0.0000 | 0.0000 | 0.0421 | 0.0000 | 0.0000 | 0.0000 |
| TCGA-BH-A28Q-01 | 0.0359 | 0.0000 | 0.0445 | 0.0307 | 0.0000 | 0.2322 | 0.0000 | 0.0987 | 0.0000 | 0.0000 | 0.0406 | 0.0000 | 0.0221 | 0.0000 | 0.0700 | 0.3285 | 0.0000 | 0.0000 | 0.0969 | 0.0000 | 0.0000 | 0.0000 |
| TCGA-BH-A2L8-01 | 0.0828 | 0.0000 | 0.0184 | 0.2204 | 0.0000 | 0.1173 | 0.0205 | 0.0944 | 0.0237 | 0.0000 | 0.0000 | 0.0111 | 0.0170 | 0.0151 | 0.0974 | 0.2204 | 0.0011 | 0.0000 | 0.0605 | 0.0000 | 0.0000 | 0.0000 |
| TCGA-BH-A42T-01 | 0.0943 | 0.0000 | 0.0000 | 0.0506 | 0.0000 | 0.0971 | 0.0000 | 0.1530 | 0.0175 | 0.0000 | 0.0000 | 0.0135 | 0.0519 | 0.0000 | 0.0279 | 0.3210 | 0.0018 | 0.0070 | 0.0000 | 0.1644 | 0.0000 | 0.0000 |
| TCGA-BH-A42U-01 | 0.0600 | 0.0732 | 0.0214 | 0.0962 | 0.0000 | 0.2057 | 0.0000 | 0.0728 | 0.0198 | 0.0000 | 0.0085 | 0.0065 | 0.0031 | 0.1964 | 0.0395 | 0.1761 | 0.0000 | 0.0000 | 0.0000 | 0.0210 | 0.0000 | 0.0000 |
| TCGA-BH-A42V-01 | 0.1089 | 0.0000 | 0.0000 | 0.1048 | 0.0000 | 0.1214 | 0.0000 | 0.1630 | 0.0096 | 0.0000 | 0.0000 | 0.0177 | 0.0205 | 0.1081 | 0.0436 | 0.2195 | 0.0000 | 0.0000 | 0.0829 | 0.0000 | 0.0000 | 0.0000 |
| TCGA-BH-A5IZ-01 | 0.0036 | 0.0027 | 0.0049 | 0.0000 | 0.0000 | 0.1706 | 0.0000 | 0.0958 | 0.0215 | 0.0000 | 0.0176 | 0.0086 | 0.0000 | 0.4494 | 0.0056 | 0.1737 | 0.0000 | 0.0000 | 0.0396 | 0.0000 | 0.0065 | 0.0000 |
| TCGA-BH-A5J0-01 | 0.0227 | 0.0000 | 0.0036 | 0.0477 | 0.0000 | 0.1877 | 0.0000 | 0.0379 | 0.0494 | 0.0000 | 0.0301 | 0.0000 | 0.0156 | 0.2315 | 0.0712 | 0.2558 | 0.0000 | 0.0000 | 0.0469 | 0.0000 | 0.0000 | 0.0000 |
| TCGA-BH-A6R8-01 | 0.0574 | 0.0000 | 0.0085 | 0.0465 | 0.0000 | 0.0666 | 0.0000 | 0.0958 | 0.0882 | 0.0000 | 0.0402 | 0.0000 | 0.0000 | 0.2269 | 0.0708 | 0.2583 | 0.0000 | 0.0000 | 0.0408 | 0.0000 | 0.0000 | 0.0000 |
| TCGA-BH-A6R9-01 | 0.0063 | 0.0000 | 0.0000 | 0.0183 | 0.0000 | 0.0579 | 0.0008 | 0.0396 | 0.0377 | 0.0000 | 0.0214 | 0.0012 | 0.0000 | 0.2643 | 0.0894 | 0.3569 | 0.0264 | 0.0000 | 0.0799 | 0.0000 | 0.0000 | 0.0000 |
| TCGA-BH-A8FY-01 | 0.0000 | 0.0341 | 0.0000 | 0.0000 | 0.0000 | 0.2021 | 0.0000 | 0.1191 | 0.0000 | 0.0000 | 0.0843 | 0.0155 | 0.0718 | 0.0000 | 0.0000 | 0.3188 | 0.0116 | 0.0000 | 0.1426 | 0.0000 | 0.0001 | 0.0000 |
| TCGA-BH-A8FZ-01 | 0.0926 | 0.0000 | 0.0114 | 0.0188 | 0.0000 | 0.1698 | 0.0047 | 0.0608 | 0.0205 | 0.0000 | 0.0296 | 0.0000 | 0.0215 | 0.2140 | 0.0215 | 0.1547 | 0.0332 | 0.0469 | 0.0999 | 0.0001 | 0.0000 | 0.0000 |
| TCGA-BH-A8G0-01 | 0.0729 | 0.0706 | 0.0000 | 0.0152 | 0.0000 | 0.0258 | 0.0000 | 0.0800 | 0.0383 | 0.0000 | 0.0553 | 0.0000 | 0.0000 | 0.4787 | 0.0234 | 0.1217 | 0.0000 | 0.0000 | 0.0180 | 0.0000 | 0.0000 | 0.0000 |
| TCGA-BH-AB28-01 | 0.2049 | 0.0000 | 0.0000 | 0.0650 | 0.0000 | 0.1160 | 0.0000 | 0.1147 | 0.0411 | 0.0000 | 0.0195 | 0.0000 | 0.0311 | 0.0000 | 0.0554 | 0.3107 | 0.0000 | 0.0139 | 0.0277 | 0.0000 | 0.0000 | 0.0000 |
| TCGA-C8-A12K-01 | 0.0046 | 0.0000 | 0.0121 | 0.0966 | 0.0000 | 0.1120 | 0.0000 | 0.0350 | 0.0584 | 0.0000 | 0.0000 | 0.0589 | 0.0034 | 0.1791 | 0.2601 | 0.1466 | 0.0000 | 0.0000 | 0.0330 | 0.0000 | 0.0000 | 0.0003 |
| TCGA-C8-A12L-01 | 0.0305 | 0.0000 | 0.0582 | 0.0000 | 0.0000 | 0.1618 | 0.0000 | 0.1624 | 0.0041 | 0.0000 | 0.0405 | 0.0277 | 0.0138 | 0.2178 | 0.0827 | 0.1181 | 0.0070 | 0.0000 | 0.0753 | 0.0000 | 0.0000 | 0.0000 |
| TCGA-C8-A12M-01 | 0.1161 | 0.0000 | 0.0807 | 0.0916 | 0.0000 | 0.1789 | 0.0000 | 0.0953 | 0.0490 | 0.0000 | 0.0276 | 0.0000 | 0.0000 | 0.0415 | 0.0508 | 0.1467 | 0.0000 | 0.0000 | 0.1219 | 0.0000 | 0.0000 | 0.0000 |
| TCGA-C8-A12N-01 | 0.1000 | 0.0000 | 0.0292 | 0.0284 | 0.0000 | 0.0441 | 0.0000 | 0.0717 | 0.0114 | 0.0000 | 0.0001 | 0.0185 | 0.0372 | 0.0000 | 0.0558 | 0.2968 | 0.1432 | 0.0041 | 0.1541 | 0.0000 | 0.0000 | 0.0055 |
| TCGA-C8-A12O-01 | 0.0585 | 0.0000 | 0.0400 | 0.0291 | 0.0000 | 0.0727 | 0.0000 | 0.0613 | 0.0278 | 0.0000 | 0.0231 | 0.0000 | 0.0000 | 0.3819 | 0.0576 | 0.1757 | 0.0000 | 0.0000 | 0.0725 | 0.0000 | 0.0000 | 0.0000 |
| TCGA-C8-A12P-01 | 0.0209 | 0.0000 | 0.0550 | 0.0000 | 0.0000 | 0.1035 | 0.0000 | 0.0631 | 0.0204 | 0.0037 | 0.0000 | 0.0241 | 0.0000 | 0.1754 | 0.1100 | 0.3137 | 0.0000 | 0.0186 | 0.0917 | 0.0000 | 0.0000 | 0.0000 |
| TCGA-C8-A12Q-01 | 0.0328 | 0.0000 | 0.0016 | 0.0000 | 0.0000 | 0.1217 | 0.0000 | 0.0636 | 0.0231 | 0.0000 | 0.0053 | 0.0094 | 0.0000 | 0.4060 | 0.0764 | 0.1977 | 0.0000 | 0.0000 | 0.0624 | 0.0000 | 0.0000 | 0.0000 |
| TCGA-C8-A12T-01 | 0.0097 | 0.0000 | 0.0119 | 0.0594 | 0.0000 | 0.0823 | 0.0000 | 0.0859 | 0.0312 | 0.0000 | 0.0046 | 0.0276 | 0.0086 | 0.0813 | 0.0630 | 0.3290 | 0.0000 | 0.0366 | 0.1689 | 0.0000 | 0.0000 | 0.0000 |
| TCGA-C8-A12U-01 | 0.0371 | 0.0000 | 0.0159 | 0.2147 | 0.0000 | 0.0714 | 0.0242 | 0.1440 | 0.0315 | 0.0000 | 0.0640 | 0.0000 | 0.0318 | 0.0738 | 0.0768 | 0.1211 | 0.0010 | 0.0000 | 0.0925 | 0.0000 | 0.0000 | 0.0000 |
| TCGA-C8-A12V-01 | 0.0039 | 0.0308 | 0.0422 | 0.0947 | 0.0000 | 0.1507 | 0.0178 | 0.0957 | 0.0549 | 0.0000 | 0.0735 | 0.0000 | 0.0028 | 0.1418 | 0.1587 | 0.1114 | 0.0046 | 0.0000 | 0.0166 | 0.0000 | 0.0000 | 0.0000 |
| TCGA-C8-A12W-01 | 0.0467 | 0.0000 | 0.1761 | 0.0270 | 0.0000 | 0.0911 | 0.0000 | 0.0090 | 0.0236 | 0.0000 | 0.0247 | 0.0000 | 0.0000 | 0.3302 | 0.0370 | 0.2119 | 0.0000 | 0.0000 | 0.0229 | 0.0000 | 0.0000 | 0.0000 |
| TCGA-C8-A12X-01 | 0.0613 | 0.0000 | 0.0001 | 0.0477 | 0.0000 | 0.1046 | 0.0000 | 0.0178 | 0.0296 | 0.0000 | 0.0297 | 0.0000 | 0.0000 | 0.2583 | 0.0271 | 0.3592 | 0.0000 | 0.0000 | 0.0344 | 0.0000 | 0.0000 | 0.0302 |
| TCGA-C8-A12Y-01 | 0.0630 | 0.0000 | 0.1299 | 0.0554 | 0.0000 | 0.0616 | 0.0000 | 0.0626 | 0.0370 | 0.0000 | 0.0000 | 0.0509 | 0.0036 | 0.0573 | 0.0899 | 0.2373 | 0.0000 | 0.0016 | 0.1496 | 0.0000 | 0.0000 | 0.0003 |
| TCGA-C8-A12Z-01 | 0.0117 | 0.0000 | 0.0090 | 0.0000 | 0.0000 | 0.2593 | 0.0000 | 0.0775 | 0.0453 | 0.0000 | 0.0451 | 0.0000 | 0.0000 | 0.3886 | 0.0137 | 0.0838 | 0.0000 | 0.0000 | 0.0643 | 0.0000 | 0.0000 | 0.0016 |
| TCGA-C8-A130-01 | 0.1215 | 0.0000 | 0.0522 | 0.0787 | 0.0000 | 0.1919 | 0.0000 | 0.0792 | 0.0199 | 0.0000 | 0.0228 | 0.0000 | 0.0000 | 0.0484 | 0.0773 | 0.2310 | 0.0000 | 0.0000 | 0.0771 | 0.0000 | 0.0000 | 0.0000 |
| TCGA-C8-A131-01 | 0.0000 | 0.0000 | 0.0193 | 0.0427 | 0.0000 | 0.1709 | 0.0000 | 0.0329 | 0.0246 | 0.0000 | 0.0204 | 0.0044 | 0.0000 | 0.4557 | 0.0346 | 0.1888 | 0.0000 | 0.0000 | 0.0058 | 0.0000 | 0.0000 | 0.0000 |
| TCGA-C8-A132-01 | 0.0902 | 0.0000 | 0.0498 | 0.1090 | 0.0000 | 0.0586 | 0.0028 | 0.1297 | 0.0663 | 0.0000 | 0.0136 | 0.0000 | 0.0182 | 0.2457 | 0.0807 | 0.1159 | 0.0000 | 0.0000 | 0.0195 | 0.0000 | 0.0000 | 0.0000 |
| TCGA-C8-A133-01 | 0.0006 | 0.0025 | 0.0000 | 0.0000 | 0.0000 | 0.2721 | 0.0000 | 0.0281 | 0.0793 | 0.0000 | 0.0032 | 0.0000 | 0.0261 | 0.0809 | 0.0265 | 0.2110 | 0.0000 | 0.0000 | 0.2696 | 0.0000 | 0.0000 | 0.0000 |
| TCGA-C8-A134-01 | 0.0050 | 0.0290 | 0.0000 | 0.0000 | 0.0000 | 0.0591 | 0.0003 | 0.1025 | 0.0076 | 0.0542 | 0.0282 | 0.0000 | 0.0000 | 0.4286 | 0.1692 | 0.1017 | 0.0000 | 0.0000 | 0.0147 | 0.0000 | 0.0000 | 0.0000 |
| TCGA-C8-A135-01 | 0.1178 | 0.0000 | 0.0591 | 0.1063 | 0.0000 | 0.2517 | 0.0045 | 0.0422 | 0.0109 | 0.0000 | 0.0000 | 0.0000 | 0.0081 | 0.0069 | 0.0492 | 0.3104 | 0.0000 | 0.0000 | 0.0328 | 0.0000 | 0.0000 | 0.0000 |
| TCGA-C8-A137-01 | 0.0000 | 0.0000 | 0.0371 | 0.0000 | 0.0000 | 0.3008 | 0.0000 | 0.0709 | 0.0557 | 0.0000 | 0.0246 | 0.0083 | 0.0000 | 0.3305 | 0.0367 | 0.0749 | 0.0000 | 0.0000 | 0.0520 | 0.0000 | 0.0085 | 0.0000 |
| TCGA-C8-A138-01 | 0.0721 | 0.0000 | 0.0633 | 0.1142 | 0.0000 | 0.1479 | 0.0093 | 0.0651 | 0.0243 | 0.0000 | 0.0061 | 0.0000 | 0.0000 | 0.2487 | 0.0376 | 0.1485 | 0.0000 | 0.0214 | 0.0415 | 0.0000 | 0.0000 | 0.0000 |
| TCGA-C8-A1HE-01 | 0.1041 | 0.0000 | 0.0298 | 0.0216 | 0.0000 | 0.1659 | 0.0000 | 0.0686 | 0.0000 | 0.0000 | 0.0204 | 0.0000 | 0.0400 | 0.0000 | 0.0674 | 0.4249 | 0.0109 | 0.0000 | 0.0321 | 0.0000 | 0.0000 | 0.0143 |
| TCGA-C8-A1HF-01 | 0.0202 | 0.0000 | 0.0677 | 0.1341 | 0.0000 | 0.1611 | 0.0045 | 0.1433 | 0.0500 | 0.0057 | 0.0000 | 0.0105 | 0.0000 | 0.0034 | 0.0911 | 0.2978 | 0.0000 | 0.0004 | 0.0103 | 0.0000 | 0.0000 | 0.0000 |
| TCGA-C8-A1HG-01 | 0.0503 | 0.0000 | 0.1583 | 0.1453 | 0.0000 | 0.0915 | 0.0046 | 0.0910 | 0.0632 | 0.0000 | 0.0000 | 0.0205 | 0.0000 | 0.0054 | 0.0693 | 0.2465 | 0.0037 | 0.0064 | 0.0439 | 0.0000 | 0.0000 | 0.0000 |
| TCGA-C8-A1HI-01 | 0.0769 | 0.0000 | 0.1145 | 0.0000 | 0.0000 | 0.0759 | 0.0000 | 0.0431 | 0.0079 | 0.0262 | 0.0000 | 0.0143 | 0.0000 | 0.0000 | 0.0823 | 0.4191 | 0.0000 | 0.0049 | 0.1349 | 0.0000 | 0.0000 | 0.0000 |
| TCGA-C8-A1HJ-01 | 0.1457 | 0.0000 | 0.0546 | 0.0481 | 0.0000 | 0.1191 | 0.0446 | 0.1290 | 0.0090 | 0.0098 | 0.0497 | 0.0000 | 0.0000 | 0.0705 | 0.1192 | 0.1362 | 0.0000 | 0.0602 | 0.0042 | 0.0000 | 0.0000 | 0.0000 |
| TCGA-C8-A1HK-01 | 0.1157 | 0.0000 | 0.1701 | 0.0665 | 0.0000 | 0.1522 | 0.0000 | 0.0893 | 0.0306 | 0.0000 | 0.0000 | 0.0043 | 0.0000 | 0.0318 | 0.0549 | 0.2575 | 0.0000 | 0.0138 | 0.0133 | 0.0000 | 0.0000 | 0.0000 |
| TCGA-C8-A1HL-01 | 0.0009 | 0.0016 | 0.0051 | 0.0000 | 0.0000 | 0.1684 | 0.0000 | 0.0122 | 0.0255 | 0.0000 | 0.0344 | 0.0000 | 0.0000 | 0.4122 | 0.0119 | 0.2491 | 0.0000 | 0.0000 | 0.0788 | 0.0000 | 0.0000 | 0.0000 |
| TCGA-C8-A1HM-01 | 0.0408 | 0.0000 | 0.0221 | 0.1530 | 0.0000 | 0.0698 | 0.0842 | 0.1355 | 0.0004 | 0.0000 | 0.0049 | 0.0677 | 0.0029 | 0.0104 | 0.1474 | 0.1680 | 0.0000 | 0.0008 | 0.0920 | 0.0000 | 0.0000 | 0.0000 |
| TCGA-C8-A1HN-01 | 0.0017 | 0.0059 | 0.0008 | 0.0000 | 0.0000 | 0.1564 | 0.0000 | 0.0635 | 0.0660 | 0.0000 | 0.0543 | 0.0000 | 0.0000 | 0.3556 | 0.0038 | 0.2528 | 0.0005 | 0.0000 | 0.0388 | 0.0000 | 0.0000 | 0.0000 |
| TCGA-C8-A1HO-01 | 0.0954 | 0.0000 | 0.1771 | 0.0366 | 0.0000 | 0.1058 | 0.0000 | 0.0386 | 0.0273 | 0.0000 | 0.0099 | 0.0000 | 0.0000 | 0.1439 | 0.0920 | 0.2191 | 0.0000 | 0.0000 | 0.0544 | 0.0000 | 0.0000 | 0.0000 |
| TCGA-C8-A26V-01 | 0.0609 | 0.0000 | 0.0255 | 0.0638 | 0.0000 | 0.1145 | 0.0030 | 0.0681 | 0.0198 | 0.0000 | 0.0365 | 0.0032 | 0.0000 | 0.1325 | 0.1021 | 0.2859 | 0.0016 | 0.0478 | 0.0347 | 0.0000 | 0.0000 | 0.0000 |
| TCGA-C8-A26W-01 | 0.1384 | 0.0000 | 0.0424 | 0.1087 | 0.0000 | 0.1972 | 0.0057 | 0.0736 | 0.0220 | 0.0000 | 0.0232 | 0.0000 | 0.0194 | 0.0000 | 0.0875 | 0.2625 | 0.0000 | 0.0000 | 0.0195 | 0.0000 | 0.0000 | 0.0000 |
| TCGA-C8-A26X-01 | 0.0453 | 0.0000 | 0.0731 | 0.0000 | 0.0000 | 0.0837 | 0.0000 | 0.1045 | 0.0146 | 0.0091 | 0.0076 | 0.0248 | 0.0000 | 0.2901 | 0.0808 | 0.2153 | 0.0000 | 0.0000 | 0.0511 | 0.0000 | 0.0000 | 0.0000 |
| TCGA-C8-A26Y-01 | 0.1064 | 0.0000 | 0.0000 | 0.0265 | 0.0000 | 0.0234 | 0.0000 | 0.1153 | 0.0409 | 0.0000 | 0.0291 | 0.0000 | 0.0000 | 0.4197 | 0.0267 | 0.2116 | 0.0000 | 0.0004 | 0.0000 | 0.0000 | 0.0000 | 0.0000 |
| TCGA-C8-A26Z-01 | 0.0783 | 0.0000 | 0.0713 | 0.0753 | 0.0000 | 0.0393 | 0.0000 | 0.0577 | 0.0000 | 0.0000 | 0.0382 | 0.0038 | 0.0178 | 0.0000 | 0.0587 | 0.4356 | 0.0104 | 0.0000 | 0.1137 | 0.0000 | 0.0000 | 0.0000 |
| TCGA-C8-A273-01 | 0.0745 | 0.0000 | 0.0217 | 0.0196 | 0.0000 | 0.1099 | 0.0000 | 0.0424 | 0.0257 | 0.0000 | 0.0164 | 0.0000 | 0.0000 | 0.3582 | 0.0336 | 0.2627 | 0.0000 | 0.0083 | 0.0268 | 0.0000 | 0.0000 | 0.0000 |
| TCGA-C8-A274-01 | 0.0920 | 0.0000 | 0.0386 | 0.0071 | 0.0000 | 0.1080 | 0.0000 | 0.0528 | 0.0160 | 0.0000 | 0.0367 | 0.0000 | 0.0281 | 0.0000 | 0.1048 | 0.4205 | 0.0086 | 0.0000 | 0.0717 | 0.0000 | 0.0000 | 0.0152 |
| TCGA-C8-A275-01 | 0.0493 | 0.0000 | 0.0035 | 0.0000 | 0.0000 | 0.2095 | 0.0105 | 0.1077 | 0.0040 | 0.0060 | 0.0000 | 0.0207 | 0.0000 | 0.1490 | 0.1148 | 0.1796 | 0.0000 | 0.0266 | 0.1182 | 0.0000 | 0.0000 | 0.0006 |
| TCGA-C8-A278-01 | 0.0364 | 0.0000 | 0.1255 | 0.0328 | 0.0000 | 0.1127 | 0.0066 | 0.0478 | 0.0245 | 0.0000 | 0.0179 | 0.0000 | 0.0000 | 0.1239 | 0.0773 | 0.2806 | 0.0000 | 0.0082 | 0.1046 | 0.0000 | 0.0000 | 0.0011 |
| TCGA-C8-A27A-01 | 0.1087 | 0.0000 | 0.1958 | 0.0000 | 0.0000 | 0.0700 | 0.0000 | 0.0344 | 0.0281 | 0.0065 | 0.0000 | 0.0083 | 0.0000 | 0.1988 | 0.1172 | 0.2182 | 0.0000 | 0.0000 | 0.0141 | 0.0000 | 0.0000 | 0.0000 |
| TCGA-C8-A27B-01 | 0.0056 | 0.0000 | 0.0219 | 0.0000 | 0.0000 | 0.0323 | 0.0000 | 0.0923 | 0.0084 | 0.0645 | 0.0000 | 0.0312 | 0.0000 | 0.4251 | 0.1823 | 0.0884 | 0.0000 | 0.0000 | 0.0434 | 0.0000 | 0.0000 | 0.0046 |
| TCGA-C8-A3M7-01 | 0.0927 | 0.0000 | 0.1682 | 0.0692 | 0.0000 | 0.1752 | 0.0000 | 0.0837 | 0.0000 | 0.0000 | 0.0000 | 0.0004 | 0.0019 | 0.0000 | 0.0568 | 0.1984 | 0.0000 | 0.0103 | 0.1431 | 0.0000 | 0.0000 | 0.0000 |
| TCGA-C8-A3M8-01 | 0.0965 | 0.0000 | 0.0666 | 0.0000 | 0.0000 | 0.0315 | 0.0000 | 0.0668 | 0.0382 | 0.0000 | 0.0299 | 0.0000 | 0.0000 | 0.3493 | 0.0410 | 0.2427 | 0.0000 | 0.0000 | 0.0374 | 0.0000 | 0.0000 | 0.0000 |
| TCGA-C8-A8HP-01 | 0.0267 | 0.0000 | 0.0637 | 0.1105 | 0.0000 | 0.1304 | 0.0000 | 0.1157 | 0.0465 | 0.0000 | 0.0258 | 0.0000 | 0.0065 | 0.1774 | 0.0806 | 0.1653 | 0.0000 | 0.0251 | 0.0258 | 0.0000 | 0.0000 | 0.0000 |
| TCGA-C8-A8HQ-01 | 0.0130 | 0.0032 | 0.0167 | 0.0000 | 0.0000 | 0.0899 | 0.0000 | 0.1321 | 0.0732 | 0.0000 | 0.0248 | 0.0203 | 0.0165 | 0.3595 | 0.0972 | 0.0870 | 0.0000 | 0.0000 | 0.0665 | 0.0000 | 0.0000 | 0.0000 |
| TCGA-C8-A8HR-01 | 0.0564 | 0.0000 | 0.0067 | 0.0873 | 0.0000 | 0.1805 | 0.0000 | 0.1075 | 0.0321 | 0.0000 | 0.0000 | 0.0039 | 0.0145 | 0.0000 | 0.1267 | 0.3732 | 0.0000 | 0.0000 | 0.0093 | 0.0020 | 0.0000 | 0.0000 |
| TCGA-D8-A13Y-01 | 0.0662 | 0.0000 | 0.0444 | 0.0104 | 0.0000 | 0.0000 | 0.0000 | 0.1598 | 0.0000 | 0.0000 | 0.0000 | 0.0361 | 0.0000 | 0.2648 | 0.0000 | 0.4043 | 0.0000 | 0.0119 | 0.0021 | 0.0000 | 0.0000 | 0.0000 |
| TCGA-D8-A13Z-01 | 0.0542 | 0.0000 | 0.0000 | 0.0000 | 0.0000 | 0.1097 | 0.0000 | 0.0687 | 0.0320 | 0.0000 | 0.0188 | 0.0091 | 0.0000 | 0.3837 | 0.0508 | 0.2143 | 0.0000 | 0.0387 | 0.0199 | 0.0000 | 0.0000 | 0.0000 |
| TCGA-D8-A140-01 | 0.0110 | 0.0021 | 0.2110 | 0.0602 | 0.0000 | 0.1333 | 0.0000 | 0.0877 | 0.0575 | 0.0000 | 0.0150 | 0.0000 | 0.0000 | 0.0513 | 0.0567 | 0.2583 | 0.0000 | 0.0119 | 0.0439 | 0.0000 | 0.0000 | 0.0000 |
| TCGA-D8-A141-01 | 0.0739 | 0.0000 | 0.0033 | 0.0641 | 0.0000 | 0.2102 | 0.0000 | 0.1202 | 0.0548 | 0.0000 | 0.0251 | 0.0000 | 0.0037 | 0.1479 | 0.0996 | 0.1638 | 0.0000 | 0.0000 | 0.0333 | 0.0000 | 0.0000 | 0.0000 |
| TCGA-D8-A142-01 | 0.0060 | 0.0135 | 0.1855 | 0.0355 | 0.0000 | 0.0775 | 0.0000 | 0.0815 | 0.0193 | 0.0000 | 0.0197 | 0.0000 | 0.0000 | 0.2745 | 0.0642 | 0.2028 | 0.0000 | 0.0000 | 0.0200 | 0.0000 | 0.0000 | 0.0000 |
| TCGA-D8-A143-01 | 0.1063 | 0.0000 | 0.1118 | 0.0501 | 0.0000 | 0.1549 | 0.0153 | 0.1168 | 0.0066 | 0.0000 | 0.0298 | 0.0000 | 0.0000 | 0.2049 | 0.0544 | 0.1415 | 0.0000 | 0.0030 | 0.0044 | 0.0000 | 0.0000 | 0.0000 |
| TCGA-D8-A145-01 | 0.1126 | 0.0000 | 0.2643 | 0.0359 | 0.0000 | 0.1280 | 0.0000 | 0.0730 | 0.0000 | 0.0000 | 0.0000 | 0.0045 | 0.0000 | 0.0948 | 0.0269 | 0.1836 | 0.0000 | 0.0000 | 0.0764 | 0.0000 | 0.0000 | 0.0000 |
| TCGA-D8-A146-01 | 0.1221 | 0.0000 | 0.0066 | 0.0168 | 0.0000 | 0.0503 | 0.0000 | 0.0770 | 0.0092 | 0.0000 | 0.0220 | 0.0000 | 0.0000 | 0.3402 | 0.0433 | 0.1724 | 0.0000 | 0.0000 | 0.1402 | 0.0000 | 0.0000 | 0.0000 |
| TCGA-D8-A147-01 | 0.0564 | 0.0000 | 0.0104 | 0.0202 | 0.0000 | 0.1281 | 0.0064 | 0.1068 | 0.0215 | 0.0000 | 0.0121 | 0.0000 | 0.0096 | 0.3065 | 0.1105 | 0.1605 | 0.0000 | 0.0066 | 0.0444 | 0.0000 | 0.0000 | 0.0000 |
| TCGA-D8-A1J8-01 | 0.0375 | 0.0000 | 0.0138 | 0.0467 | 0.0000 | 0.2159 | 0.0000 | 0.1168 | 0.0216 | 0.0000 | 0.0000 | 0.0225 | 0.0000 | 0.0828 | 0.1619 | 0.2104 | 0.0000 | 0.0478 | 0.0223 | 0.0000 | 0.0000 | 0.0000 |
| TCGA-D8-A1J9-01 | 0.0017 | 0.0001 | 0.0048 | 0.0000 | 0.0000 | 0.1480 | 0.0000 | 0.0453 | 0.0996 | 0.0000 | 0.0495 | 0.0189 | 0.0185 | 0.2685 | 0.0354 | 0.2121 | 0.0017 | 0.0000 | 0.0960 | 0.0000 | 0.0000 | 0.0000 |
| TCGA-D8-A1JA-01 | 0.0698 | 0.0000 | 0.1108 | 0.0096 | 0.0000 | 0.1241 | 0.0000 | 0.0369 | 0.0000 | 0.0000 | 0.0244 | 0.0000 | 0.0000 | 0.0000 | 0.0658 | 0.2294 | 0.0000 | 0.2335 | 0.0197 | 0.0000 | 0.0000 | 0.0761 |
| TCGA-D8-A1JB-01 | 0.1486 | 0.0000 | 0.0000 | 0.1641 | 0.0000 | 0.1102 | 0.0000 | 0.1041 | 0.0647 | 0.0136 | 0.0094 | 0.0000 | 0.0027 | 0.0081 | 0.1373 | 0.1765 | 0.0000 | 0.0000 | 0.0607 | 0.0000 | 0.0000 | 0.0000 |
| TCGA-D8-A1JC-01 | 0.0386 | 0.0000 | 0.0002 | 0.0459 | 0.0000 | 0.1229 | 0.0038 | 0.0606 | 0.0234 | 0.0000 | 0.0065 | 0.0021 | 0.0000 | 0.3764 | 0.0704 | 0.1924 | 0.0000 | 0.0059 | 0.0497 | 0.0000 | 0.0000 | 0.0012 |
| TCGA-D8-A1JD-01 | 0.1002 | 0.0000 | 0.0114 | 0.0195 | 0.0000 | 0.1399 | 0.0000 | 0.0691 | 0.0297 | 0.0000 | 0.0408 | 0.0000 | 0.0353 | 0.0000 | 0.0815 | 0.3366 | 0.0260 | 0.0000 | 0.0993 | 0.0000 | 0.0000 | 0.0107 |
| TCGA-D8-A1JE-01 | 0.0019 | 0.0000 | 0.0037 | 0.0000 | 0.0000 | 0.2684 | 0.0000 | 0.0031 | 0.0012 | 0.0000 | 0.0282 | 0.0000 | 0.0294 | 0.0000 | 0.0891 | 0.3982 | 0.0094 | 0.0000 | 0.1674 | 0.0000 | 0.0000 | 0.0000 |
| TCGA-D8-A1JF-01 | 0.1180 | 0.0000 | 0.2036 | 0.0134 | 0.0000 | 0.1681 | 0.0000 | 0.0477 | 0.0029 | 0.0101 | 0.0166 | 0.0000 | 0.0000 | 0.0758 | 0.0507 | 0.2488 | 0.0000 | 0.0005 | 0.0437 | 0.0000 | 0.0000 | 0.0000 |
| TCGA-D8-A1JG-01 | 0.0429 | 0.0000 | 0.1329 | 0.0054 | 0.0000 | 0.2142 | 0.0000 | 0.0516 | 0.0195 | 0.0000 | 0.0534 | 0.0000 | 0.0026 | 0.1138 | 0.1248 | 0.2017 | 0.0059 | 0.0000 | 0.0312 | 0.0000 | 0.0000 | 0.0000 |
| TCGA-D8-A1JH-01 | 0.0089 | 0.0000 | 0.0197 | 0.1276 | 0.0000 | 0.0697 | 0.0000 | 0.0703 | 0.0179 | 0.0000 | 0.0000 | 0.0001 | 0.0397 | 0.0903 | 0.0198 | 0.3235 | 0.0057 | 0.0000 | 0.2069 | 0.0000 | 0.0000 | 0.0000 |
| TCGA-D8-A1JI-01 | 0.0002 | 0.0069 | 0.0638 | 0.0000 | 0.0000 | 0.1927 | 0.0007 | 0.1059 | 0.0166 | 0.0000 | 0.1014 | 0.0000 | 0.0598 | 0.0416 | 0.0000 | 0.2402 | 0.0294 | 0.0000 | 0.1336 | 0.0000 | 0.0071 | 0.0000 |
| TCGA-D8-A1JJ-01 | 0.0001 | 0.0097 | 0.0015 | 0.0000 | 0.0000 | 0.2460 | 0.0000 | 0.0351 | 0.0758 | 0.0000 | 0.0238 | 0.0180 | 0.0000 | 0.4072 | 0.0267 | 0.0761 | 0.0000 | 0.0000 | 0.0801 | 0.0000 | 0.0000 | 0.0000 |
| TCGA-D8-A1JK-01 | 0.0404 | 0.0000 | 0.0372 | 0.0396 | 0.0000 | 0.1064 | 0.0166 | 0.0770 | 0.0408 | 0.0000 | 0.0308 | 0.0055 | 0.0000 | 0.2754 | 0.1369 | 0.1653 | 0.0000 | 0.0000 | 0.0282 | 0.0000 | 0.0000 | 0.0000 |
| TCGA-D8-A1JL-01 | 0.0473 | 0.0000 | 0.0063 | 0.0177 | 0.0000 | 0.1719 | 0.0088 | 0.0886 | 0.0381 | 0.0000 | 0.0302 | 0.0000 | 0.0000 | 0.3408 | 0.1345 | 0.0923 | 0.0000 | 0.0000 | 0.0234 | 0.0000 | 0.0000 | 0.0000 |
| TCGA-D8-A1JM-01 | 0.0426 | 0.0000 | 0.0000 | 0.2058 | 0.0000 | 0.0334 | 0.0138 | 0.2202 | 0.0295 | 0.0000 | 0.0000 | 0.0666 | 0.0000 | 0.0669 | 0.1440 | 0.0687 | 0.0000 | 0.0031 | 0.1056 | 0.0000 | 0.0000 | 0.0000 |
| TCGA-D8-A1JN-01 | 0.0669 | 0.0000 | 0.0115 | 0.0800 | 0.0000 | 0.1763 | 0.0000 | 0.1200 | 0.0275 | 0.0085 | 0.0000 | 0.0153 | 0.0477 | 0.0000 | 0.0510 | 0.2672 | 0.0000 | 0.0002 | 0.1277 | 0.0000 | 0.0000 | 0.0000 |
| TCGA-D8-A1JP-01 | 0.0645 | 0.0000 | 0.0182 | 0.0946 | 0.0000 | 0.1453 | 0.0000 | 0.0284 | 0.0403 | 0.0000 | 0.0268 | 0.0000 | 0.0059 | 0.0244 | 0.0714 | 0.4514 | 0.0000 | 0.0000 | 0.0251 | 0.0000 | 0.0000 | 0.0037 |
| TCGA-D8-A1JS-01 | 0.0005 | 0.0078 | 0.0034 | 0.0000 | 0.0000 | 0.2280 | 0.0000 | 0.1498 | 0.0171 | 0.0000 | 0.0524 | 0.0000 | 0.0204 | 0.2979 | 0.0162 | 0.1897 | 0.0000 | 0.0000 | 0.0169 | 0.0000 | 0.0000 | 0.0000 |
| TCGA-D8-A1JT-01 | 0.0266 | 0.0000 | 0.0000 | 0.0000 | 0.0000 | 0.0570 | 0.0000 | 0.0812 | 0.0000 | 0.0153 | 0.0000 | 0.0166 | 0.0000 | 0.2668 | 0.0462 | 0.4136 | 0.0000 | 0.0398 | 0.0268 | 0.0000 | 0.0000 | 0.0099 |
| TCGA-D8-A1JU-01 | 0.0209 | 0.0000 | 0.0213 | 0.0934 | 0.0000 | 0.0967 | 0.0000 | 0.0502 | 0.0000 | 0.0000 | 0.0179 | 0.0087 | 0.0067 | 0.0000 | 0.0472 | 0.4121 | 0.0000 | 0.0000 | 0.2248 | 0.0000 | 0.0000 | 0.0000 |
| TCGA-D8-A1X5-01 | 0.0000 | 0.0000 | 0.0956 | 0.0000 | 0.0000 | 0.1836 | 0.0000 | 0.0489 | 0.0221 | 0.0000 | 0.0314 | 0.0009 | 0.0583 | 0.0048 | 0.0739 | 0.3895 | 0.0000 | 0.0000 | 0.0764 | 0.0000 | 0.0000 | 0.0145 |
| TCGA-D8-A1X6-01 | 0.0001 | 0.0089 | 0.0000 | 0.0000 | 0.0000 | 0.2734 | 0.0000 | 0.0498 | 0.0486 | 0.0000 | 0.0226 | 0.0000 | 0.0341 | 0.0038 | 0.0087 | 0.5309 | 0.0000 | 0.0000 | 0.0190 | 0.0000 | 0.0000 | 0.0000 |
| TCGA-D8-A1X7-01 | 0.0335 | 0.0000 | 0.0159 | 0.0000 | 0.0000 | 0.1006 | 0.0000 | 0.0956 | 0.0016 | 0.0000 | 0.0165 | 0.0000 | 0.0000 | 0.2840 | 0.0200 | 0.3564 | 0.0000 | 0.0000 | 0.0653 | 0.0000 | 0.0000 | 0.0106 |
| TCGA-D8-A1X8-01 | 0.0263 | 0.0000 | 0.0423 | 0.0000 | 0.0000 | 0.2192 | 0.0000 | 0.0321 | 0.0000 | 0.0000 | 0.0317 | 0.0000 | 0.0416 | 0.0000 | 0.0714 | 0.4338 | 0.0000 | 0.0000 | 0.1003 | 0.0012 | 0.0000 | 0.0000 |
| TCGA-D8-A1X9-01 | 0.0345 | 0.0000 | 0.0000 | 0.0319 | 0.0000 | 0.1808 | 0.0000 | 0.0752 | 0.0274 | 0.0000 | 0.0201 | 0.0002 | 0.0030 | 0.1732 | 0.0641 | 0.2703 | 0.0000 | 0.0696 | 0.0475 | 0.0000 | 0.0000 | 0.0022 |
| TCGA-D8-A1XA-01 | 0.0016 | 0.0142 | 0.0026 | 0.0000 | 0.0084 | 0.2135 | 0.0000 | 0.0557 | 0.0958 | 0.0000 | 0.0723 | 0.0000 | 0.0000 | 0.3561 | 0.0152 | 0.0725 | 0.0000 | 0.0000 | 0.0919 | 0.0000 | 0.0000 | 0.0000 |
| TCGA-D8-A1XB-01 | 0.0538 | 0.0000 | 0.0467 | 0.0000 | 0.0000 | 0.1274 | 0.0000 | 0.0398 | 0.0281 | 0.0000 | 0.0000 | 0.0092 | 0.0000 | 0.0872 | 0.0508 | 0.2708 | 0.0086 | 0.0000 | 0.2777 | 0.0000 | 0.0000 | 0.0000 |
| TCGA-D8-A1XC-01 | 0.0018 | 0.0000 | 0.0318 | 0.0000 | 0.0000 | 0.2344 | 0.0000 | 0.0032 | 0.0486 | 0.0000 | 0.0874 | 0.0000 | 0.0145 | 0.2232 | 0.0140 | 0.1278 | 0.0115 | 0.0000 | 0.2017 | 0.0000 | 0.0000 | 0.0000 |
| TCGA-D8-A1XD-01 | 0.0740 | 0.0000 | 0.1335 | 0.0000 | 0.0000 | 0.1509 | 0.0000 | 0.0520 | 0.0150 | 0.0000 | 0.0000 | 0.0564 | 0.0111 | 0.1789 | 0.0052 | 0.2447 | 0.0000 | 0.0000 | 0.0782 | 0.0000 | 0.0000 | 0.0000 |
| TCGA-D8-A1XF-01 | 0.0360 | 0.0000 | 0.2044 | 0.0011 | 0.0000 | 0.0461 | 0.0000 | 0.0385 | 0.0186 | 0.0000 | 0.0152 | 0.0000 | 0.0000 | 0.2213 | 0.0569 | 0.3213 | 0.0000 | 0.0000 | 0.0406 | 0.0000 | 0.0000 | 0.0000 |
| TCGA-D8-A1XG-01 | 0.0526 | 0.0000 | 0.0008 | 0.0000 | 0.0046 | 0.1214 | 0.0000 | 0.0547 | 0.0144 | 0.0000 | 0.0723 | 0.0056 | 0.0183 | 0.0000 | 0.0241 | 0.4547 | 0.0639 | 0.0000 | 0.1107 | 0.0000 | 0.0000 | 0.0020 |
| TCGA-D8-A1XJ-01 | 0.0000 | 0.0000 | 0.0000 | 0.0000 | 0.0000 | 0.3002 | 0.0000 | 0.0256 | 0.0875 | 0.0000 | 0.0376 | 0.0000 | 0.0000 | 0.3807 | 0.0118 | 0.1081 | 0.0000 | 0.0000 | 0.0485 | 0.0000 | 0.0000 | 0.0000 |
| TCGA-D8-A1XK-01 | 0.0302 | 0.0000 | 0.0177 | 0.0248 | 0.0000 | 0.1065 | 0.0198 | 0.0819 | 0.0000 | 0.0000 | 0.0389 | 0.0000 | 0.0000 | 0.4673 | 0.0665 | 0.1372 | 0.0000 | 0.0000 | 0.0092 | 0.0000 | 0.0000 | 0.0000 |
| TCGA-D8-A1XL-01 | 0.0773 | 0.0000 | 0.0693 | 0.1086 | 0.0000 | 0.1368 | 0.0217 | 0.1734 | 0.0256 | 0.0000 | 0.0182 | 0.0000 | 0.0208 | 0.0064 | 0.0998 | 0.2393 | 0.0000 | 0.0000 | 0.0022 | 0.0000 | 0.0000 | 0.0006 |
| TCGA-D8-A1XM-01 | 0.0652 | 0.0000 | 0.0003 | 0.0362 | 0.0000 | 0.1593 | 0.0000 | 0.0389 | 0.0376 | 0.0000 | 0.0406 | 0.0000 | 0.0201 | 0.0430 | 0.0705 | 0.3801 | 0.0288 | 0.0000 | 0.0794 | 0.0000 | 0.0000 | 0.0000 |
| TCGA-D8-A1XO-01 | 0.0806 | 0.0000 | 0.0209 | 0.0421 | 0.0000 | 0.2227 | 0.0000 | 0.0419 | 0.0000 | 0.0000 | 0.0018 | 0.0000 | 0.1390 | 0.0000 | 0.0384 | 0.3050 | 0.0468 | 0.0119 | 0.0477 | 0.0000 | 0.0000 | 0.0013 |
| TCGA-D8-A1XQ-01 | 0.0300 | 0.0000 | 0.1055 | 0.0148 | 0.0000 | 0.1331 | 0.0047 | 0.0468 | 0.0132 | 0.0000 | 0.0203 | 0.0000 | 0.0000 | 0.2027 | 0.1314 | 0.2826 | 0.0000 | 0.0000 | 0.0144 | 0.0000 | 0.0000 | 0.0005 |
| TCGA-D8-A1XR-01 | 0.0014 | 0.0000 | 0.0456 | 0.0000 | 0.0000 | 0.1651 | 0.0015 | 0.0322 | 0.0555 | 0.0000 | 0.0564 | 0.0000 | 0.0000 | 0.4000 | 0.0000 | 0.1830 | 0.0000 | 0.0100 | 0.0492 | 0.0000 | 0.0000 | 0.0000 |
| TCGA-D8-A1XS-01 | 0.0758 | 0.0000 | 0.0487 | 0.0158 | 0.0000 | 0.1496 | 0.0000 | 0.0523 | 0.0267 | 0.0000 | 0.0338 | 0.0000 | 0.0301 | 0.0180 | 0.0674 | 0.3881 | 0.0000 | 0.0015 | 0.0914 | 0.0000 | 0.0000 | 0.0009 |
| TCGA-D8-A1XT-01 | 0.0345 | 0.0000 | 0.0000 | 0.0000 | 0.0000 | 0.0621 | 0.0000 | 0.0614 | 0.0092 | 0.0000 | 0.0244 | 0.0000 | 0.0000 | 0.3675 | 0.1411 | 0.2718 | 0.0000 | 0.0000 | 0.0280 | 0.0000 | 0.0000 | 0.0000 |
| TCGA-D8-A1XU-01 | 0.0829 | 0.0000 | 0.0620 | 0.0130 | 0.0000 | 0.1032 | 0.0000 | 0.0243 | 0.0260 | 0.0000 | 0.0234 | 0.0000 | 0.0151 | 0.0000 | 0.0653 | 0.4017 | 0.0919 | 0.0000 | 0.0881 | 0.0000 | 0.0000 | 0.0032 |
| TCGA-D8-A1XV-01 | 0.0000 | 0.0071 | 0.0254 | 0.0000 | 0.0000 | 0.2366 | 0.0000 | 0.0026 | 0.0390 | 0.0000 | 0.0977 | 0.0000 | 0.0189 | 0.1224 | 0.0122 | 0.1032 | 0.0168 | 0.0000 | 0.3181 | 0.0000 | 0.0000 | 0.0000 |
| TCGA-D8-A1XW-01 | 0.0002 | 0.0067 | 0.0000 | 0.0000 | 0.0000 | 0.2291 | 0.0000 | 0.0230 | 0.0288 | 0.0000 | 0.0117 | 0.0137 | 0.0152 | 0.1447 | 0.1357 | 0.2920 | 0.0126 | 0.0000 | 0.0867 | 0.0000 | 0.0000 | 0.0000 |
| TCGA-D8-A1XY-01 | 0.0015 | 0.0039 | 0.0004 | 0.0000 | 0.0000 | 0.2317 | 0.0000 | 0.0527 | 0.0802 | 0.0000 | 0.0000 | 0.0282 | 0.0377 | 0.2439 | 0.0037 | 0.1541 | 0.0000 | 0.0107 | 0.1341 | 0.0000 | 0.0171 | 0.0000 |
| TCGA-D8-A1XZ-01 | 0.0887 | 0.0000 | 0.0668 | 0.0220 | 0.0000 | 0.1792 | 0.0000 | 0.0985 | 0.0176 | 0.0000 | 0.0109 | 0.0000 | 0.0094 | 0.0394 | 0.0810 | 0.3458 | 0.0000 | 0.0000 | 0.0406 | 0.0000 | 0.0000 | 0.0000 |
| TCGA-D8-A1Y0-01 | 0.0994 | 0.0000 | 0.0211 | 0.0053 | 0.0000 | 0.2588 | 0.0000 | 0.0829 | 0.0143 | 0.0000 | 0.0431 | 0.0000 | 0.0204 | 0.0000 | 0.0993 | 0.2545 | 0.0015 | 0.0000 | 0.0993 | 0.0000 | 0.0000 | 0.0000 |
| TCGA-D8-A1Y1-01 | 0.0000 | 0.0000 | 0.0000 | 0.0000 | 0.0000 | 0.1501 | 0.0000 | 0.0931 | 0.0359 | 0.0000 | 0.0134 | 0.0000 | 0.0188 | 0.2237 | 0.0000 | 0.3919 | 0.0000 | 0.0000 | 0.0640 | 0.0000 | 0.0093 | 0.0000 |
| TCGA-D8-A1Y2-01 | 0.0256 | 0.0000 | 0.0000 | 0.0280 | 0.0000 | 0.1216 | 0.0000 | 0.0141 | 0.0121 | 0.0034 | 0.0187 | 0.0000 | 0.0000 | 0.5049 | 0.0405 | 0.2121 | 0.0000 | 0.0000 | 0.0191 | 0.0000 | 0.0000 | 0.0000 |
| TCGA-D8-A1Y3-01 | 0.0179 | 0.0000 | 0.0000 | 0.0422 | 0.0000 | 0.0886 | 0.0000 | 0.0752 | 0.0128 | 0.0000 | 0.0000 | 0.0146 | 0.0039 | 0.0412 | 0.0600 | 0.5418 | 0.0000 | 0.0000 | 0.0910 | 0.0000 | 0.0000 | 0.0109 |
| TCGA-D8-A27E-01 | 0.0029 | 0.0010 | 0.0000 | 0.0615 | 0.0000 | 0.1341 | 0.0000 | 0.0962 | 0.0279 | 0.0000 | 0.0125 | 0.0000 | 0.0537 | 0.0000 | 0.0295 | 0.3686 | 0.0071 | 0.0000 | 0.1906 | 0.0000 | 0.0144 | 0.0000 |
| TCGA-D8-A27F-01 | 0.0408 | 0.0000 | 0.0100 | 0.0000 | 0.0000 | 0.0953 | 0.0000 | 0.0296 | 0.0213 | 0.0000 | 0.0381 | 0.0000 | 0.0000 | 0.6239 | 0.0089 | 0.1313 | 0.0000 | 0.0000 | 0.0007 | 0.0000 | 0.0000 | 0.0000 |
| TCGA-D8-A27G-01 | 0.0385 | 0.0000 | 0.0103 | 0.0495 | 0.0000 | 0.1061 | 0.0016 | 0.1288 | 0.0216 | 0.0000 | 0.0901 | 0.0000 | 0.0314 | 0.1401 | 0.1154 | 0.1400 | 0.0054 | 0.0000 | 0.1211 | 0.0000 | 0.0000 | 0.0000 |
| TCGA-D8-A27H-01 | 0.1046 | 0.0000 | 0.0532 | 0.0000 | 0.0000 | 0.0992 | 0.0000 | 0.0524 | 0.0635 | 0.0000 | 0.0035 | 0.0164 | 0.0153 | 0.2467 | 0.0302 | 0.2782 | 0.0000 | 0.0000 | 0.0367 | 0.0000 | 0.0000 | 0.0000 |
| TCGA-D8-A27I-01 | 0.0877 | 0.0000 | 0.0398 | 0.0711 | 0.0000 | 0.2089 | 0.0000 | 0.0128 | 0.0208 | 0.0000 | 0.0133 | 0.0000 | 0.0051 | 0.0000 | 0.0691 | 0.2640 | 0.0412 | 0.0000 | 0.1658 | 0.0000 | 0.0000 | 0.0004 |
| TCGA-D8-A27K-01 | 0.0974 | 0.0000 | 0.0000 | 0.0000 | 0.0000 | 0.1536 | 0.0000 | 0.1023 | 0.0066 | 0.0000 | 0.0964 | 0.0000 | 0.0336 | 0.0752 | 0.0874 | 0.1785 | 0.0009 | 0.0000 | 0.1667 | 0.0000 | 0.0014 | 0.0000 |
| TCGA-D8-A27L-01 | 0.0679 | 0.0000 | 0.0453 | 0.0000 | 0.0000 | 0.1280 | 0.0000 | 0.0609 | 0.0448 | 0.0000 | 0.0321 | 0.0000 | 0.0084 | 0.0831 | 0.0668 | 0.2619 | 0.0101 | 0.0000 | 0.1909 | 0.0000 | 0.0000 | 0.0000 |
| TCGA-D8-A27M-01 | 0.0966 | 0.0621 | 0.0356 | 0.0690 | 0.0000 | 0.1376 | 0.0000 | 0.0803 | 0.0411 | 0.0000 | 0.0515 | 0.0000 | 0.0112 | 0.0171 | 0.1509 | 0.2030 | 0.0000 | 0.0120 | 0.0320 | 0.0000 | 0.0000 | 0.0000 |
| TCGA-D8-A27N-01 | 0.0467 | 0.0000 | 0.0320 | 0.0237 | 0.0000 | 0.1561 | 0.0000 | 0.0052 | 0.0110 | 0.0000 | 0.0300 | 0.0000 | 0.0000 | 0.3702 | 0.0434 | 0.2529 | 0.0000 | 0.0000 | 0.0287 | 0.0000 | 0.0000 | 0.0000 |
| TCGA-D8-A27P-01 | 0.0019 | 0.0053 | 0.0202 | 0.0000 | 0.0000 | 0.1895 | 0.0000 | 0.0703 | 0.0305 | 0.0000 | 0.0489 | 0.0156 | 0.0000 | 0.4405 | 0.0085 | 0.0787 | 0.0000 | 0.0000 | 0.0902 | 0.0000 | 0.0000 | 0.0000 |
| TCGA-D8-A27R-01 | 0.0413 | 0.0000 | 0.0281 | 0.1140 | 0.0000 | 0.0822 | 0.0000 | 0.0543 | 0.0569 | 0.0000 | 0.0000 | 0.0042 | 0.0290 | 0.0189 | 0.0878 | 0.3614 | 0.0081 | 0.0000 | 0.1140 | 0.0000 | 0.0000 | 0.0000 |
| TCGA-D8-A27T-01 | 0.0661 | 0.0000 | 0.0077 | 0.0824 | 0.0000 | 0.1318 | 0.0000 | 0.0794 | 0.0288 | 0.0000 | 0.0373 | 0.0000 | 0.0300 | 0.1144 | 0.0898 | 0.2233 | 0.0000 | 0.0000 | 0.1089 | 0.0000 | 0.0000 | 0.0000 |
| TCGA-D8-A27V-01 | 0.1014 | 0.0000 | 0.0794 | 0.0000 | 0.0000 | 0.2026 | 0.0000 | 0.0247 | 0.0000 | 0.0000 | 0.0000 | 0.0299 | 0.0165 | 0.0000 | 0.1033 | 0.2995 | 0.0193 | 0.0000 | 0.1233 | 0.0000 | 0.0000 | 0.0000 |
| TCGA-D8-A27W-01 | 0.1039 | 0.0000 | 0.1942 | 0.0355 | 0.0000 | 0.1339 | 0.0000 | 0.0212 | 0.0209 | 0.0000 | 0.0239 | 0.0000 | 0.0000 | 0.0678 | 0.0321 | 0.3248 | 0.0000 | 0.0000 | 0.0417 | 0.0000 | 0.0000 | 0.0001 |
| TCGA-D8-A3Z5-01 | 0.1262 | 0.0000 | 0.2642 | 0.1225 | 0.0000 | 0.0279 | 0.0000 | 0.1913 | 0.0000 | 0.0000 | 0.0000 | 0.0477 | 0.0189 | 0.0000 | 0.0334 | 0.1679 | 0.0000 | 0.0000 | 0.0000 | 0.0000 | 0.0000 | 0.0000 |
| TCGA-D8-A3Z6-01 | 0.0140 | 0.0000 | 0.0340 | 0.0544 | 0.0000 | 0.0404 | 0.0000 | 0.1136 | 0.0676 | 0.0000 | 0.0539 | 0.0000 | 0.0193 | 0.1118 | 0.0568 | 0.3408 | 0.0000 | 0.0000 | 0.0935 | 0.0000 | 0.0000 | 0.0000 |
| TCGA-D8-A4Z1-01 | 0.1203 | 0.0000 | 0.0663 | 0.0919 | 0.0000 | 0.1264 | 0.0000 | 0.1260 | 0.0000 | 0.0000 | 0.0421 | 0.0000 | 0.0168 | 0.0000 | 0.0548 | 0.1918 | 0.0000 | 0.0000 | 0.1635 | 0.0000 | 0.0000 | 0.0000 |
| TCGA-D8-A73U-01 | 0.0478 | 0.0000 | 0.0124 | 0.1760 | 0.0000 | 0.1504 | 0.0069 | 0.0501 | 0.0751 | 0.0000 | 0.0295 | 0.0051 | 0.0274 | 0.1445 | 0.0818 | 0.1234 | 0.0000 | 0.0000 | 0.0698 | 0.0000 | 0.0000 | 0.0000 |
| TCGA-D8-A73W-01 | 0.0476 | 0.0000 | 0.0000 | 0.0082 | 0.0000 | 0.0748 | 0.0000 | 0.0284 | 0.0203 | 0.0000 | 0.0322 | 0.0000 | 0.0000 | 0.4355 | 0.0320 | 0.2961 | 0.0000 | 0.0000 | 0.0156 | 0.0000 | 0.0000 | 0.0094 |
| TCGA-D8-A73X-01 | 0.0709 | 0.0000 | 0.0096 | 0.0223 | 0.0000 | 0.0579 | 0.0000 | 0.0324 | 0.0077 | 0.0000 | 0.0061 | 0.0086 | 0.0000 | 0.4154 | 0.0225 | 0.2189 | 0.0000 | 0.0000 | 0.1278 | 0.0000 | 0.0000 | 0.0000 |
| TCGA-E2-A105-01 | 0.0041 | 0.0021 | 0.0043 | 0.0000 | 0.0000 | 0.2047 | 0.0000 | 0.0493 | 0.0659 | 0.0000 | 0.0369 | 0.0161 | 0.0000 | 0.4472 | 0.0275 | 0.0892 | 0.0000 | 0.0000 | 0.0459 | 0.0000 | 0.0000 | 0.0067 |
| TCGA-E2-A106-01 | 0.1115 | 0.0000 | 0.1749 | 0.0000 | 0.0000 | 0.0041 | 0.0000 | 0.1282 | 0.0003 | 0.0000 | 0.0079 | 0.0170 | 0.0000 | 0.1381 | 0.0943 | 0.1927 | 0.0000 | 0.0000 | 0.1311 | 0.0000 | 0.0000 | 0.0000 |
| TCGA-E2-A107-01 | 0.0137 | 0.0000 | 0.0444 | 0.0801 | 0.0000 | 0.1406 | 0.0000 | 0.0170 | 0.0418 | 0.0000 | 0.0161 | 0.0270 | 0.0186 | 0.0596 | 0.0160 | 0.3107 | 0.0000 | 0.0332 | 0.1811 | 0.0000 | 0.0000 | 0.0000 |
| TCGA-E2-A108-01 | 0.0390 | 0.0000 | 0.0000 | 0.1629 | 0.0000 | 0.1700 | 0.0000 | 0.1446 | 0.0480 | 0.0000 | 0.0001 | 0.0000 | 0.0079 | 0.0430 | 0.0948 | 0.2149 | 0.0000 | 0.0000 | 0.0747 | 0.0000 | 0.0000 | 0.0000 |
| TCGA-E2-A109-01 | 0.1148 | 0.0000 | 0.0983 | 0.0250 | 0.0000 | 0.1131 | 0.0000 | 0.0556 | 0.0386 | 0.0000 | 0.0072 | 0.0057 | 0.0000 | 0.2495 | 0.0426 | 0.1866 | 0.0000 | 0.0025 | 0.0606 | 0.0000 | 0.0000 | 0.0000 |
| TCGA-E2-A10A-01 | 0.0255 | 0.0000 | 0.0178 | 0.0000 | 0.0000 | 0.1136 | 0.0000 | 0.0284 | 0.0227 | 0.0000 | 0.0170 | 0.0000 | 0.0000 | 0.4223 | 0.0463 | 0.2603 | 0.0000 | 0.0000 | 0.0461 | 0.0000 | 0.0000 | 0.0000 |
| TCGA-E2-A10B-01 | 0.0419 | 0.0000 | 0.0427 | 0.0058 | 0.0000 | 0.1415 | 0.0000 | 0.1030 | 0.0298 | 0.0000 | 0.0527 | 0.0000 | 0.0163 | 0.0212 | 0.0545 | 0.3525 | 0.0202 | 0.0000 | 0.1178 | 0.0000 | 0.0000 | 0.0000 |
| TCGA-E2-A10C-01 | 0.0592 | 0.0000 | 0.0157 | 0.1147 | 0.0000 | 0.1267 | 0.0000 | 0.0844 | 0.0727 | 0.0000 | 0.0122 | 0.0000 | 0.0000 | 0.2544 | 0.0537 | 0.1647 | 0.0000 | 0.0000 | 0.0417 | 0.0000 | 0.0000 | 0.0000 |
| TCGA-E2-A10E-01 | 0.0657 | 0.0000 | 0.0330 | 0.0219 | 0.0000 | 0.2149 | 0.0000 | 0.1211 | 0.0065 | 0.0000 | 0.0011 | 0.0128 | 0.0075 | 0.0762 | 0.1456 | 0.1840 | 0.0314 | 0.0000 | 0.0783 | 0.0000 | 0.0000 | 0.0000 |
| TCGA-E2-A10F-01 | 0.0467 | 0.0000 | 0.0070 | 0.0725 | 0.0000 | 0.1835 | 0.0000 | 0.0540 | 0.0152 | 0.0000 | 0.0083 | 0.0062 | 0.0099 | 0.1081 | 0.0819 | 0.2724 | 0.0081 | 0.0000 | 0.1262 | 0.0000 | 0.0000 | 0.0000 |
| TCGA-E2-A14N-01 | 0.1237 | 0.0000 | 0.0002 | 0.0323 | 0.0000 | 0.2192 | 0.0417 | 0.1015 | 0.0101 | 0.0000 | 0.0383 | 0.0000 | 0.0000 | 0.0847 | 0.1989 | 0.1225 | 0.0000 | 0.0182 | 0.0086 | 0.0000 | 0.0000 | 0.0000 |
| TCGA-E2-A14O-01 | 0.0380 | 0.0000 | 0.0000 | 0.0468 | 0.0000 | 0.0973 | 0.0000 | 0.0318 | 0.0180 | 0.0000 | 0.0044 | 0.0140 | 0.0000 | 0.5059 | 0.0624 | 0.1359 | 0.0021 | 0.0000 | 0.0433 | 0.0000 | 0.0000 | 0.0000 |
| TCGA-E2-A14P-01 | 0.0692 | 0.0000 | 0.0754 | 0.0496 | 0.0000 | 0.0954 | 0.0017 | 0.1446 | 0.0561 | 0.0000 | 0.0040 | 0.0000 | 0.0147 | 0.1760 | 0.1277 | 0.1591 | 0.0000 | 0.0000 | 0.0265 | 0.0000 | 0.0000 | 0.0000 |
| TCGA-E2-A14Q-01 | 0.1092 | 0.0000 | 0.0575 | 0.1275 | 0.0000 | 0.1794 | 0.0000 | 0.0766 | 0.0058 | 0.0090 | 0.0000 | 0.0000 | 0.0000 | 0.0000 | 0.1090 | 0.2144 | 0.0008 | 0.0000 | 0.1108 | 0.0000 | 0.0000 | 0.0000 |
| TCGA-E2-A14R-01 | 0.0129 | 0.0000 | 0.0709 | 0.0683 | 0.0000 | 0.0528 | 0.0167 | 0.1041 | 0.0197 | 0.0000 | 0.0000 | 0.0193 | 0.0000 | 0.3724 | 0.1170 | 0.1072 | 0.0000 | 0.0016 | 0.0372 | 0.0000 | 0.0000 | 0.0000 |
| TCGA-E2-A14S-01 | 0.0377 | 0.0000 | 0.0089 | 0.0195 | 0.0000 | 0.2211 | 0.0020 | 0.0130 | 0.0054 | 0.0000 | 0.0495 | 0.0000 | 0.0000 | 0.1594 | 0.0358 | 0.4344 | 0.0000 | 0.0000 | 0.0133 | 0.0001 | 0.0000 | 0.0000 |
| TCGA-E2-A14T-01 | 0.0509 | 0.0000 | 0.0054 | 0.0162 | 0.0000 | 0.0204 | 0.0000 | 0.0160 | 0.0035 | 0.0000 | 0.0000 | 0.0000 | 0.0000 | 0.6772 | 0.0248 | 0.1491 | 0.0000 | 0.0000 | 0.0365 | 0.0000 | 0.0000 | 0.0000 |
| TCGA-E2-A14U-01 | 0.0000 | 0.0010 | 0.0096 | 0.0000 | 0.0000 | 0.1547 | 0.0000 | 0.1557 | 0.0009 | 0.0000 | 0.0808 | 0.0033 | 0.0336 | 0.1079 | 0.0324 | 0.1206 | 0.0046 | 0.0000 | 0.2950 | 0.0000 | 0.0000 | 0.0000 |
| TCGA-E2-A14V-01 | 0.0923 | 0.0000 | 0.0355 | 0.0450 | 0.0000 | 0.1409 | 0.0000 | 0.0619 | 0.0442 | 0.0000 | 0.0166 | 0.0000 | 0.0000 | 0.1696 | 0.0593 | 0.1933 | 0.0000 | 0.0093 | 0.1320 | 0.0000 | 0.0000 | 0.0000 |
| TCGA-E2-A14W-01 | 0.0000 | 0.0021 | 0.0341 | 0.0000 | 0.0581 | 0.0009 | 0.0008 | 0.0545 | 0.0787 | 0.0000 | 0.0426 | 0.0002 | 0.0000 | 0.5211 | 0.0000 | 0.1023 | 0.0000 | 0.0000 | 0.0872 | 0.0000 | 0.0173 | 0.0000 |
| TCGA-E2-A14X-01 | 0.1892 | 0.0000 | 0.1439 | 0.0630 | 0.0000 | 0.1638 | 0.0048 | 0.1359 | 0.0000 | 0.0153 | 0.0000 | 0.0000 | 0.0000 | 0.0548 | 0.0738 | 0.1423 | 0.0000 | 0.0019 | 0.0114 | 0.0000 | 0.0000 | 0.0000 |
| TCGA-E2-A14Y-01 | 0.0000 | 0.0000 | 0.0489 | 0.0000 | 0.0000 | 0.0603 | 0.0000 | 0.0991 | 0.0000 | 0.0000 | 0.0404 | 0.0047 | 0.0000 | 0.3914 | 0.0458 | 0.0982 | 0.0000 | 0.1568 | 0.0523 | 0.0000 | 0.0000 | 0.0022 |
| TCGA-E2-A14Z-01 | 0.0790 | 0.0000 | 0.0197 | 0.0554 | 0.0000 | 0.1124 | 0.0089 | 0.1338 | 0.0547 | 0.0000 | 0.0558 | 0.0000 | 0.0449 | 0.0907 | 0.0894 | 0.1334 | 0.0037 | 0.0027 | 0.1155 | 0.0000 | 0.0000 | 0.0000 |
| TCGA-E2-A150-01 | 0.0227 | 0.0000 | 0.0000 | 0.0123 | 0.0000 | 0.1398 | 0.0083 | 0.0859 | 0.0395 | 0.0000 | 0.0513 | 0.0000 | 0.0000 | 0.4275 | 0.0969 | 0.1037 | 0.0000 | 0.0000 | 0.0119 | 0.0000 | 0.0000 | 0.0000 |
| TCGA-E2-A152-01 | 0.0088 | 0.0000 | 0.0768 | 0.0000 | 0.0000 | 0.0465 | 0.0000 | 0.0332 | 0.0211 | 0.0049 | 0.0000 | 0.0259 | 0.0000 | 0.3049 | 0.0451 | 0.3475 | 0.0000 | 0.0000 | 0.0851 | 0.0000 | 0.0000 | 0.0000 |
| TCGA-E2-A153-01 | 0.0000 | 0.0044 | 0.0000 | 0.0000 | 0.0000 | 0.1773 | 0.0000 | 0.0392 | 0.0372 | 0.0000 | 0.0152 | 0.0075 | 0.0000 | 0.4114 | 0.0001 | 0.1997 | 0.0000 | 0.0003 | 0.1075 | 0.0000 | 0.0000 | 0.0000 |
| TCGA-E2-A154-01 | 0.0000 | 0.0054 | 0.0282 | 0.0000 | 0.0000 | 0.2086 | 0.0000 | 0.0138 | 0.0703 | 0.0000 | 0.0755 | 0.0018 | 0.0246 | 0.3342 | 0.0000 | 0.0238 | 0.0000 | 0.0000 | 0.2138 | 0.0000 | 0.0000 | 0.0000 |
| TCGA-E2-A155-01 | 0.1588 | 0.0000 | 0.0620 | 0.0239 | 0.0000 | 0.1086 | 0.0000 | 0.0704 | 0.0000 | 0.0000 | 0.0000 | 0.0077 | 0.0000 | 0.1746 | 0.0528 | 0.2432 | 0.0000 | 0.0000 | 0.0981 | 0.0000 | 0.0000 | 0.0000 |
| TCGA-E2-A156-01 | 0.0281 | 0.0000 | 0.0678 | 0.0000 | 0.0000 | 0.0525 | 0.0000 | 0.0468 | 0.0190 | 0.0000 | 0.0000 | 0.0371 | 0.0000 | 0.1732 | 0.0000 | 0.4230 | 0.0000 | 0.0325 | 0.1199 | 0.0000 | 0.0000 | 0.0000 |
| TCGA-E2-A158-01 | 0.1043 | 0.0000 | 0.1001 | 0.0512 | 0.0000 | 0.0498 | 0.0000 | 0.2161 | 0.0000 | 0.0000 | 0.0000 | 0.0161 | 0.0096 | 0.0921 | 0.1510 | 0.1368 | 0.0303 | 0.0068 | 0.0359 | 0.0000 | 0.0000 | 0.0000 |
| TCGA-E2-A159-01 | 0.0251 | 0.0000 | 0.0438 | 0.0809 | 0.0000 | 0.1974 | 0.0086 | 0.0647 | 0.0420 | 0.0000 | 0.0264 | 0.0000 | 0.0000 | 0.1411 | 0.1394 | 0.1816 | 0.0051 | 0.0000 | 0.0438 | 0.0000 | 0.0000 | 0.0000 |
| TCGA-E2-A15A-01 | 0.0020 | 0.0000 | 0.0445 | 0.1152 | 0.0000 | 0.0325 | 0.0000 | 0.0944 | 0.0039 | 0.0000 | 0.0351 | 0.0073 | 0.0222 | 0.1447 | 0.1420 | 0.2057 | 0.0071 | 0.0000 | 0.1433 | 0.0000 | 0.0000 | 0.0000 |
| TCGA-E2-A15C-01 | 0.1960 | 0.0000 | 0.1663 | 0.0261 | 0.0000 | 0.1233 | 0.0000 | 0.1159 | 0.0000 | 0.0000 | 0.0129 | 0.0000 | 0.0000 | 0.0703 | 0.0578 | 0.1733 | 0.0000 | 0.0000 | 0.0583 | 0.0000 | 0.0000 | 0.0000 |
| TCGA-E2-A15D-01 | 0.1091 | 0.0000 | 0.0111 | 0.0764 | 0.0000 | 0.1172 | 0.0000 | 0.1679 | 0.0000 | 0.0135 | 0.0000 | 0.0066 | 0.0031 | 0.1183 | 0.0820 | 0.2415 | 0.0000 | 0.0000 | 0.0533 | 0.0000 | 0.0000 | 0.0000 |
| TCGA-E2-A15E-01 | 0.1083 | 0.0000 | 0.0300 | 0.0380 | 0.0000 | 0.1009 | 0.0000 | 0.0740 | 0.0000 | 0.0000 | 0.0000 | 0.0544 | 0.0268 | 0.0520 | 0.0245 | 0.2310 | 0.0106 | 0.0126 | 0.2369 | 0.0000 | 0.0000 | 0.0000 |
| TCGA-E2-A15F-01 | 0.0361 | 0.0123 | 0.0124 | 0.0000 | 0.0000 | 0.2491 | 0.0000 | 0.1047 | 0.0413 | 0.0000 | 0.0355 | 0.0000 | 0.0000 | 0.2135 | 0.0000 | 0.1696 | 0.0000 | 0.0748 | 0.0508 | 0.0000 | 0.0000 | 0.0000 |
| TCGA-E2-A15G-01 | 0.0827 | 0.0000 | 0.0067 | 0.0531 | 0.0000 | 0.0843 | 0.0000 | 0.0569 | 0.0000 | 0.0000 | 0.0213 | 0.0000 | 0.0937 | 0.0000 | 0.0408 | 0.4436 | 0.0049 | 0.0000 | 0.1120 | 0.0000 | 0.0000 | 0.0000 |
| TCGA-E2-A15H-01 | 0.1215 | 0.0000 | 0.1141 | 0.0606 | 0.0000 | 0.0414 | 0.0000 | 0.1117 | 0.0180 | 0.0000 | 0.0115 | 0.0096 | 0.0000 | 0.2155 | 0.0626 | 0.1961 | 0.0000 | 0.0000 | 0.0376 | 0.0000 | 0.0000 | 0.0000 |
| TCGA-E2-A15I-01 | 0.1282 | 0.0000 | 0.0008 | 0.0510 | 0.0000 | 0.1608 | 0.0000 | 0.1191 | 0.0019 | 0.0505 | 0.0000 | 0.0000 | 0.0000 | 0.1142 | 0.0725 | 0.2137 | 0.0000 | 0.0044 | 0.0831 | 0.0000 | 0.0000 | 0.0000 |
| TCGA-E2-A15J-01 | 0.0000 | 0.0153 | 0.0075 | 0.0000 | 0.0000 | 0.2482 | 0.0000 | 0.2104 | 0.0284 | 0.0000 | 0.0421 | 0.0207 | 0.0377 | 0.2907 | 0.0000 | 0.0000 | 0.0000 | 0.0562 | 0.0391 | 0.0000 | 0.0036 | 0.0000 |
| TCGA-E2-A15K-01 | 0.1349 | 0.0000 | 0.0000 | 0.1144 | 0.0000 | 0.0799 | 0.0000 | 0.1142 | 0.0669 | 0.0000 | 0.0329 | 0.0000 | 0.0000 | 0.1959 | 0.0415 | 0.1718 | 0.0000 | 0.0071 | 0.0404 | 0.0000 | 0.0000 | 0.0000 |
| TCGA-E2-A15L-01 | 0.0120 | 0.0000 | 0.0913 | 0.0000 | 0.0000 | 0.0911 | 0.0000 | 0.0788 | 0.0159 | 0.0000 | 0.0000 | 0.0249 | 0.0000 | 0.2667 | 0.0213 | 0.2875 | 0.0000 | 0.0000 | 0.1106 | 0.0000 | 0.0000 | 0.0000 |
| TCGA-E2-A15M-01 | 0.0732 | 0.0000 | 0.0000 | 0.0000 | 0.0000 | 0.3193 | 0.0194 | 0.0709 | 0.0169 | 0.0179 | 0.0314 | 0.0000 | 0.0124 | 0.0269 | 0.0600 | 0.2914 | 0.0119 | 0.0140 | 0.0345 | 0.0000 | 0.0000 | 0.0000 |
| TCGA-E2-A15O-01 | 0.0015 | 0.0138 | 0.0000 | 0.0000 | 0.0000 | 0.1542 | 0.0000 | 0.1127 | 0.0676 | 0.0000 | 0.1033 | 0.0000 | 0.0050 | 0.3500 | 0.0505 | 0.0802 | 0.0000 | 0.0000 | 0.0611 | 0.0000 | 0.0000 | 0.0000 |
| TCGA-E2-A15P-01 | 0.1205 | 0.0000 | 0.1070 | 0.0500 | 0.0000 | 0.0694 | 0.0000 | 0.0563 | 0.0163 | 0.0000 | 0.0146 | 0.0000 | 0.0000 | 0.0000 | 0.0668 | 0.3446 | 0.0000 | 0.0202 | 0.1343 | 0.0000 | 0.0000 | 0.0000 |
| TCGA-E2-A15R-01 | 0.0756 | 0.0000 | 0.0953 | 0.1198 | 0.0000 | 0.1103 | 0.0000 | 0.0515 | 0.0000 | 0.0000 | 0.0000 | 0.0389 | 0.0221 | 0.0000 | 0.0160 | 0.3198 | 0.0000 | 0.0000 | 0.1507 | 0.0000 | 0.0000 | 0.0000 |
| TCGA-E2-A15S-01 | 0.0019 | 0.0000 | 0.0235 | 0.1219 | 0.0000 | 0.1661 | 0.0000 | 0.0243 | 0.0241 | 0.0000 | 0.1256 | 0.0000 | 0.0000 | 0.2998 | 0.0347 | 0.1185 | 0.0158 | 0.0000 | 0.0439 | 0.0000 | 0.0000 | 0.0000 |
| TCGA-E2-A15T-01 | 0.0054 | 0.0000 | 0.0579 | 0.0000 | 0.0000 | 0.2020 | 0.0000 | 0.0706 | 0.0425 | 0.0000 | 0.0217 | 0.0091 | 0.0000 | 0.3996 | 0.0000 | 0.1163 | 0.0000 | 0.0000 | 0.0648 | 0.0000 | 0.0100 | 0.0000 |
| TCGA-E2-A1AZ-01 | 0.0991 | 0.0000 | 0.0000 | 0.0000 | 0.0000 | 0.1568 | 0.0000 | 0.1679 | 0.0016 | 0.0299 | 0.0088 | 0.0075 | 0.0141 | 0.1095 | 0.1308 | 0.1207 | 0.0000 | 0.0383 | 0.1150 | 0.0000 | 0.0000 | 0.0000 |
| TCGA-E2-A1B0-01 | 0.0659 | 0.0000 | 0.0425 | 0.0222 | 0.0000 | 0.1238 | 0.0041 | 0.0957 | 0.0000 | 0.0146 | 0.0000 | 0.0000 | 0.0000 | 0.2903 | 0.0548 | 0.1750 | 0.0000 | 0.0803 | 0.0307 | 0.0000 | 0.0000 | 0.0000 |
| TCGA-E2-A1B1-01 | 0.0388 | 0.0000 | 0.0299 | 0.0000 | 0.0000 | 0.2487 | 0.0000 | 0.0494 | 0.0099 | 0.0017 | 0.0165 | 0.0000 | 0.0164 | 0.2156 | 0.1190 | 0.1656 | 0.0013 | 0.0000 | 0.0871 | 0.0000 | 0.0000 | 0.0000 |
| TCGA-E2-A1B4-01 | 0.1052 | 0.0000 | 0.0251 | 0.0366 | 0.0000 | 0.1258 | 0.0000 | 0.0761 | 0.0973 | 0.0000 | 0.0000 | 0.0000 | 0.0004 | 0.1285 | 0.0497 | 0.2594 | 0.0000 | 0.0042 | 0.0916 | 0.0000 | 0.0000 | 0.0000 |
| TCGA-E2-A1B5-01 | 0.0662 | 0.0882 | 0.0000 | 0.1357 | 0.0000 | 0.1333 | 0.0140 | 0.0725 | 0.1418 | 0.0000 | 0.0924 | 0.0000 | 0.0270 | 0.0219 | 0.1108 | 0.0621 | 0.0053 | 0.0000 | 0.0232 | 0.0000 | 0.0056 | 0.0000 |
| TCGA-E2-A1B6-01 | 0.0843 | 0.1247 | 0.0000 | 0.1262 | 0.0000 | 0.1207 | 0.0106 | 0.0589 | 0.0980 | 0.0000 | 0.0839 | 0.0000 | 0.0193 | 0.1102 | 0.1062 | 0.0439 | 0.0000 | 0.0131 | 0.0000 | 0.0000 | 0.0000 | 0.0000 |
| TCGA-E2-A1BC-01 | 0.1094 | 0.0000 | 0.0638 | 0.0000 | 0.0000 | 0.2832 | 0.0000 | 0.1030 | 0.0000 | 0.0000 | 0.0000 | 0.0657 | 0.0117 | 0.0701 | 0.0468 | 0.1907 | 0.0161 | 0.0000 | 0.0395 | 0.0000 | 0.0000 | 0.0000 |
| TCGA-E2-A1BD-01 | 0.0000 | 0.0015 | 0.0057 | 0.0000 | 0.0000 | 0.1427 | 0.0000 | 0.1201 | 0.0931 | 0.0000 | 0.0456 | 0.0000 | 0.0157 | 0.2791 | 0.0163 | 0.0835 | 0.0000 | 0.0115 | 0.1852 | 0.0000 | 0.0000 | 0.0000 |
| TCGA-E2-A1IE-01 | 0.0676 | 0.0000 | 0.0859 | 0.0000 | 0.0000 | 0.1163 | 0.0000 | 0.0695 | 0.0132 | 0.0000 | 0.0000 | 0.0429 | 0.0000 | 0.0823 | 0.0424 | 0.3647 | 0.0000 | 0.0000 | 0.1153 | 0.0000 | 0.0000 | 0.0000 |
| TCGA-E2-A1IF-01 | 0.0579 | 0.0000 | 0.0192 | 0.0580 | 0.0000 | 0.0943 | 0.0000 | 0.0377 | 0.0282 | 0.0000 | 0.0055 | 0.0048 | 0.0000 | 0.4579 | 0.0196 | 0.1830 | 0.0000 | 0.0000 | 0.0339 | 0.0000 | 0.0000 | 0.0000 |
| TCGA-E2-A1IG-01 | 0.1110 | 0.0000 | 0.0095 | 0.0308 | 0.0000 | 0.1652 | 0.0000 | 0.0486 | 0.0377 | 0.0000 | 0.0094 | 0.0066 | 0.0000 | 0.1828 | 0.0644 | 0.3110 | 0.0000 | 0.0000 | 0.0166 | 0.0000 | 0.0000 | 0.0064 |
| TCGA-E2-A1IH-01 | 0.0270 | 0.0298 | 0.0001 | 0.1095 | 0.0000 | 0.1799 | 0.0000 | 0.1121 | 0.0021 | 0.0411 | 0.0000 | 0.0000 | 0.0076 | 0.2682 | 0.1002 | 0.0879 | 0.0000 | 0.0000 | 0.0344 | 0.0000 | 0.0000 | 0.0000 |
| TCGA-E2-A1II-01 | 0.0373 | 0.0000 | 0.0000 | 0.1648 | 0.0000 | 0.1984 | 0.0582 | 0.0914 | 0.0710 | 0.0000 | 0.0352 | 0.0050 | 0.0132 | 0.0302 | 0.1421 | 0.1160 | 0.0000 | 0.0034 | 0.0340 | 0.0000 | 0.0000 | 0.0000 |
| TCGA-E2-A1IJ-01 | 0.0111 | 0.0000 | 0.0222 | 0.1737 | 0.0000 | 0.0265 | 0.0000 | 0.1067 | 0.0516 | 0.0000 | 0.0074 | 0.0230 | 0.0343 | 0.0914 | 0.0969 | 0.1454 | 0.0104 | 0.0000 | 0.1994 | 0.0000 | 0.0000 | 0.0000 |
| TCGA-E2-A1IK-01 | 0.0894 | 0.0000 | 0.0847 | 0.1051 | 0.0000 | 0.1225 | 0.0016 | 0.0473 | 0.0381 | 0.0035 | 0.0147 | 0.0000 | 0.0000 | 0.1401 | 0.0994 | 0.2318 | 0.0000 | 0.0000 | 0.0075 | 0.0143 | 0.0000 | 0.0000 |
| TCGA-E2-A1IL-01 | 0.0476 | 0.0000 | 0.0862 | 0.0000 | 0.0000 | 0.1065 | 0.0000 | 0.1071 | 0.0000 | 0.0000 | 0.0000 | 0.0552 | 0.0000 | 0.1356 | 0.0980 | 0.2964 | 0.0000 | 0.0000 | 0.0675 | 0.0000 | 0.0000 | 0.0000 |
| TCGA-E2-A1IN-01 | 0.0000 | 0.0000 | 0.0013 | 0.0000 | 0.0000 | 0.2805 | 0.0000 | 0.0450 | 0.0631 | 0.0000 | 0.0161 | 0.0000 | 0.0282 | 0.0185 | 0.0100 | 0.3201 | 0.0000 | 0.0043 | 0.2129 | 0.0000 | 0.0000 | 0.0000 |
| TCGA-E2-A1IO-01 | 0.1138 | 0.0000 | 0.0267 | 0.1229 | 0.0000 | 0.1726 | 0.0000 | 0.0744 | 0.0480 | 0.0000 | 0.0125 | 0.0000 | 0.0000 | 0.0780 | 0.0819 | 0.1869 | 0.0000 | 0.0173 | 0.0651 | 0.0000 | 0.0000 | 0.0000 |
| TCGA-E2-A1IU-01 | 0.1616 | 0.0000 | 0.0960 | 0.0707 | 0.0000 | 0.0921 | 0.0000 | 0.1160 | 0.0000 | 0.0000 | 0.0000 | 0.0000 | 0.0170 | 0.0000 | 0.0598 | 0.3099 | 0.0000 | 0.0000 | 0.0769 | 0.0000 | 0.0000 | 0.0000 |
| TCGA-E2-A1L6-01 | 0.0430 | 0.0000 | 0.0246 | 0.0000 | 0.0000 | 0.0042 | 0.0000 | 0.1276 | 0.0043 | 0.0096 | 0.0190 | 0.0000 | 0.0000 | 0.4240 | 0.0393 | 0.2483 | 0.0000 | 0.0000 | 0.0560 | 0.0000 | 0.0000 | 0.0000 |
| TCGA-E2-A1L7-01 | 0.0679 | 0.0000 | 0.0285 | 0.1519 | 0.0000 | 0.0971 | 0.0137 | 0.1343 | 0.0335 | 0.0226 | 0.0000 | 0.0001 | 0.0000 | 0.1014 | 0.0936 | 0.2164 | 0.0084 | 0.0068 | 0.0238 | 0.0000 | 0.0000 | 0.0000 |
| TCGA-E2-A1L8-01 | 0.1179 | 0.0000 | 0.0771 | 0.0606 | 0.0000 | 0.1044 | 0.0000 | 0.0638 | 0.0039 | 0.0000 | 0.0048 | 0.0000 | 0.0000 | 0.0000 | 0.0583 | 0.2080 | 0.1723 | 0.0605 | 0.0683 | 0.0000 | 0.0000 | 0.0000 |
| TCGA-E2-A1L9-01 | 0.0002 | 0.0051 | 0.0025 | 0.0000 | 0.0000 | 0.0805 | 0.0000 | 0.0270 | 0.0421 | 0.0000 | 0.0260 | 0.0000 | 0.0000 | 0.4943 | 0.0124 | 0.2386 | 0.0000 | 0.0114 | 0.0598 | 0.0000 | 0.0000 | 0.0000 |
| TCGA-E2-A1LA-01 | 0.0222 | 0.0000 | 0.0746 | 0.1174 | 0.0000 | 0.0363 | 0.0000 | 0.1359 | 0.0334 | 0.0000 | 0.0000 | 0.0328 | 0.0185 | 0.2618 | 0.0824 | 0.1268 | 0.0000 | 0.0000 | 0.0579 | 0.0000 | 0.0000 | 0.0000 |
| TCGA-E2-A1LB-01 | 0.0349 | 0.0000 | 0.1181 | 0.0303 | 0.0000 | 0.0832 | 0.0000 | 0.0822 | 0.0173 | 0.0000 | 0.0000 | 0.0317 | 0.0101 | 0.0000 | 0.0292 | 0.4817 | 0.0000 | 0.0000 | 0.0813 | 0.0000 | 0.0000 | 0.0000 |
| TCGA-E2-A1LE-01 | 0.0415 | 0.0000 | 0.0101 | 0.1169 | 0.0000 | 0.1968 | 0.0000 | 0.0639 | 0.0379 | 0.0000 | 0.0109 | 0.0000 | 0.0000 | 0.2510 | 0.0736 | 0.1852 | 0.0000 | 0.0000 | 0.0123 | 0.0000 | 0.0000 | 0.0000 |
| TCGA-E2-A1LG-01 | 0.0332 | 0.0000 | 0.1241 | 0.0388 | 0.0000 | 0.0561 | 0.0000 | 0.1508 | 0.0000 | 0.0008 | 0.0000 | 0.0105 | 0.0000 | 0.1236 | 0.2072 | 0.1827 | 0.0156 | 0.0045 | 0.0522 | 0.0000 | 0.0000 | 0.0000 |
| TCGA-E2-A1LH-01 | 0.0338 | 0.0078 | 0.0000 | 0.0784 | 0.0000 | 0.1764 | 0.0000 | 0.1026 | 0.0564 | 0.0000 | 0.0093 | 0.0156 | 0.0000 | 0.2037 | 0.1760 | 0.0966 | 0.0052 | 0.0000 | 0.0381 | 0.0000 | 0.0000 | 0.0000 |
| TCGA-E2-A1LI-01 | 0.0923 | 0.0000 | 0.0391 | 0.0073 | 0.0000 | 0.0846 | 0.0034 | 0.1083 | 0.0291 | 0.0000 | 0.0313 | 0.0000 | 0.0138 | 0.3410 | 0.0875 | 0.1487 | 0.0000 | 0.0000 | 0.0000 | 0.0137 | 0.0000 | 0.0000 |
| TCGA-E2-A1LK-01 | 0.0037 | 0.0000 | 0.0027 | 0.0000 | 0.0000 | 0.0360 | 0.0000 | 0.0270 | 0.0589 | 0.0000 | 0.0115 | 0.0000 | 0.0000 | 0.6752 | 0.0087 | 0.1086 | 0.0000 | 0.0596 | 0.0000 | 0.0065 | 0.0000 | 0.0016 |
| TCGA-E2-A1LL-01 | 0.0056 | 0.0000 | 0.0603 | 0.0062 | 0.0000 | 0.0961 | 0.0000 | 0.1292 | 0.0256 | 0.0000 | 0.0000 | 0.0325 | 0.0386 | 0.3343 | 0.0000 | 0.1148 | 0.0091 | 0.0000 | 0.1346 | 0.0000 | 0.0131 | 0.0000 |
| TCGA-E2-A1LS-01 | 0.1451 | 0.0000 | 0.0515 | 0.0936 | 0.0000 | 0.0765 | 0.0000 | 0.1236 | 0.0000 | 0.0000 | 0.0352 | 0.0000 | 0.0127 | 0.0000 | 0.0813 | 0.3180 | 0.0000 | 0.0624 | 0.0000 | 0.0000 | 0.0000 | 0.0000 |
| TCGA-E2-A2P5-01 | 0.0161 | 0.0000 | 0.0151 | 0.0821 | 0.0000 | 0.2686 | 0.0000 | 0.0969 | 0.0705 | 0.0000 | 0.0305 | 0.0169 | 0.0212 | 0.0098 | 0.0751 | 0.1787 | 0.0052 | 0.0094 | 0.1038 | 0.0000 | 0.0000 | 0.0000 |
| TCGA-E2-A2P6-01 | 0.0865 | 0.0000 | 0.0353 | 0.0000 | 0.0000 | 0.1265 | 0.0000 | 0.0728 | 0.0113 | 0.0000 | 0.0000 | 0.0104 | 0.0296 | 0.0000 | 0.0713 | 0.3893 | 0.0000 | 0.0000 | 0.1670 | 0.0000 | 0.0000 | 0.0000 |
| TCGA-E2-A3DX-01 | 0.1203 | 0.0216 | 0.0323 | 0.2379 | 0.0000 | 0.1506 | 0.0076 | 0.0694 | 0.0000 | 0.0000 | 0.0000 | 0.0000 | 0.0160 | 0.0103 | 0.0608 | 0.1974 | 0.0000 | 0.0000 | 0.0756 | 0.0000 | 0.0000 | 0.0000 |
| TCGA-E2-A56Z-01 | 0.0436 | 0.0000 | 0.0217 | 0.0280 | 0.0000 | 0.0789 | 0.0000 | 0.0917 | 0.0909 | 0.0000 | 0.0118 | 0.0042 | 0.0000 | 0.2911 | 0.1111 | 0.1748 | 0.0000 | 0.0000 | 0.0521 | 0.0000 | 0.0000 | 0.0000 |
| TCGA-E2-A570-01 | 0.1068 | 0.0000 | 0.0131 | 0.0421 | 0.0000 | 0.0834 | 0.0000 | 0.0973 | 0.0143 | 0.0000 | 0.0027 | 0.0124 | 0.0000 | 0.1245 | 0.0264 | 0.3341 | 0.0000 | 0.0000 | 0.1387 | 0.0000 | 0.0000 | 0.0040 |
| TCGA-E2-A572-01 | 0.0000 | 0.0121 | 0.0967 | 0.2467 | 0.0000 | 0.0000 | 0.0000 | 0.0850 | 0.1004 | 0.0000 | 0.0053 | 0.0000 | 0.0405 | 0.1244 | 0.0176 | 0.1264 | 0.0000 | 0.0274 | 0.1160 | 0.0000 | 0.0017 | 0.0000 |
| TCGA-E2-A573-01 | 0.0341 | 0.0000 | 0.0580 | 0.0000 | 0.0000 | 0.1025 | 0.0046 | 0.1706 | 0.0224 | 0.0000 | 0.0211 | 0.0156 | 0.0000 | 0.3496 | 0.0901 | 0.1101 | 0.0000 | 0.0062 | 0.0150 | 0.0000 | 0.0000 | 0.0000 |
| TCGA-E2-A574-01 | 0.0016 | 0.0034 | 0.0061 | 0.0000 | 0.0000 | 0.1525 | 0.0000 | 0.0613 | 0.0606 | 0.0000 | 0.0473 | 0.0095 | 0.0000 | 0.4689 | 0.0000 | 0.1585 | 0.0000 | 0.0000 | 0.0303 | 0.0000 | 0.0000 | 0.0000 |
| TCGA-E2-A576-01 | 0.0521 | 0.0000 | 0.0898 | 0.1106 | 0.0000 | 0.0700 | 0.0000 | 0.0729 | 0.0453 | 0.0000 | 0.0000 | 0.0548 | 0.0037 | 0.0246 | 0.0653 | 0.1627 | 0.0000 | 0.0055 | 0.2426 | 0.0000 | 0.0000 | 0.0000 |
| TCGA-E2-A9RU-01 | 0.0028 | 0.0000 | 0.0032 | 0.0101 | 0.0000 | 0.0000 | 0.0000 | 0.1136 | 0.0334 | 0.0000 | 0.0108 | 0.0000 | 0.0000 | 0.4529 | 0.0840 | 0.2353 | 0.0000 | 0.0000 | 0.0539 | 0.0000 | 0.0000 | 0.0000 |
| TCGA-E9-A1N3-01 | 0.0000 | 0.0975 | 0.0097 | 0.0246 | 0.0000 | 0.3542 | 0.0000 | 0.0433 | 0.0683 | 0.0000 | 0.0478 | 0.0000 | 0.0648 | 0.0600 | 0.0236 | 0.0742 | 0.0100 | 0.0000 | 0.1218 | 0.0000 | 0.0000 | 0.0000 |
| TCGA-E9-A1N4-01 | 0.0371 | 0.0000 | 0.0000 | 0.0120 | 0.0000 | 0.0623 | 0.0000 | 0.0558 | 0.0032 | 0.0000 | 0.0000 | 0.0036 | 0.0000 | 0.4131 | 0.0756 | 0.2398 | 0.0000 | 0.0000 | 0.0975 | 0.0000 | 0.0000 | 0.0000 |
| TCGA-E9-A1N5-01 | 0.0352 | 0.0000 | 0.0000 | 0.1037 | 0.0000 | 0.1336 | 0.0000 | 0.0543 | 0.0488 | 0.0000 | 0.0000 | 0.0387 | 0.0099 | 0.2158 | 0.0348 | 0.2103 | 0.0000 | 0.0000 | 0.1150 | 0.0000 | 0.0000 | 0.0000 |
| TCGA-E9-A1N6-01 | 0.0255 | 0.0000 | 0.0070 | 0.0101 | 0.0000 | 0.0856 | 0.0000 | 0.0443 | 0.0136 | 0.0000 | 0.0096 | 0.0000 | 0.0000 | 0.4313 | 0.0482 | 0.3009 | 0.0000 | 0.0000 | 0.0239 | 0.0000 | 0.0000 | 0.0000 |
| TCGA-E9-A1N8-01 | 0.0015 | 0.0000 | 0.0000 | 0.0000 | 0.0000 | 0.2791 | 0.0000 | 0.1986 | 0.0000 | 0.0000 | 0.0000 | 0.0591 | 0.0000 | 0.3598 | 0.0639 | 0.0000 | 0.0000 | 0.0000 | 0.0380 | 0.0000 | 0.0000 | 0.0000 |
| TCGA-E9-A1N9-01 | 0.0676 | 0.0000 | 0.0463 | 0.0289 | 0.0000 | 0.2978 | 0.0000 | 0.0674 | 0.0420 | 0.0000 | 0.0366 | 0.0000 | 0.0214 | 0.0774 | 0.1149 | 0.1874 | 0.0005 | 0.0000 | 0.0120 | 0.0000 | 0.0000 | 0.0000 |
| TCGA-E9-A1NA-01 | 0.0000 | 0.0000 | 0.0072 | 0.0000 | 0.0000 | 0.1812 | 0.0000 | 0.0665 | 0.0551 | 0.0000 | 0.0080 | 0.0000 | 0.0181 | 0.0909 | 0.0000 | 0.4028 | 0.0000 | 0.0644 | 0.1051 | 0.0000 | 0.0005 | 0.0000 |
| TCGA-E9-A1NC-01 | 0.0312 | 0.0000 | 0.0559 | 0.0488 | 0.0000 | 0.0000 | 0.0000 | 0.0953 | 0.0779 | 0.0000 | 0.0183 | 0.0000 | 0.0000 | 0.3184 | 0.1718 | 0.1311 | 0.0000 | 0.0000 | 0.0512 | 0.0000 | 0.0000 | 0.0000 |
| TCGA-E9-A1ND-01 | 0.0666 | 0.0000 | 0.0133 | 0.0501 | 0.0000 | 0.1188 | 0.0367 | 0.0972 | 0.0504 | 0.0000 | 0.0806 | 0.0000 | 0.0128 | 0.0528 | 0.2231 | 0.1513 | 0.0000 | 0.0194 | 0.0255 | 0.0000 | 0.0000 | 0.0014 |
| TCGA-E9-A1NE-01 | 0.0989 | 0.0000 | 0.0000 | 0.1363 | 0.0000 | 0.2895 | 0.0078 | 0.1379 | 0.0055 | 0.0455 | 0.0000 | 0.0026 | 0.0103 | 0.0000 | 0.1088 | 0.1312 | 0.0000 | 0.0000 | 0.0256 | 0.0000 | 0.0000 | 0.0000 |
| TCGA-E9-A1NF-01 | 0.0027 | 0.0000 | 0.0090 | 0.0000 | 0.0000 | 0.1059 | 0.0000 | 0.0236 | 0.0361 | 0.0000 | 0.0278 | 0.0000 | 0.0000 | 0.3918 | 0.0430 | 0.3349 | 0.0000 | 0.0000 | 0.0253 | 0.0000 | 0.0000 | 0.0000 |
| TCGA-E9-A1NG-01 | 0.0000 | 0.0000 | 0.0007 | 0.0000 | 0.0000 | 0.1849 | 0.0000 | 0.0035 | 0.0366 | 0.0000 | 0.0000 | 0.0382 | 0.0069 | 0.2074 | 0.0000 | 0.4565 | 0.0000 | 0.0197 | 0.0456 | 0.0000 | 0.0000 | 0.0000 |
| TCGA-E9-A1NH-01 | 0.0955 | 0.0000 | 0.0574 | 0.0359 | 0.0000 | 0.1058 | 0.0000 | 0.0642 | 0.0272 | 0.0000 | 0.0049 | 0.0025 | 0.0000 | 0.1551 | 0.0521 | 0.2672 | 0.0569 | 0.0000 | 0.0667 | 0.0000 | 0.0000 | 0.0087 |
| TCGA-E9-A1NI-01 | 0.0012 | 0.0060 | 0.0088 | 0.0000 | 0.0000 | 0.1807 | 0.0000 | 0.0843 | 0.0593 | 0.0000 | 0.0244 | 0.0155 | 0.0000 | 0.4518 | 0.0060 | 0.0945 | 0.0016 | 0.0000 | 0.0660 | 0.0000 | 0.0000 | 0.0000 |
| TCGA-E9-A1QZ-01 | 0.0217 | 0.1319 | 0.0000 | 0.0379 | 0.0000 | 0.1730 | 0.0000 | 0.0735 | 0.0388 | 0.0021 | 0.0201 | 0.0000 | 0.0085 | 0.0486 | 0.1547 | 0.1324 | 0.0128 | 0.0000 | 0.1440 | 0.0000 | 0.0000 | 0.0000 |
| TCGA-E9-A1R0-01 | 0.1562 | 0.0000 | 0.1087 | 0.0840 | 0.0000 | 0.1320 | 0.0000 | 0.0638 | 0.0339 | 0.0000 | 0.0255 | 0.0000 | 0.0000 | 0.0711 | 0.0227 | 0.1842 | 0.0000 | 0.0163 | 0.1015 | 0.0000 | 0.0000 | 0.0000 |
| TCGA-E9-A1R2-01 | 0.1427 | 0.0000 | 0.0263 | 0.0814 | 0.0000 | 0.1516 | 0.0000 | 0.1705 | 0.0000 | 0.0000 | 0.0121 | 0.0000 | 0.0034 | 0.0000 | 0.1184 | 0.2205 | 0.0000 | 0.0284 | 0.0447 | 0.0000 | 0.0000 | 0.0000 |
| TCGA-E9-A1R3-01 | 0.0289 | 0.0000 | 0.0007 | 0.0000 | 0.0000 | 0.1290 | 0.0000 | 0.0396 | 0.0000 | 0.0000 | 0.0219 | 0.0000 | 0.0000 | 0.1612 | 0.0343 | 0.3999 | 0.0564 | 0.0580 | 0.0633 | 0.0000 | 0.0000 | 0.0068 |
| TCGA-E9-A1R4-01 | 0.0086 | 0.0000 | 0.0067 | 0.0831 | 0.0000 | 0.1530 | 0.0018 | 0.0241 | 0.0521 | 0.0000 | 0.0442 | 0.0088 | 0.0248 | 0.0649 | 0.1309 | 0.2677 | 0.0165 | 0.0000 | 0.1129 | 0.0000 | 0.0000 | 0.0000 |
| TCGA-E9-A1R5-01 | 0.0609 | 0.0000 | 0.0024 | 0.0000 | 0.0000 | 0.1445 | 0.0000 | 0.0192 | 0.0356 | 0.0250 | 0.0000 | 0.0000 | 0.0000 | 0.3213 | 0.0459 | 0.2677 | 0.0000 | 0.0000 | 0.0774 | 0.0000 | 0.0000 | 0.0000 |
| TCGA-E9-A1R6-01 | 0.0016 | 0.0077 | 0.0058 | 0.0000 | 0.0000 | 0.1845 | 0.0000 | 0.0289 | 0.0623 | 0.0000 | 0.0640 | 0.0000 | 0.0000 | 0.4069 | 0.0056 | 0.1783 | 0.0000 | 0.0000 | 0.0543 | 0.0000 | 0.0000 | 0.0000 |
| TCGA-E9-A1R7-01 | 0.1181 | 0.0000 | 0.1308 | 0.0000 | 0.0000 | 0.2202 | 0.0000 | 0.0300 | 0.0256 | 0.0000 | 0.0000 | 0.0385 | 0.0092 | 0.0276 | 0.0593 | 0.2710 | 0.0000 | 0.0000 | 0.0697 | 0.0000 | 0.0000 | 0.0000 |
| TCGA-E9-A1RA-01 | 0.0330 | 0.0468 | 0.0000 | 0.0577 | 0.0000 | 0.0288 | 0.0000 | 0.1619 | 0.0255 | 0.0000 | 0.0594 | 0.0000 | 0.0000 | 0.3306 | 0.0292 | 0.1573 | 0.0000 | 0.0000 | 0.0697 | 0.0000 | 0.0000 | 0.0000 |
| TCGA-E9-A1RB-01 | 0.0035 | 0.0022 | 0.0012 | 0.0000 | 0.0000 | 0.0990 | 0.0000 | 0.0719 | 0.0481 | 0.0000 | 0.0261 | 0.0000 | 0.0000 | 0.5376 | 0.0037 | 0.1463 | 0.0000 | 0.0000 | 0.0605 | 0.0000 | 0.0000 | 0.0000 |
| TCGA-E9-A1RC-01 | 0.0000 | 0.0000 | 0.0468 | 0.0000 | 0.0068 | 0.0844 | 0.0000 | 0.1054 | 0.0168 | 0.0000 | 0.0347 | 0.0046 | 0.0000 | 0.5644 | 0.0000 | 0.0505 | 0.0000 | 0.0443 | 0.0413 | 0.0000 | 0.0000 | 0.0000 |
| TCGA-E9-A1RD-01 | 0.0000 | 0.0076 | 0.0024 | 0.0000 | 0.0000 | 0.3962 | 0.0000 | 0.0222 | 0.0615 | 0.0000 | 0.0000 | 0.0000 | 0.0377 | 0.0000 | 0.0000 | 0.3010 | 0.0000 | 0.0182 | 0.1531 | 0.0000 | 0.0000 | 0.0000 |
| TCGA-E9-A1RE-01 | 0.1195 | 0.0000 | 0.0437 | 0.0199 | 0.0000 | 0.0513 | 0.0000 | 0.1006 | 0.0352 | 0.0000 | 0.0140 | 0.0000 | 0.0000 | 0.2315 | 0.0258 | 0.1785 | 0.0000 | 0.0000 | 0.1800 | 0.0000 | 0.0000 | 0.0000 |
| TCGA-E9-A1RF-01 | 0.0099 | 0.0000 | 0.0182 | 0.1627 | 0.0000 | 0.0650 | 0.0025 | 0.1060 | 0.0303 | 0.0000 | 0.0243 | 0.0027 | 0.0238 | 0.0996 | 0.1483 | 0.2050 | 0.0146 | 0.0000 | 0.0871 | 0.0000 | 0.0000 | 0.0000 |
| TCGA-E9-A1RG-01 | 0.0627 | 0.0000 | 0.0514 | 0.0753 | 0.0000 | 0.1389 | 0.0000 | 0.0543 | 0.0568 | 0.0000 | 0.0059 | 0.0318 | 0.0133 | 0.0748 | 0.0686 | 0.3077 | 0.0066 | 0.0000 | 0.0509 | 0.0000 | 0.0000 | 0.0009 |
| TCGA-E9-A1RH-01 | 0.0000 | 0.0000 | 0.0855 | 0.0000 | 0.0000 | 0.1586 | 0.0000 | 0.0664 | 0.0077 | 0.0000 | 0.0000 | 0.0402 | 0.0000 | 0.3554 | 0.1129 | 0.0671 | 0.0328 | 0.0000 | 0.0734 | 0.0000 | 0.0000 | 0.0000 |
| TCGA-E9-A1RI-01 | 0.0804 | 0.0000 | 0.0735 | 0.0012 | 0.0000 | 0.1602 | 0.0000 | 0.0453 | 0.0036 | 0.0000 | 0.0213 | 0.0000 | 0.0000 | 0.1661 | 0.0402 | 0.3323 | 0.0000 | 0.0000 | 0.0759 | 0.0000 | 0.0000 | 0.0000 |
| TCGA-E9-A226-01 | 0.0013 | 0.0000 | 0.0000 | 0.0000 | 0.0000 | 0.3824 | 0.0000 | 0.0528 | 0.0469 | 0.0000 | 0.0649 | 0.0000 | 0.0159 | 0.1978 | 0.0000 | 0.1995 | 0.0000 | 0.0307 | 0.0077 | 0.0000 | 0.0000 | 0.0000 |
| TCGA-E9-A227-01 | 0.0859 | 0.0000 | 0.0096 | 0.1415 | 0.0000 | 0.1559 | 0.0134 | 0.0548 | 0.0113 | 0.0168 | 0.0000 | 0.0000 | 0.0237 | 0.0000 | 0.0545 | 0.2747 | 0.0617 | 0.0388 | 0.0576 | 0.0000 | 0.0000 | 0.0000 |
| TCGA-E9-A228-01 | 0.0015 | 0.0000 | 0.0237 | 0.0000 | 0.0000 | 0.1463 | 0.0000 | 0.0048 | 0.0374 | 0.0000 | 0.0531 | 0.0000 | 0.0000 | 0.5435 | 0.0134 | 0.1033 | 0.0072 | 0.0000 | 0.0659 | 0.0000 | 0.0000 | 0.0000 |
| TCGA-E9-A229-01 | 0.0702 | 0.0000 | 0.0254 | 0.0437 | 0.0000 | 0.1190 | 0.0000 | 0.0566 | 0.0373 | 0.0000 | 0.0299 | 0.0000 | 0.0701 | 0.0273 | 0.1091 | 0.3378 | 0.0000 | 0.0000 | 0.0703 | 0.0000 | 0.0000 | 0.0034 |
| TCGA-E9-A22A-01 | 0.0063 | 0.0000 | 0.0188 | 0.0792 | 0.0000 | 0.2236 | 0.0000 | 0.0592 | 0.0717 | 0.0000 | 0.0000 | 0.0000 | 0.0000 | 0.1368 | 0.1246 | 0.1859 | 0.0000 | 0.0000 | 0.0845 | 0.0000 | 0.0000 | 0.0094 |
| TCGA-E9-A22B-01 | 0.1095 | 0.0000 | 0.0453 | 0.0188 | 0.0000 | 0.1486 | 0.0000 | 0.0418 | 0.0196 | 0.0000 | 0.0086 | 0.0000 | 0.0000 | 0.0515 | 0.0929 | 0.3292 | 0.0307 | 0.0000 | 0.0634 | 0.0000 | 0.0000 | 0.0402 |
| TCGA-E9-A22D-01 | 0.0700 | 0.0000 | 0.0000 | 0.1840 | 0.0000 | 0.1206 | 0.0937 | 0.0807 | 0.0463 | 0.0000 | 0.0673 | 0.0000 | 0.0162 | 0.0569 | 0.0884 | 0.1158 | 0.0040 | 0.0000 | 0.0562 | 0.0000 | 0.0000 | 0.0000 |
| TCGA-E9-A22E-01 | 0.0689 | 0.0000 | 0.0146 | 0.0545 | 0.0000 | 0.1300 | 0.0000 | 0.0171 | 0.0524 | 0.0000 | 0.0237 | 0.0000 | 0.0000 | 0.2691 | 0.0951 | 0.2120 | 0.0000 | 0.0000 | 0.0626 | 0.0000 | 0.0000 | 0.0000 |
| TCGA-E9-A22G-01 | 0.0510 | 0.0000 | 0.0000 | 0.0155 | 0.0000 | 0.1366 | 0.0100 | 0.0737 | 0.0067 | 0.0000 | 0.0266 | 0.0000 | 0.0000 | 0.3492 | 0.0571 | 0.0605 | 0.0000 | 0.1933 | 0.0198 | 0.0000 | 0.0000 | 0.0000 |
| TCGA-E9-A22H-01 | 0.1419 | 0.0000 | 0.0176 | 0.0779 | 0.0000 | 0.0906 | 0.0000 | 0.0774 | 0.0195 | 0.0000 | 0.0283 | 0.0000 | 0.0032 | 0.0017 | 0.0400 | 0.4023 | 0.0014 | 0.0071 | 0.0913 | 0.0000 | 0.0000 | 0.0000 |
| TCGA-E9-A243-01 | 0.0647 | 0.0000 | 0.0436 | 0.0910 | 0.0000 | 0.1296 | 0.0189 | 0.0613 | 0.0237 | 0.0000 | 0.0000 | 0.0189 | 0.0000 | 0.0739 | 0.1533 | 0.2253 | 0.0000 | 0.0000 | 0.0784 | 0.0000 | 0.0000 | 0.0173 |
| TCGA-E9-A244-01 | 0.0430 | 0.0000 | 0.0265 | 0.0735 | 0.0000 | 0.0883 | 0.0000 | 0.1250 | 0.0271 | 0.0000 | 0.0000 | 0.0000 | 0.0000 | 0.2838 | 0.1133 | 0.2013 | 0.0000 | 0.0000 | 0.0182 | 0.0000 | 0.0000 | 0.0000 |
| TCGA-E9-A245-01 | 0.0000 | 0.0289 | 0.0167 | 0.0000 | 0.0000 | 0.2913 | 0.0000 | 0.0827 | 0.0000 | 0.0000 | 0.0116 | 0.0000 | 0.0345 | 0.0670 | 0.0436 | 0.2182 | 0.0278 | 0.0000 | 0.1776 | 0.0000 | 0.0000 | 0.0000 |
| TCGA-E9-A247-01 | 0.1346 | 0.0000 | 0.0572 | 0.0338 | 0.0000 | 0.0756 | 0.0000 | 0.0410 | 0.0339 | 0.0000 | 0.0013 | 0.0160 | 0.0000 | 0.1488 | 0.0610 | 0.2611 | 0.0000 | 0.0351 | 0.1008 | 0.0000 | 0.0000 | 0.0000 |
| TCGA-E9-A248-01 | 0.0338 | 0.0540 | 0.0678 | 0.1446 | 0.0000 | 0.1279 | 0.0104 | 0.0750 | 0.0447 | 0.0135 | 0.0000 | 0.0000 | 0.0000 | 0.1234 | 0.0895 | 0.1759 | 0.0000 | 0.0000 | 0.0395 | 0.0000 | 0.0000 | 0.0000 |
| TCGA-E9-A249-01 | 0.1050 | 0.0000 | 0.0000 | 0.0500 | 0.0000 | 0.0763 | 0.0000 | 0.1846 | 0.0116 | 0.0000 | 0.0296 | 0.0000 | 0.0000 | 0.1958 | 0.0699 | 0.2600 | 0.0000 | 0.0000 | 0.0171 | 0.0000 | 0.0000 | 0.0000 |
| TCGA-E9-A24A-01 | 0.0003 | 0.0000 | 0.0051 | 0.0000 | 0.0000 | 0.2624 | 0.0000 | 0.0311 | 0.0230 | 0.0000 | 0.0408 | 0.0000 | 0.0093 | 0.1240 | 0.0428 | 0.3565 | 0.0000 | 0.0000 | 0.1003 | 0.0000 | 0.0000 | 0.0045 |
| TCGA-E9-A295-01 | 0.0620 | 0.0000 | 0.0298 | 0.0000 | 0.0000 | 0.1480 | 0.0000 | 0.0690 | 0.0714 | 0.0000 | 0.0282 | 0.0000 | 0.0300 | 0.0602 | 0.1007 | 0.2291 | 0.0127 | 0.0000 | 0.1589 | 0.0000 | 0.0000 | 0.0000 |
| TCGA-E9-A2JS-01 | 0.0632 | 0.0000 | 0.0033 | 0.0389 | 0.0000 | 0.0865 | 0.0000 | 0.0444 | 0.0695 | 0.0000 | 0.0253 | 0.0000 | 0.0000 | 0.1613 | 0.1000 | 0.2245 | 0.0000 | 0.0000 | 0.1830 | 0.0000 | 0.0000 | 0.0000 |
| TCGA-E9-A2JT-01 | 0.2713 | 0.1749 | 0.0000 | 0.1595 | 0.0000 | 0.1536 | 0.0128 | 0.0515 | 0.0100 | 0.0000 | 0.0112 | 0.0000 | 0.0022 | 0.0371 | 0.0448 | 0.0563 | 0.0000 | 0.0092 | 0.0057 | 0.0000 | 0.0000 | 0.0000 |
| TCGA-E9-A3HO-01 | 0.0358 | 0.0000 | 0.0000 | 0.0592 | 0.0000 | 0.0543 | 0.0000 | 0.1252 | 0.0781 | 0.0000 | 0.0679 | 0.0226 | 0.0600 | 0.1371 | 0.1585 | 0.0788 | 0.0115 | 0.0000 | 0.1112 | 0.0000 | 0.0000 | 0.0000 |
| TCGA-E9-A3Q9-01 | 0.0779 | 0.0000 | 0.0504 | 0.1031 | 0.0000 | 0.1021 | 0.0000 | 0.0549 | 0.0000 | 0.0000 | 0.0000 | 0.0127 | 0.1162 | 0.0000 | 0.0274 | 0.4090 | 0.0000 | 0.0000 | 0.0462 | 0.0000 | 0.0000 | 0.0000 |
| TCGA-E9-A3QA-01 | 0.0236 | 0.0520 | 0.0000 | 0.0546 | 0.0000 | 0.0902 | 0.0135 | 0.1003 | 0.0862 | 0.0000 | 0.0669 | 0.0000 | 0.0000 | 0.2023 | 0.1884 | 0.0742 | 0.0000 | 0.0333 | 0.0145 | 0.0000 | 0.0000 | 0.0000 |
| TCGA-E9-A3X8-01 | 0.0139 | 0.0000 | 0.1008 | 0.2546 | 0.0000 | 0.1141 | 0.0000 | 0.0881 | 0.0754 | 0.0000 | 0.0424 | 0.0135 | 0.0145 | 0.0001 | 0.0693 | 0.1770 | 0.0001 | 0.0000 | 0.0361 | 0.0000 | 0.0000 | 0.0000 |
| TCGA-E9-A54X-01 | 0.0196 | 0.0000 | 0.0968 | 0.0000 | 0.0000 | 0.1959 | 0.0000 | 0.0479 | 0.0017 | 0.0000 | 0.0574 | 0.0000 | 0.0000 | 0.4240 | 0.0000 | 0.1105 | 0.0000 | 0.0000 | 0.0382 | 0.0000 | 0.0079 | 0.0000 |
| TCGA-E9-A54Y-01 | 0.0991 | 0.0000 | 0.0000 | 0.0000 | 0.0000 | 0.2346 | 0.0000 | 0.1461 | 0.0767 | 0.0000 | 0.0820 | 0.0000 | 0.0012 | 0.2384 | 0.0170 | 0.0741 | 0.0000 | 0.0000 | 0.0000 | 0.0252 | 0.0000 | 0.0056 |
| TCGA-E9-A5FK-01 | 0.0418 | 0.0479 | 0.0000 | 0.2294 | 0.0000 | 0.1236 | 0.0000 | 0.1224 | 0.1109 | 0.0000 | 0.0000 | 0.0121 | 0.0120 | 0.0186 | 0.0620 | 0.1610 | 0.0000 | 0.0166 | 0.0416 | 0.0000 | 0.0000 | 0.0000 |
| TCGA-E9-A5FL-01 | 0.0000 | 0.0028 | 0.0248 | 0.0216 | 0.0000 | 0.1583 | 0.0000 | 0.0328 | 0.0405 | 0.0000 | 0.0155 | 0.0081 | 0.0000 | 0.0432 | 0.0229 | 0.5777 | 0.0015 | 0.0195 | 0.0309 | 0.0000 | 0.0000 | 0.0000 |
| TCGA-E9-A5UO-01 | 0.0019 | 0.0075 | 0.0044 | 0.0000 | 0.0000 | 0.3551 | 0.0000 | 0.0647 | 0.0794 | 0.0000 | 0.0658 | 0.0000 | 0.0203 | 0.2507 | 0.0052 | 0.1257 | 0.0000 | 0.0000 | 0.0193 | 0.0000 | 0.0000 | 0.0000 |
| TCGA-E9-A5UP-01 | 0.0005 | 0.0096 | 0.0000 | 0.0000 | 0.0000 | 0.0000 | 0.0000 | 0.0797 | 0.1229 | 0.0000 | 0.0606 | 0.0006 | 0.0000 | 0.5699 | 0.0000 | 0.0000 | 0.0000 | 0.1001 | 0.0560 | 0.0000 | 0.0000 | 0.0000 |
| TCGA-E9-A6HE-01 | 0.0031 | 0.0014 | 0.0000 | 0.1131 | 0.0000 | 0.0000 | 0.0000 | 0.2056 | 0.0510 | 0.0000 | 0.0355 | 0.0000 | 0.0233 | 0.2094 | 0.0383 | 0.2359 | 0.0007 | 0.0000 | 0.0826 | 0.0000 | 0.0000 | 0.0000 |
| TCGA-EW-A1IW-01 | 0.1322 | 0.0000 | 0.0310 | 0.0308 | 0.0000 | 0.2185 | 0.0000 | 0.0697 | 0.0153 | 0.0000 | 0.0178 | 0.0000 | 0.0000 | 0.0081 | 0.0802 | 0.2784 | 0.0375 | 0.0086 | 0.0719 | 0.0000 | 0.0000 | 0.0000 |
| TCGA-EW-A1IX-01 | 0.0833 | 0.0000 | 0.0237 | 0.0105 | 0.0000 | 0.1490 | 0.0000 | 0.0528 | 0.0394 | 0.0000 | 0.0417 | 0.0000 | 0.0016 | 0.2037 | 0.0058 | 0.2244 | 0.0000 | 0.0163 | 0.0552 | 0.0925 | 0.0000 | 0.0000 |
| TCGA-EW-A1IY-01 | 0.1222 | 0.0000 | 0.0359 | 0.0000 | 0.0000 | 0.1891 | 0.0000 | 0.1273 | 0.0173 | 0.1042 | 0.0000 | 0.0000 | 0.0000 | 0.0053 | 0.1586 | 0.0878 | 0.0647 | 0.0336 | 0.0540 | 0.0000 | 0.0000 | 0.0000 |
| TCGA-EW-A1IZ-01 | 0.0875 | 0.0000 | 0.0692 | 0.3034 | 0.0000 | 0.0045 | 0.0221 | 0.1757 | 0.0116 | 0.0539 | 0.0000 | 0.0029 | 0.0000 | 0.0341 | 0.0787 | 0.1295 | 0.0000 | 0.0038 | 0.0229 | 0.0000 | 0.0000 | 0.0000 |
| TCGA-EW-A1J1-01 | 0.0848 | 0.0000 | 0.0395 | 0.0536 | 0.0000 | 0.1760 | 0.0000 | 0.1031 | 0.0401 | 0.0000 | 0.0000 | 0.0178 | 0.0000 | 0.1149 | 0.0844 | 0.2376 | 0.0000 | 0.0000 | 0.0482 | 0.0000 | 0.0000 | 0.0000 |
| TCGA-EW-A1J2-01 | 0.0448 | 0.0000 | 0.0220 | 0.0741 | 0.0000 | 0.1409 | 0.0000 | 0.0771 | 0.0000 | 0.0000 | 0.0192 | 0.0000 | 0.0069 | 0.1441 | 0.0592 | 0.3114 | 0.0011 | 0.0000 | 0.0991 | 0.0000 | 0.0000 | 0.0000 |
| TCGA-EW-A1J3-01 | 0.0235 | 0.0000 | 0.0123 | 0.0000 | 0.0000 | 0.2706 | 0.0000 | 0.1778 | 0.0354 | 0.0000 | 0.0016 | 0.0216 | 0.0265 | 0.0921 | 0.0514 | 0.1820 | 0.0081 | 0.0440 | 0.0371 | 0.0081 | 0.0021 | 0.0056 |
| TCGA-EW-A1J5-01 | 0.0928 | 0.0000 | 0.0062 | 0.0573 | 0.0000 | 0.1740 | 0.0137 | 0.1245 | 0.0091 | 0.0000 | 0.0090 | 0.0000 | 0.0406 | 0.0048 | 0.0803 | 0.2828 | 0.0000 | 0.0207 | 0.0841 | 0.0000 | 0.0000 | 0.0000 |
| TCGA-EW-A1J6-01 | 0.1111 | 0.0000 | 0.0058 | 0.0237 | 0.0000 | 0.1978 | 0.0130 | 0.1137 | 0.0168 | 0.0000 | 0.0497 | 0.0000 | 0.0251 | 0.0000 | 0.1259 | 0.2946 | 0.0000 | 0.0008 | 0.0221 | 0.0000 | 0.0000 | 0.0000 |
| TCGA-EW-A1OV-01 | 0.1315 | 0.0068 | 0.0000 | 0.1199 | 0.0000 | 0.2020 | 0.0060 | 0.0761 | 0.0604 | 0.0000 | 0.0155 | 0.0000 | 0.0000 | 0.1653 | 0.1159 | 0.0848 | 0.0000 | 0.0000 | 0.0156 | 0.0000 | 0.0000 | 0.0000 |
| TCGA-EW-A1OW-01 | 0.0407 | 0.0000 | 0.0762 | 0.0000 | 0.0000 | 0.0797 | 0.0000 | 0.1538 | 0.0055 | 0.0000 | 0.0024 | 0.0151 | 0.0000 | 0.3655 | 0.0946 | 0.1243 | 0.0000 | 0.0000 | 0.0423 | 0.0000 | 0.0000 | 0.0000 |
| TCGA-EW-A1OX-01 | 0.0000 | 0.0081 | 0.0454 | 0.0000 | 0.0000 | 0.2112 | 0.0000 | 0.0365 | 0.0364 | 0.0000 | 0.0429 | 0.0000 | 0.0206 | 0.3078 | 0.0000 | 0.0399 | 0.0030 | 0.0000 | 0.2483 | 0.0000 | 0.0000 | 0.0000 |
| TCGA-EW-A1OY-01 | 0.0863 | 0.0000 | 0.0949 | 0.0162 | 0.0000 | 0.1446 | 0.0000 | 0.0759 | 0.0306 | 0.0000 | 0.0170 | 0.0000 | 0.0000 | 0.1650 | 0.1308 | 0.2068 | 0.0000 | 0.0000 | 0.0319 | 0.0000 | 0.0000 | 0.0000 |
| TCGA-EW-A1OZ-01 | 0.0609 | 0.0000 | 0.0381 | 0.0222 | 0.0000 | 0.1024 | 0.0044 | 0.0299 | 0.0342 | 0.0000 | 0.0101 | 0.0000 | 0.0000 | 0.2747 | 0.0546 | 0.3037 | 0.0000 | 0.0000 | 0.0647 | 0.0000 | 0.0000 | 0.0000 |
| TCGA-EW-A1P0-01 | 0.0798 | 0.0000 | 0.0265 | 0.0304 | 0.0000 | 0.2065 | 0.0067 | 0.0456 | 0.0283 | 0.0000 | 0.0147 | 0.0000 | 0.0000 | 0.2092 | 0.0693 | 0.2609 | 0.0000 | 0.0000 | 0.0220 | 0.0000 | 0.0000 | 0.0000 |
| TCGA-EW-A1P1-01 | 0.0474 | 0.0000 | 0.0000 | 0.0496 | 0.0000 | 0.1724 | 0.0000 | 0.0755 | 0.0174 | 0.0000 | 0.0198 | 0.0000 | 0.0000 | 0.2644 | 0.0880 | 0.2333 | 0.0000 | 0.0020 | 0.0301 | 0.0000 | 0.0000 | 0.0000 |
| TCGA-EW-A1P3-01 | 0.0786 | 0.0000 | 0.0783 | 0.0351 | 0.0000 | 0.1201 | 0.0000 | 0.1384 | 0.0081 | 0.0000 | 0.0515 | 0.0000 | 0.0214 | 0.0000 | 0.1286 | 0.2590 | 0.0024 | 0.0000 | 0.0784 | 0.0000 | 0.0000 | 0.0000 |
| TCGA-EW-A1P4-01 | 0.0213 | 0.0000 | 0.0103 | 0.0237 | 0.0000 | 0.2300 | 0.0234 | 0.1519 | 0.0172 | 0.0000 | 0.0061 | 0.1151 | 0.0116 | 0.1205 | 0.1024 | 0.0598 | 0.0098 | 0.0042 | 0.0802 | 0.0000 | 0.0000 | 0.0126 |
| TCGA-EW-A1P5-01 | 0.0343 | 0.0000 | 0.0020 | 0.0000 | 0.0000 | 0.1259 | 0.0000 | 0.0609 | 0.0471 | 0.0000 | 0.0309 | 0.0000 | 0.0000 | 0.3022 | 0.0590 | 0.2606 | 0.0000 | 0.0184 | 0.0556 | 0.0000 | 0.0000 | 0.0031 |
| TCGA-EW-A1P6-01 | 0.1543 | 0.0000 | 0.1239 | 0.0492 | 0.0000 | 0.1041 | 0.0000 | 0.0701 | 0.0000 | 0.0000 | 0.0147 | 0.0000 | 0.0000 | 0.0279 | 0.0432 | 0.3299 | 0.0000 | 0.0000 | 0.0827 | 0.0000 | 0.0000 | 0.0000 |
| TCGA-EW-A1P7-01 | 0.1604 | 0.1784 | 0.0000 | 0.1395 | 0.0000 | 0.0388 | 0.0000 | 0.1545 | 0.0100 | 0.0000 | 0.0203 | 0.0000 | 0.0000 | 0.1279 | 0.0637 | 0.1006 | 0.0000 | 0.0000 | 0.0058 | 0.0000 | 0.0000 | 0.0000 |
| TCGA-EW-A1P8-01 | 0.0297 | 0.0000 | 0.0079 | 0.0289 | 0.0000 | 0.1119 | 0.0000 | 0.0875 | 0.0226 | 0.0000 | 0.0090 | 0.0000 | 0.0216 | 0.1081 | 0.0614 | 0.3850 | 0.1153 | 0.0000 | 0.0000 | 0.0097 | 0.0000 | 0.0014 |
| TCGA-EW-A1PA-01 | 0.0738 | 0.0000 | 0.0508 | 0.0549 | 0.0000 | 0.1125 | 0.0000 | 0.0955 | 0.0007 | 0.0000 | 0.0229 | 0.0000 | 0.0000 | 0.1388 | 0.1028 | 0.1438 | 0.0000 | 0.0000 | 0.2035 | 0.0000 | 0.0000 | 0.0000 |
| TCGA-EW-A1PB-01 | 0.0358 | 0.0000 | 0.0000 | 0.0776 | 0.0000 | 0.0977 | 0.0000 | 0.0842 | 0.0137 | 0.0000 | 0.0497 | 0.0000 | 0.0023 | 0.0513 | 0.3511 | 0.1758 | 0.0000 | 0.0000 | 0.0608 | 0.0000 | 0.0000 | 0.0000 |
| TCGA-EW-A1PC-01 | 0.0275 | 0.0000 | 0.0000 | 0.0632 | 0.0000 | 0.0728 | 0.0000 | 0.2313 | 0.0046 | 0.0000 | 0.0000 | 0.0676 | 0.0265 | 0.0000 | 0.0138 | 0.1797 | 0.0000 | 0.2380 | 0.0684 | 0.0000 | 0.0000 | 0.0065 |
| TCGA-EW-A1PD-01 | 0.0860 | 0.0000 | 0.0000 | 0.0089 | 0.0000 | 0.1130 | 0.0000 | 0.0000 | 0.0228 | 0.0000 | 0.0207 | 0.0000 | 0.0000 | 0.4333 | 0.0466 | 0.2161 | 0.0000 | 0.0000 | 0.0527 | 0.0000 | 0.0000 | 0.0000 |
| TCGA-EW-A1PE-01 | 0.0431 | 0.0000 | 0.0594 | 0.0431 | 0.0000 | 0.1246 | 0.0000 | 0.1003 | 0.0159 | 0.0000 | 0.0278 | 0.0000 | 0.0000 | 0.1687 | 0.1186 | 0.2504 | 0.0000 | 0.0000 | 0.0480 | 0.0000 | 0.0000 | 0.0000 |
| TCGA-EW-A1PF-01 | 0.0921 | 0.0000 | 0.0587 | 0.0000 | 0.0000 | 0.1036 | 0.0000 | 0.0670 | 0.0254 | 0.0000 | 0.0265 | 0.0000 | 0.0000 | 0.2375 | 0.0709 | 0.2669 | 0.0000 | 0.0000 | 0.0514 | 0.0000 | 0.0000 | 0.0000 |
| TCGA-EW-A1PG-01 | 0.0258 | 0.0000 | 0.0565 | 0.0000 | 0.0000 | 0.3135 | 0.0000 | 0.1712 | 0.0197 | 0.0000 | 0.0001 | 0.0000 | 0.0760 | 0.1776 | 0.0000 | 0.0000 | 0.0000 | 0.1256 | 0.0295 | 0.0000 | 0.0044 | 0.0000 |
| TCGA-EW-A1PH-01 | 0.1352 | 0.0000 | 0.1095 | 0.0553 | 0.0000 | 0.1102 | 0.0136 | 0.1376 | 0.0000 | 0.0000 | 0.0000 | 0.0049 | 0.0007 | 0.1600 | 0.0626 | 0.1357 | 0.0000 | 0.0734 | 0.0000 | 0.0000 | 0.0000 | 0.0011 |
| TCGA-EW-A2FR-01 | 0.0250 | 0.0000 | 0.0321 | 0.0405 | 0.0000 | 0.1110 | 0.0000 | 0.0358 | 0.0295 | 0.0000 | 0.0159 | 0.0000 | 0.0000 | 0.3983 | 0.0336 | 0.2463 | 0.0000 | 0.0000 | 0.0319 | 0.0000 | 0.0000 | 0.0000 |
| TCGA-EW-A2FS-01 | 0.1399 | 0.0000 | 0.2347 | 0.1152 | 0.0000 | 0.0903 | 0.0000 | 0.1286 | 0.0062 | 0.0080 | 0.0000 | 0.0000 | 0.0000 | 0.0388 | 0.0678 | 0.1625 | 0.0000 | 0.0000 | 0.0000 | 0.0079 | 0.0000 | 0.0000 |
| TCGA-EW-A2FV-01 | 0.0343 | 0.0000 | 0.0319 | 0.0000 | 0.0000 | 0.3190 | 0.0000 | 0.0609 | 0.0000 | 0.0000 | 0.0000 | 0.0233 | 0.0308 | 0.0885 | 0.1374 | 0.1545 | 0.0107 | 0.0000 | 0.1088 | 0.0000 | 0.0000 | 0.0000 |
| TCGA-EW-A2FW-01 | 0.0620 | 0.0000 | 0.0556 | 0.0000 | 0.0000 | 0.0462 | 0.0000 | 0.0571 | 0.0183 | 0.0000 | 0.0398 | 0.0000 | 0.0000 | 0.1995 | 0.0448 | 0.3829 | 0.0000 | 0.0000 | 0.0938 | 0.0000 | 0.0000 | 0.0000 |
| TCGA-EW-A3E8-01 | 0.0558 | 0.0421 | 0.0000 | 0.0795 | 0.0000 | 0.0849 | 0.0000 | 0.1186 | 0.0969 | 0.0000 | 0.0446 | 0.0031 | 0.0317 | 0.1365 | 0.1346 | 0.0536 | 0.0012 | 0.0000 | 0.1169 | 0.0000 | 0.0000 | 0.0000 |
| TCGA-EW-A3U0-01 | 0.0737 | 0.0000 | 0.0088 | 0.1180 | 0.0000 | 0.0550 | 0.0089 | 0.1501 | 0.0612 | 0.0000 | 0.0291 | 0.0142 | 0.0446 | 0.1538 | 0.1136 | 0.1220 | 0.0000 | 0.0000 | 0.0469 | 0.0000 | 0.0000 | 0.0000 |
| TCGA-EW-A423-01 | 0.0052 | 0.0000 | 0.0000 | 0.0732 | 0.0000 | 0.2521 | 0.0000 | 0.0154 | 0.0332 | 0.0000 | 0.0632 | 0.0000 | 0.0068 | 0.1235 | 0.0437 | 0.3620 | 0.0000 | 0.0000 | 0.0218 | 0.0000 | 0.0000 | 0.0000 |
| TCGA-EW-A424-01 | 0.1154 | 0.0000 | 0.1450 | 0.0853 | 0.0000 | 0.0082 | 0.0000 | 0.1331 | 0.0000 | 0.0000 | 0.0345 | 0.0000 | 0.0327 | 0.0000 | 0.0088 | 0.2955 | 0.0000 | 0.0000 | 0.1413 | 0.0000 | 0.0000 | 0.0000 |
| TCGA-EW-A6S9-01 | 0.0963 | 0.0000 | 0.1119 | 0.0000 | 0.0000 | 0.0268 | 0.0000 | 0.0338 | 0.0125 | 0.0000 | 0.0052 | 0.0036 | 0.0000 | 0.3186 | 0.0696 | 0.2316 | 0.0000 | 0.0000 | 0.0902 | 0.0000 | 0.0000 | 0.0000 |
| TCGA-EW-A6SA-01 | 0.1046 | 0.0000 | 0.0946 | 0.0375 | 0.0000 | 0.0548 | 0.0000 | 0.0736 | 0.0157 | 0.0000 | 0.0023 | 0.0225 | 0.0000 | 0.2706 | 0.0322 | 0.1607 | 0.0000 | 0.0000 | 0.1309 | 0.0000 | 0.0000 | 0.0000 |
| TCGA-EW-A6SB-01 | 0.0000 | 0.0381 | 0.2255 | 0.0694 | 0.0000 | 0.1666 | 0.0000 | 0.1154 | 0.0723 | 0.0000 | 0.0000 | 0.0533 | 0.0034 | 0.1143 | 0.0449 | 0.0818 | 0.0120 | 0.0000 | 0.0029 | 0.0000 | 0.0000 | 0.0000 |
| TCGA-EW-A6SC-01 | 0.0022 | 0.0000 | 0.0442 | 0.0000 | 0.0000 | 0.2484 | 0.0000 | 0.1391 | 0.0056 | 0.0000 | 0.0143 | 0.0133 | 0.0524 | 0.1666 | 0.0017 | 0.1395 | 0.0287 | 0.0149 | 0.0000 | 0.1194 | 0.0096 | 0.0000 |
| TCGA-EW-A6SD-01 | 0.0504 | 0.0000 | 0.0995 | 0.0494 | 0.0000 | 0.1615 | 0.0000 | 0.0970 | 0.0404 | 0.0000 | 0.0301 | 0.0000 | 0.0058 | 0.1169 | 0.1138 | 0.1903 | 0.0060 | 0.0000 | 0.0390 | 0.0000 | 0.0000 | 0.0000 |
| TCGA-GI-A2C8-01 | 0.0779 | 0.0000 | 0.0003 | 0.0200 | 0.0000 | 0.0711 | 0.0000 | 0.0258 | 0.0100 | 0.0000 | 0.0299 | 0.0000 | 0.0000 | 0.3166 | 0.0326 | 0.3557 | 0.0000 | 0.0000 | 0.0602 | 0.0000 | 0.0000 | 0.0000 |
| TCGA-GI-A2C9-01 | 0.0247 | 0.0000 | 0.0000 | 0.0000 | 0.0000 | 0.1140 | 0.0000 | 0.0706 | 0.0196 | 0.0000 | 0.0000 | 0.0254 | 0.0000 | 0.2214 | 0.0535 | 0.3283 | 0.0349 | 0.0453 | 0.0625 | 0.0000 | 0.0000 | 0.0000 |
| TCGA-GM-A2D9-01 | 0.0780 | 0.0000 | 0.0004 | 0.0494 | 0.0000 | 0.1385 | 0.0000 | 0.0384 | 0.0213 | 0.0000 | 0.0052 | 0.0000 | 0.0000 | 0.2201 | 0.0736 | 0.3416 | 0.0000 | 0.0000 | 0.0335 | 0.0000 | 0.0000 | 0.0000 |
| TCGA-GM-A2DA-01 | 0.0618 | 0.0000 | 0.0523 | 0.1530 | 0.0000 | 0.2736 | 0.0016 | 0.0871 | 0.0553 | 0.0000 | 0.0000 | 0.0196 | 0.0270 | 0.0364 | 0.1175 | 0.0295 | 0.0094 | 0.0000 | 0.0741 | 0.0000 | 0.0016 | 0.0000 |
| TCGA-GM-A2DB-01 | 0.0804 | 0.0216 | 0.0000 | 0.0000 | 0.0000 | 0.0663 | 0.0000 | 0.1283 | 0.0127 | 0.0000 | 0.0297 | 0.0000 | 0.0000 | 0.3728 | 0.1403 | 0.0896 | 0.0000 | 0.0000 | 0.0582 | 0.0000 | 0.0000 | 0.0000 |
| TCGA-GM-A2DC-01 | 0.0817 | 0.0000 | 0.0457 | 0.1115 | 0.0000 | 0.0868 | 0.0000 | 0.0968 | 0.0278 | 0.0000 | 0.0157 | 0.0372 | 0.0183 | 0.0000 | 0.0836 | 0.2834 | 0.0000 | 0.0000 | 0.1114 | 0.0000 | 0.0000 | 0.0000 |
| TCGA-GM-A2DD-01 | 0.0283 | 0.0037 | 0.0000 | 0.0000 | 0.0000 | 0.2724 | 0.0000 | 0.1115 | 0.0488 | 0.0000 | 0.0287 | 0.0175 | 0.0251 | 0.0943 | 0.1234 | 0.1845 | 0.0001 | 0.0269 | 0.0347 | 0.0000 | 0.0000 | 0.0000 |
| TCGA-GM-A2DF-01 | 0.0113 | 0.0315 | 0.1448 | 0.0592 | 0.0000 | 0.1052 | 0.0000 | 0.0754 | 0.0357 | 0.0000 | 0.0038 | 0.0013 | 0.0000 | 0.2884 | 0.0888 | 0.1545 | 0.0000 | 0.0000 | 0.0000 | 0.0000 | 0.0000 | 0.0000 |
| TCGA-GM-A2DH-01 | 0.0691 | 0.0000 | 0.0063 | 0.0553 | 0.0000 | 0.1770 | 0.0018 | 0.1969 | 0.0135 | 0.0000 | 0.0387 | 0.0080 | 0.0216 | 0.1404 | 0.0960 | 0.0636 | 0.0031 | 0.0132 | 0.0955 | 0.0000 | 0.0000 | 0.0000 |
| TCGA-GM-A2DI-01 | 0.0954 | 0.0000 | 0.0000 | 0.1467 | 0.0000 | 0.3332 | 0.0433 | 0.0000 | 0.0009 | 0.0000 | 0.0193 | 0.0000 | 0.0051 | 0.0893 | 0.0976 | 0.1489 | 0.0000 | 0.0000 | 0.0203 | 0.0000 | 0.0000 | 0.0000 |
| TCGA-GM-A2DK-01 | 0.0622 | 0.0000 | 0.0214 | 0.0595 | 0.0000 | 0.0676 | 0.0000 | 0.0403 | 0.0100 | 0.0000 | 0.0000 | 0.0000 | 0.0000 | 0.3969 | 0.0401 | 0.2680 | 0.0000 | 0.0000 | 0.0340 | 0.0000 | 0.0000 | 0.0000 |
| TCGA-GM-A2DL-01 | 0.0384 | 0.0000 | 0.0000 | 0.0000 | 0.0000 | 0.1749 | 0.0000 | 0.0535 | 0.0495 | 0.0000 | 0.0000 | 0.0233 | 0.0000 | 0.2417 | 0.0649 | 0.2829 | 0.0038 | 0.0000 | 0.0671 | 0.0000 | 0.0000 | 0.0000 |
| TCGA-GM-A2DM-01 | 0.0000 | 0.0049 | 0.0000 | 0.0587 | 0.0000 | 0.1752 | 0.0000 | 0.0316 | 0.0619 | 0.0000 | 0.0287 | 0.0000 | 0.0328 | 0.3157 | 0.0000 | 0.1942 | 0.0143 | 0.0302 | 0.0386 | 0.0000 | 0.0131 | 0.0000 |
| TCGA-GM-A2DN-01 | 0.0668 | 0.0000 | 0.0345 | 0.1322 | 0.0000 | 0.0334 | 0.0000 | 0.1475 | 0.0215 | 0.0000 | 0.0079 | 0.0045 | 0.0260 | 0.3050 | 0.0927 | 0.0807 | 0.0000 | 0.0000 | 0.0474 | 0.0000 | 0.0000 | 0.0000 |
| TCGA-GM-A2DO-01 | 0.2386 | 0.0044 | 0.0000 | 0.1714 | 0.0000 | 0.0948 | 0.0231 | 0.0818 | 0.0221 | 0.0606 | 0.0000 | 0.0000 | 0.0000 | 0.1145 | 0.0834 | 0.0826 | 0.0000 | 0.0021 | 0.0205 | 0.0000 | 0.0000 | 0.0000 |
| TCGA-GM-A3NW-01 | 0.0790 | 0.0000 | 0.0179 | 0.0647 | 0.0000 | 0.2452 | 0.0000 | 0.0579 | 0.0000 | 0.0000 | 0.0088 | 0.0000 | 0.0028 | 0.0000 | 0.0736 | 0.2409 | 0.0241 | 0.0000 | 0.1851 | 0.0000 | 0.0000 | 0.0000 |
| TCGA-GM-A3NY-01 | 0.1153 | 0.0000 | 0.0948 | 0.0091 | 0.0000 | 0.1684 | 0.0000 | 0.1708 | 0.0046 | 0.0000 | 0.0266 | 0.0097 | 0.0149 | 0.0016 | 0.1323 | 0.1898 | 0.0000 | 0.0000 | 0.0619 | 0.0000 | 0.0000 | 0.0000 |
| TCGA-GM-A3XG-01 | 0.0035 | 0.0084 | 0.0000 | 0.0000 | 0.0000 | 0.1232 | 0.0000 | 0.2317 | 0.0019 | 0.0000 | 0.0364 | 0.0173 | 0.0000 | 0.4264 | 0.0567 | 0.0399 | 0.0000 | 0.0000 | 0.0545 | 0.0000 | 0.0000 | 0.0000 |
| TCGA-GM-A3XL-01 | 0.0017 | 0.0060 | 0.0710 | 0.1784 | 0.0000 | 0.0813 | 0.0000 | 0.1408 | 0.0030 | 0.0000 | 0.0562 | 0.0091 | 0.0048 | 0.0795 | 0.1404 | 0.1888 | 0.0000 | 0.0078 | 0.0313 | 0.0000 | 0.0000 | 0.0000 |
| TCGA-GM-A3XN-01 | 0.0639 | 0.0000 | 0.0499 | 0.0715 | 0.0000 | 0.1460 | 0.0000 | 0.0819 | 0.0289 | 0.0000 | 0.0183 | 0.0000 | 0.0000 | 0.1722 | 0.0937 | 0.2364 | 0.0000 | 0.0000 | 0.0372 | 0.0000 | 0.0000 | 0.0000 |
| TCGA-GM-A4E0-01 | 0.0661 | 0.0000 | 0.0021 | 0.1819 | 0.0000 | 0.1316 | 0.0000 | 0.0826 | 0.0000 | 0.0000 | 0.0000 | 0.0000 | 0.0751 | 0.0000 | 0.0723 | 0.3694 | 0.0000 | 0.0000 | 0.0189 | 0.0000 | 0.0000 | 0.0000 |
| TCGA-GM-A5PV-01 | 0.0757 | 0.0000 | 0.0001 | 0.0918 | 0.0000 | 0.1962 | 0.0000 | 0.1248 | 0.0183 | 0.0000 | 0.0000 | 0.0132 | 0.0000 | 0.0886 | 0.0702 | 0.2721 | 0.0126 | 0.0061 | 0.0304 | 0.0000 | 0.0000 | 0.0000 |
| TCGA-GM-A5PX-01 | 0.0929 | 0.0000 | 0.0064 | 0.0645 | 0.0000 | 0.1996 | 0.0000 | 0.0925 | 0.0000 | 0.0000 | 0.0000 | 0.0134 | 0.0000 | 0.0248 | 0.0470 | 0.2747 | 0.0000 | 0.0000 | 0.1842 | 0.0000 | 0.0000 | 0.0000 |
| TCGA-HN-A2NL-01 | 0.0516 | 0.0000 | 0.0490 | 0.1573 | 0.0000 | 0.0790 | 0.0000 | 0.1047 | 0.0524 | 0.0000 | 0.0000 | 0.0014 | 0.0000 | 0.2269 | 0.0892 | 0.1585 | 0.0000 | 0.0000 | 0.0299 | 0.0000 | 0.0000 | 0.0000 |
| TCGA-HN-A2OB-01 | 0.1035 | 0.0000 | 0.0655 | 0.0767 | 0.0000 | 0.1026 | 0.0000 | 0.0694 | 0.0165 | 0.0000 | 0.0050 | 0.0066 | 0.0000 | 0.0470 | 0.0281 | 0.2753 | 0.0000 | 0.0000 | 0.2038 | 0.0000 | 0.0000 | 0.0000 |
| TCGA-JL-A3YW-01 | 0.0118 | 0.0000 | 0.0044 | 0.0266 | 0.0000 | 0.1486 | 0.0032 | 0.0361 | 0.0313 | 0.0000 | 0.0578 | 0.0000 | 0.0000 | 0.4424 | 0.0773 | 0.1488 | 0.0000 | 0.0000 | 0.0116 | 0.0000 | 0.0000 | 0.0000 |
| TCGA-JL-A3YX-01 | 0.0954 | 0.0000 | 0.0260 | 0.0612 | 0.0000 | 0.0495 | 0.0000 | 0.0965 | 0.0000 | 0.0000 | 0.0000 | 0.0300 | 0.0094 | 0.0092 | 0.0108 | 0.2300 | 0.0000 | 0.0108 | 0.3711 | 0.0000 | 0.0000 | 0.0000 |
| TCGA-LD-A66U-01 | 0.0211 | 0.0015 | 0.0268 | 0.1970 | 0.0000 | 0.2407 | 0.0000 | 0.0930 | 0.0644 | 0.0000 | 0.0000 | 0.0116 | 0.0206 | 0.0000 | 0.0947 | 0.1692 | 0.0073 | 0.0000 | 0.0522 | 0.0000 | 0.0000 | 0.0000 |
| TCGA-LD-A74U-01 | 0.0022 | 0.0000 | 0.0000 | 0.0000 | 0.0000 | 0.0814 | 0.0000 | 0.0211 | 0.0293 | 0.0000 | 0.0122 | 0.0021 | 0.0000 | 0.4690 | 0.0412 | 0.2041 | 0.0000 | 0.0000 | 0.1374 | 0.0000 | 0.0000 | 0.0000 |
| TCGA-LD-A7W5-01 | 0.1128 | 0.0111 | 0.0000 | 0.1427 | 0.0000 | 0.2186 | 0.0000 | 0.1477 | 0.0656 | 0.0000 | 0.0168 | 0.0023 | 0.0263 | 0.0643 | 0.0700 | 0.1037 | 0.0000 | 0.0000 | 0.0181 | 0.0000 | 0.0000 | 0.0000 |
| TCGA-LD-A7W6-01 | 0.0548 | 0.0000 | 0.0751 | 0.1726 | 0.0000 | 0.0913 | 0.0000 | 0.1049 | 0.0503 | 0.0000 | 0.0366 | 0.0000 | 0.0196 | 0.0000 | 0.0756 | 0.1889 | 0.0000 | 0.0000 | 0.1302 | 0.0000 | 0.0000 | 0.0000 |
| TCGA-LD-A9QF-01 | 0.0211 | 0.0221 | 0.0000 | 0.0603 | 0.0000 | 0.0357 | 0.0000 | 0.0727 | 0.0866 | 0.0000 | 0.0178 | 0.0036 | 0.0000 | 0.4722 | 0.0668 | 0.0914 | 0.0000 | 0.0000 | 0.0498 | 0.0000 | 0.0000 | 0.0000 |
| TCGA-LL-A440-01 | 0.0279 | 0.0000 | 0.0000 | 0.0102 | 0.0000 | 0.0903 | 0.0000 | 0.0284 | 0.0130 | 0.0000 | 0.0250 | 0.0000 | 0.0000 | 0.4426 | 0.0179 | 0.2617 | 0.0018 | 0.0000 | 0.0811 | 0.0000 | 0.0000 | 0.0000 |
| TCGA-LL-A441-01 | 0.2422 | 0.1485 | 0.0000 | 0.0496 | 0.0000 | 0.0314 | 0.0001 | 0.1319 | 0.0018 | 0.0000 | 0.0610 | 0.0000 | 0.0000 | 0.1895 | 0.0636 | 0.0568 | 0.0000 | 0.0000 | 0.0237 | 0.0000 | 0.0000 | 0.0000 |
| TCGA-LL-A442-01 | 0.0011 | 0.0000 | 0.0000 | 0.0000 | 0.0000 | 0.2200 | 0.0000 | 0.0195 | 0.0424 | 0.0000 | 0.0091 | 0.0000 | 0.0451 | 0.0000 | 0.0000 | 0.5518 | 0.0033 | 0.0000 | 0.1032 | 0.0000 | 0.0000 | 0.0045 |
| TCGA-LL-A50Y-01 | 0.0461 | 0.0292 | 0.0153 | 0.0882 | 0.0000 | 0.1060 | 0.0000 | 0.0985 | 0.0168 | 0.0000 | 0.0000 | 0.0224 | 0.0030 | 0.0981 | 0.0245 | 0.3415 | 0.0337 | 0.0036 | 0.0719 | 0.0000 | 0.0000 | 0.0011 |
| TCGA-LL-A5YL-01 | 0.0954 | 0.0000 | 0.0065 | 0.1055 | 0.0000 | 0.1479 | 0.0000 | 0.0756 | 0.0229 | 0.0000 | 0.0000 | 0.0428 | 0.0130 | 0.0000 | 0.0448 | 0.3451 | 0.0000 | 0.0000 | 0.0934 | 0.0000 | 0.0000 | 0.0071 |
| TCGA-LL-A5YM-01 | 0.0034 | 0.0065 | 0.0211 | 0.0724 | 0.0018 | 0.0000 | 0.0000 | 0.0305 | 0.0693 | 0.0026 | 0.0473 | 0.0000 | 0.0000 | 0.3579 | 0.0486 | 0.2346 | 0.0000 | 0.0000 | 0.0848 | 0.0191 | 0.0000 | 0.0000 |
| TCGA-LL-A5YN-01 | 0.1081 | 0.0554 | 0.0000 | 0.1255 | 0.0000 | 0.2440 | 0.0000 | 0.0547 | 0.0993 | 0.0000 | 0.0000 | 0.0200 | 0.0000 | 0.1205 | 0.0721 | 0.0882 | 0.0000 | 0.0000 | 0.0122 | 0.0000 | 0.0000 | 0.0000 |
| TCGA-LL-A5YO-01 | 0.1129 | 0.0204 | 0.0000 | 0.0957 | 0.0000 | 0.1379 | 0.0000 | 0.0835 | 0.1083 | 0.0000 | 0.0248 | 0.0123 | 0.0191 | 0.1606 | 0.1368 | 0.0701 | 0.0000 | 0.0000 | 0.0175 | 0.0000 | 0.0000 | 0.0000 |
| TCGA-LL-A5YP-01 | 0.0422 | 0.0000 | 0.0441 | 0.0219 | 0.0000 | 0.1551 | 0.0000 | 0.0656 | 0.0473 | 0.0000 | 0.0246 | 0.0000 | 0.0191 | 0.1501 | 0.0640 | 0.3490 | 0.0000 | 0.0024 | 0.0147 | 0.0000 | 0.0000 | 0.0000 |
| TCGA-LL-A6FP-01 | 0.0041 | 0.0000 | 0.1601 | 0.0627 | 0.0299 | 0.0428 | 0.0000 | 0.0256 | 0.0559 | 0.0000 | 0.0245 | 0.0000 | 0.0906 | 0.1768 | 0.0000 | 0.1677 | 0.0316 | 0.0000 | 0.1276 | 0.0000 | 0.0000 | 0.0000 |
| TCGA-LL-A6FQ-01 | 0.0402 | 0.0000 | 0.0000 | 0.0368 | 0.0000 | 0.0631 | 0.0000 | 0.0557 | 0.0046 | 0.0000 | 0.0145 | 0.0048 | 0.0427 | 0.3867 | 0.0201 | 0.3039 | 0.0000 | 0.0000 | 0.0270 | 0.0000 | 0.0000 | 0.0000 |
| TCGA-LL-A6FR-01 | 0.0015 | 0.0000 | 0.0293 | 0.0220 | 0.0000 | 0.0000 | 0.0000 | 0.0478 | 0.0513 | 0.0000 | 0.0681 | 0.0000 | 0.0000 | 0.6200 | 0.0000 | 0.1513 | 0.0000 | 0.0000 | 0.0060 | 0.0026 | 0.0000 | 0.0000 |
| TCGA-LL-A73Y-01 | 0.0385 | 0.0285 | 0.0006 | 0.0000 | 0.0000 | 0.1430 | 0.0000 | 0.0752 | 0.0403 | 0.0000 | 0.0000 | 0.0133 | 0.0000 | 0.4838 | 0.0029 | 0.0910 | 0.0000 | 0.0592 | 0.0237 | 0.0000 | 0.0000 | 0.0000 |
| TCGA-LL-A73Z-01 | 0.0450 | 0.0000 | 0.0199 | 0.0660 | 0.0000 | 0.1077 | 0.0000 | 0.1039 | 0.0562 | 0.0000 | 0.0187 | 0.0000 | 0.0211 | 0.0254 | 0.2027 | 0.2678 | 0.0007 | 0.0000 | 0.0650 | 0.0000 | 0.0000 | 0.0000 |
| TCGA-LL-A740-01 | 0.0423 | 0.0000 | 0.0061 | 0.0000 | 0.0000 | 0.1029 | 0.0000 | 0.0379 | 0.0298 | 0.0000 | 0.0260 | 0.0000 | 0.0000 | 0.3735 | 0.0017 | 0.2623 | 0.0384 | 0.0436 | 0.0354 | 0.0000 | 0.0000 | 0.0000 |
| TCGA-LL-A7SZ-01 | 0.0155 | 0.0008 | 0.2812 | 0.1056 | 0.0000 | 0.0428 | 0.0000 | 0.1336 | 0.0364 | 0.0000 | 0.0304 | 0.0000 | 0.0008 | 0.0842 | 0.0295 | 0.2264 | 0.0000 | 0.0000 | 0.0129 | 0.0000 | 0.0000 | 0.0000 |
| TCGA-LL-A7T0-01 | 0.0136 | 0.0054 | 0.0169 | 0.0658 | 0.0000 | 0.0150 | 0.0000 | 0.1050 | 0.1045 | 0.0000 | 0.0338 | 0.0120 | 0.0107 | 0.3592 | 0.0447 | 0.1534 | 0.0000 | 0.0000 | 0.0601 | 0.0000 | 0.0000 | 0.0000 |
| TCGA-LL-A8F5-01 | 0.0284 | 0.0000 | 0.1271 | 0.0000 | 0.0000 | 0.0521 | 0.0000 | 0.1798 | 0.0158 | 0.0000 | 0.0322 | 0.0000 | 0.0000 | 0.3534 | 0.0799 | 0.1000 | 0.0000 | 0.0000 | 0.0312 | 0.0000 | 0.0000 | 0.0000 |
| TCGA-LL-A9Q3-01 | 0.0485 | 0.0000 | 0.0413 | 0.1132 | 0.0000 | 0.2047 | 0.0000 | 0.0167 | 0.0453 | 0.0000 | 0.0256 | 0.0000 | 0.0069 | 0.0075 | 0.0623 | 0.3029 | 0.0000 | 0.0111 | 0.1140 | 0.0000 | 0.0000 | 0.0000 |
| TCGA-LQ-A4E4-01 | 0.0011 | 0.0005 | 0.0000 | 0.0000 | 0.0000 | 0.3415 | 0.0000 | 0.0224 | 0.0718 | 0.0000 | 0.0958 | 0.0000 | 0.0539 | 0.0586 | 0.0081 | 0.1353 | 0.0000 | 0.0000 | 0.2109 | 0.0000 | 0.0000 | 0.0000 |
| TCGA-MS-A51U-01 | 0.0727 | 0.0000 | 0.0626 | 0.1511 | 0.0000 | 0.0891 | 0.0000 | 0.1343 | 0.0424 | 0.0000 | 0.0436 | 0.0000 | 0.0424 | 0.0409 | 0.0876 | 0.1725 | 0.0000 | 0.0000 | 0.0608 | 0.0000 | 0.0000 | 0.0000 |
| TCGA-OK-A5Q2-01 | 0.1906 | 0.0263 | 0.0299 | 0.1247 | 0.0000 | 0.1160 | 0.0000 | 0.1267 | 0.0635 | 0.0000 | 0.0228 | 0.0000 | 0.0000 | 0.0651 | 0.0444 | 0.1679 | 0.0000 | 0.0000 | 0.0197 | 0.0000 | 0.0000 | 0.0024 |
| TCGA-OL-A5D6-01 | 0.1029 | 0.0000 | 0.0840 | 0.0367 | 0.0000 | 0.1174 | 0.0000 | 0.0671 | 0.0246 | 0.0000 | 0.0147 | 0.0000 | 0.0000 | 0.0204 | 0.0541 | 0.3877 | 0.0326 | 0.0000 | 0.0578 | 0.0000 | 0.0000 | 0.0000 |
| TCGA-OL-A5D7-01 | 0.0612 | 0.0257 | 0.0190 | 0.1580 | 0.0000 | 0.0616 | 0.0455 | 0.2204 | 0.0247 | 0.0000 | 0.0398 | 0.0000 | 0.0041 | 0.0870 | 0.1215 | 0.1108 | 0.0000 | 0.0083 | 0.0124 | 0.0000 | 0.0000 | 0.0000 |
| TCGA-OL-A5D8-01 | 0.0240 | 0.0000 | 0.0082 | 0.0635 | 0.0000 | 0.0043 | 0.0000 | 0.2042 | 0.0519 | 0.0000 | 0.0325 | 0.0083 | 0.0119 | 0.2390 | 0.0586 | 0.2355 | 0.0000 | 0.0000 | 0.0581 | 0.0000 | 0.0000 | 0.0000 |
| TCGA-OL-A5DA-01 | 0.0908 | 0.0000 | 0.0621 | 0.1206 | 0.0000 | 0.1493 | 0.0000 | 0.2013 | 0.0076 | 0.0043 | 0.0000 | 0.0000 | 0.0000 | 0.0097 | 0.0902 | 0.2169 | 0.0000 | 0.0000 | 0.0471 | 0.0000 | 0.0000 | 0.0000 |
| TCGA-OL-A5RU-01 | 0.0938 | 0.0670 | 0.0000 | 0.1778 | 0.0000 | 0.1194 | 0.0193 | 0.1370 | 0.0367 | 0.0000 | 0.0184 | 0.0000 | 0.0000 | 0.1006 | 0.0514 | 0.1356 | 0.0000 | 0.0024 | 0.0407 | 0.0000 | 0.0000 | 0.0000 |
| TCGA-OL-A5RV-01 | 0.1891 | 0.0000 | 0.0557 | 0.0907 | 0.0000 | 0.0476 | 0.0000 | 0.1133 | 0.0000 | 0.0000 | 0.0106 | 0.0000 | 0.0015 | 0.0071 | 0.0331 | 0.2465 | 0.0000 | 0.0000 | 0.2048 | 0.0000 | 0.0000 | 0.0000 |
| TCGA-OL-A5RW-01 | 0.0312 | 0.0000 | 0.0469 | 0.0174 | 0.0000 | 0.0954 | 0.0000 | 0.1177 | 0.0000 | 0.0000 | 0.0033 | 0.0147 | 0.0018 | 0.1994 | 0.0556 | 0.2632 | 0.0000 | 0.1269 | 0.0265 | 0.0000 | 0.0000 | 0.0000 |
| TCGA-OL-A5RX-01 | 0.0651 | 0.0000 | 0.0602 | 0.0493 | 0.0000 | 0.1567 | 0.0000 | 0.2240 | 0.0256 | 0.0000 | 0.1123 | 0.0000 | 0.0412 | 0.1041 | 0.0324 | 0.0570 | 0.0000 | 0.0000 | 0.0706 | 0.0000 | 0.0017 | 0.0000 |
| TCGA-OL-A5RY-01 | 0.1485 | 0.0544 | 0.0155 | 0.0648 | 0.0000 | 0.1679 | 0.0000 | 0.1004 | 0.0008 | 0.0000 | 0.0000 | 0.0073 | 0.0106 | 0.0168 | 0.0756 | 0.2952 | 0.0009 | 0.0000 | 0.0415 | 0.0000 | 0.0000 | 0.0000 |
| TCGA-OL-A5RZ-01 | 0.0076 | 0.0034 | 0.0040 | 0.0416 | 0.0000 | 0.0665 | 0.0000 | 0.1529 | 0.0245 | 0.0000 | 0.0258 | 0.0000 | 0.0000 | 0.3785 | 0.0000 | 0.2506 | 0.0000 | 0.0000 | 0.0420 | 0.0000 | 0.0026 | 0.0000 |
| TCGA-OL-A5S0-01 | 0.0183 | 0.0000 | 0.0000 | 0.0000 | 0.0000 | 0.0277 | 0.0000 | 0.1126 | 0.0518 | 0.0021 | 0.0216 | 0.0000 | 0.0000 | 0.4003 | 0.1240 | 0.2041 | 0.0000 | 0.0000 | 0.0375 | 0.0000 | 0.0000 | 0.0000 |
| TCGA-OL-A66H-01 | 0.0428 | 0.0000 | 0.0241 | 0.0415 | 0.0000 | 0.1513 | 0.0065 | 0.0211 | 0.0257 | 0.0000 | 0.0357 | 0.0000 | 0.0372 | 0.0044 | 0.0629 | 0.4771 | 0.0000 | 0.0000 | 0.0642 | 0.0000 | 0.0000 | 0.0054 |
| TCGA-OL-A66I-01 | 0.0000 | 0.0005 | 0.0000 | 0.1928 | 0.0000 | 0.1819 | 0.0166 | 0.0640 | 0.0825 | 0.0000 | 0.0000 | 0.0207 | 0.0042 | 0.1173 | 0.1311 | 0.0940 | 0.0386 | 0.0018 | 0.0540 | 0.0000 | 0.0000 | 0.0000 |
| TCGA-OL-A66J-01 | 0.0219 | 0.0000 | 0.0000 | 0.0442 | 0.0000 | 0.0301 | 0.0000 | 0.1054 | 0.0064 | 0.0000 | 0.0329 | 0.0057 | 0.0000 | 0.3848 | 0.0313 | 0.2770 | 0.0000 | 0.0000 | 0.0602 | 0.0000 | 0.0000 | 0.0000 |
| TCGA-OL-A66K-01 | 0.1134 | 0.0000 | 0.0190 | 0.0531 | 0.0000 | 0.0897 | 0.0000 | 0.0875 | 0.0000 | 0.0000 | 0.0068 | 0.0018 | 0.0000 | 0.1602 | 0.0000 | 0.3232 | 0.0000 | 0.0411 | 0.1042 | 0.0000 | 0.0000 | 0.0000 |
| TCGA-OL-A66L-01 | 0.0335 | 0.0000 | 0.0223 | 0.1302 | 0.0000 | 0.2160 | 0.0000 | 0.0881 | 0.0000 | 0.0000 | 0.0000 | 0.0312 | 0.0290 | 0.0263 | 0.0411 | 0.2878 | 0.0000 | 0.0175 | 0.0763 | 0.0000 | 0.0000 | 0.0007 |
| TCGA-OL-A66N-01 | 0.0054 | 0.0445 | 0.1406 | 0.0997 | 0.0000 | 0.0962 | 0.0000 | 0.0895 | 0.0026 | 0.0000 | 0.0000 | 0.0445 | 0.0116 | 0.0000 | 0.0067 | 0.2534 | 0.0000 | 0.0064 | 0.1989 | 0.0000 | 0.0000 | 0.0000 |
| TCGA-OL-A66O-01 | 0.0526 | 0.0000 | 0.2218 | 0.1210 | 0.0000 | 0.0273 | 0.0000 | 0.0651 | 0.0233 | 0.0000 | 0.0000 | 0.0634 | 0.0102 | 0.0305 | 0.0102 | 0.2724 | 0.0000 | 0.0070 | 0.0951 | 0.0000 | 0.0000 | 0.0000 |
| TCGA-OL-A66P-01 | 0.0195 | 0.0000 | 0.1055 | 0.2478 | 0.0000 | 0.0000 | 0.0000 | 0.0672 | 0.0708 | 0.0000 | 0.0078 | 0.0438 | 0.0190 | 0.1282 | 0.0539 | 0.1758 | 0.0000 | 0.0000 | 0.0606 | 0.0000 | 0.0000 | 0.0000 |
| TCGA-OL-A6VO-01 | 0.0000 | 0.0067 | 0.0000 | 0.0000 | 0.0000 | 0.0676 | 0.0000 | 0.0993 | 0.0411 | 0.0000 | 0.0086 | 0.0121 | 0.0000 | 0.5945 | 0.0596 | 0.1026 | 0.0000 | 0.0052 | 0.0023 | 0.0000 | 0.0004 | 0.0000 |
| TCGA-OL-A6VQ-01 | 0.1375 | 0.0000 | 0.0289 | 0.0472 | 0.0000 | 0.2547 | 0.0060 | 0.0144 | 0.0000 | 0.0000 | 0.0277 | 0.0000 | 0.0195 | 0.0000 | 0.0781 | 0.1365 | 0.0601 | 0.0000 | 0.1826 | 0.0069 | 0.0000 | 0.0000 |
| TCGA-OL-A6VR-01 | 0.0844 | 0.0000 | 0.0438 | 0.0000 | 0.0000 | 0.0760 | 0.0000 | 0.1306 | 0.0326 | 0.0000 | 0.0040 | 0.0274 | 0.0061 | 0.2864 | 0.0184 | 0.2222 | 0.0000 | 0.0000 | 0.0682 | 0.0000 | 0.0000 | 0.0000 |
| TCGA-OL-A97C-01 | 0.0133 | 0.0000 | 0.0007 | 0.1648 | 0.0000 | 0.0236 | 0.0000 | 0.1732 | 0.0018 | 0.0000 | 0.0064 | 0.0030 | 0.0000 | 0.3517 | 0.0803 | 0.1772 | 0.0000 | 0.0000 | 0.0038 | 0.0000 | 0.0000 | 0.0000 |
| TCGA-PE-A5DC-01 | 0.0885 | 0.0000 | 0.0083 | 0.0759 | 0.0000 | 0.2055 | 0.0000 | 0.0336 | 0.0324 | 0.0000 | 0.0259 | 0.0000 | 0.0000 | 0.2448 | 0.0768 | 0.1328 | 0.0000 | 0.0000 | 0.0755 | 0.0000 | 0.0000 | 0.0000 |
| TCGA-PE-A5DD-01 | 0.0003 | 0.0165 | 0.0098 | 0.2105 | 0.0000 | 0.0414 | 0.0000 | 0.0821 | 0.0517 | 0.0000 | 0.0000 | 0.0000 | 0.0269 | 0.1707 | 0.0731 | 0.0431 | 0.0000 | 0.0000 | 0.2740 | 0.0000 | 0.0000 | 0.0000 |
| TCGA-PE-A5DE-01 | 0.0724 | 0.0000 | 0.0237 | 0.2453 | 0.0000 | 0.0724 | 0.0011 | 0.1486 | 0.0463 | 0.0000 | 0.0031 | 0.0140 | 0.0254 | 0.0885 | 0.1520 | 0.0641 | 0.0095 | 0.0000 | 0.0336 | 0.0000 | 0.0000 | 0.0000 |
| TCGA-PL-A8LV-01 | 0.1165 | 0.0000 | 0.0759 | 0.0499 | 0.0000 | 0.0817 | 0.0000 | 0.1786 | 0.0536 | 0.0000 | 0.0034 | 0.0133 | 0.0186 | 0.2004 | 0.1272 | 0.0601 | 0.0000 | 0.0000 | 0.0210 | 0.0000 | 0.0000 | 0.0000 |
| TCGA-PL-A8LX-01 | 0.0020 | 0.0000 | 0.0380 | 0.0000 | 0.0000 | 0.2732 | 0.0000 | 0.0150 | 0.0493 | 0.0000 | 0.0385 | 0.0191 | 0.0284 | 0.0350 | 0.0043 | 0.3554 | 0.0100 | 0.0000 | 0.1310 | 0.0000 | 0.0000 | 0.0008 |
| TCGA-PL-A8LY-01 | 0.0830 | 0.0000 | 0.0906 | 0.1247 | 0.0000 | 0.0454 | 0.0000 | 0.1507 | 0.0220 | 0.0000 | 0.0435 | 0.0139 | 0.0183 | 0.0000 | 0.0104 | 0.3580 | 0.0000 | 0.0000 | 0.0396 | 0.0000 | 0.0000 | 0.0000 |
| TCGA-PL-A8LZ-01 | 0.0350 | 0.0000 | 0.0037 | 0.0000 | 0.0000 | 0.2457 | 0.0000 | 0.1393 | 0.0187 | 0.0000 | 0.0413 | 0.0110 | 0.0087 | 0.0891 | 0.2150 | 0.1330 | 0.0000 | 0.0249 | 0.0346 | 0.0000 | 0.0000 | 0.0000 |
| TCGA-S3-A6ZF-01 | 0.1766 | 0.0000 | 0.0053 | 0.0823 | 0.0000 | 0.0814 | 0.0095 | 0.0569 | 0.0241 | 0.0000 | 0.0041 | 0.0000 | 0.0005 | 0.0404 | 0.0650 | 0.3452 | 0.0000 | 0.0284 | 0.0802 | 0.0000 | 0.0000 | 0.0000 |
| TCGA-S3-A6ZG-01 | 0.0845 | 0.0000 | 0.0951 | 0.0667 | 0.0000 | 0.0999 | 0.0000 | 0.0514 | 0.0258 | 0.0000 | 0.0073 | 0.0182 | 0.0084 | 0.0615 | 0.0361 | 0.2744 | 0.0000 | 0.0000 | 0.1707 | 0.0000 | 0.0000 | 0.0000 |
| TCGA-S3-A6ZH-01 | 0.0749 | 0.0000 | 0.0369 | 0.0906 | 0.0000 | 0.0534 | 0.0000 | 0.0823 | 0.0612 | 0.0000 | 0.0124 | 0.0166 | 0.0298 | 0.0000 | 0.0627 | 0.2071 | 0.0128 | 0.0972 | 0.1622 | 0.0000 | 0.0000 | 0.0000 |
| TCGA-S3-AA0Z-01 | 0.0609 | 0.0000 | 0.0148 | 0.0803 | 0.0000 | 0.0021 | 0.0000 | 0.1622 | 0.0565 | 0.0000 | 0.0244 | 0.0057 | 0.0003 | 0.3047 | 0.0771 | 0.1668 | 0.0000 | 0.0000 | 0.0443 | 0.0000 | 0.0000 | 0.0000 |
| TCGA-S3-AA10-01 | 0.0209 | 0.0284 | 0.0414 | 0.0915 | 0.0000 | 0.0778 | 0.0160 | 0.1963 | 0.0416 | 0.0000 | 0.0000 | 0.0176 | 0.0000 | 0.1667 | 0.1922 | 0.0999 | 0.0000 | 0.0000 | 0.0097 | 0.0000 | 0.0000 | 0.0000 |
| TCGA-S3-AA11-01 | 0.0021 | 0.0048 | 0.0014 | 0.0000 | 0.0000 | 0.2102 | 0.0000 | 0.0940 | 0.0605 | 0.0000 | 0.0673 | 0.0000 | 0.0000 | 0.4293 | 0.0000 | 0.0147 | 0.0000 | 0.0000 | 0.1159 | 0.0000 | 0.0000 | 0.0000 |
| TCGA-S3-AA12-01 | 0.0759 | 0.0000 | 0.1285 | 0.0426 | 0.0000 | 0.0913 | 0.0000 | 0.0509 | 0.0370 | 0.0000 | 0.0255 | 0.0000 | 0.0153 | 0.0836 | 0.0252 | 0.2983 | 0.0000 | 0.0000 | 0.1249 | 0.0000 | 0.0000 | 0.0010 |
| TCGA-S3-AA14-01 | 0.0549 | 0.0000 | 0.0737 | 0.0841 | 0.0000 | 0.1209 | 0.0000 | 0.1315 | 0.0032 | 0.0000 | 0.0490 | 0.0000 | 0.0306 | 0.0113 | 0.0399 | 0.2604 | 0.0000 | 0.0000 | 0.1406 | 0.0000 | 0.0000 | 0.0000 |
| TCGA-S3-AA15-01 | 0.0750 | 0.1383 | 0.2218 | 0.0922 | 0.0000 | 0.0517 | 0.0129 | 0.0646 | 0.0142 | 0.0000 | 0.0201 | 0.0000 | 0.0089 | 0.0672 | 0.0360 | 0.1604 | 0.0000 | 0.0000 | 0.0366 | 0.0000 | 0.0000 | 0.0000 |
| TCGA-S3-AA17-01 | 0.0892 | 0.0000 | 0.0146 | 0.0345 | 0.0000 | 0.1812 | 0.0175 | 0.0909 | 0.0200 | 0.0000 | 0.0783 | 0.0000 | 0.0331 | 0.1623 | 0.1057 | 0.1449 | 0.0000 | 0.0003 | 0.0275 | 0.0000 | 0.0000 | 0.0000 |
| TCGA-UL-AAZ6-01 | 0.0342 | 0.0000 | 0.0612 | 0.0100 | 0.0000 | 0.0786 | 0.0000 | 0.0310 | 0.0086 | 0.0000 | 0.0000 | 0.0173 | 0.0000 | 0.3503 | 0.0483 | 0.3162 | 0.0000 | 0.0000 | 0.0444 | 0.0000 | 0.0000 | 0.0000 |
| TCGA-UU-A93S-01 | 0.0542 | 0.0000 | 0.0692 | 0.0200 | 0.0000 | 0.0000 | 0.0000 | 0.1456 | 0.0096 | 0.0000 | 0.0341 | 0.0300 | 0.0000 | 0.3178 | 0.0424 | 0.2229 | 0.0000 | 0.0081 | 0.0459 | 0.0000 | 0.0000 | 0.0000 |
| TCGA-V7-A7HQ-01 | 0.0772 | 0.0000 | 0.0512 | 0.0359 | 0.0000 | 0.0000 | 0.0000 | 0.0990 | 0.0377 | 0.0000 | 0.0000 | 0.0262 | 0.0000 | 0.1350 | 0.0132 | 0.4359 | 0.0000 | 0.0000 | 0.0886 | 0.0000 | 0.0000 | 0.0000 |
| TCGA-W8-A86G-01 | 0.0021 | 0.0000 | 0.0404 | 0.0000 | 0.0000 | 0.2769 | 0.0000 | 0.0659 | 0.0434 | 0.0000 | 0.1544 | 0.0000 | 0.0557 | 0.1019 | 0.0365 | 0.0161 | 0.0400 | 0.0000 | 0.1669 | 0.0000 | 0.0000 | 0.0000 |
| TCGA-WT-AB41-01 | 0.0545 | 0.0000 | 0.0259 | 0.1493 | 0.0000 | 0.1349 | 0.0000 | 0.1790 | 0.0669 | 0.0000 | 0.0000 | 0.0061 | 0.0023 | 0.0636 | 0.0291 | 0.2403 | 0.0000 | 0.0266 | 0.0213 | 0.0000 | 0.0000 | 0.0000 |
| TCGA-WT-AB44-01 | 0.0502 | 0.0000 | 0.0000 | 0.0693 | 0.0000 | 0.1248 | 0.0000 | 0.0807 | 0.0044 | 0.0000 | 0.0259 | 0.0013 | 0.0014 | 0.1215 | 0.0172 | 0.4057 | 0.0000 | 0.0440 | 0.0534 | 0.0000 | 0.0000 | 0.0000 |
| TCGA-XX-A899-01 | 0.1125 | 0.0751 | 0.0000 | 0.0848 | 0.0000 | 0.1178 | 0.0213 | 0.0468 | 0.0000 | 0.0000 | 0.0244 | 0.0000 | 0.0171 | 0.0860 | 0.0578 | 0.2277 | 0.0000 | 0.0005 | 0.1281 | 0.0000 | 0.0000 | 0.0000 |
| TCGA-XX-A89A-01 | 0.0684 | 0.0000 | 0.0165 | 0.0509 | 0.0000 | 0.1694 | 0.0000 | 0.0870 | 0.0257 | 0.0000 | 0.0114 | 0.0000 | 0.0103 | 0.0704 | 0.0537 | 0.3887 | 0.0000 | 0.0201 | 0.0276 | 0.0000 | 0.0000 | 0.0000 |
| TCGA-Z7-A8R5-01 | 0.0504 | 0.0084 | 0.0000 | 0.1249 | 0.0000 | 0.0000 | 0.0000 | 0.1810 | 0.0053 | 0.0000 | 0.0545 | 0.0000 | 0.0345 | 0.1665 | 0.0193 | 0.3058 | 0.0000 | 0.0000 | 0.0494 | 0.0000 | 0.0000 | 0.0000 |
| TCGA-Z7-A8R6-01 | 0.0522 | 0.0000 | 0.0000 | 0.0543 | 0.0000 | 0.1318 | 0.0000 | 0.0613 | 0.0443 | 0.0000 | 0.0054 | 0.0108 | 0.0000 | 0.2782 | 0.0699 | 0.2191 | 0.0014 | 0.0000 | 0.0713 | 0.0000 | 0.0000 | 0.0000 |

**Supplementary Table 3.** The immunologic infiltrations of METABRIC patients quantitively evaluated by CIBERSORT algorithms

| **Sample** | **B cells naive** | **B cells memory** | **Plasma cells** | **T cells CD8** | **T cells CD4 naive** | **T cells CD4 memory resting** | **T cells CD4 memory activated** | **T cells follicular helper** | **T cells regulatory (Tregs)** | **T cells gamma delta** | **NK cells resting** | **NK cells activated** | **Monocytes** | **Macrophages M0** | **Macrophages M1** | **Macrophages M2** | **Dendritic cells resting** | **Dendritic cells activated** | **Mast cells resting** | **Mast cells activated** | **Eosinophils** | **Neutrophils** |
| --- | --- | --- | --- | --- | --- | --- | --- | --- | --- | --- | --- | --- | --- | --- | --- | --- | --- | --- | --- | --- | --- | --- |
| MB-0362 | 0.0386 | 0.0000 | 0.0047 | 0.0805 | 0.0000 | 0.0000 | 0.0000 | 0.0504 | 0.0000 | 0.0000 | 0.0000 | 0.0503 | 0.0207 | 0.0000 | 0.0211 | 0.1752 | 0.0305 | 0.0000 | 0.4573 | 0.0707 | 0.0000 | 0.0000 |
| MB-0346 | 0.0000 | 0.0342 | 0.0093 | 0.0000 | 0.0000 | 0.0980 | 0.0284 | 0.0657 | 0.0000 | 0.0000 | 0.0076 | 0.0131 | 0.0859 | 0.2308 | 0.0134 | 0.2989 | 0.0000 | 0.0466 | 0.0680 | 0.0000 | 0.0000 | 0.0000 |
| MB-0386 | 0.0105 | 0.0320 | 0.0000 | 0.0527 | 0.0000 | 0.0400 | 0.0000 | 0.0748 | 0.0000 | 0.0752 | 0.0000 | 0.0000 | 0.0214 | 0.1104 | 0.0640 | 0.3986 | 0.0000 | 0.0000 | 0.0000 | 0.1205 | 0.0000 | 0.0000 |
| MB-0574 | 0.0342 | 0.0000 | 0.0000 | 0.0150 | 0.0000 | 0.0000 | 0.0000 | 0.0336 | 0.0235 | 0.0308 | 0.0000 | 0.0207 | 0.0000 | 0.2742 | 0.0304 | 0.3458 | 0.0000 | 0.0000 | 0.1898 | 0.0000 | 0.0000 | 0.0020 |
| MB-0503 | 0.0273 | 0.0000 | 0.0186 | 0.1046 | 0.0000 | 0.0112 | 0.0000 | 0.0678 | 0.0000 | 0.0139 | 0.0000 | 0.0435 | 0.0103 | 0.0931 | 0.0979 | 0.1247 | 0.0000 | 0.0000 | 0.3051 | 0.0819 | 0.0000 | 0.0000 |
| MB-0641 | 0.0379 | 0.0000 | 0.0083 | 0.0693 | 0.0000 | 0.0885 | 0.0251 | 0.0563 | 0.0000 | 0.0524 | 0.0000 | 0.0558 | 0.0621 | 0.0362 | 0.0329 | 0.1014 | 0.0000 | 0.0000 | 0.3737 | 0.0000 | 0.0000 | 0.0000 |
| MB-0201 | 0.0164 | 0.0000 | 0.0337 | 0.0292 | 0.0000 | 0.0000 | 0.0000 | 0.0617 | 0.0089 | 0.0687 | 0.0000 | 0.0284 | 0.0000 | 0.2068 | 0.0453 | 0.3500 | 0.0108 | 0.0000 | 0.1168 | 0.0236 | 0.0000 | 0.0000 |
| MB-0218 | 0.0346 | 0.0000 | 0.0016 | 0.0123 | 0.0000 | 0.0256 | 0.0000 | 0.0335 | 0.0109 | 0.0150 | 0.0000 | 0.0245 | 0.0000 | 0.1991 | 0.0409 | 0.3369 | 0.0000 | 0.0000 | 0.1790 | 0.0749 | 0.0000 | 0.0110 |
| MB-0316 | 0.0184 | 0.0000 | 0.1646 | 0.0944 | 0.0000 | 0.0000 | 0.0058 | 0.0941 | 0.0077 | 0.0538 | 0.0000 | 0.0795 | 0.0037 | 0.1898 | 0.1805 | 0.0632 | 0.0000 | 0.0000 | 0.0444 | 0.0000 | 0.0000 | 0.0000 |
| MB-0189 | 0.0148 | 0.0000 | 0.0289 | 0.1873 | 0.0000 | 0.0474 | 0.0000 | 0.0564 | 0.0004 | 0.0000 | 0.0000 | 0.0522 | 0.0868 | 0.0396 | 0.0983 | 0.1353 | 0.0225 | 0.0000 | 0.2300 | 0.0000 | 0.0000 | 0.0000 |
| MB-0891 | 0.0219 | 0.0000 | 0.0277 | 0.0166 | 0.0000 | 0.1906 | 0.0000 | 0.0564 | 0.0000 | 0.1108 | 0.0000 | 0.0232 | 0.0770 | 0.0504 | 0.1391 | 0.0742 | 0.0211 | 0.0000 | 0.1889 | 0.0019 | 0.0000 | 0.0000 |
| MB-0658 | 0.0439 | 0.0000 | 0.0000 | 0.1198 | 0.0000 | 0.0006 | 0.0090 | 0.0522 | 0.0025 | 0.0982 | 0.0000 | 0.0604 | 0.0289 | 0.1918 | 0.1980 | 0.1093 | 0.0113 | 0.0000 | 0.0740 | 0.0000 | 0.0000 | 0.0000 |
| MB-0899 | 0.0540 | 0.0000 | 0.0385 | 0.0925 | 0.0000 | 0.0977 | 0.0000 | 0.0665 | 0.0092 | 0.0404 | 0.0000 | 0.0863 | 0.0193 | 0.0938 | 0.0738 | 0.2032 | 0.0159 | 0.0000 | 0.0779 | 0.0312 | 0.0000 | 0.0000 |
| MB-0605 | 0.0081 | 0.0000 | 0.0571 | 0.0890 | 0.0000 | 0.0555 | 0.0000 | 0.0250 | 0.0000 | 0.0057 | 0.0000 | 0.0644 | 0.1229 | 0.0000 | 0.0281 | 0.2791 | 0.0023 | 0.0000 | 0.1740 | 0.0886 | 0.0000 | 0.0002 |
| MB-0258 | 0.0110 | 0.0000 | 0.0186 | 0.1697 | 0.0619 | 0.0724 | 0.0000 | 0.0186 | 0.0000 | 0.0937 | 0.0000 | 0.0896 | 0.0840 | 0.0095 | 0.1424 | 0.1479 | 0.0133 | 0.0000 | 0.0673 | 0.0000 | 0.0000 | 0.0000 |
| MB-0506 | 0.0045 | 0.0144 | 0.0517 | 0.0000 | 0.0000 | 0.0590 | 0.0000 | 0.0325 | 0.0316 | 0.0482 | 0.0000 | 0.0559 | 0.0000 | 0.2532 | 0.0982 | 0.1553 | 0.0207 | 0.0000 | 0.1749 | 0.0000 | 0.0000 | 0.0000 |
| MB-0420 | 0.0224 | 0.0000 | 0.0000 | 0.0190 | 0.0000 | 0.0925 | 0.0000 | 0.0091 | 0.0096 | 0.0116 | 0.0000 | 0.0224 | 0.0385 | 0.2575 | 0.0657 | 0.4233 | 0.0000 | 0.0000 | 0.0000 | 0.0283 | 0.0000 | 0.0000 |
| MB-0223 | 0.0000 | 0.0139 | 0.2053 | 0.1354 | 0.0000 | 0.0060 | 0.0000 | 0.0698 | 0.0000 | 0.0545 | 0.0000 | 0.0400 | 0.0000 | 0.1930 | 0.0389 | 0.2091 | 0.0000 | 0.0000 | 0.0341 | 0.0000 | 0.0000 | 0.0000 |
| MB-0445 | 0.0390 | 0.0000 | 0.0309 | 0.0357 | 0.0000 | 0.0314 | 0.0000 | 0.0573 | 0.0401 | 0.0000 | 0.0000 | 0.0810 | 0.0640 | 0.0625 | 0.0529 | 0.1795 | 0.0710 | 0.0027 | 0.2519 | 0.0000 | 0.0000 | 0.0000 |
| MB-0199 | 0.0000 | 0.0576 | 0.0308 | 0.0177 | 0.0000 | 0.1365 | 0.0000 | 0.0414 | 0.0000 | 0.1518 | 0.0000 | 0.0288 | 0.0347 | 0.1126 | 0.0574 | 0.1253 | 0.0257 | 0.0000 | 0.1672 | 0.0125 | 0.0000 | 0.0000 |
| MB-0517 | 0.0245 | 0.0000 | 0.0518 | 0.0570 | 0.0000 | 0.0659 | 0.0000 | 0.0393 | 0.0000 | 0.0085 | 0.0000 | 0.0553 | 0.0488 | 0.0000 | 0.0111 | 0.0905 | 0.0000 | 0.0000 | 0.5472 | 0.0000 | 0.0000 | 0.0000 |
| MB-0155 | 0.0182 | 0.0000 | 0.0631 | 0.0302 | 0.0000 | 0.0371 | 0.0000 | 0.0633 | 0.0133 | 0.1295 | 0.0000 | 0.0777 | 0.0656 | 0.1078 | 0.0739 | 0.1427 | 0.0145 | 0.0000 | 0.1630 | 0.0000 | 0.0000 | 0.0000 |
| MB-0428 | 0.0555 | 0.0000 | 0.0000 | 0.1084 | 0.0000 | 0.0708 | 0.0354 | 0.0425 | 0.0132 | 0.0620 | 0.0000 | 0.0713 | 0.0216 | 0.1152 | 0.1548 | 0.1457 | 0.0000 | 0.0000 | 0.1035 | 0.0000 | 0.0000 | 0.0000 |
| MB-0117 | 0.0069 | 0.0000 | 0.1752 | 0.0434 | 0.0108 | 0.0000 | 0.0000 | 0.0349 | 0.0000 | 0.1301 | 0.0000 | 0.0882 | 0.0012 | 0.0459 | 0.0868 | 0.0524 | 0.0157 | 0.0000 | 0.3086 | 0.0000 | 0.0000 | 0.0000 |
| MB-0906 | 0.0000 | 0.0000 | 0.4138 | 0.0277 | 0.0000 | 0.0029 | 0.0078 | 0.0343 | 0.0000 | 0.0569 | 0.0000 | 0.0913 | 0.0195 | 0.1259 | 0.0455 | 0.1123 | 0.0037 | 0.0127 | 0.0458 | 0.0000 | 0.0000 | 0.0000 |
| MB-0249 | 0.0193 | 0.0017 | 0.0000 | 0.0000 | 0.0155 | 0.0000 | 0.0000 | 0.0534 | 0.0000 | 0.0926 | 0.0000 | 0.0249 | 0.0000 | 0.4169 | 0.1039 | 0.2042 | 0.0120 | 0.0000 | 0.0419 | 0.0041 | 0.0000 | 0.0095 |
| MB-0660 | 0.0299 | 0.0000 | 0.0000 | 0.0871 | 0.0000 | 0.0329 | 0.0000 | 0.0643 | 0.0000 | 0.0228 | 0.0272 | 0.0000 | 0.0347 | 0.0212 | 0.1147 | 0.2845 | 0.0378 | 0.0000 | 0.1550 | 0.0822 | 0.0000 | 0.0057 |
| MB-0497 | 0.0180 | 0.0000 | 0.0000 | 0.0465 | 0.0000 | 0.1432 | 0.0000 | 0.0443 | 0.0170 | 0.1498 | 0.0000 | 0.0570 | 0.0000 | 0.1143 | 0.1272 | 0.0827 | 0.0000 | 0.0000 | 0.1849 | 0.0151 | 0.0000 | 0.0000 |
| MB-0434 | 0.1003 | 0.0000 | 0.0963 | 0.0104 | 0.0000 | 0.0797 | 0.0000 | 0.0529 | 0.0062 | 0.0000 | 0.0441 | 0.0164 | 0.0245 | 0.2287 | 0.0992 | 0.1189 | 0.0104 | 0.0000 | 0.0812 | 0.0311 | 0.0000 | 0.0000 |
| MB-0143 | 0.0000 | 0.0317 | 0.0000 | 0.1209 | 0.0000 | 0.0607 | 0.0000 | 0.1241 | 0.0000 | 0.0000 | 0.0000 | 0.0603 | 0.0069 | 0.0406 | 0.0861 | 0.2609 | 0.0211 | 0.0000 | 0.1868 | 0.0000 | 0.0000 | 0.0000 |
| MB-0513 | 0.0000 | 0.0000 | 0.0044 | 0.0060 | 0.0000 | 0.0495 | 0.0000 | 0.0453 | 0.0370 | 0.0280 | 0.0000 | 0.0867 | 0.0395 | 0.0338 | 0.0717 | 0.4195 | 0.0311 | 0.0000 | 0.0000 | 0.1476 | 0.0000 | 0.0000 |
| MB-0541 | 0.0000 | 0.0000 | 0.1870 | 0.0000 | 0.0000 | 0.0819 | 0.0000 | 0.0356 | 0.0173 | 0.0647 | 0.0000 | 0.0657 | 0.0176 | 0.1565 | 0.1208 | 0.1309 | 0.0100 | 0.0000 | 0.1120 | 0.0000 | 0.0000 | 0.0000 |
| MB-0653 | 0.0000 | 0.0000 | 0.0050 | 0.0029 | 0.0000 | 0.0318 | 0.0324 | 0.1006 | 0.0000 | 0.1015 | 0.0000 | 0.0555 | 0.0793 | 0.2338 | 0.1046 | 0.1916 | 0.0000 | 0.0000 | 0.0602 | 0.0000 | 0.0000 | 0.0006 |
| MB-0455 | 0.0485 | 0.0053 | 0.0148 | 0.0512 | 0.0000 | 0.1427 | 0.0089 | 0.1012 | 0.0000 | 0.0725 | 0.0000 | 0.0724 | 0.0000 | 0.1289 | 0.1084 | 0.1156 | 0.0459 | 0.0000 | 0.0838 | 0.0000 | 0.0000 | 0.0000 |
| MB-0540 | 0.0007 | 0.0993 | 0.0000 | 0.0951 | 0.1815 | 0.2004 | 0.0930 | 0.0058 | 0.0458 | 0.0106 | 0.0079 | 0.0000 | 0.0217 | 0.0863 | 0.0461 | 0.0818 | 0.0000 | 0.0000 | 0.0242 | 0.0000 | 0.0000 | 0.0000 |
| MB-0384 | 0.0558 | 0.0000 | 0.0085 | 0.0454 | 0.0000 | 0.1383 | 0.0000 | 0.0269 | 0.0224 | 0.0000 | 0.0000 | 0.0174 | 0.0000 | 0.1766 | 0.1063 | 0.2987 | 0.0000 | 0.0000 | 0.1037 | 0.0000 | 0.0000 | 0.0000 |
| MB-0637 | 0.0167 | 0.0000 | 0.0672 | 0.0000 | 0.0203 | 0.0742 | 0.0000 | 0.0877 | 0.0000 | 0.1101 | 0.0000 | 0.0904 | 0.0000 | 0.1560 | 0.1481 | 0.1555 | 0.0172 | 0.0000 | 0.0565 | 0.0000 | 0.0000 | 0.0000 |
| MB-0157 | 0.0000 | 0.0168 | 0.0543 | 0.1253 | 0.0000 | 0.0000 | 0.0619 | 0.0494 | 0.0000 | 0.0069 | 0.0000 | 0.1207 | 0.0059 | 0.1736 | 0.1924 | 0.1739 | 0.0000 | 0.0000 | 0.0189 | 0.0000 | 0.0000 | 0.0000 |
| MB-0443 | 0.0411 | 0.0000 | 0.0134 | 0.1746 | 0.0000 | 0.2578 | 0.0000 | 0.0145 | 0.0000 | 0.1052 | 0.0000 | 0.0487 | 0.0356 | 0.0636 | 0.0958 | 0.0963 | 0.0159 | 0.0000 | 0.0228 | 0.0148 | 0.0000 | 0.0000 |
| MB-0584 | 0.0035 | 0.0000 | 0.0000 | 0.0733 | 0.0000 | 0.0000 | 0.0000 | 0.0669 | 0.0000 | 0.0477 | 0.0000 | 0.0748 | 0.0326 | 0.1367 | 0.0516 | 0.2886 | 0.0125 | 0.0000 | 0.0000 | 0.2116 | 0.0000 | 0.0000 |
| MB-0292 | 0.0000 | 0.0118 | 0.0050 | 0.0139 | 0.0000 | 0.0636 | 0.0000 | 0.0390 | 0.0012 | 0.0122 | 0.0040 | 0.0000 | 0.0000 | 0.3623 | 0.0494 | 0.3277 | 0.0270 | 0.0000 | 0.0120 | 0.0709 | 0.0000 | 0.0000 |
| MB-0322 | 0.0000 | 0.0000 | 0.0238 | 0.0713 | 0.0000 | 0.0747 | 0.0000 | 0.0639 | 0.0287 | 0.0000 | 0.0000 | 0.0552 | 0.0405 | 0.0000 | 0.0531 | 0.3677 | 0.0284 | 0.0000 | 0.1865 | 0.0000 | 0.0000 | 0.0064 |
| MB-0501 | 0.0000 | 0.0258 | 0.0000 | 0.0272 | 0.0000 | 0.0000 | 0.0000 | 0.0059 | 0.0586 | 0.0393 | 0.0000 | 0.0508 | 0.0037 | 0.0000 | 0.0313 | 0.5884 | 0.0143 | 0.0000 | 0.0000 | 0.1351 | 0.0040 | 0.0156 |
| MB-0401 | 0.0012 | 0.0000 | 0.0047 | 0.0356 | 0.0000 | 0.0986 | 0.0000 | 0.0627 | 0.0000 | 0.0107 | 0.0000 | 0.0635 | 0.0318 | 0.2199 | 0.0852 | 0.3049 | 0.0025 | 0.0000 | 0.0788 | 0.0000 | 0.0000 | 0.0000 |
| MB-0140 | 0.0000 | 0.0013 | 0.0269 | 0.0351 | 0.0000 | 0.1136 | 0.0000 | 0.0606 | 0.0000 | 0.0000 | 0.0000 | 0.0374 | 0.0095 | 0.0369 | 0.0966 | 0.4365 | 0.0057 | 0.0000 | 0.0539 | 0.0861 | 0.0000 | 0.0000 |
| MB-0606 | 0.0032 | 0.0315 | 0.0000 | 0.0174 | 0.0744 | 0.0000 | 0.0000 | 0.0245 | 0.0376 | 0.0601 | 0.0000 | 0.0066 | 0.0000 | 0.2373 | 0.1546 | 0.2217 | 0.0000 | 0.0000 | 0.0000 | 0.1311 | 0.0000 | 0.0000 |
| MB-0666 | 0.0279 | 0.0000 | 0.0194 | 0.0481 | 0.0000 | 0.1174 | 0.0000 | 0.1106 | 0.0000 | 0.0000 | 0.0000 | 0.1169 | 0.0109 | 0.0864 | 0.1512 | 0.1576 | 0.0186 | 0.0000 | 0.1350 | 0.0000 | 0.0000 | 0.0000 |
| MB-0598 | 0.0142 | 0.0000 | 0.0032 | 0.0566 | 0.0000 | 0.0951 | 0.0000 | 0.0561 | 0.0129 | 0.0000 | 0.0000 | 0.0838 | 0.0425 | 0.0168 | 0.0676 | 0.2392 | 0.0078 | 0.0050 | 0.2975 | 0.0000 | 0.0000 | 0.0015 |
| MB-0453 | 0.0172 | 0.0000 | 0.0135 | 0.0249 | 0.0000 | 0.0027 | 0.0000 | 0.0906 | 0.0000 | 0.0266 | 0.0000 | 0.0667 | 0.0000 | 0.4841 | 0.0048 | 0.1843 | 0.0000 | 0.0096 | 0.0000 | 0.0749 | 0.0000 | 0.0000 |
| MB-0138 | 0.0127 | 0.0000 | 0.1519 | 0.0937 | 0.0000 | 0.1399 | 0.0000 | 0.0292 | 0.0269 | 0.0020 | 0.0000 | 0.0449 | 0.0138 | 0.0026 | 0.0982 | 0.1001 | 0.0177 | 0.0000 | 0.2555 | 0.0109 | 0.0000 | 0.0000 |
| MB-0579 | 0.0446 | 0.0000 | 0.0007 | 0.0094 | 0.0000 | 0.0000 | 0.0000 | 0.0464 | 0.0258 | 0.0185 | 0.0000 | 0.0194 | 0.0000 | 0.3915 | 0.0138 | 0.3030 | 0.0000 | 0.0000 | 0.1173 | 0.0060 | 0.0000 | 0.0036 |
| MB-0471 | 0.0059 | 0.0000 | 0.0082 | 0.0000 | 0.1029 | 0.0000 | 0.0000 | 0.0324 | 0.0038 | 0.0670 | 0.0000 | 0.0110 | 0.0343 | 0.3109 | 0.0229 | 0.2242 | 0.0196 | 0.0000 | 0.0438 | 0.1132 | 0.0000 | 0.0000 |
| MB-0619 | 0.1077 | 0.0000 | 0.0000 | 0.0787 | 0.0795 | 0.0671 | 0.0293 | 0.0588 | 0.0000 | 0.1160 | 0.0000 | 0.0515 | 0.0415 | 0.0492 | 0.1650 | 0.0683 | 0.0086 | 0.0000 | 0.0789 | 0.0000 | 0.0000 | 0.0000 |
| MB-0171 | 0.0148 | 0.0000 | 0.0561 | 0.0721 | 0.0000 | 0.1154 | 0.0000 | 0.0435 | 0.0000 | 0.0659 | 0.0000 | 0.0932 | 0.0227 | 0.0000 | 0.0667 | 0.1428 | 0.0357 | 0.0000 | 0.2277 | 0.0433 | 0.0000 | 0.0000 |
| MB-0310 | 0.0008 | 0.0378 | 0.0204 | 0.0342 | 0.0000 | 0.1680 | 0.0000 | 0.1126 | 0.0000 | 0.1054 | 0.0000 | 0.0849 | 0.0072 | 0.0466 | 0.0985 | 0.1488 | 0.0128 | 0.0000 | 0.0000 | 0.1221 | 0.0000 | 0.0000 |
| MB-0621 | 0.0177 | 0.0000 | 0.0420 | 0.1170 | 0.0000 | 0.0055 | 0.0000 | 0.0411 | 0.0000 | 0.0164 | 0.0000 | 0.0614 | 0.0040 | 0.0538 | 0.0367 | 0.2270 | 0.0112 | 0.0000 | 0.3664 | 0.0000 | 0.0000 | 0.0000 |
| MB-0614 | 0.0467 | 0.0000 | 0.0000 | 0.1506 | 0.0000 | 0.1078 | 0.0000 | 0.0592 | 0.0000 | 0.0605 | 0.0000 | 0.1320 | 0.0422 | 0.0570 | 0.1555 | 0.0705 | 0.0279 | 0.0000 | 0.0901 | 0.0000 | 0.0000 | 0.0000 |
| MB-0372 | 0.0000 | 0.0000 | 0.0000 | 0.1461 | 0.0000 | 0.0134 | 0.0000 | 0.0936 | 0.0362 | 0.0479 | 0.0000 | 0.1520 | 0.0000 | 0.0155 | 0.0466 | 0.2640 | 0.1007 | 0.0283 | 0.0513 | 0.0000 | 0.0000 | 0.0044 |
| MB-0374 | 0.0000 | 0.0042 | 0.0000 | 0.0111 | 0.0000 | 0.0077 | 0.0000 | 0.0749 | 0.0322 | 0.0488 | 0.0000 | 0.0322 | 0.0000 | 0.3271 | 0.0721 | 0.1528 | 0.0000 | 0.0000 | 0.1730 | 0.0640 | 0.0000 | 0.0000 |
| MB-0382 | 0.0122 | 0.0010 | 0.0000 | 0.0901 | 0.0000 | 0.0196 | 0.0000 | 0.0894 | 0.0000 | 0.0838 | 0.0000 | 0.0370 | 0.0105 | 0.0000 | 0.1663 | 0.1132 | 0.0300 | 0.0000 | 0.3467 | 0.0000 | 0.0000 | 0.0000 |
| MB-0066 | 0.0132 | 0.0000 | 0.0682 | 0.0640 | 0.0000 | 0.0442 | 0.0000 | 0.0589 | 0.0000 | 0.1721 | 0.0000 | 0.0893 | 0.0083 | 0.0944 | 0.1382 | 0.0942 | 0.0182 | 0.0000 | 0.1368 | 0.0000 | 0.0000 | 0.0000 |
| MB-0144 | 0.0000 | 0.0416 | 0.0000 | 0.0704 | 0.0000 | 0.0680 | 0.0000 | 0.0388 | 0.0000 | 0.1810 | 0.0000 | 0.0089 | 0.0225 | 0.1415 | 0.0986 | 0.1420 | 0.0180 | 0.0000 | 0.1609 | 0.0079 | 0.0000 | 0.0000 |
| MB-0596 | 0.0012 | 0.0277 | 0.1561 | 0.0842 | 0.0000 | 0.0366 | 0.0000 | 0.0387 | 0.0017 | 0.0640 | 0.0000 | 0.1052 | 0.0181 | 0.0498 | 0.0964 | 0.1767 | 0.0136 | 0.0000 | 0.1301 | 0.0000 | 0.0000 | 0.0000 |
| MB-0164 | 0.0074 | 0.0000 | 0.0062 | 0.0040 | 0.0085 | 0.0192 | 0.0000 | 0.0285 | 0.0047 | 0.0260 | 0.0000 | 0.0181 | 0.0684 | 0.4258 | 0.0191 | 0.2380 | 0.0000 | 0.0087 | 0.0000 | 0.1175 | 0.0000 | 0.0000 |
| MB-0215 | 0.0000 | 0.0044 | 0.0490 | 0.0486 | 0.0000 | 0.0747 | 0.0000 | 0.0527 | 0.0000 | 0.0066 | 0.0000 | 0.0464 | 0.0144 | 0.0717 | 0.1454 | 0.1538 | 0.0015 | 0.0000 | 0.3307 | 0.0000 | 0.0000 | 0.0000 |
| MB-0146 | 0.0000 | 0.0187 | 0.0017 | 0.0236 | 0.0000 | 0.1322 | 0.0000 | 0.0448 | 0.0070 | 0.0346 | 0.0000 | 0.0465 | 0.0031 | 0.0233 | 0.0754 | 0.3632 | 0.0243 | 0.0000 | 0.1585 | 0.0289 | 0.0000 | 0.0142 |
| MB-0229 | 0.0110 | 0.0000 | 0.0200 | 0.0000 | 0.0000 | 0.0359 | 0.0000 | 0.0916 | 0.0000 | 0.0284 | 0.0000 | 0.0092 | 0.0000 | 0.1870 | 0.0320 | 0.2091 | 0.0000 | 0.0000 | 0.3073 | 0.0684 | 0.0000 | 0.0000 |
| MB-0505 | 0.0000 | 0.0000 | 0.0191 | 0.0703 | 0.0000 | 0.1080 | 0.0000 | 0.0183 | 0.0000 | 0.0467 | 0.0000 | 0.0652 | 0.0354 | 0.0000 | 0.0380 | 0.1512 | 0.0000 | 0.0000 | 0.4438 | 0.0038 | 0.0000 | 0.0000 |
| MB-0102 | 0.0279 | 0.0000 | 0.1424 | 0.0277 | 0.0262 | 0.1104 | 0.0111 | 0.0480 | 0.0000 | 0.0201 | 0.0127 | 0.0647 | 0.0242 | 0.0275 | 0.1741 | 0.0923 | 0.0117 | 0.0000 | 0.1791 | 0.0000 | 0.0000 | 0.0000 |
| MB-0569 | 0.0000 | 0.0243 | 0.0872 | 0.0177 | 0.0000 | 0.1367 | 0.0000 | 0.0299 | 0.0162 | 0.0527 | 0.0000 | 0.0642 | 0.0051 | 0.0443 | 0.0579 | 0.2262 | 0.0055 | 0.0000 | 0.1895 | 0.0425 | 0.0000 | 0.0000 |
| MB-0516 | 0.0000 | 0.0000 | 0.0030 | 0.0282 | 0.0000 | 0.0547 | 0.0093 | 0.0597 | 0.0034 | 0.0000 | 0.0000 | 0.0697 | 0.0851 | 0.1130 | 0.1138 | 0.2827 | 0.0115 | 0.0910 | 0.0000 | 0.0672 | 0.0000 | 0.0077 |
| MB-0272 | 0.0237 | 0.0000 | 0.0606 | 0.0441 | 0.0000 | 0.0324 | 0.0000 | 0.0625 | 0.0319 | 0.0806 | 0.0000 | 0.0217 | 0.0000 | 0.2289 | 0.0768 | 0.3100 | 0.0080 | 0.0000 | 0.0189 | 0.0000 | 0.0000 | 0.0000 |
| MB-0585 | 0.0000 | 0.0224 | 0.0000 | 0.0340 | 0.0000 | 0.0696 | 0.0000 | 0.0394 | 0.0051 | 0.0000 | 0.0000 | 0.0756 | 0.0324 | 0.0193 | 0.0577 | 0.2030 | 0.0100 | 0.0000 | 0.4316 | 0.0000 | 0.0000 | 0.0000 |
| MB-0494 | 0.0000 | 0.0000 | 0.0568 | 0.0679 | 0.0000 | 0.0282 | 0.0219 | 0.0071 | 0.0000 | 0.0000 | 0.0000 | 0.0417 | 0.0370 | 0.3107 | 0.1168 | 0.2966 | 0.0000 | 0.0000 | 0.0150 | 0.0004 | 0.0000 | 0.0000 |
| MB-0306 | 0.0184 | 0.0000 | 0.0281 | 0.0673 | 0.0000 | 0.0356 | 0.0056 | 0.0882 | 0.0000 | 0.0615 | 0.0000 | 0.0960 | 0.0531 | 0.0570 | 0.0978 | 0.0670 | 0.0000 | 0.0000 | 0.3243 | 0.0000 | 0.0000 | 0.0000 |
| MB-0463 | 0.0002 | 0.0005 | 0.0324 | 0.0243 | 0.0000 | 0.0354 | 0.0000 | 0.0440 | 0.0246 | 0.0000 | 0.0000 | 0.0147 | 0.0000 | 0.1419 | 0.0349 | 0.3092 | 0.0128 | 0.0000 | 0.3243 | 0.0000 | 0.0000 | 0.0007 |
| MB-0198 | 0.0233 | 0.0000 | 0.0124 | 0.0000 | 0.0000 | 0.1282 | 0.0000 | 0.0510 | 0.0000 | 0.0076 | 0.0000 | 0.0547 | 0.0053 | 0.0000 | 0.0470 | 0.3433 | 0.0178 | 0.0000 | 0.1607 | 0.1436 | 0.0000 | 0.0050 |
| MB-0203 | 0.0000 | 0.0282 | 0.0000 | 0.0000 | 0.0043 | 0.1275 | 0.0000 | 0.0273 | 0.0000 | 0.1147 | 0.0000 | 0.0731 | 0.0389 | 0.0570 | 0.1556 | 0.2060 | 0.0499 | 0.0000 | 0.1176 | 0.0000 | 0.0000 | 0.0000 |
| MB-0607 | 0.0237 | 0.0000 | 0.0248 | 0.2457 | 0.0000 | 0.0774 | 0.0000 | 0.0388 | 0.0000 | 0.0000 | 0.0000 | 0.0868 | 0.0642 | 0.0227 | 0.1239 | 0.1137 | 0.0215 | 0.0000 | 0.1568 | 0.0000 | 0.0000 | 0.0000 |
| MB-0631 | 0.0000 | 0.0000 | 0.0099 | 0.0842 | 0.0000 | 0.1261 | 0.0000 | 0.0293 | 0.0000 | 0.0407 | 0.0000 | 0.0896 | 0.0354 | 0.0000 | 0.1191 | 0.1721 | 0.0101 | 0.0000 | 0.2650 | 0.0185 | 0.0000 | 0.0000 |
| MB-0363 | 0.0351 | 0.0000 | 0.0084 | 0.1014 | 0.0000 | 0.1368 | 0.0111 | 0.0978 | 0.0000 | 0.1530 | 0.0000 | 0.0521 | 0.0201 | 0.1119 | 0.1117 | 0.0985 | 0.0000 | 0.0000 | 0.0622 | 0.0000 | 0.0000 | 0.0000 |
| MB-0427 | 0.0462 | 0.0000 | 0.0000 | 0.0974 | 0.0788 | 0.1535 | 0.0000 | 0.0688 | 0.0123 | 0.1135 | 0.0000 | 0.0576 | 0.0042 | 0.0898 | 0.0943 | 0.1059 | 0.0000 | 0.0000 | 0.0354 | 0.0422 | 0.0000 | 0.0000 |
| MB-0519 | 0.1130 | 0.0000 | 0.0029 | 0.0838 | 0.2099 | 0.1532 | 0.0673 | 0.0011 | 0.0000 | 0.0028 | 0.0376 | 0.0000 | 0.0749 | 0.0388 | 0.1169 | 0.0365 | 0.0008 | 0.0000 | 0.0603 | 0.0000 | 0.0000 | 0.0000 |
| MB-0371 | 0.0011 | 0.0000 | 0.0000 | 0.0050 | 0.0000 | 0.0350 | 0.0000 | 0.0450 | 0.0221 | 0.1074 | 0.0000 | 0.0606 | 0.0000 | 0.2443 | 0.0729 | 0.2549 | 0.0035 | 0.0000 | 0.1111 | 0.0371 | 0.0000 | 0.0000 |
| MB-0380 | 0.0238 | 0.0000 | 0.0713 | 0.0741 | 0.0000 | 0.0056 | 0.0000 | 0.0674 | 0.0000 | 0.0577 | 0.0000 | 0.0434 | 0.0000 | 0.2689 | 0.1482 | 0.2011 | 0.0000 | 0.0000 | 0.0385 | 0.0000 | 0.0000 | 0.0000 |
| MB-0221 | 0.0000 | 0.0210 | 0.1994 | 0.0248 | 0.0000 | 0.1100 | 0.0006 | 0.0609 | 0.0031 | 0.0869 | 0.0000 | 0.0632 | 0.0176 | 0.0387 | 0.0917 | 0.1802 | 0.0267 | 0.0000 | 0.0752 | 0.0000 | 0.0000 | 0.0000 |
| MB-0348 | 0.0156 | 0.0143 | 0.0000 | 0.0000 | 0.0360 | 0.0000 | 0.0000 | 0.0597 | 0.0508 | 0.0000 | 0.0664 | 0.0000 | 0.0166 | 0.3595 | 0.0244 | 0.3268 | 0.0000 | 0.0000 | 0.0000 | 0.0197 | 0.0100 | 0.0000 |
| MB-0261 | 0.0000 | 0.0212 | 0.0000 | 0.0265 | 0.0000 | 0.0616 | 0.0000 | 0.0503 | 0.0000 | 0.0187 | 0.0000 | 0.0452 | 0.0135 | 0.0000 | 0.0579 | 0.3257 | 0.0009 | 0.0000 | 0.3785 | 0.0000 | 0.0000 | 0.0000 |
| MB-0576 | 0.0000 | 0.0019 | 0.0020 | 0.0931 | 0.0000 | 0.1195 | 0.0000 | 0.0126 | 0.0000 | 0.0000 | 0.0000 | 0.0517 | 0.0333 | 0.0000 | 0.0222 | 0.3500 | 0.0066 | 0.0000 | 0.3071 | 0.0000 | 0.0000 | 0.0000 |
| MB-0385 | 0.0083 | 0.0000 | 0.0393 | 0.0862 | 0.0000 | 0.0402 | 0.0000 | 0.0518 | 0.0036 | 0.0495 | 0.0000 | 0.0808 | 0.0000 | 0.1180 | 0.0556 | 0.3421 | 0.0000 | 0.0000 | 0.1247 | 0.0000 | 0.0000 | 0.0000 |
| MB-0659 | 0.0000 | 0.0264 | 0.0000 | 0.0789 | 0.0333 | 0.0000 | 0.0000 | 0.0350 | 0.0907 | 0.0000 | 0.0000 | 0.0374 | 0.0201 | 0.4319 | 0.0000 | 0.2275 | 0.0000 | 0.0000 | 0.0000 | 0.0179 | 0.0010 | 0.0000 |
| MB-0270 | 0.0034 | 0.0000 | 0.0052 | 0.0282 | 0.0000 | 0.1094 | 0.0000 | 0.0439 | 0.0000 | 0.0793 | 0.0000 | 0.0755 | 0.0226 | 0.0000 | 0.1063 | 0.1640 | 0.0528 | 0.0000 | 0.3093 | 0.0000 | 0.0000 | 0.0000 |
| MB-0379 | 0.0000 | 0.0000 | 0.0098 | 0.0629 | 0.0000 | 0.2758 | 0.0000 | 0.0144 | 0.0000 | 0.0321 | 0.0000 | 0.0304 | 0.0000 | 0.1059 | 0.0775 | 0.1877 | 0.0035 | 0.0000 | 0.2000 | 0.0000 | 0.0000 | 0.0000 |
| MB-0527 | 0.0000 | 0.0570 | 0.0367 | 0.0304 | 0.0000 | 0.1123 | 0.0000 | 0.1211 | 0.0000 | 0.0629 | 0.0000 | 0.0722 | 0.0000 | 0.1558 | 0.0764 | 0.1554 | 0.0000 | 0.0000 | 0.0053 | 0.1146 | 0.0000 | 0.0000 |
| MB-0624 | 0.0000 | 0.0476 | 0.1287 | 0.0934 | 0.0000 | 0.0310 | 0.0000 | 0.0599 | 0.0000 | 0.0689 | 0.0000 | 0.0974 | 0.0457 | 0.0000 | 0.0294 | 0.1053 | 0.0229 | 0.0000 | 0.0000 | 0.2698 | 0.0000 | 0.0000 |
| MB-0273 | 0.0000 | 0.0665 | 0.0000 | 0.0000 | 0.0000 | 0.2813 | 0.0000 | 0.0083 | 0.0000 | 0.0547 | 0.0516 | 0.0000 | 0.0780 | 0.0000 | 0.0086 | 0.3210 | 0.0164 | 0.0000 | 0.0574 | 0.0210 | 0.0352 | 0.0000 |
| MB-0050 | 0.0517 | 0.0000 | 0.0541 | 0.0643 | 0.0000 | 0.1586 | 0.0000 | 0.1257 | 0.0000 | 0.1266 | 0.0000 | 0.0704 | 0.0257 | 0.0000 | 0.0626 | 0.1132 | 0.0559 | 0.0000 | 0.0912 | 0.0000 | 0.0000 | 0.0000 |
| MB-0460 | 0.0000 | 0.0060 | 0.0030 | 0.0745 | 0.0254 | 0.1338 | 0.0000 | 0.0265 | 0.0071 | 0.0000 | 0.0000 | 0.0555 | 0.0492 | 0.0835 | 0.0837 | 0.2163 | 0.0007 | 0.0000 | 0.2349 | 0.0000 | 0.0000 | 0.0000 |
| MB-0654 | 0.0049 | 0.0000 | 0.0005 | 0.1083 | 0.0000 | 0.0000 | 0.0000 | 0.0555 | 0.0455 | 0.0340 | 0.0000 | 0.0324 | 0.0107 | 0.1197 | 0.0755 | 0.2660 | 0.0056 | 0.0000 | 0.2411 | 0.0000 | 0.0000 | 0.0000 |
| MB-0454 | 0.0214 | 0.0000 | 0.0074 | 0.0000 | 0.0000 | 0.0786 | 0.0000 | 0.0451 | 0.0000 | 0.0023 | 0.0000 | 0.0428 | 0.0048 | 0.0000 | 0.0094 | 0.5017 | 0.0521 | 0.0000 | 0.0000 | 0.2271 | 0.0030 | 0.0044 |
| MB-0392 | 0.0056 | 0.0000 | 0.0055 | 0.0357 | 0.0000 | 0.1686 | 0.0000 | 0.0485 | 0.0000 | 0.0099 | 0.0000 | 0.0507 | 0.1626 | 0.0116 | 0.1159 | 0.1546 | 0.0373 | 0.0000 | 0.1935 | 0.0000 | 0.0000 | 0.0000 |
| MB-0336 | 0.0243 | 0.0000 | 0.0030 | 0.0738 | 0.0000 | 0.0942 | 0.0000 | 0.0145 | 0.0149 | 0.0194 | 0.0000 | 0.0846 | 0.0290 | 0.1118 | 0.0753 | 0.2771 | 0.0533 | 0.0000 | 0.0644 | 0.0572 | 0.0000 | 0.0030 |
| MB-0467 | 0.0459 | 0.0000 | 0.1160 | 0.0231 | 0.0000 | 0.0891 | 0.0000 | 0.0322 | 0.0009 | 0.0000 | 0.0000 | 0.0109 | 0.0000 | 0.3438 | 0.0472 | 0.1369 | 0.0000 | 0.0000 | 0.1149 | 0.0390 | 0.0000 | 0.0000 |
| MB-0349 | 0.0052 | 0.0201 | 0.0000 | 0.0303 | 0.0000 | 0.1288 | 0.0000 | 0.0136 | 0.0000 | 0.0798 | 0.0000 | 0.0000 | 0.0143 | 0.0014 | 0.1736 | 0.3344 | 0.0182 | 0.0000 | 0.0837 | 0.0965 | 0.0000 | 0.0000 |
| MB-0378 | 0.0000 | 0.0007 | 0.0550 | 0.0257 | 0.0000 | 0.0178 | 0.1012 | 0.0594 | 0.0000 | 0.0000 | 0.0000 | 0.0419 | 0.0161 | 0.2134 | 0.2066 | 0.2170 | 0.0000 | 0.0000 | 0.0397 | 0.0017 | 0.0000 | 0.0040 |
| MB-0176 | 0.0654 | 0.0000 | 0.0503 | 0.0322 | 0.0000 | 0.1879 | 0.0000 | 0.0537 | 0.0000 | 0.0519 | 0.0000 | 0.0137 | 0.0193 | 0.0000 | 0.0332 | 0.1698 | 0.0183 | 0.0000 | 0.2178 | 0.0866 | 0.0000 | 0.0000 |
| MB-0429 | 0.0049 | 0.0013 | 0.0000 | 0.0535 | 0.0000 | 0.0532 | 0.0000 | 0.0203 | 0.0001 | 0.0658 | 0.0000 | 0.0349 | 0.0624 | 0.0000 | 0.0466 | 0.3425 | 0.0931 | 0.0000 | 0.0571 | 0.1639 | 0.0000 | 0.0003 |
| MB-0397 | 0.0073 | 0.0000 | 0.0577 | 0.0146 | 0.1082 | 0.0579 | 0.0000 | 0.0000 | 0.0000 | 0.0311 | 0.0000 | 0.0060 | 0.2911 | 0.0530 | 0.0000 | 0.0000 | 0.0119 | 0.0260 | 0.2881 | 0.0027 | 0.0000 | 0.0444 |
| MB-0571 | 0.0000 | 0.0000 | 0.0019 | 0.0021 | 0.0033 | 0.0363 | 0.0000 | 0.0294 | 0.0025 | 0.0118 | 0.0000 | 0.0110 | 0.0392 | 0.1447 | 0.0991 | 0.1795 | 0.0358 | 0.0000 | 0.4034 | 0.0000 | 0.0000 | 0.0000 |
| MB-0426 | 0.0546 | 0.0000 | 0.0412 | 0.0022 | 0.0000 | 0.2118 | 0.0000 | 0.0329 | 0.0000 | 0.0019 | 0.0000 | 0.0285 | 0.0448 | 0.0497 | 0.0283 | 0.2956 | 0.0193 | 0.0000 | 0.1892 | 0.0000 | 0.0000 | 0.0000 |
| MB-0135 | 0.0000 | 0.0806 | 0.0000 | 0.0000 | 0.0000 | 0.0835 | 0.0000 | 0.0012 | 0.0000 | 0.1525 | 0.0000 | 0.0000 | 0.0000 | 0.1569 | 0.0297 | 0.1963 | 0.0166 | 0.0000 | 0.2667 | 0.0160 | 0.0000 | 0.0000 |
| MB-0112 | 0.0295 | 0.0000 | 0.0082 | 0.0313 | 0.0000 | 0.2778 | 0.0000 | 0.0570 | 0.0044 | 0.0713 | 0.0000 | 0.0658 | 0.0116 | 0.0000 | 0.1023 | 0.1480 | 0.0194 | 0.0000 | 0.1734 | 0.0000 | 0.0000 | 0.0000 |
| MB-0352 | 0.0000 | 0.0000 | 0.0316 | 0.0354 | 0.0000 | 0.0851 | 0.0000 | 0.0666 | 0.0000 | 0.0994 | 0.0000 | 0.1010 | 0.0000 | 0.2440 | 0.0818 | 0.1814 | 0.0015 | 0.0000 | 0.0722 | 0.0000 | 0.0000 | 0.0000 |
| MB-0644 | 0.0000 | 0.0145 | 0.0152 | 0.1456 | 0.0000 | 0.1494 | 0.0000 | 0.0534 | 0.0000 | 0.0000 | 0.0000 | 0.1155 | 0.0361 | 0.0144 | 0.0837 | 0.1982 | 0.0155 | 0.0000 | 0.0942 | 0.0642 | 0.0000 | 0.0000 |
| MB-0601 | 0.0000 | 0.0000 | 0.0000 | 0.0173 | 0.0000 | 0.0000 | 0.0000 | 0.0248 | 0.0242 | 0.0118 | 0.0000 | 0.0166 | 0.0000 | 0.4827 | 0.0205 | 0.3225 | 0.0000 | 0.0000 | 0.0511 | 0.0144 | 0.0000 | 0.0141 |
| MB-0568 | 0.0000 | 0.0404 | 0.0000 | 0.0758 | 0.0000 | 0.0303 | 0.0000 | 0.0704 | 0.0000 | 0.1158 | 0.0000 | 0.0086 | 0.0260 | 0.0000 | 0.1043 | 0.1464 | 0.0109 | 0.0000 | 0.3118 | 0.0593 | 0.0000 | 0.0000 |
| MB-0328 | 0.0000 | 0.0120 | 0.0000 | 0.0579 | 0.0000 | 0.1354 | 0.0000 | 0.0540 | 0.0156 | 0.0110 | 0.0000 | 0.0668 | 0.0000 | 0.2458 | 0.1130 | 0.1668 | 0.0082 | 0.0000 | 0.0873 | 0.0261 | 0.0000 | 0.0000 |
| MB-0325 | 0.0092 | 0.0000 | 0.1056 | 0.0008 | 0.0000 | 0.0107 | 0.0102 | 0.0281 | 0.0000 | 0.0798 | 0.0000 | 0.0531 | 0.0000 | 0.3547 | 0.0949 | 0.1995 | 0.0000 | 0.0000 | 0.0000 | 0.0535 | 0.0000 | 0.0000 |
| MB-0358 | 0.0097 | 0.0000 | 0.0271 | 0.0715 | 0.0000 | 0.0956 | 0.0000 | 0.0460 | 0.0000 | 0.0387 | 0.0000 | 0.0587 | 0.0283 | 0.1880 | 0.1526 | 0.1479 | 0.0101 | 0.0000 | 0.1258 | 0.0000 | 0.0000 | 0.0000 |
| MB-0413 | 0.0056 | 0.0000 | 0.0091 | 0.0193 | 0.0000 | 0.1010 | 0.0000 | 0.0851 | 0.0124 | 0.0313 | 0.0000 | 0.0600 | 0.0106 | 0.1235 | 0.1185 | 0.1403 | 0.0000 | 0.0000 | 0.2833 | 0.0000 | 0.0000 | 0.0000 |
| MB-0636 | 0.0168 | 0.0000 | 0.0000 | 0.0313 | 0.0000 | 0.0123 | 0.0000 | 0.0511 | 0.0000 | 0.0486 | 0.0000 | 0.0437 | 0.0115 | 0.1787 | 0.0124 | 0.2400 | 0.0000 | 0.0083 | 0.2567 | 0.0885 | 0.0000 | 0.0000 |
| MB-0145 | 0.0535 | 0.0000 | 0.0350 | 0.0324 | 0.0000 | 0.3411 | 0.0000 | 0.0234 | 0.0000 | 0.0368 | 0.0000 | 0.0000 | 0.0000 | 0.0000 | 0.0633 | 0.1729 | 0.0213 | 0.0000 | 0.2142 | 0.0061 | 0.0000 | 0.0000 |
| MB-0195 | 0.0000 | 0.0325 | 0.0211 | 0.0718 | 0.0000 | 0.0408 | 0.0006 | 0.1050 | 0.0000 | 0.0047 | 0.0000 | 0.0632 | 0.0141 | 0.1913 | 0.0926 | 0.2592 | 0.0000 | 0.0000 | 0.0970 | 0.0000 | 0.0000 | 0.0061 |
| MB-0422 | 0.0525 | 0.0000 | 0.0059 | 0.0459 | 0.0000 | 0.0168 | 0.0000 | 0.0694 | 0.0000 | 0.0556 | 0.0000 | 0.0830 | 0.0033 | 0.0831 | 0.0504 | 0.2619 | 0.0000 | 0.0000 | 0.0000 | 0.2712 | 0.0000 | 0.0011 |
| MB-0483 | 0.0313 | 0.0000 | 0.0000 | 0.0277 | 0.0000 | 0.1503 | 0.0000 | 0.0750 | 0.0000 | 0.1023 | 0.0000 | 0.1022 | 0.0507 | 0.1520 | 0.2024 | 0.0721 | 0.0178 | 0.0000 | 0.0024 | 0.0137 | 0.0000 | 0.0000 |
| MB-0317 | 0.0594 | 0.0000 | 0.0077 | 0.0089 | 0.0000 | 0.1273 | 0.0000 | 0.0443 | 0.0000 | 0.0000 | 0.0000 | 0.0591 | 0.0102 | 0.0880 | 0.0668 | 0.4808 | 0.0000 | 0.0000 | 0.0000 | 0.0409 | 0.0000 | 0.0066 |
| MB-0486 | 0.0000 | 0.0274 | 0.0000 | 0.1044 | 0.0000 | 0.0777 | 0.0000 | 0.0200 | 0.0000 | 0.1907 | 0.0000 | 0.0627 | 0.0211 | 0.0000 | 0.0623 | 0.2300 | 0.0203 | 0.0000 | 0.0637 | 0.1196 | 0.0000 | 0.0000 |
| MB-0139 | 0.0113 | 0.0000 | 0.0000 | 0.1401 | 0.0000 | 0.0187 | 0.0000 | 0.0447 | 0.0000 | 0.1490 | 0.0000 | 0.0000 | 0.0365 | 0.0273 | 0.0824 | 0.1026 | 0.0278 | 0.0000 | 0.3595 | 0.0000 | 0.0000 | 0.0000 |
| MB-0257 | 0.0786 | 0.0000 | 0.0059 | 0.0000 | 0.0000 | 0.1221 | 0.0000 | 0.0567 | 0.0000 | 0.0296 | 0.0000 | 0.0454 | 0.0150 | 0.0517 | 0.0835 | 0.3052 | 0.0519 | 0.0000 | 0.1545 | 0.0000 | 0.0000 | 0.0000 |
| MB-0345 | 0.0000 | 0.0000 | 0.0338 | 0.0265 | 0.0000 | 0.3148 | 0.0000 | 0.0066 | 0.0000 | 0.0000 | 0.0045 | 0.0069 | 0.0663 | 0.0000 | 0.0127 | 0.1749 | 0.0080 | 0.0000 | 0.3316 | 0.0134 | 0.0000 | 0.0000 |
| MB-0375 | 0.0271 | 0.0000 | 0.0000 | 0.1422 | 0.0000 | 0.0305 | 0.0529 | 0.0796 | 0.0000 | 0.1096 | 0.0000 | 0.0679 | 0.0417 | 0.0722 | 0.1197 | 0.1553 | 0.0355 | 0.0000 | 0.0657 | 0.0000 | 0.0000 | 0.0000 |
| MB-0419 | 0.0319 | 0.0000 | 0.0000 | 0.0059 | 0.0000 | 0.0786 | 0.0000 | 0.0101 | 0.0559 | 0.0000 | 0.0107 | 0.0429 | 0.0330 | 0.0287 | 0.0173 | 0.2197 | 0.0074 | 0.0000 | 0.4580 | 0.0000 | 0.0000 | 0.0000 |
| MB-0480 | 0.0172 | 0.0000 | 0.0106 | 0.0198 | 0.0000 | 0.0000 | 0.0000 | 0.0755 | 0.0116 | 0.0584 | 0.0000 | 0.0441 | 0.0000 | 0.2416 | 0.0399 | 0.1808 | 0.0000 | 0.0000 | 0.3004 | 0.0000 | 0.0000 | 0.0000 |
| MB-0311 | 0.0000 | 0.0008 | 0.0054 | 0.0000 | 0.0000 | 0.0000 | 0.0000 | 0.0483 | 0.0363 | 0.0147 | 0.0000 | 0.0331 | 0.0000 | 0.4999 | 0.0032 | 0.1216 | 0.0000 | 0.0000 | 0.2367 | 0.0000 | 0.0000 | 0.0000 |
| MB-0324 | 0.0000 | 0.0017 | 0.0260 | 0.0569 | 0.0000 | 0.2137 | 0.0000 | 0.0636 | 0.0000 | 0.0190 | 0.0000 | 0.0703 | 0.0342 | 0.0000 | 0.1093 | 0.1939 | 0.0180 | 0.0000 | 0.1900 | 0.0031 | 0.0000 | 0.0000 |
| MB-0368 | 0.0116 | 0.0000 | 0.0234 | 0.1145 | 0.0000 | 0.0000 | 0.0000 | 0.1363 | 0.0000 | 0.0600 | 0.0000 | 0.1255 | 0.0289 | 0.0000 | 0.1004 | 0.2469 | 0.0289 | 0.0000 | 0.0000 | 0.1236 | 0.0000 | 0.0000 |
| MB-0389 | 0.0000 | 0.0000 | 0.5043 | 0.0493 | 0.0000 | 0.0655 | 0.0000 | 0.0290 | 0.0000 | 0.0186 | 0.0000 | 0.0383 | 0.0020 | 0.0015 | 0.0494 | 0.1371 | 0.0000 | 0.0000 | 0.0744 | 0.0306 | 0.0000 | 0.0000 |
| MB-0248 | 0.0319 | 0.0000 | 0.0000 | 0.0791 | 0.0000 | 0.0499 | 0.0000 | 0.0856 | 0.0000 | 0.2117 | 0.0000 | 0.0178 | 0.0000 | 0.2249 | 0.0370 | 0.1387 | 0.0409 | 0.0000 | 0.0826 | 0.0000 | 0.0000 | 0.0000 |
| MB-0035 | 0.1195 | 0.0000 | 0.0690 | 0.0000 | 0.0000 | 0.2012 | 0.0000 | 0.0955 | 0.0000 | 0.0000 | 0.0101 | 0.1232 | 0.0941 | 0.0164 | 0.0128 | 0.1159 | 0.0065 | 0.0000 | 0.1198 | 0.0000 | 0.0158 | 0.0000 |
| MB-0904 | 0.0000 | 0.0000 | 0.0014 | 0.1292 | 0.0000 | 0.0000 | 0.0000 | 0.0836 | 0.0134 | 0.0239 | 0.0000 | 0.0339 | 0.0043 | 0.3294 | 0.1135 | 0.1929 | 0.0384 | 0.0000 | 0.0344 | 0.0017 | 0.0000 | 0.0000 |
| MB-0119 | 0.0000 | 0.0008 | 0.0167 | 0.0654 | 0.0000 | 0.0803 | 0.0000 | 0.0466 | 0.0000 | 0.0925 | 0.0000 | 0.1129 | 0.0213 | 0.0655 | 0.0902 | 0.2563 | 0.0125 | 0.0000 | 0.1391 | 0.0000 | 0.0000 | 0.0000 |
| MB-0650 | 0.0045 | 0.0262 | 0.0283 | 0.1540 | 0.0000 | 0.0395 | 0.0000 | 0.0927 | 0.0522 | 0.0977 | 0.0000 | 0.1222 | 0.0199 | 0.0431 | 0.0942 | 0.1652 | 0.0114 | 0.0000 | 0.0489 | 0.0000 | 0.0000 | 0.0000 |
| MB-0204 | 0.0164 | 0.0000 | 0.0083 | 0.0406 | 0.0000 | 0.0472 | 0.0000 | 0.0366 | 0.0000 | 0.0203 | 0.0000 | 0.0072 | 0.0176 | 0.2356 | 0.0284 | 0.2388 | 0.0000 | 0.0000 | 0.2429 | 0.0602 | 0.0000 | 0.0000 |
| MB-0184 | 0.0133 | 0.0000 | 0.0148 | 0.0422 | 0.0476 | 0.0000 | 0.0000 | 0.0237 | 0.0184 | 0.0181 | 0.0000 | 0.0708 | 0.0259 | 0.2293 | 0.0059 | 0.3775 | 0.0000 | 0.0000 | 0.0000 | 0.1127 | 0.0000 | 0.0000 |
| MB-0600 | 0.0227 | 0.0000 | 0.0098 | 0.0912 | 0.0000 | 0.1224 | 0.0000 | 0.0435 | 0.0211 | 0.0000 | 0.0000 | 0.0594 | 0.0000 | 0.0623 | 0.0546 | 0.2277 | 0.0000 | 0.0000 | 0.2852 | 0.0000 | 0.0000 | 0.0000 |
| MB-0400 | 0.0000 | 0.0118 | 0.0000 | 0.0047 | 0.0000 | 0.0809 | 0.0109 | 0.0441 | 0.0000 | 0.0695 | 0.0000 | 0.0199 | 0.0110 | 0.2698 | 0.1262 | 0.2720 | 0.0121 | 0.0000 | 0.0000 | 0.0672 | 0.0000 | 0.0000 |
| MB-0511 | 0.0001 | 0.0095 | 0.0000 | 0.0135 | 0.0000 | 0.0728 | 0.0000 | 0.0209 | 0.0000 | 0.0038 | 0.0000 | 0.0234 | 0.0277 | 0.1231 | 0.0777 | 0.1457 | 0.0459 | 0.0000 | 0.4359 | 0.0000 | 0.0000 | 0.0000 |
| MB-0500 | 0.0384 | 0.0000 | 0.0556 | 0.0000 | 0.0197 | 0.0000 | 0.0000 | 0.0757 | 0.0248 | 0.0000 | 0.0485 | 0.0000 | 0.0179 | 0.2958 | 0.1703 | 0.1965 | 0.0000 | 0.0000 | 0.0000 | 0.0568 | 0.0000 | 0.0000 |
| MB-0150 | 0.0016 | 0.0000 | 0.0062 | 0.0668 | 0.0000 | 0.0000 | 0.0234 | 0.0847 | 0.0125 | 0.0310 | 0.0000 | 0.1804 | 0.0000 | 0.1979 | 0.1499 | 0.1810 | 0.0217 | 0.0005 | 0.0422 | 0.0000 | 0.0000 | 0.0000 |
| MB-0895 | 0.0000 | 0.0158 | 0.0256 | 0.0528 | 0.0000 | 0.0866 | 0.0000 | 0.0657 | 0.0000 | 0.0598 | 0.0000 | 0.0512 | 0.0148 | 0.1479 | 0.1196 | 0.1329 | 0.0234 | 0.0000 | 0.2041 | 0.0000 | 0.0000 | 0.0000 |
| MB-0366 | 0.0169 | 0.0000 | 0.0151 | 0.0000 | 0.0000 | 0.0334 | 0.0000 | 0.0533 | 0.0211 | 0.0904 | 0.0000 | 0.0291 | 0.0108 | 0.3518 | 0.0793 | 0.1967 | 0.0000 | 0.0000 | 0.0763 | 0.0256 | 0.0000 | 0.0000 |
| MB-0173 | 0.0263 | 0.0000 | 0.0686 | 0.0123 | 0.0286 | 0.0214 | 0.0000 | 0.0544 | 0.0000 | 0.1535 | 0.0000 | 0.0000 | 0.0189 | 0.0349 | 0.0799 | 0.3810 | 0.0018 | 0.0000 | 0.0773 | 0.0411 | 0.0000 | 0.0000 |
| MB-0131 | 0.0021 | 0.0192 | 0.0000 | 0.0269 | 0.0000 | 0.0000 | 0.0000 | 0.0227 | 0.0532 | 0.0136 | 0.0105 | 0.0000 | 0.0000 | 0.1683 | 0.0073 | 0.5324 | 0.0005 | 0.0000 | 0.0000 | 0.1269 | 0.0006 | 0.0159 |
| MB-0206 | 0.0012 | 0.0000 | 0.0625 | 0.0522 | 0.0000 | 0.0000 | 0.0427 | 0.1370 | 0.0000 | 0.0853 | 0.0000 | 0.1073 | 0.0324 | 0.0820 | 0.1483 | 0.1624 | 0.0063 | 0.0000 | 0.0805 | 0.0000 | 0.0000 | 0.0000 |
| MB-0315 | 0.0730 | 0.0000 | 0.0550 | 0.0674 | 0.0000 | 0.0112 | 0.0000 | 0.0430 | 0.0139 | 0.0280 | 0.0000 | 0.0546 | 0.0000 | 0.1026 | 0.0356 | 0.1494 | 0.0000 | 0.0000 | 0.3663 | 0.0000 | 0.0000 | 0.0000 |
| MB-0361 | 0.0000 | 0.0537 | 0.0000 | 0.0805 | 0.0000 | 0.0461 | 0.0000 | 0.0686 | 0.0100 | 0.0359 | 0.0000 | 0.0599 | 0.0725 | 0.1329 | 0.1178 | 0.1118 | 0.0222 | 0.0000 | 0.1883 | 0.0000 | 0.0000 | 0.0000 |
| MB-0545 | 0.0000 | 0.0020 | 0.0005 | 0.1146 | 0.0000 | 0.0968 | 0.0023 | 0.0368 | 0.0000 | 0.0000 | 0.0000 | 0.0867 | 0.1444 | 0.0090 | 0.0937 | 0.1506 | 0.0074 | 0.0000 | 0.2522 | 0.0029 | 0.0000 | 0.0000 |
| MB-0370 | 0.0000 | 0.0068 | 0.0040 | 0.0657 | 0.0000 | 0.1376 | 0.0000 | 0.0426 | 0.0095 | 0.0000 | 0.0000 | 0.1095 | 0.2708 | 0.0494 | 0.1011 | 0.1451 | 0.0101 | 0.0000 | 0.0048 | 0.0431 | 0.0000 | 0.0000 |
| MB-0642 | 0.0085 | 0.0106 | 0.0000 | 0.0584 | 0.0565 | 0.0000 | 0.0000 | 0.0752 | 0.0000 | 0.0646 | 0.0000 | 0.0306 | 0.0000 | 0.2295 | 0.0248 | 0.2427 | 0.0071 | 0.0000 | 0.1582 | 0.0232 | 0.0000 | 0.0101 |
| MB-0431 | 0.0134 | 0.0000 | 0.0046 | 0.1681 | 0.0000 | 0.0914 | 0.0000 | 0.0310 | 0.0000 | 0.0000 | 0.0000 | 0.0375 | 0.0240 | 0.0000 | 0.0564 | 0.3123 | 0.0225 | 0.0000 | 0.1597 | 0.0790 | 0.0000 | 0.0000 |
| MB-0181 | 0.0000 | 0.0191 | 0.0226 | 0.0595 | 0.0000 | 0.0898 | 0.0000 | 0.0339 | 0.0000 | 0.0000 | 0.0000 | 0.0636 | 0.0427 | 0.0000 | 0.0198 | 0.1214 | 0.0041 | 0.0000 | 0.3830 | 0.1406 | 0.0000 | 0.0000 |
| MB-0603 | 0.0755 | 0.0000 | 0.0094 | 0.0397 | 0.0000 | 0.0875 | 0.0000 | 0.0297 | 0.0000 | 0.0000 | 0.0000 | 0.0084 | 0.0191 | 0.0437 | 0.0195 | 0.3049 | 0.0000 | 0.0000 | 0.3611 | 0.0000 | 0.0000 | 0.0016 |
| MB-0295 | 0.0131 | 0.0000 | 0.0027 | 0.0901 | 0.0030 | 0.0126 | 0.0000 | 0.0653 | 0.0000 | 0.0889 | 0.0000 | 0.0648 | 0.0497 | 0.1222 | 0.1268 | 0.1309 | 0.0415 | 0.0000 | 0.1883 | 0.0000 | 0.0000 | 0.0000 |
| MB-0618 | 0.0382 | 0.0000 | 0.0146 | 0.0069 | 0.0000 | 0.0642 | 0.0000 | 0.0852 | 0.0000 | 0.0000 | 0.0000 | 0.0835 | 0.1159 | 0.0000 | 0.0600 | 0.2532 | 0.0082 | 0.0000 | 0.0000 | 0.2702 | 0.0000 | 0.0000 |
| MB-0496 | 0.0186 | 0.0000 | 0.0612 | 0.0211 | 0.0000 | 0.1514 | 0.0000 | 0.0444 | 0.0000 | 0.0561 | 0.0000 | 0.0331 | 0.1150 | 0.0000 | 0.0554 | 0.1692 | 0.0000 | 0.0000 | 0.2479 | 0.0265 | 0.0000 | 0.0000 |
| MB-0411 | 0.0138 | 0.0000 | 0.0567 | 0.0614 | 0.0088 | 0.0159 | 0.0000 | 0.0858 | 0.0000 | 0.0994 | 0.0000 | 0.0111 | 0.0000 | 0.1951 | 0.0889 | 0.2208 | 0.0000 | 0.0000 | 0.1128 | 0.0295 | 0.0000 | 0.0000 |
| MB-0285 | 0.0081 | 0.0000 | 0.0020 | 0.0528 | 0.0000 | 0.1039 | 0.0000 | 0.0259 | 0.0000 | 0.0054 | 0.0000 | 0.0724 | 0.1603 | 0.0000 | 0.0184 | 0.3251 | 0.0018 | 0.0000 | 0.1846 | 0.0392 | 0.0000 | 0.0000 |
| MB-0360 | 0.0000 | 0.0154 | 0.0000 | 0.0109 | 0.0000 | 0.0237 | 0.0000 | 0.0512 | 0.0077 | 0.0000 | 0.0000 | 0.0513 | 0.1946 | 0.0000 | 0.0181 | 0.5355 | 0.0201 | 0.0000 | 0.0558 | 0.0000 | 0.0118 | 0.0038 |
| MB-0359 | 0.0450 | 0.0000 | 0.0166 | 0.0256 | 0.0415 | 0.1669 | 0.0000 | 0.0745 | 0.0077 | 0.0596 | 0.0000 | 0.0536 | 0.0335 | 0.1622 | 0.1175 | 0.1018 | 0.0033 | 0.0000 | 0.0908 | 0.0000 | 0.0000 | 0.0000 |
| MB-0344 | 0.0281 | 0.0000 | 0.0247 | 0.0905 | 0.0000 | 0.1058 | 0.0000 | 0.0520 | 0.0000 | 0.1366 | 0.0000 | 0.1398 | 0.0261 | 0.0000 | 0.0917 | 0.1249 | 0.0135 | 0.0000 | 0.1662 | 0.0000 | 0.0000 | 0.0000 |
| MB-0583 | 0.0504 | 0.0000 | 0.0137 | 0.0751 | 0.0000 | 0.0790 | 0.0000 | 0.0307 | 0.0090 | 0.0124 | 0.0000 | 0.0383 | 0.0222 | 0.0000 | 0.0673 | 0.3016 | 0.0350 | 0.0000 | 0.2651 | 0.0000 | 0.0000 | 0.0000 |
| MB-0202 | 0.0000 | 0.0495 | 0.0000 | 0.1182 | 0.0000 | 0.0163 | 0.0000 | 0.0394 | 0.0000 | 0.0000 | 0.0000 | 0.0361 | 0.0227 | 0.0320 | 0.0730 | 0.4274 | 0.0237 | 0.0000 | 0.0337 | 0.1281 | 0.0000 | 0.0000 |
| MB-0485 | 0.0000 | 0.0000 | 0.1242 | 0.0096 | 0.0000 | 0.2034 | 0.0000 | 0.0287 | 0.0336 | 0.0655 | 0.0000 | 0.0716 | 0.0356 | 0.0000 | 0.1061 | 0.1435 | 0.0092 | 0.0000 | 0.1689 | 0.0000 | 0.0000 | 0.0000 |
| MB-0609 | 0.0000 | 0.0000 | 0.0094 | 0.0563 | 0.0000 | 0.0184 | 0.0000 | 0.0722 | 0.0000 | 0.0372 | 0.0000 | 0.0706 | 0.0410 | 0.1079 | 0.1277 | 0.1089 | 0.0165 | 0.0000 | 0.3339 | 0.0000 | 0.0000 | 0.0000 |
| MB-0538 | 0.0032 | 0.0082 | 0.0289 | 0.1208 | 0.0000 | 0.1034 | 0.0277 | 0.0569 | 0.0000 | 0.1178 | 0.0000 | 0.0000 | 0.1580 | 0.0119 | 0.1347 | 0.1941 | 0.0118 | 0.0000 | 0.0150 | 0.0000 | 0.0076 | 0.0000 |
| MB-0197 | 0.0144 | 0.0000 | 0.0281 | 0.0944 | 0.0000 | 0.0778 | 0.0192 | 0.0396 | 0.0350 | 0.0611 | 0.0000 | 0.0683 | 0.0076 | 0.1800 | 0.1244 | 0.2157 | 0.0000 | 0.0000 | 0.0343 | 0.0000 | 0.0000 | 0.0000 |
| MB-0410 | 0.0255 | 0.0000 | 0.0172 | 0.1443 | 0.0000 | 0.1475 | 0.0000 | 0.0581 | 0.0069 | 0.0836 | 0.0000 | 0.0886 | 0.0000 | 0.0820 | 0.1129 | 0.0576 | 0.0000 | 0.0000 | 0.1757 | 0.0000 | 0.0000 | 0.0000 |
| MB-0528 | 0.0033 | 0.0078 | 0.0000 | 0.0559 | 0.0000 | 0.0510 | 0.0000 | 0.1109 | 0.0169 | 0.1838 | 0.0000 | 0.1136 | 0.0000 | 0.0000 | 0.1231 | 0.3069 | 0.0072 | 0.0000 | 0.0000 | 0.0197 | 0.0000 | 0.0000 |
| MB-0165 | 0.0274 | 0.0000 | 0.0879 | 0.0487 | 0.0280 | 0.1258 | 0.0000 | 0.0186 | 0.0000 | 0.0468 | 0.0000 | 0.0977 | 0.0000 | 0.1518 | 0.1483 | 0.0720 | 0.0000 | 0.0000 | 0.1470 | 0.0000 | 0.0000 | 0.0000 |
| MB-0152 | 0.0189 | 0.0000 | 0.1698 | 0.0217 | 0.0000 | 0.0414 | 0.0000 | 0.0695 | 0.0122 | 0.0230 | 0.0095 | 0.0374 | 0.0021 | 0.2355 | 0.2023 | 0.0953 | 0.0000 | 0.0000 | 0.0616 | 0.0000 | 0.0000 | 0.0000 |
| MB-0148 | 0.0020 | 0.0000 | 0.1783 | 0.0356 | 0.0000 | 0.0676 | 0.0000 | 0.1250 | 0.0000 | 0.0314 | 0.0000 | 0.0957 | 0.0000 | 0.0959 | 0.1148 | 0.1411 | 0.0496 | 0.0000 | 0.0611 | 0.0000 | 0.0000 | 0.0019 |
| MB-0594 | 0.0043 | 0.0000 | 0.2314 | 0.0733 | 0.0000 | 0.0000 | 0.0000 | 0.0593 | 0.0000 | 0.0703 | 0.0000 | 0.1008 | 0.0092 | 0.1496 | 0.1108 | 0.0354 | 0.0653 | 0.0000 | 0.0904 | 0.0000 | 0.0000 | 0.0000 |
| MB-0521 | 0.0193 | 0.0000 | 0.1669 | 0.0572 | 0.0000 | 0.2061 | 0.0000 | 0.0450 | 0.0000 | 0.0485 | 0.0000 | 0.0493 | 0.0372 | 0.0000 | 0.1399 | 0.1250 | 0.0265 | 0.0000 | 0.0790 | 0.0000 | 0.0000 | 0.0000 |
| MB-0532 | 0.0073 | 0.0063 | 0.0000 | 0.0895 | 0.0000 | 0.0909 | 0.0084 | 0.0303 | 0.0472 | 0.1571 | 0.0000 | 0.1004 | 0.0000 | 0.0728 | 0.1508 | 0.1615 | 0.0000 | 0.0000 | 0.0774 | 0.0000 | 0.0000 | 0.0000 |
| MB-0536 | 0.0181 | 0.0000 | 0.0000 | 0.0355 | 0.0000 | 0.0435 | 0.0000 | 0.0807 | 0.0001 | 0.1227 | 0.0000 | 0.0950 | 0.0242 | 0.0855 | 0.1663 | 0.2007 | 0.0564 | 0.0000 | 0.0712 | 0.0000 | 0.0000 | 0.0000 |
| MB-0319 | 0.0424 | 0.0000 | 0.0165 | 0.0626 | 0.0000 | 0.0846 | 0.0000 | 0.0684 | 0.0000 | 0.0672 | 0.0000 | 0.0662 | 0.0000 | 0.0000 | 0.0491 | 0.3817 | 0.0090 | 0.0000 | 0.1478 | 0.0000 | 0.0000 | 0.0045 |
| MB-0491 | 0.0149 | 0.0000 | 0.0028 | 0.0427 | 0.0000 | 0.1717 | 0.0000 | 0.0666 | 0.0000 | 0.0889 | 0.0000 | 0.0852 | 0.0257 | 0.0804 | 0.1248 | 0.1574 | 0.0169 | 0.0000 | 0.1219 | 0.0000 | 0.0000 | 0.0000 |
| MB-0404 | 0.0663 | 0.0000 | 0.0234 | 0.0199 | 0.0000 | 0.1065 | 0.0000 | 0.0342 | 0.0000 | 0.0370 | 0.0000 | 0.0146 | 0.0088 | 0.1095 | 0.0510 | 0.3378 | 0.0000 | 0.0000 | 0.1891 | 0.0018 | 0.0000 | 0.0000 |
| MB-0243 | 0.0431 | 0.0000 | 0.0080 | 0.0000 | 0.0000 | 0.1328 | 0.0000 | 0.0431 | 0.0000 | 0.0649 | 0.0000 | 0.0235 | 0.0209 | 0.1112 | 0.1068 | 0.2949 | 0.0071 | 0.0000 | 0.1019 | 0.0417 | 0.0000 | 0.0000 |
| MB-0580 | 0.0065 | 0.0000 | 0.1809 | 0.0441 | 0.0000 | 0.1041 | 0.0000 | 0.0306 | 0.0000 | 0.1866 | 0.0000 | 0.1066 | 0.0000 | 0.0107 | 0.1238 | 0.0964 | 0.0000 | 0.0000 | 0.1097 | 0.0000 | 0.0000 | 0.0000 |
| MB-0312 | 0.0446 | 0.0000 | 0.0122 | 0.0786 | 0.0000 | 0.0688 | 0.0000 | 0.0211 | 0.0000 | 0.0238 | 0.0000 | 0.0128 | 0.0000 | 0.0546 | 0.0170 | 0.3247 | 0.0000 | 0.0000 | 0.2694 | 0.0716 | 0.0000 | 0.0007 |
| MB-0060 | 0.0006 | 0.0000 | 0.0116 | 0.1587 | 0.0000 | 0.0000 | 0.0333 | 0.1044 | 0.0223 | 0.0143 | 0.0000 | 0.1432 | 0.0219 | 0.2650 | 0.0803 | 0.1272 | 0.0059 | 0.0000 | 0.0113 | 0.0000 | 0.0000 | 0.0000 |
| MB-0649 | 0.0332 | 0.0000 | 0.0305 | 0.0728 | 0.0000 | 0.1203 | 0.0000 | 0.0472 | 0.0307 | 0.0236 | 0.0000 | 0.0472 | 0.0174 | 0.0896 | 0.0759 | 0.0823 | 0.0061 | 0.0000 | 0.3230 | 0.0000 | 0.0000 | 0.0000 |
| MB-0643 | 0.0289 | 0.0000 | 0.1218 | 0.0000 | 0.0000 | 0.0640 | 0.0609 | 0.1113 | 0.0000 | 0.0889 | 0.0000 | 0.0837 | 0.0000 | 0.1134 | 0.1566 | 0.0693 | 0.0028 | 0.0000 | 0.0915 | 0.0000 | 0.0000 | 0.0068 |
| MB-0581 | 0.0724 | 0.0000 | 0.1604 | 0.0259 | 0.0000 | 0.0639 | 0.0000 | 0.0931 | 0.0131 | 0.0000 | 0.0000 | 0.0638 | 0.0000 | 0.2016 | 0.0503 | 0.1053 | 0.0000 | 0.0257 | 0.0000 | 0.1134 | 0.0000 | 0.0111 |
| MB-0383 | 0.0000 | 0.0000 | 0.0559 | 0.0160 | 0.0000 | 0.0000 | 0.0000 | 0.0489 | 0.0183 | 0.0686 | 0.0000 | 0.0078 | 0.0000 | 0.4468 | 0.0539 | 0.2391 | 0.0000 | 0.0000 | 0.0348 | 0.0101 | 0.0000 | 0.0000 |
| MB-0142 | 0.0345 | 0.0000 | 0.0387 | 0.0173 | 0.0000 | 0.1032 | 0.0000 | 0.0930 | 0.0000 | 0.0174 | 0.0000 | 0.0381 | 0.0296 | 0.0091 | 0.0382 | 0.3228 | 0.0107 | 0.0000 | 0.2390 | 0.0085 | 0.0000 | 0.0000 |
| MB-0320 | 0.0000 | 0.0000 | 0.0682 | 0.0985 | 0.0000 | 0.0555 | 0.0000 | 0.0199 | 0.0000 | 0.0049 | 0.0000 | 0.0559 | 0.0421 | 0.0000 | 0.0300 | 0.1381 | 0.0028 | 0.0000 | 0.4841 | 0.0000 | 0.0000 | 0.0000 |
| MB-0207 | 0.0044 | 0.0291 | 0.0000 | 0.0189 | 0.0000 | 0.1337 | 0.0000 | 0.0663 | 0.0000 | 0.0507 | 0.0000 | 0.0121 | 0.0461 | 0.0898 | 0.0613 | 0.1266 | 0.0227 | 0.0000 | 0.2642 | 0.0742 | 0.0000 | 0.0000 |
| MB-0167 | 0.0000 | 0.0307 | 0.1139 | 0.0771 | 0.0000 | 0.0000 | 0.0000 | 0.0501 | 0.0265 | 0.0692 | 0.0000 | 0.0122 | 0.0183 | 0.2333 | 0.1121 | 0.1433 | 0.0000 | 0.0000 | 0.1132 | 0.0000 | 0.0000 | 0.0000 |
| MB-0591 | 0.0367 | 0.0000 | 0.0000 | 0.0016 | 0.0464 | 0.0000 | 0.0000 | 0.0247 | 0.0402 | 0.0530 | 0.0000 | 0.0000 | 0.0000 | 0.4804 | 0.0294 | 0.2179 | 0.0000 | 0.0000 | 0.0389 | 0.0307 | 0.0000 | 0.0000 |
| MB-0613 | 0.0000 | 0.0049 | 0.0030 | 0.0000 | 0.0000 | 0.0472 | 0.0000 | 0.0083 | 0.0418 | 0.0276 | 0.0000 | 0.0312 | 0.0000 | 0.3025 | 0.0248 | 0.3976 | 0.0000 | 0.0000 | 0.1111 | 0.0000 | 0.0000 | 0.0000 |
| MB-0313 | 0.0044 | 0.0000 | 0.0064 | 0.0376 | 0.0000 | 0.0527 | 0.0331 | 0.0742 | 0.0000 | 0.0051 | 0.0000 | 0.0135 | 0.0394 | 0.2560 | 0.0931 | 0.1635 | 0.0000 | 0.0000 | 0.1503 | 0.0707 | 0.0000 | 0.0000 |
| MB-0599 | 0.0738 | 0.0000 | 0.1018 | 0.0593 | 0.0000 | 0.0353 | 0.0000 | 0.0470 | 0.0000 | 0.0246 | 0.0000 | 0.0187 | 0.0000 | 0.0274 | 0.0418 | 0.1921 | 0.0000 | 0.0000 | 0.3782 | 0.0000 | 0.0000 | 0.0000 |
| MB-0286 | 0.0000 | 0.0099 | 0.0046 | 0.1265 | 0.0000 | 0.0434 | 0.0000 | 0.0183 | 0.0180 | 0.0889 | 0.0000 | 0.0841 | 0.0110 | 0.0231 | 0.0694 | 0.1722 | 0.0054 | 0.0000 | 0.3253 | 0.0000 | 0.0000 | 0.0000 |
| MB-0575 | 0.0266 | 0.0000 | 0.0163 | 0.0402 | 0.0000 | 0.0443 | 0.0000 | 0.1034 | 0.0000 | 0.0683 | 0.0000 | 0.0413 | 0.0000 | 0.2622 | 0.0844 | 0.1714 | 0.0000 | 0.0000 | 0.1416 | 0.0000 | 0.0000 | 0.0000 |
| MB-0214 | 0.0145 | 0.0042 | 0.0000 | 0.0278 | 0.0000 | 0.0756 | 0.0190 | 0.1294 | 0.0000 | 0.0771 | 0.0000 | 0.0000 | 0.0461 | 0.1178 | 0.1286 | 0.1875 | 0.0748 | 0.0053 | 0.0922 | 0.0000 | 0.0000 | 0.0000 |
| MB-0589 | 0.0275 | 0.0000 | 0.0079 | 0.0078 | 0.0000 | 0.0850 | 0.0000 | 0.1017 | 0.0000 | 0.1189 | 0.0000 | 0.0106 | 0.0446 | 0.0588 | 0.1498 | 0.1868 | 0.0406 | 0.0000 | 0.1599 | 0.0000 | 0.0000 | 0.0000 |
| MB-0406 | 0.0267 | 0.0000 | 0.0003 | 0.0344 | 0.0000 | 0.0871 | 0.0000 | 0.0528 | 0.0091 | 0.0000 | 0.0000 | 0.0468 | 0.0243 | 0.0544 | 0.0250 | 0.3580 | 0.0046 | 0.0000 | 0.2748 | 0.0000 | 0.0000 | 0.0017 |
| MB-0452 | 0.0171 | 0.0173 | 0.0000 | 0.0347 | 0.0000 | 0.0000 | 0.0004 | 0.1316 | 0.0503 | 0.1389 | 0.0000 | 0.0211 | 0.0504 | 0.1327 | 0.0904 | 0.2621 | 0.0000 | 0.0000 | 0.0000 | 0.0531 | 0.0000 | 0.0000 |
| MB-0524 | 0.0000 | 0.0389 | 0.0000 | 0.0136 | 0.0000 | 0.0895 | 0.0000 | 0.0634 | 0.0000 | 0.1509 | 0.0000 | 0.0118 | 0.0196 | 0.2915 | 0.0972 | 0.1002 | 0.0220 | 0.0000 | 0.0000 | 0.1014 | 0.0000 | 0.0000 |
| MB-0880 | 0.0000 | 0.0030 | 0.0000 | 0.0041 | 0.0000 | 0.0000 | 0.0000 | 0.0552 | 0.0171 | 0.0204 | 0.0000 | 0.0311 | 0.0238 | 0.4469 | 0.0110 | 0.2965 | 0.0029 | 0.0000 | 0.0000 | 0.0879 | 0.0000 | 0.0000 |
| MB-0573 | 0.0121 | 0.0643 | 0.0000 | 0.0955 | 0.0000 | 0.1257 | 0.0000 | 0.0380 | 0.0000 | 0.0000 | 0.0000 | 0.0860 | 0.0312 | 0.0984 | 0.0344 | 0.0304 | 0.0059 | 0.0000 | 0.3782 | 0.0000 | 0.0000 | 0.0000 |
| MB-0664 | 0.0000 | 0.0168 | 0.0000 | 0.0374 | 0.0000 | 0.0739 | 0.0000 | 0.0918 | 0.0000 | 0.0044 | 0.0000 | 0.0487 | 0.0006 | 0.3636 | 0.0681 | 0.1791 | 0.0000 | 0.0465 | 0.0000 | 0.0692 | 0.0000 | 0.0000 |
| MB-0446 | 0.0000 | 0.0000 | 0.0240 | 0.0038 | 0.0000 | 0.0656 | 0.0000 | 0.0445 | 0.0000 | 0.0173 | 0.0000 | 0.0698 | 0.2057 | 0.2247 | 0.1669 | 0.1449 | 0.0000 | 0.0000 | 0.0328 | 0.0000 | 0.0000 | 0.0000 |
| MB-0008 | 0.0020 | 0.0000 | 0.0332 | 0.0022 | 0.0000 | 0.0893 | 0.0000 | 0.0264 | 0.0000 | 0.0869 | 0.0000 | 0.0820 | 0.0403 | 0.0603 | 0.0742 | 0.1799 | 0.0152 | 0.0000 | 0.3080 | 0.0000 | 0.0000 | 0.0000 |
| MB-0656 | 0.0229 | 0.0000 | 0.0600 | 0.0000 | 0.0000 | 0.1473 | 0.0124 | 0.0193 | 0.0000 | 0.1145 | 0.0000 | 0.0199 | 0.1334 | 0.0838 | 0.1557 | 0.1472 | 0.0000 | 0.0000 | 0.0635 | 0.0199 | 0.0000 | 0.0000 |
| MB-0154 | 0.0035 | 0.0000 | 0.0271 | 0.0397 | 0.0000 | 0.0401 | 0.0000 | 0.0851 | 0.0000 | 0.1204 | 0.0000 | 0.1258 | 0.0280 | 0.0000 | 0.1151 | 0.1662 | 0.0443 | 0.0000 | 0.0000 | 0.2046 | 0.0000 | 0.0000 |
| MB-0597 | 0.0206 | 0.0000 | 0.0432 | 0.0333 | 0.0000 | 0.2612 | 0.0000 | 0.0000 | 0.0000 | 0.0374 | 0.0000 | 0.0405 | 0.1246 | 0.0000 | 0.0394 | 0.1845 | 0.0068 | 0.0000 | 0.1256 | 0.0829 | 0.0000 | 0.0000 |
| MB-0550 | 0.0097 | 0.0077 | 0.0000 | 0.0436 | 0.0000 | 0.0452 | 0.0000 | 0.1234 | 0.0405 | 0.1238 | 0.0000 | 0.0295 | 0.0000 | 0.2854 | 0.0745 | 0.1461 | 0.0000 | 0.0000 | 0.0087 | 0.0619 | 0.0000 | 0.0000 |
| MB-0616 | 0.0078 | 0.0012 | 0.0000 | 0.0358 | 0.0000 | 0.0483 | 0.0000 | 0.0587 | 0.0000 | 0.0053 | 0.0000 | 0.0482 | 0.0039 | 0.0524 | 0.0251 | 0.2337 | 0.0051 | 0.0000 | 0.4746 | 0.0000 | 0.0000 | 0.0000 |
| MB-0412 | 0.0014 | 0.0180 | 0.0000 | 0.0520 | 0.0000 | 0.0593 | 0.0026 | 0.0257 | 0.0000 | 0.0994 | 0.0000 | 0.0091 | 0.0000 | 0.1211 | 0.1317 | 0.1399 | 0.0089 | 0.0000 | 0.3280 | 0.0000 | 0.0000 | 0.0029 |
| MB-0122 | 0.0035 | 0.0000 | 0.0181 | 0.1015 | 0.0000 | 0.0768 | 0.0000 | 0.0639 | 0.0000 | 0.0892 | 0.0000 | 0.0000 | 0.0176 | 0.0999 | 0.0260 | 0.1696 | 0.0542 | 0.0000 | 0.2796 | 0.0000 | 0.0000 | 0.0000 |
| MB-0425 | 0.0190 | 0.0000 | 0.0041 | 0.0181 | 0.0000 | 0.0285 | 0.0000 | 0.0444 | 0.0000 | 0.0545 | 0.0000 | 0.0325 | 0.0000 | 0.2604 | 0.0324 | 0.3777 | 0.0025 | 0.0000 | 0.0617 | 0.0643 | 0.0000 | 0.0000 |
| MB-0314 | 0.0501 | 0.0000 | 0.0130 | 0.0000 | 0.0000 | 0.0000 | 0.0000 | 0.0809 | 0.0397 | 0.0382 | 0.0054 | 0.0000 | 0.0000 | 0.4122 | 0.0603 | 0.2293 | 0.0000 | 0.0000 | 0.0324 | 0.0252 | 0.0000 | 0.0132 |
| MB-0356 | 0.0000 | 0.0797 | 0.0000 | 0.2081 | 0.0000 | 0.0000 | 0.0000 | 0.0646 | 0.0000 | 0.0949 | 0.0000 | 0.0000 | 0.0209 | 0.0297 | 0.1244 | 0.1035 | 0.0288 | 0.0000 | 0.1875 | 0.0579 | 0.0000 | 0.0000 |
| MB-0440 | 0.0175 | 0.0000 | 0.0013 | 0.0008 | 0.0000 | 0.1497 | 0.0000 | 0.0713 | 0.0178 | 0.0012 | 0.0000 | 0.0416 | 0.0189 | 0.2046 | 0.0588 | 0.2419 | 0.0000 | 0.0000 | 0.1190 | 0.0555 | 0.0000 | 0.0000 |
| MB-0398 | 0.0135 | 0.0000 | 0.0000 | 0.0656 | 0.0000 | 0.0486 | 0.0000 | 0.0708 | 0.0000 | 0.2370 | 0.0000 | 0.1067 | 0.0000 | 0.1301 | 0.1132 | 0.1188 | 0.0065 | 0.0000 | 0.0891 | 0.0000 | 0.0000 | 0.0000 |
| MB-0438 | 0.0184 | 0.0000 | 0.1366 | 0.0755 | 0.0000 | 0.0262 | 0.0000 | 0.0133 | 0.0275 | 0.1366 | 0.0000 | 0.0808 | 0.0000 | 0.1546 | 0.1093 | 0.1079 | 0.0276 | 0.0000 | 0.0859 | 0.0000 | 0.0000 | 0.0000 |
| MB-0449 | 0.0168 | 0.0096 | 0.0000 | 0.0278 | 0.0000 | 0.1154 | 0.0000 | 0.0691 | 0.0000 | 0.1163 | 0.0000 | 0.0000 | 0.0244 | 0.2468 | 0.0400 | 0.1800 | 0.0000 | 0.0000 | 0.1174 | 0.0364 | 0.0000 | 0.0000 |
| MB-0162 | 0.0000 | 0.0140 | 0.0910 | 0.0967 | 0.0000 | 0.0260 | 0.0000 | 0.0528 | 0.0000 | 0.0127 | 0.0000 | 0.0791 | 0.0000 | 0.0350 | 0.0527 | 0.1363 | 0.0012 | 0.0000 | 0.4025 | 0.0000 | 0.0000 | 0.0000 |
| MB-0593 | 0.0025 | 0.0000 | 0.0733 | 0.2405 | 0.0000 | 0.0041 | 0.0000 | 0.0600 | 0.0440 | 0.0190 | 0.0000 | 0.1077 | 0.0460 | 0.0000 | 0.1390 | 0.1633 | 0.0060 | 0.0000 | 0.0945 | 0.0000 | 0.0000 | 0.0000 |
| MB-0301 | 0.0000 | 0.0000 | 0.0657 | 0.0000 | 0.0000 | 0.0000 | 0.0000 | 0.0444 | 0.0235 | 0.0446 | 0.0000 | 0.0181 | 0.0000 | 0.2364 | 0.0379 | 0.3744 | 0.0000 | 0.0000 | 0.1251 | 0.0257 | 0.0000 | 0.0043 |
| MB-0628 | 0.0000 | 0.0194 | 0.0000 | 0.0255 | 0.0010 | 0.0374 | 0.0000 | 0.0000 | 0.0413 | 0.0000 | 0.0265 | 0.0000 | 0.0000 | 0.2681 | 0.0090 | 0.1435 | 0.0000 | 0.0000 | 0.4284 | 0.0000 | 0.0000 | 0.0000 |
| MB-0106 | 0.0000 | 0.0000 | 0.0242 | 0.0892 | 0.0000 | 0.0000 | 0.0000 | 0.0844 | 0.0000 | 0.0122 | 0.0000 | 0.0390 | 0.0050 | 0.3190 | 0.0334 | 0.2833 | 0.0064 | 0.0000 | 0.0000 | 0.1038 | 0.0000 | 0.0000 |
| MB-0341 | 0.0447 | 0.0000 | 0.0202 | 0.0283 | 0.0000 | 0.0505 | 0.0000 | 0.0922 | 0.0000 | 0.0731 | 0.0000 | 0.0376 | 0.0015 | 0.1345 | 0.1207 | 0.2487 | 0.0000 | 0.0000 | 0.1480 | 0.0000 | 0.0000 | 0.0000 |
| MB-0474 | 0.0000 | 0.1178 | 0.0000 | 0.1374 | 0.0180 | 0.0000 | 0.0002 | 0.0546 | 0.0000 | 0.1755 | 0.0000 | 0.0552 | 0.0108 | 0.0674 | 0.1079 | 0.1734 | 0.0197 | 0.0000 | 0.0553 | 0.0069 | 0.0000 | 0.0000 |
| MB-0394 | 0.0047 | 0.0000 | 0.0652 | 0.0309 | 0.0000 | 0.0770 | 0.0000 | 0.0539 | 0.0331 | 0.0755 | 0.0000 | 0.0601 | 0.0441 | 0.1436 | 0.0848 | 0.2822 | 0.0120 | 0.0000 | 0.0186 | 0.0143 | 0.0000 | 0.0000 |
| MB-0373 | 0.0000 | 0.0000 | 0.0061 | 0.0620 | 0.0309 | 0.0000 | 0.0088 | 0.0851 | 0.0527 | 0.0021 | 0.0000 | 0.0745 | 0.0308 | 0.0837 | 0.0777 | 0.2154 | 0.0825 | 0.0000 | 0.1877 | 0.0000 | 0.0000 | 0.0000 |
| MB-0247 | 0.0000 | 0.0208 | 0.0280 | 0.0687 | 0.0000 | 0.0190 | 0.0000 | 0.0512 | 0.0000 | 0.0789 | 0.0000 | 0.0175 | 0.0000 | 0.1333 | 0.0547 | 0.1165 | 0.0082 | 0.0000 | 0.4032 | 0.0000 | 0.0000 | 0.0000 |
| MB-0225 | 0.0000 | 0.0104 | 0.0000 | 0.0274 | 0.0000 | 0.0480 | 0.0000 | 0.0360 | 0.0000 | 0.0202 | 0.0000 | 0.0282 | 0.0055 | 0.0398 | 0.0375 | 0.2378 | 0.0228 | 0.0000 | 0.4864 | 0.0000 | 0.0000 | 0.0000 |
| MB-0424 | 0.0000 | 0.0000 | 0.0954 | 0.0118 | 0.0000 | 0.0521 | 0.0000 | 0.0219 | 0.0000 | 0.0624 | 0.0000 | 0.0499 | 0.0308 | 0.1525 | 0.0449 | 0.2448 | 0.0078 | 0.0000 | 0.2259 | 0.0000 | 0.0000 | 0.0000 |
| MB-0209 | 0.0000 | 0.0090 | 0.0000 | 0.0000 | 0.0000 | 0.0005 | 0.0000 | 0.0078 | 0.0010 | 0.0137 | 0.0050 | 0.0011 | 0.0000 | 0.7740 | 0.0247 | 0.1015 | 0.0048 | 0.0000 | 0.0552 | 0.0000 | 0.0018 | 0.0000 |
| MB-0115 | 0.0255 | 0.0000 | 0.0310 | 0.0000 | 0.0000 | 0.0453 | 0.0188 | 0.1084 | 0.0000 | 0.1087 | 0.0000 | 0.0684 | 0.0000 | 0.2800 | 0.2322 | 0.0241 | 0.0000 | 0.0000 | 0.0576 | 0.0000 | 0.0000 | 0.0000 |
| MB-0136 | 0.0043 | 0.0000 | 0.0174 | 0.1042 | 0.0000 | 0.0932 | 0.0129 | 0.0664 | 0.0072 | 0.0873 | 0.0000 | 0.0942 | 0.0169 | 0.0915 | 0.0874 | 0.1526 | 0.0196 | 0.0000 | 0.1448 | 0.0000 | 0.0000 | 0.0000 |
| MB-0542 | 0.0515 | 0.0000 | 0.0393 | 0.0790 | 0.0000 | 0.1853 | 0.0320 | 0.0519 | 0.0000 | 0.0661 | 0.0000 | 0.0493 | 0.0219 | 0.1273 | 0.1328 | 0.0746 | 0.0000 | 0.0000 | 0.0890 | 0.0000 | 0.0000 | 0.0000 |
| MB-0354 | 0.0317 | 0.0000 | 0.0138 | 0.1014 | 0.0000 | 0.0000 | 0.0621 | 0.0961 | 0.0000 | 0.1533 | 0.0000 | 0.0583 | 0.0094 | 0.1022 | 0.1165 | 0.0983 | 0.0040 | 0.0000 | 0.1530 | 0.0000 | 0.0000 | 0.0000 |
| MB-0151 | 0.0040 | 0.0036 | 0.0027 | 0.0779 | 0.0000 | 0.0071 | 0.0000 | 0.0709 | 0.0255 | 0.0500 | 0.0000 | 0.0839 | 0.0000 | 0.1461 | 0.1305 | 0.2376 | 0.0042 | 0.0000 | 0.1560 | 0.0000 | 0.0000 | 0.0000 |
| MB-0590 | 0.0407 | 0.0000 | 0.0103 | 0.0836 | 0.0000 | 0.0000 | 0.0054 | 0.0976 | 0.0379 | 0.0063 | 0.0000 | 0.0782 | 0.0514 | 0.2373 | 0.1088 | 0.1658 | 0.0000 | 0.0000 | 0.0767 | 0.0000 | 0.0000 | 0.0000 |
| MB-0608 | 0.0000 | 0.0000 | 0.3880 | 0.1174 | 0.0000 | 0.0000 | 0.0424 | 0.0192 | 0.0133 | 0.0000 | 0.0000 | 0.0638 | 0.0000 | 0.1278 | 0.1200 | 0.1072 | 0.0000 | 0.0000 | 0.0000 | 0.0000 | 0.0000 | 0.0010 |
| MB-0657 | 0.0434 | 0.0000 | 0.0197 | 0.0694 | 0.0000 | 0.0845 | 0.0040 | 0.0955 | 0.0000 | 0.0000 | 0.0000 | 0.1218 | 0.1471 | 0.0000 | 0.0687 | 0.0902 | 0.0053 | 0.0000 | 0.2257 | 0.0247 | 0.0000 | 0.0000 |
| MB-0559 | 0.0292 | 0.0000 | 0.0102 | 0.0000 | 0.0066 | 0.0394 | 0.0000 | 0.0119 | 0.0000 | 0.0000 | 0.0000 | 0.0437 | 0.0000 | 0.0695 | 0.0078 | 0.2681 | 0.0000 | 0.0000 | 0.5038 | 0.0000 | 0.0000 | 0.0098 |
| MB-0893 | 0.0000 | 0.0000 | 0.0000 | 0.0121 | 0.0000 | 0.0372 | 0.0141 | 0.0267 | 0.0000 | 0.1143 | 0.0000 | 0.0090 | 0.0734 | 0.2574 | 0.0415 | 0.3166 | 0.0542 | 0.0000 | 0.0435 | 0.0000 | 0.0000 | 0.0000 |
| MB-0514 | 0.0277 | 0.0000 | 0.0297 | 0.1396 | 0.0000 | 0.0707 | 0.0000 | 0.0838 | 0.0000 | 0.0160 | 0.0000 | 0.0593 | 0.0013 | 0.0000 | 0.0435 | 0.2038 | 0.0000 | 0.0000 | 0.3225 | 0.0000 | 0.0000 | 0.0020 |
| MB-0395 | 0.0298 | 0.0000 | 0.0110 | 0.0193 | 0.0000 | 0.1265 | 0.0000 | 0.0965 | 0.0000 | 0.1324 | 0.0000 | 0.0517 | 0.0469 | 0.0637 | 0.0931 | 0.2404 | 0.0000 | 0.0429 | 0.0460 | 0.0000 | 0.0000 | 0.0000 |
| MB-0294 | 0.0000 | 0.0000 | 0.1633 | 0.0724 | 0.0000 | 0.0616 | 0.0003 | 0.0724 | 0.0283 | 0.0000 | 0.0000 | 0.0416 | 0.0318 | 0.0532 | 0.1278 | 0.0907 | 0.0131 | 0.0000 | 0.2436 | 0.0000 | 0.0000 | 0.0000 |
| MB-0439 | 0.0000 | 0.0260 | 0.0000 | 0.0000 | 0.0181 | 0.0000 | 0.0000 | 0.0498 | 0.0394 | 0.0000 | 0.0286 | 0.0099 | 0.0108 | 0.4234 | 0.0251 | 0.3301 | 0.0000 | 0.0000 | 0.0177 | 0.0210 | 0.0000 | 0.0000 |
| MB-0481 | 0.0037 | 0.0000 | 0.0000 | 0.1268 | 0.0000 | 0.0248 | 0.0000 | 0.1440 | 0.0089 | 0.0870 | 0.0000 | 0.0953 | 0.0125 | 0.0947 | 0.1464 | 0.1889 | 0.0478 | 0.0000 | 0.0000 | 0.0194 | 0.0000 | 0.0000 |
| MB-0529 | 0.0029 | 0.0000 | 0.0425 | 0.1551 | 0.0000 | 0.0118 | 0.0000 | 0.0218 | 0.0000 | 0.0077 | 0.0000 | 0.0552 | 0.0000 | 0.0000 | 0.0454 | 0.2843 | 0.0073 | 0.0000 | 0.3660 | 0.0000 | 0.0000 | 0.0000 |
| MB-0224 | 0.0050 | 0.0000 | 0.0677 | 0.0826 | 0.0000 | 0.2197 | 0.0000 | 0.0037 | 0.0200 | 0.0000 | 0.0000 | 0.0246 | 0.0426 | 0.0528 | 0.0602 | 0.2476 | 0.0235 | 0.0000 | 0.1336 | 0.0165 | 0.0000 | 0.0000 |
| MB-0302 | 0.0475 | 0.0491 | 0.0000 | 0.0510 | 0.0000 | 0.1006 | 0.0000 | 0.0889 | 0.0164 | 0.0000 | 0.0091 | 0.0209 | 0.0394 | 0.0386 | 0.0971 | 0.1918 | 0.0000 | 0.0000 | 0.2496 | 0.0000 | 0.0000 | 0.0000 |
| MB-0126 | 0.0096 | 0.0307 | 0.0000 | 0.0491 | 0.0000 | 0.0000 | 0.0000 | 0.0232 | 0.0475 | 0.0169 | 0.0355 | 0.0000 | 0.0000 | 0.2283 | 0.0308 | 0.3209 | 0.0047 | 0.0000 | 0.1922 | 0.0105 | 0.0000 | 0.0000 |
| MB-0220 | 0.0342 | 0.0000 | 0.0018 | 0.1020 | 0.0000 | 0.3036 | 0.0552 | 0.0161 | 0.0000 | 0.0000 | 0.0006 | 0.0163 | 0.0854 | 0.1707 | 0.0638 | 0.0741 | 0.0000 | 0.0000 | 0.0763 | 0.0000 | 0.0000 | 0.0000 |
| MB-0192 | 0.0739 | 0.0229 | 0.0000 | 0.1154 | 0.0032 | 0.1150 | 0.0182 | 0.0663 | 0.0000 | 0.1139 | 0.0000 | 0.0636 | 0.0109 | 0.1239 | 0.1174 | 0.0907 | 0.0000 | 0.0000 | 0.0648 | 0.0000 | 0.0000 | 0.0000 |
| MB-0121 | 0.0000 | 0.0166 | 0.0010 | 0.0381 | 0.0000 | 0.1124 | 0.0000 | 0.0039 | 0.0000 | 0.0298 | 0.0079 | 0.0040 | 0.1342 | 0.0177 | 0.0152 | 0.5037 | 0.0910 | 0.0000 | 0.0157 | 0.0000 | 0.0000 | 0.0088 |
| MB-0239 | 0.0084 | 0.0070 | 0.0000 | 0.0344 | 0.0000 | 0.0408 | 0.0112 | 0.0152 | 0.0000 | 0.1202 | 0.0000 | 0.0519 | 0.0679 | 0.0302 | 0.0333 | 0.3101 | 0.0139 | 0.0000 | 0.2555 | 0.0000 | 0.0000 | 0.0000 |
| MB-0364 | 0.0001 | 0.0000 | 0.0149 | 0.0201 | 0.0000 | 0.0811 | 0.0000 | 0.0215 | 0.0000 | 0.0000 | 0.0000 | 0.0628 | 0.0702 | 0.0000 | 0.0269 | 0.2184 | 0.0022 | 0.0071 | 0.4615 | 0.0131 | 0.0000 | 0.0000 |
| MB-0232 | 0.0273 | 0.0000 | 0.0000 | 0.0392 | 0.0000 | 0.1095 | 0.0000 | 0.0957 | 0.0058 | 0.0000 | 0.0000 | 0.0338 | 0.0423 | 0.2522 | 0.0351 | 0.1975 | 0.0084 | 0.0000 | 0.0885 | 0.0648 | 0.0000 | 0.0000 |
| MB-0884 | 0.0565 | 0.0000 | 0.0151 | 0.0281 | 0.0000 | 0.1361 | 0.0234 | 0.0861 | 0.0000 | 0.0859 | 0.0000 | 0.0285 | 0.0147 | 0.1459 | 0.1110 | 0.1625 | 0.0178 | 0.0000 | 0.0883 | 0.0000 | 0.0000 | 0.0000 |
| MB-0238 | 0.0301 | 0.0000 | 0.0774 | 0.0345 | 0.0396 | 0.0000 | 0.0092 | 0.1209 | 0.0048 | 0.0730 | 0.0034 | 0.0208 | 0.0564 | 0.1057 | 0.2010 | 0.1296 | 0.0042 | 0.0000 | 0.0895 | 0.0000 | 0.0000 | 0.0000 |
| MB-0194 | 0.0086 | 0.0000 | 0.0066 | 0.0000 | 0.0000 | 0.1545 | 0.0000 | 0.0322 | 0.0000 | 0.0454 | 0.0000 | 0.0074 | 0.0109 | 0.0611 | 0.1062 | 0.1189 | 0.0678 | 0.0000 | 0.3193 | 0.0610 | 0.0000 | 0.0000 |
| MB-0882 | 0.0324 | 0.0000 | 0.0000 | 0.0186 | 0.0000 | 0.1366 | 0.0000 | 0.0208 | 0.0529 | 0.0000 | 0.0000 | 0.0534 | 0.0000 | 0.1859 | 0.0072 | 0.2306 | 0.0000 | 0.0060 | 0.2447 | 0.0028 | 0.0000 | 0.0081 |
| MB-0010 | 0.0109 | 0.0000 | 0.0022 | 0.0537 | 0.0000 | 0.0012 | 0.0000 | 0.0585 | 0.0093 | 0.0654 | 0.0000 | 0.0349 | 0.0000 | 0.2834 | 0.0356 | 0.4248 | 0.0000 | 0.0000 | 0.0113 | 0.0036 | 0.0000 | 0.0051 |
| MB-0236 | 0.0034 | 0.0000 | 0.0345 | 0.0414 | 0.0000 | 0.0801 | 0.0000 | 0.0469 | 0.0000 | 0.0477 | 0.0000 | 0.0554 | 0.0823 | 0.0779 | 0.0901 | 0.0995 | 0.0356 | 0.0000 | 0.3053 | 0.0000 | 0.0000 | 0.0000 |
| MB-0377 | 0.0127 | 0.0000 | 0.0219 | 0.0699 | 0.0000 | 0.1714 | 0.0000 | 0.0485 | 0.0000 | 0.0719 | 0.0000 | 0.0608 | 0.2216 | 0.0267 | 0.0451 | 0.0882 | 0.0000 | 0.0000 | 0.1011 | 0.0603 | 0.0000 | 0.0000 |
| MB-0123 | 0.0000 | 0.0111 | 0.0091 | 0.0311 | 0.0000 | 0.2178 | 0.0000 | 0.0103 | 0.0000 | 0.0000 | 0.0000 | 0.0266 | 0.0605 | 0.0233 | 0.0663 | 0.1723 | 0.0270 | 0.0000 | 0.3288 | 0.0158 | 0.0000 | 0.0000 |
| MB-0504 | 0.0000 | 0.0032 | 0.0522 | 0.0300 | 0.0000 | 0.1076 | 0.0000 | 0.0338 | 0.0087 | 0.0000 | 0.0000 | 0.0718 | 0.0560 | 0.0000 | 0.0282 | 0.1590 | 0.0017 | 0.0026 | 0.4451 | 0.0000 | 0.0000 | 0.0000 |
| MB-0475 | 0.0029 | 0.0000 | 0.0035 | 0.0556 | 0.0000 | 0.0687 | 0.0059 | 0.0702 | 0.0000 | 0.0000 | 0.0000 | 0.1122 | 0.0355 | 0.1225 | 0.1529 | 0.1956 | 0.0083 | 0.0000 | 0.1664 | 0.0000 | 0.0000 | 0.0000 |
| MB-0321 | 0.0495 | 0.0000 | 0.0095 | 0.0056 | 0.0000 | 0.0000 | 0.0000 | 0.1104 | 0.0136 | 0.0511 | 0.0000 | 0.0549 | 0.0646 | 0.2165 | 0.0188 | 0.1408 | 0.0177 | 0.0000 | 0.0000 | 0.2350 | 0.0120 | 0.0000 |
| MB-0482 | 0.0000 | 0.0000 | 0.0185 | 0.0257 | 0.0000 | 0.0620 | 0.0000 | 0.0675 | 0.0464 | 0.0000 | 0.0000 | 0.0468 | 0.0917 | 0.1765 | 0.2144 | 0.1088 | 0.0239 | 0.0000 | 0.1178 | 0.0000 | 0.0000 | 0.0000 |
| MB-0101 | 0.0058 | 0.0000 | 0.0155 | 0.0290 | 0.0000 | 0.0607 | 0.0000 | 0.1027 | 0.0127 | 0.0883 | 0.0000 | 0.0858 | 0.0287 | 0.1672 | 0.0739 | 0.1867 | 0.0157 | 0.0000 | 0.1272 | 0.0000 | 0.0000 | 0.0000 |
| MB-0662 | 0.0108 | 0.0128 | 0.0104 | 0.0637 | 0.0000 | 0.0451 | 0.0002 | 0.1001 | 0.0395 | 0.0163 | 0.0000 | 0.0628 | 0.0437 | 0.1859 | 0.2399 | 0.0797 | 0.0000 | 0.0000 | 0.0891 | 0.0000 | 0.0000 | 0.0000 |
| MB-0291 | 0.0148 | 0.0000 | 0.0232 | 0.0157 | 0.0000 | 0.0733 | 0.0000 | 0.0260 | 0.0032 | 0.0094 | 0.0000 | 0.0197 | 0.0191 | 0.4709 | 0.0905 | 0.1060 | 0.0066 | 0.0000 | 0.1216 | 0.0000 | 0.0000 | 0.0000 |
| MB-0465 | 0.0076 | 0.0000 | 0.0186 | 0.1552 | 0.0000 | 0.0000 | 0.0000 | 0.0790 | 0.0000 | 0.0563 | 0.0000 | 0.0866 | 0.0839 | 0.0394 | 0.1190 | 0.2445 | 0.0000 | 0.0000 | 0.0389 | 0.0709 | 0.0000 | 0.0000 |
| MB-0630 | 0.0000 | 0.0000 | 0.0132 | 0.0000 | 0.0000 | 0.0849 | 0.0000 | 0.0411 | 0.0000 | 0.0000 | 0.0000 | 0.0306 | 0.0000 | 0.2921 | 0.0350 | 0.2630 | 0.0000 | 0.0000 | 0.1376 | 0.1025 | 0.0000 | 0.0000 |
| MB-0036 | 0.0325 | 0.0000 | 0.0048 | 0.0419 | 0.0000 | 0.0571 | 0.0000 | 0.0678 | 0.0013 | 0.0059 | 0.0000 | 0.0783 | 0.0019 | 0.0000 | 0.0304 | 0.4244 | 0.1396 | 0.0000 | 0.0802 | 0.0339 | 0.0000 | 0.0000 |
| MB-0166 | 0.0000 | 0.0000 | 0.0494 | 0.0804 | 0.0000 | 0.0735 | 0.0000 | 0.0617 | 0.0176 | 0.0120 | 0.0000 | 0.0342 | 0.0472 | 0.1574 | 0.1053 | 0.1841 | 0.0254 | 0.0000 | 0.1518 | 0.0000 | 0.0000 | 0.0000 |
| MB-0180 | 0.0000 | 0.0190 | 0.0061 | 0.0173 | 0.0000 | 0.0000 | 0.0000 | 0.0781 | 0.0095 | 0.0000 | 0.0000 | 0.0188 | 0.0000 | 0.1676 | 0.0201 | 0.0849 | 0.0000 | 0.0000 | 0.5745 | 0.0042 | 0.0000 | 0.0000 |
| MB-0525 | 0.0000 | 0.0000 | 0.0273 | 0.0000 | 0.0000 | 0.0620 | 0.0000 | 0.0976 | 0.0000 | 0.0000 | 0.0154 | 0.0145 | 0.0000 | 0.3253 | 0.0319 | 0.2128 | 0.0057 | 0.0000 | 0.1411 | 0.0663 | 0.0000 | 0.0000 |
| MB-0307 | 0.0344 | 0.0000 | 0.1775 | 0.0848 | 0.0000 | 0.0579 | 0.0000 | 0.0938 | 0.0000 | 0.0543 | 0.0000 | 0.0682 | 0.0584 | 0.0478 | 0.1098 | 0.0640 | 0.0049 | 0.0000 | 0.1113 | 0.0330 | 0.0000 | 0.0000 |
| MB-0002 | 0.0203 | 0.0000 | 0.0146 | 0.0415 | 0.0000 | 0.0413 | 0.0590 | 0.0990 | 0.0000 | 0.0149 | 0.0000 | 0.0624 | 0.0379 | 0.0736 | 0.0743 | 0.0644 | 0.0183 | 0.0000 | 0.3786 | 0.0000 | 0.0000 | 0.0000 |
| MB-0466 | 0.0057 | 0.0000 | 0.0217 | 0.0631 | 0.0000 | 0.1353 | 0.0033 | 0.0115 | 0.0000 | 0.0000 | 0.0000 | 0.0410 | 0.0326 | 0.0236 | 0.0354 | 0.1151 | 0.0399 | 0.0000 | 0.4720 | 0.0000 | 0.0000 | 0.0000 |
| MB-0632 | 0.0336 | 0.0000 | 0.0184 | 0.0045 | 0.0000 | 0.0704 | 0.0000 | 0.0110 | 0.0327 | 0.0000 | 0.0000 | 0.0167 | 0.0000 | 0.2604 | 0.0251 | 0.1280 | 0.0000 | 0.0000 | 0.3991 | 0.0000 | 0.0000 | 0.0000 |
| MB-0120 | 0.0405 | 0.0000 | 0.1753 | 0.0489 | 0.0000 | 0.0752 | 0.0419 | 0.0551 | 0.0049 | 0.0031 | 0.0000 | 0.0692 | 0.0211 | 0.0434 | 0.1427 | 0.1440 | 0.0074 | 0.0000 | 0.1270 | 0.0000 | 0.0000 | 0.0000 |
| MB-0507 | 0.0339 | 0.0228 | 0.0000 | 0.0040 | 0.0000 | 0.1022 | 0.0217 | 0.0451 | 0.0000 | 0.1233 | 0.0000 | 0.0237 | 0.0132 | 0.1140 | 0.0876 | 0.1539 | 0.0000 | 0.0000 | 0.2400 | 0.0146 | 0.0000 | 0.0000 |
| MB-0287 | 0.0159 | 0.0000 | 0.0000 | 0.0333 | 0.0000 | 0.1868 | 0.0000 | 0.0521 | 0.0337 | 0.0305 | 0.0000 | 0.0407 | 0.0094 | 0.0957 | 0.0840 | 0.2351 | 0.0000 | 0.0000 | 0.1759 | 0.0000 | 0.0000 | 0.0069 |
| MB-0869 | 0.0174 | 0.0000 | 0.0000 | 0.0438 | 0.0000 | 0.0055 | 0.0774 | 0.1227 | 0.0000 | 0.0725 | 0.0000 | 0.0369 | 0.0000 | 0.3074 | 0.1469 | 0.1152 | 0.0191 | 0.0000 | 0.0000 | 0.0249 | 0.0000 | 0.0105 |
| MB-4633 | 0.0313 | 0.0000 | 0.0000 | 0.0626 | 0.0000 | 0.0980 | 0.0000 | 0.0378 | 0.0028 | 0.0389 | 0.0000 | 0.0438 | 0.0001 | 0.3037 | 0.0527 | 0.1891 | 0.0000 | 0.0000 | 0.1392 | 0.0000 | 0.0000 | 0.0000 |
| MB-4627 | 0.0737 | 0.0000 | 0.0496 | 0.0557 | 0.0000 | 0.1232 | 0.0000 | 0.0352 | 0.0213 | 0.0023 | 0.0000 | 0.0442 | 0.0260 | 0.0701 | 0.1518 | 0.1796 | 0.0155 | 0.0000 | 0.1517 | 0.0000 | 0.0000 | 0.0000 |
| MB-4004 | 0.0000 | 0.0145 | 0.0327 | 0.0000 | 0.0475 | 0.1145 | 0.0334 | 0.1299 | 0.0000 | 0.0389 | 0.0000 | 0.0189 | 0.0275 | 0.1890 | 0.1435 | 0.1577 | 0.0000 | 0.0000 | 0.0521 | 0.0000 | 0.0000 | 0.0000 |
| MB-4708 | 0.0000 | 0.0000 | 0.0435 | 0.1906 | 0.0000 | 0.0666 | 0.0000 | 0.0279 | 0.0154 | 0.0000 | 0.0000 | 0.0961 | 0.0475 | 0.0000 | 0.0987 | 0.1794 | 0.0158 | 0.0000 | 0.2186 | 0.0000 | 0.0000 | 0.0000 |
| MB-4618 | 0.0106 | 0.0000 | 0.0542 | 0.0000 | 0.0000 | 0.0811 | 0.0156 | 0.0868 | 0.0618 | 0.0308 | 0.0497 | 0.0015 | 0.0000 | 0.2527 | 0.1366 | 0.1488 | 0.0000 | 0.0000 | 0.0699 | 0.0000 | 0.0000 | 0.0000 |
| MB-4641 | 0.0059 | 0.0222 | 0.0000 | 0.0331 | 0.0683 | 0.0000 | 0.0000 | 0.0000 | 0.0466 | 0.0255 | 0.0000 | 0.0242 | 0.0164 | 0.3267 | 0.0418 | 0.1291 | 0.0000 | 0.0000 | 0.2125 | 0.0477 | 0.0000 | 0.0000 |
| MB-4622 | 0.0170 | 0.0000 | 0.0519 | 0.0000 | 0.0000 | 0.0300 | 0.0336 | 0.0373 | 0.0201 | 0.1816 | 0.0000 | 0.0614 | 0.0174 | 0.3515 | 0.1110 | 0.0527 | 0.0000 | 0.0000 | 0.0344 | 0.0000 | 0.0000 | 0.0000 |
| MB-4634 | 0.0053 | 0.0184 | 0.0000 | 0.0000 | 0.1158 | 0.0000 | 0.0000 | 0.0245 | 0.1000 | 0.0386 | 0.0000 | 0.0793 | 0.0000 | 0.4540 | 0.0278 | 0.1153 | 0.0000 | 0.0000 | 0.0014 | 0.0196 | 0.0000 | 0.0000 |
| MB-4688 | 0.0144 | 0.0000 | 0.0000 | 0.0858 | 0.0000 | 0.0355 | 0.0000 | 0.0983 | 0.0000 | 0.0911 | 0.0000 | 0.0476 | 0.0000 | 0.3069 | 0.1640 | 0.0521 | 0.0000 | 0.0000 | 0.1043 | 0.0000 | 0.0000 | 0.0000 |
| MB-4665 | 0.0000 | 0.0097 | 0.0762 | 0.0655 | 0.0000 | 0.0000 | 0.0000 | 0.1106 | 0.0408 | 0.0000 | 0.0023 | 0.0401 | 0.0025 | 0.2477 | 0.0108 | 0.2493 | 0.0000 | 0.0192 | 0.1252 | 0.0000 | 0.0000 | 0.0000 |
| MB-4666 | 0.0494 | 0.0000 | 0.0239 | 0.0446 | 0.0000 | 0.0233 | 0.0000 | 0.0521 | 0.0428 | 0.0000 | 0.0000 | 0.0200 | 0.0000 | 0.4383 | 0.0703 | 0.1369 | 0.0000 | 0.0000 | 0.0984 | 0.0000 | 0.0000 | 0.0000 |
| MB-4640 | 0.0000 | 0.0000 | 0.0109 | 0.1378 | 0.0000 | 0.0317 | 0.0000 | 0.0515 | 0.0418 | 0.0385 | 0.0000 | 0.0310 | 0.0000 | 0.3119 | 0.1294 | 0.1207 | 0.0032 | 0.0000 | 0.0914 | 0.0000 | 0.0000 | 0.0000 |
| MB-4691 | 0.0555 | 0.0000 | 0.0060 | 0.1504 | 0.0000 | 0.0000 | 0.0000 | 0.1066 | 0.0035 | 0.0000 | 0.0000 | 0.0250 | 0.0000 | 0.0472 | 0.0675 | 0.4441 | 0.0124 | 0.0000 | 0.0816 | 0.0000 | 0.0000 | 0.0000 |
| MB-4671 | 0.0434 | 0.0000 | 0.0348 | 0.0000 | 0.0998 | 0.0089 | 0.0000 | 0.0572 | 0.0216 | 0.0877 | 0.0000 | 0.0552 | 0.0000 | 0.3471 | 0.0656 | 0.0650 | 0.0000 | 0.0000 | 0.0945 | 0.0004 | 0.0000 | 0.0187 |
| MB-4667 | 0.0132 | 0.0000 | 0.0536 | 0.0445 | 0.0788 | 0.0350 | 0.0000 | 0.0288 | 0.0286 | 0.2018 | 0.0000 | 0.1201 | 0.0147 | 0.1600 | 0.1004 | 0.0347 | 0.0000 | 0.0000 | 0.0857 | 0.0000 | 0.0000 | 0.0000 |
| MB-4721 | 0.0000 | 0.0000 | 0.0140 | 0.1956 | 0.0000 | 0.0579 | 0.0000 | 0.0277 | 0.0000 | 0.0010 | 0.0000 | 0.0040 | 0.0089 | 0.0000 | 0.1715 | 0.2898 | 0.0223 | 0.0000 | 0.2075 | 0.0000 | 0.0000 | 0.0000 |
| MB-4723 | 0.0149 | 0.0049 | 0.0000 | 0.0067 | 0.0716 | 0.0000 | 0.0000 | 0.0208 | 0.0204 | 0.0902 | 0.0000 | 0.0301 | 0.0000 | 0.3159 | 0.0485 | 0.1889 | 0.0000 | 0.0000 | 0.1602 | 0.0270 | 0.0000 | 0.0000 |
| MB-4718 | 0.0000 | 0.0056 | 0.0609 | 0.0379 | 0.0000 | 0.2825 | 0.0000 | 0.0314 | 0.0000 | 0.0149 | 0.0010 | 0.0439 | 0.0284 | 0.0000 | 0.0656 | 0.2072 | 0.0000 | 0.0000 | 0.2208 | 0.0000 | 0.0000 | 0.0000 |
| MB-4716 | 0.0271 | 0.0044 | 0.0000 | 0.0179 | 0.0310 | 0.1129 | 0.0000 | 0.0703 | 0.0603 | 0.0731 | 0.0000 | 0.0636 | 0.0274 | 0.1531 | 0.1680 | 0.1092 | 0.0000 | 0.0000 | 0.0818 | 0.0000 | 0.0000 | 0.0000 |
| MB-4729 | 0.0793 | 0.0000 | 0.0000 | 0.0631 | 0.1807 | 0.0000 | 0.0000 | 0.0000 | 0.1094 | 0.0527 | 0.0000 | 0.0903 | 0.0000 | 0.3130 | 0.0638 | 0.0291 | 0.0000 | 0.0013 | 0.0136 | 0.0038 | 0.0000 | 0.0000 |
| MB-4717 | 0.0000 | 0.0121 | 0.0184 | 0.0000 | 0.0000 | 0.0000 | 0.0000 | 0.0857 | 0.0517 | 0.0158 | 0.0000 | 0.0271 | 0.0398 | 0.4344 | 0.0313 | 0.1604 | 0.0000 | 0.0158 | 0.0000 | 0.1074 | 0.0000 | 0.0000 |
| MB-4724 | 0.0000 | 0.0004 | 0.0488 | 0.0621 | 0.0000 | 0.0936 | 0.0153 | 0.0558 | 0.0007 | 0.0608 | 0.0000 | 0.0784 | 0.0064 | 0.1583 | 0.1322 | 0.1795 | 0.0360 | 0.0000 | 0.0717 | 0.0000 | 0.0000 | 0.0000 |
| MB-4770 | 0.0075 | 0.0000 | 0.0408 | 0.0332 | 0.0090 | 0.0216 | 0.0000 | 0.0547 | 0.0136 | 0.0305 | 0.0000 | 0.0851 | 0.0250 | 0.2803 | 0.1615 | 0.1527 | 0.0000 | 0.0000 | 0.0844 | 0.0000 | 0.0000 | 0.0000 |
| MB-4752 | 0.0000 | 0.0013 | 0.0318 | 0.0913 | 0.0000 | 0.1918 | 0.0000 | 0.0776 | 0.0156 | 0.0137 | 0.0000 | 0.0199 | 0.0000 | 0.1531 | 0.0989 | 0.2784 | 0.0000 | 0.0000 | 0.0199 | 0.0000 | 0.0000 | 0.0067 |
| MB-4762 | 0.0086 | 0.0000 | 0.0139 | 0.0000 | 0.0000 | 0.1107 | 0.0000 | 0.0044 | 0.0150 | 0.0000 | 0.0091 | 0.0178 | 0.0000 | 0.2039 | 0.0163 | 0.0921 | 0.0000 | 0.0000 | 0.5039 | 0.0024 | 0.0000 | 0.0019 |
| MB-5169 | 0.0477 | 0.0000 | 0.0019 | 0.0245 | 0.0000 | 0.0358 | 0.0000 | 0.0860 | 0.0028 | 0.0173 | 0.0000 | 0.0923 | 0.0000 | 0.2483 | 0.0662 | 0.2060 | 0.0000 | 0.0000 | 0.1046 | 0.0648 | 0.0000 | 0.0020 |
| MB-4796 | 0.0000 | 0.0179 | 0.0000 | 0.0671 | 0.0000 | 0.1131 | 0.0000 | 0.0000 | 0.0000 | 0.0472 | 0.0000 | 0.0500 | 0.0000 | 0.2000 | 0.0636 | 0.1606 | 0.0102 | 0.0000 | 0.2610 | 0.0093 | 0.0000 | 0.0000 |
| MB-4790 | 0.0287 | 0.0000 | 0.1048 | 0.0389 | 0.0000 | 0.1626 | 0.0000 | 0.0000 | 0.0049 | 0.0000 | 0.0379 | 0.0000 | 0.0119 | 0.1505 | 0.0999 | 0.2136 | 0.0008 | 0.0000 | 0.1454 | 0.0000 | 0.0000 | 0.0000 |
| MB-4797 | 0.0292 | 0.0277 | 0.0000 | 0.0144 | 0.0591 | 0.0005 | 0.0000 | 0.0758 | 0.0000 | 0.0825 | 0.0000 | 0.0000 | 0.0764 | 0.0640 | 0.1130 | 0.1729 | 0.0140 | 0.0000 | 0.2675 | 0.0000 | 0.0029 | 0.0000 |
| MB-4800 | 0.0764 | 0.0000 | 0.0000 | 0.0000 | 0.0000 | 0.0000 | 0.0000 | 0.0368 | 0.0608 | 0.0351 | 0.0688 | 0.0000 | 0.0009 | 0.5992 | 0.0110 | 0.1025 | 0.0000 | 0.0000 | 0.0038 | 0.0045 | 0.0000 | 0.0000 |
| MB-5541 | 0.0349 | 0.0000 | 0.0479 | 0.0000 | 0.0988 | 0.2978 | 0.0000 | 0.0518 | 0.0000 | 0.0000 | 0.0371 | 0.0188 | 0.0416 | 0.1664 | 0.1075 | 0.0463 | 0.0046 | 0.0040 | 0.0334 | 0.0091 | 0.0000 | 0.0000 |
| MB-5549 | 0.0089 | 0.0000 | 0.1344 | 0.0204 | 0.0240 | 0.0358 | 0.0000 | 0.0902 | 0.0372 | 0.0769 | 0.0000 | 0.0666 | 0.0000 | 0.1964 | 0.1292 | 0.1450 | 0.0262 | 0.0000 | 0.0088 | 0.0000 | 0.0000 | 0.0000 |
| MB-5519 | 0.0000 | 0.0020 | 0.0456 | 0.1397 | 0.0114 | 0.1520 | 0.0000 | 0.0877 | 0.0168 | 0.0000 | 0.0000 | 0.0323 | 0.0302 | 0.2605 | 0.0968 | 0.0879 | 0.0080 | 0.0000 | 0.0292 | 0.0000 | 0.0000 | 0.0000 |
| MB-5495 | 0.0674 | 0.0000 | 0.0438 | 0.1062 | 0.0000 | 0.0409 | 0.0000 | 0.0950 | 0.0217 | 0.0000 | 0.0000 | 0.0457 | 0.0387 | 0.2338 | 0.0565 | 0.1221 | 0.0000 | 0.0000 | 0.1283 | 0.0000 | 0.0000 | 0.0000 |
| MB-4832 | 0.0773 | 0.0000 | 0.0114 | 0.0614 | 0.0000 | 0.0000 | 0.0000 | 0.0784 | 0.0066 | 0.0000 | 0.0012 | 0.0000 | 0.0000 | 0.4068 | 0.0384 | 0.2326 | 0.0000 | 0.0000 | 0.0860 | 0.0000 | 0.0000 | 0.0000 |
| MB-4745 | 0.0069 | 0.0000 | 0.0222 | 0.1561 | 0.0000 | 0.0997 | 0.0061 | 0.0508 | 0.0099 | 0.0000 | 0.0000 | 0.0853 | 0.0000 | 0.1642 | 0.2164 | 0.0813 | 0.0100 | 0.0069 | 0.0842 | 0.0000 | 0.0000 | 0.0000 |
| MB-4825 | 0.0000 | 0.0451 | 0.0000 | 0.0235 | 0.0550 | 0.0000 | 0.0000 | 0.0308 | 0.0667 | 0.0452 | 0.0226 | 0.0000 | 0.0000 | 0.4059 | 0.0365 | 0.1811 | 0.0000 | 0.0000 | 0.0000 | 0.0877 | 0.0000 | 0.0000 |
| MB-4814 | 0.0748 | 0.0000 | 0.0516 | 0.0247 | 0.0000 | 0.0428 | 0.0000 | 0.1449 | 0.0000 | 0.0000 | 0.0540 | 0.0159 | 0.0000 | 0.3242 | 0.1247 | 0.1025 | 0.0000 | 0.0000 | 0.0397 | 0.0000 | 0.0000 | 0.0000 |
| MB-4757 | 0.0087 | 0.0000 | 0.0290 | 0.0234 | 0.0383 | 0.0782 | 0.0000 | 0.1064 | 0.0092 | 0.0292 | 0.0000 | 0.0692 | 0.0209 | 0.1244 | 0.1943 | 0.1959 | 0.0020 | 0.0000 | 0.0707 | 0.0000 | 0.0000 | 0.0000 |
| MB-4694 | 0.0222 | 0.0000 | 0.0201 | 0.0080 | 0.0000 | 0.0173 | 0.0000 | 0.1419 | 0.0325 | 0.0872 | 0.0000 | 0.0916 | 0.0160 | 0.2277 | 0.2480 | 0.0000 | 0.0000 | 0.0000 | 0.0875 | 0.0000 | 0.0000 | 0.0000 |
| MB-4698 | 0.0000 | 0.0117 | 0.0362 | 0.2441 | 0.0000 | 0.0900 | 0.0000 | 0.0835 | 0.0098 | 0.0000 | 0.0000 | 0.0664 | 0.0231 | 0.0648 | 0.1130 | 0.1301 | 0.0005 | 0.0000 | 0.1268 | 0.0000 | 0.0000 | 0.0000 |
| MB-4715 | 0.0023 | 0.0000 | 0.0245 | 0.0817 | 0.0000 | 0.0000 | 0.0186 | 0.1273 | 0.0246 | 0.0329 | 0.0000 | 0.0469 | 0.0000 | 0.2969 | 0.1827 | 0.1380 | 0.0133 | 0.0000 | 0.0000 | 0.0102 | 0.0000 | 0.0000 |
| MB-4685 | 0.1135 | 0.0000 | 0.0143 | 0.0000 | 0.1183 | 0.0000 | 0.0000 | 0.0617 | 0.0033 | 0.0696 | 0.0000 | 0.0415 | 0.0000 | 0.2085 | 0.0139 | 0.1938 | 0.0007 | 0.0000 | 0.1440 | 0.0169 | 0.0000 | 0.0000 |
| MB-4712 | 0.0647 | 0.0000 | 0.0000 | 0.0522 | 0.0000 | 0.0534 | 0.0000 | 0.1505 | 0.0221 | 0.0000 | 0.0223 | 0.0000 | 0.0131 | 0.2434 | 0.1864 | 0.1243 | 0.0000 | 0.0000 | 0.0676 | 0.0000 | 0.0000 | 0.0000 |
| MB-4672 | 0.0508 | 0.0000 | 0.0208 | 0.0004 | 0.0173 | 0.0773 | 0.0000 | 0.0000 | 0.0255 | 0.0000 | 0.0000 | 0.0917 | 0.0000 | 0.0857 | 0.0365 | 0.1927 | 0.0329 | 0.0000 | 0.3683 | 0.0000 | 0.0000 | 0.0000 |
| MB-4704 | 0.0091 | 0.0000 | 0.0426 | 0.0738 | 0.0782 | 0.0734 | 0.0000 | 0.0595 | 0.0194 | 0.0388 | 0.0000 | 0.0291 | 0.0476 | 0.0952 | 0.1196 | 0.2347 | 0.0000 | 0.0000 | 0.0790 | 0.0000 | 0.0000 | 0.0000 |
| MB-4655 | 0.0000 | 0.0000 | 0.0789 | 0.1441 | 0.0265 | 0.0000 | 0.0000 | 0.0909 | 0.0189 | 0.0707 | 0.0000 | 0.0901 | 0.0158 | 0.2056 | 0.0929 | 0.0628 | 0.0000 | 0.0028 | 0.1000 | 0.0000 | 0.0000 | 0.0000 |
| MB-4651 | 0.0000 | 0.0000 | 0.0247 | 0.0717 | 0.0000 | 0.1431 | 0.0000 | 0.0616 | 0.0092 | 0.0503 | 0.0000 | 0.0615 | 0.0100 | 0.0950 | 0.0815 | 0.1503 | 0.0008 | 0.0000 | 0.2403 | 0.0000 | 0.0000 | 0.0000 |
| MB-4661 | 0.0556 | 0.0000 | 0.0090 | 0.0704 | 0.0000 | 0.0830 | 0.0000 | 0.1010 | 0.0118 | 0.0251 | 0.0000 | 0.0843 | 0.0000 | 0.0619 | 0.1490 | 0.1690 | 0.0318 | 0.0000 | 0.1481 | 0.0000 | 0.0000 | 0.0000 |
| MB-4649 | 0.0203 | 0.0000 | 0.1225 | 0.0582 | 0.0000 | 0.0000 | 0.0000 | 0.0674 | 0.0618 | 0.0894 | 0.0000 | 0.0045 | 0.0000 | 0.3170 | 0.1383 | 0.0715 | 0.0000 | 0.0000 | 0.0488 | 0.0000 | 0.0000 | 0.0000 |
| MB-4642 | 0.0896 | 0.0000 | 0.0000 | 0.0208 | 0.0000 | 0.2214 | 0.0000 | 0.0000 | 0.0269 | 0.0000 | 0.0550 | 0.0000 | 0.0000 | 0.3335 | 0.0476 | 0.1724 | 0.0000 | 0.0000 | 0.0000 | 0.0329 | 0.0000 | 0.0000 |
| MB-4674 | 0.0279 | 0.0000 | 0.0000 | 0.0000 | 0.0453 | 0.0000 | 0.0000 | 0.0207 | 0.0426 | 0.0000 | 0.1411 | 0.0000 | 0.0046 | 0.1874 | 0.0156 | 0.1940 | 0.0536 | 0.0000 | 0.2324 | 0.0000 | 0.0348 | 0.0000 |
| MB-4648 | 0.0549 | 0.0000 | 0.0116 | 0.0432 | 0.0000 | 0.0237 | 0.0000 | 0.0858 | 0.0153 | 0.0000 | 0.0000 | 0.0514 | 0.0000 | 0.1163 | 0.0265 | 0.4091 | 0.0000 | 0.0000 | 0.0000 | 0.1614 | 0.0000 | 0.0008 |
| MB-5197 | 0.0352 | 0.0000 | 0.0167 | 0.0022 | 0.0000 | 0.0444 | 0.0000 | 0.0408 | 0.0066 | 0.0206 | 0.0000 | 0.0098 | 0.0315 | 0.3206 | 0.0446 | 0.2169 | 0.0008 | 0.0000 | 0.2093 | 0.0000 | 0.0000 | 0.0000 |
| MB-5152 | 0.0299 | 0.0000 | 0.0000 | 0.1447 | 0.0000 | 0.0000 | 0.0000 | 0.0552 | 0.0000 | 0.0000 | 0.0000 | 0.0581 | 0.0149 | 0.0000 | 0.0768 | 0.5409 | 0.0265 | 0.0000 | 0.0492 | 0.0000 | 0.0000 | 0.0037 |
| MB-5201 | 0.0708 | 0.0000 | 0.0219 | 0.0000 | 0.0028 | 0.0770 | 0.0000 | 0.0663 | 0.0204 | 0.0000 | 0.0048 | 0.0389 | 0.0000 | 0.2874 | 0.0489 | 0.2791 | 0.0123 | 0.0000 | 0.0635 | 0.0060 | 0.0000 | 0.0000 |
| MB-5189 | 0.0320 | 0.0000 | 0.0109 | 0.0736 | 0.0000 | 0.0536 | 0.0000 | 0.0690 | 0.0154 | 0.0000 | 0.0141 | 0.0684 | 0.0138 | 0.0249 | 0.0817 | 0.3347 | 0.0071 | 0.0000 | 0.2009 | 0.0000 | 0.0000 | 0.0000 |
| MB-5281 | 0.0000 | 0.0000 | 0.0220 | 0.0000 | 0.0000 | 0.0602 | 0.0000 | 0.0903 | 0.0439 | 0.0000 | 0.0000 | 0.0335 | 0.0000 | 0.4353 | 0.0523 | 0.2060 | 0.0000 | 0.0000 | 0.0509 | 0.0055 | 0.0000 | 0.0000 |
| MB-5212 | 0.0060 | 0.0000 | 0.0204 | 0.0648 | 0.0390 | 0.0357 | 0.0199 | 0.0999 | 0.0181 | 0.0315 | 0.0000 | 0.0790 | 0.0296 | 0.1384 | 0.0932 | 0.1525 | 0.0047 | 0.0455 | 0.1217 | 0.0000 | 0.0000 | 0.0000 |
| MB-5172 | 0.0312 | 0.0000 | 0.0000 | 0.0505 | 0.0000 | 0.1474 | 0.0000 | 0.0000 | 0.0306 | 0.1190 | 0.0000 | 0.0600 | 0.0140 | 0.1492 | 0.1412 | 0.1175 | 0.0000 | 0.0000 | 0.1394 | 0.0000 | 0.0000 | 0.0000 |
| MB-5183 | 0.0127 | 0.0000 | 0.0025 | 0.0705 | 0.0000 | 0.0854 | 0.0000 | 0.1233 | 0.0100 | 0.0000 | 0.0000 | 0.0369 | 0.0362 | 0.3718 | 0.0572 | 0.1356 | 0.0000 | 0.0000 | 0.0225 | 0.0355 | 0.0000 | 0.0000 |
| MB-5185 | 0.0175 | 0.0086 | 0.0000 | 0.0000 | 0.0390 | 0.0698 | 0.0000 | 0.0186 | 0.0578 | 0.0544 | 0.0000 | 0.0526 | 0.0000 | 0.2980 | 0.0137 | 0.2715 | 0.0000 | 0.0201 | 0.0000 | 0.0784 | 0.0000 | 0.0000 |
| MB-5014 | 0.0174 | 0.0000 | 0.0455 | 0.0378 | 0.0000 | 0.0439 | 0.0000 | 0.0526 | 0.0458 | 0.0000 | 0.0000 | 0.0560 | 0.0262 | 0.1352 | 0.0888 | 0.3038 | 0.0211 | 0.0000 | 0.1257 | 0.0000 | 0.0000 | 0.0002 |
| MB-4994 | 0.0082 | 0.0017 | 0.0000 | 0.0181 | 0.0000 | 0.0921 | 0.0000 | 0.1346 | 0.0175 | 0.0007 | 0.0000 | 0.0564 | 0.0166 | 0.0000 | 0.0698 | 0.4355 | 0.0314 | 0.0000 | 0.0000 | 0.1174 | 0.0000 | 0.0000 |
| MB-5017 | 0.0317 | 0.0000 | 0.0000 | 0.1887 | 0.0409 | 0.0000 | 0.0324 | 0.0603 | 0.0436 | 0.0588 | 0.0000 | 0.0916 | 0.0000 | 0.1408 | 0.1121 | 0.0842 | 0.0000 | 0.0000 | 0.1149 | 0.0000 | 0.0000 | 0.0000 |
| MB-4982 | 0.0000 | 0.0000 | 0.0346 | 0.0990 | 0.0000 | 0.0000 | 0.0000 | 0.1080 | 0.0831 | 0.0538 | 0.0000 | 0.0483 | 0.0000 | 0.3511 | 0.0418 | 0.1271 | 0.0000 | 0.0000 | 0.0532 | 0.0000 | 0.0000 | 0.0000 |
| MB-5004 | 0.0000 | 0.0118 | 0.0629 | 0.0782 | 0.0000 | 0.0000 | 0.0080 | 0.1863 | 0.0365 | 0.0042 | 0.0000 | 0.1031 | 0.0089 | 0.2350 | 0.1094 | 0.0871 | 0.0000 | 0.0000 | 0.0685 | 0.0000 | 0.0000 | 0.0000 |
| MB-4986 | 0.0025 | 0.0000 | 0.0045 | 0.0430 | 0.0000 | 0.0000 | 0.0000 | 0.0585 | 0.0520 | 0.0651 | 0.0000 | 0.0000 | 0.0000 | 0.4385 | 0.0489 | 0.2772 | 0.0000 | 0.0000 | 0.0068 | 0.0000 | 0.0000 | 0.0031 |
| MB-5327 | 0.0204 | 0.0000 | 0.2340 | 0.0000 | 0.0000 | 0.0652 | 0.0000 | 0.0325 | 0.0371 | 0.0297 | 0.0000 | 0.0162 | 0.0000 | 0.3287 | 0.0618 | 0.1242 | 0.0000 | 0.0000 | 0.0000 | 0.0501 | 0.0000 | 0.0000 |
| MB-5341 | 0.0000 | 0.0000 | 0.0285 | 0.1366 | 0.0033 | 0.0000 | 0.0000 | 0.0869 | 0.0686 | 0.0183 | 0.0000 | 0.0000 | 0.0123 | 0.0130 | 0.0412 | 0.0000 | 0.0543 | 0.0000 | 0.5325 | 0.0045 | 0.0000 | 0.0000 |
| MB-5323 | 0.0385 | 0.0226 | 0.0088 | 0.0743 | 0.0704 | 0.0610 | 0.0000 | 0.0673 | 0.0311 | 0.0752 | 0.0000 | 0.0977 | 0.0391 | 0.1636 | 0.1173 | 0.0772 | 0.0000 | 0.0000 | 0.0561 | 0.0000 | 0.0000 | 0.0000 |
| MB-5328 | 0.0000 | 0.0044 | 0.0621 | 0.1896 | 0.0016 | 0.0413 | 0.0202 | 0.0483 | 0.0000 | 0.0596 | 0.0000 | 0.0596 | 0.0321 | 0.1734 | 0.0730 | 0.1602 | 0.0049 | 0.0000 | 0.0697 | 0.0000 | 0.0000 | 0.0000 |
| MB-5318 | 0.0717 | 0.0000 | 0.0017 | 0.0165 | 0.0280 | 0.0000 | 0.0000 | 0.0487 | 0.0562 | 0.0483 | 0.0000 | 0.0047 | 0.0000 | 0.4193 | 0.0463 | 0.1991 | 0.0000 | 0.0056 | 0.0539 | 0.0000 | 0.0000 | 0.0000 |
| MB-5287 | 0.0025 | 0.0000 | 0.0359 | 0.2375 | 0.0000 | 0.0000 | 0.0000 | 0.1047 | 0.0037 | 0.0000 | 0.0000 | 0.0425 | 0.0186 | 0.0800 | 0.1054 | 0.1853 | 0.0000 | 0.0000 | 0.1839 | 0.0000 | 0.0000 | 0.0000 |
| MB-5324 | 0.0536 | 0.0000 | 0.0020 | 0.0672 | 0.0000 | 0.0920 | 0.0000 | 0.0566 | 0.0081 | 0.0015 | 0.0000 | 0.0201 | 0.0417 | 0.2162 | 0.1035 | 0.2738 | 0.0000 | 0.0000 | 0.0638 | 0.0000 | 0.0000 | 0.0000 |
| MB-4682 | 0.0000 | 0.1100 | 0.0000 | 0.0351 | 0.0803 | 0.0000 | 0.0000 | 0.0338 | 0.0431 | 0.0677 | 0.0000 | 0.0576 | 0.0037 | 0.1123 | 0.0908 | 0.1418 | 0.0183 | 0.0000 | 0.2053 | 0.0000 | 0.0000 | 0.0000 |
| MB-4710 | 0.0000 | 0.0186 | 0.0065 | 0.1337 | 0.0240 | 0.0040 | 0.0000 | 0.1070 | 0.0000 | 0.0000 | 0.0000 | 0.0649 | 0.0126 | 0.1362 | 0.1132 | 0.1548 | 0.0000 | 0.0000 | 0.2244 | 0.0000 | 0.0000 | 0.0000 |
| MB-4701 | 0.0000 | 0.0000 | 0.0215 | 0.0896 | 0.0000 | 0.1580 | 0.0000 | 0.0660 | 0.0093 | 0.0000 | 0.0498 | 0.0000 | 0.0307 | 0.3962 | 0.0693 | 0.0675 | 0.0000 | 0.0000 | 0.0421 | 0.0000 | 0.0000 | 0.0000 |
| MB-4709 | 0.0000 | 0.0000 | 0.0319 | 0.1180 | 0.0000 | 0.0974 | 0.0000 | 0.0707 | 0.0058 | 0.0000 | 0.0000 | 0.0451 | 0.0000 | 0.0257 | 0.0673 | 0.3897 | 0.0165 | 0.0052 | 0.1266 | 0.0000 | 0.0000 | 0.0000 |
| MB-4686 | 0.0000 | 0.0000 | 0.0116 | 0.0354 | 0.0000 | 0.1370 | 0.0000 | 0.0854 | 0.0113 | 0.0086 | 0.0059 | 0.0234 | 0.0000 | 0.2438 | 0.1441 | 0.1219 | 0.0064 | 0.0000 | 0.1652 | 0.0000 | 0.0000 | 0.0000 |
| MB-4706 | 0.0000 | 0.0122 | 0.0000 | 0.2149 | 0.0000 | 0.0000 | 0.0000 | 0.1150 | 0.0102 | 0.0858 | 0.0000 | 0.1111 | 0.0269 | 0.0239 | 0.1579 | 0.1019 | 0.0244 | 0.0000 | 0.1159 | 0.0000 | 0.0000 | 0.0000 |
| MB-4719 | 0.0411 | 0.0000 | 0.0009 | 0.0270 | 0.0000 | 0.0886 | 0.0000 | 0.0396 | 0.0000 | 0.0000 | 0.0000 | 0.0662 | 0.0098 | 0.1356 | 0.0387 | 0.4025 | 0.0244 | 0.0000 | 0.0823 | 0.0432 | 0.0000 | 0.0000 |
| MB-4702 | 0.0435 | 0.0000 | 0.0113 | 0.0286 | 0.0000 | 0.0951 | 0.0031 | 0.0569 | 0.0332 | 0.0808 | 0.0000 | 0.0031 | 0.0000 | 0.1923 | 0.1590 | 0.2237 | 0.0000 | 0.0000 | 0.0694 | 0.0000 | 0.0000 | 0.0000 |
| MB-4908 | 0.0071 | 0.0000 | 0.0085 | 0.0000 | 0.0000 | 0.0678 | 0.0000 | 0.0835 | 0.0441 | 0.0399 | 0.0000 | 0.0513 | 0.0000 | 0.4061 | 0.1039 | 0.1612 | 0.0000 | 0.0000 | 0.0265 | 0.0000 | 0.0000 | 0.0000 |
| MB-4871 | 0.0000 | 0.0286 | 0.0069 | 0.0624 | 0.0000 | 0.0000 | 0.0000 | 0.0989 | 0.0350 | 0.0000 | 0.0000 | 0.0324 | 0.0000 | 0.4588 | 0.1223 | 0.0573 | 0.0000 | 0.0000 | 0.0973 | 0.0000 | 0.0000 | 0.0000 |
| MB-4906 | 0.0853 | 0.0000 | 0.0733 | 0.0608 | 0.0000 | 0.0476 | 0.0000 | 0.1197 | 0.0512 | 0.0000 | 0.0366 | 0.0164 | 0.0000 | 0.1183 | 0.0781 | 0.1226 | 0.0000 | 0.0000 | 0.1547 | 0.0000 | 0.0000 | 0.0354 |
| MB-4911 | 0.0364 | 0.0000 | 0.0710 | 0.0609 | 0.0000 | 0.0000 | 0.0000 | 0.0819 | 0.0103 | 0.0000 | 0.0000 | 0.0981 | 0.0136 | 0.1354 | 0.1137 | 0.1548 | 0.0000 | 0.0000 | 0.0000 | 0.2194 | 0.0000 | 0.0043 |
| MB-4866 | 0.0010 | 0.0041 | 0.0000 | 0.1166 | 0.0000 | 0.0000 | 0.0000 | 0.1384 | 0.0315 | 0.1545 | 0.0000 | 0.0601 | 0.0153 | 0.1677 | 0.1417 | 0.0528 | 0.0000 | 0.0000 | 0.1163 | 0.0000 | 0.0000 | 0.0000 |
| MB-4858 | 0.0446 | 0.0000 | 0.0083 | 0.0569 | 0.0000 | 0.0340 | 0.0000 | 0.0259 | 0.0460 | 0.0506 | 0.0000 | 0.0449 | 0.0000 | 0.3098 | 0.1363 | 0.1598 | 0.0000 | 0.0000 | 0.0827 | 0.0000 | 0.0000 | 0.0000 |
| MB-4862 | 0.0000 | 0.0051 | 0.0000 | 0.0713 | 0.0029 | 0.0000 | 0.0000 | 0.0721 | 0.0370 | 0.0503 | 0.0000 | 0.0513 | 0.0000 | 0.2974 | 0.0392 | 0.2870 | 0.0000 | 0.0000 | 0.0865 | 0.0000 | 0.0000 | 0.0000 |
| MB-4872 | 0.0581 | 0.0000 | 0.0000 | 0.1766 | 0.0796 | 0.0274 | 0.0000 | 0.0292 | 0.0336 | 0.0191 | 0.0000 | 0.1222 | 0.0544 | 0.0753 | 0.1107 | 0.0668 | 0.0000 | 0.0000 | 0.1471 | 0.0000 | 0.0000 | 0.0000 |
| MB-4887 | 0.0119 | 0.0000 | 0.0007 | 0.0775 | 0.0000 | 0.0988 | 0.0000 | 0.0220 | 0.0143 | 0.0000 | 0.0000 | 0.0578 | 0.0000 | 0.3556 | 0.1438 | 0.0560 | 0.0000 | 0.0000 | 0.1616 | 0.0000 | 0.0000 | 0.0000 |
| MB-4867 | 0.0278 | 0.0000 | 0.0058 | 0.1609 | 0.0000 | 0.0794 | 0.0000 | 0.0479 | 0.0196 | 0.0000 | 0.0000 | 0.0572 | 0.0329 | 0.0595 | 0.1136 | 0.1276 | 0.0158 | 0.0000 | 0.2521 | 0.0000 | 0.0000 | 0.0000 |
| MB-4888 | 0.0473 | 0.0000 | 0.1061 | 0.0589 | 0.0000 | 0.0694 | 0.0000 | 0.0390 | 0.0000 | 0.0093 | 0.0077 | 0.0000 | 0.0096 | 0.2641 | 0.2542 | 0.0967 | 0.0134 | 0.0000 | 0.0241 | 0.0000 | 0.0000 | 0.0000 |
| MB-4929 | 0.0181 | 0.0000 | 0.0000 | 0.0420 | 0.0000 | 0.0870 | 0.0000 | 0.0000 | 0.0494 | 0.0000 | 0.0713 | 0.0000 | 0.0000 | 0.4327 | 0.0500 | 0.1062 | 0.0000 | 0.0000 | 0.0746 | 0.0434 | 0.0000 | 0.0254 |
| MB-4945 | 0.0060 | 0.0000 | 0.0783 | 0.0790 | 0.0277 | 0.1080 | 0.0000 | 0.0822 | 0.0178 | 0.0271 | 0.0000 | 0.0165 | 0.0147 | 0.2761 | 0.1358 | 0.0736 | 0.0041 | 0.0000 | 0.0530 | 0.0000 | 0.0000 | 0.0000 |
| MB-4930 | 0.0000 | 0.0098 | 0.0654 | 0.0776 | 0.0000 | 0.0154 | 0.0000 | 0.1027 | 0.0158 | 0.0000 | 0.0000 | 0.0242 | 0.0060 | 0.3074 | 0.1385 | 0.0890 | 0.0000 | 0.0000 | 0.1480 | 0.0000 | 0.0000 | 0.0000 |
| MB-4894 | 0.0657 | 0.0000 | 0.0389 | 0.0577 | 0.0000 | 0.0000 | 0.0000 | 0.1364 | 0.0886 | 0.0000 | 0.0000 | 0.0539 | 0.0000 | 0.2448 | 0.1278 | 0.1626 | 0.0000 | 0.0000 | 0.0236 | 0.0000 | 0.0000 | 0.0000 |
| MB-4898 | 0.1491 | 0.0000 | 0.0424 | 0.0622 | 0.0094 | 0.0869 | 0.0000 | 0.0000 | 0.0627 | 0.0000 | 0.0246 | 0.0238 | 0.0434 | 0.0142 | 0.0295 | 0.2656 | 0.0122 | 0.0000 | 0.1739 | 0.0000 | 0.0000 | 0.0000 |
| MB-4670 | 0.0681 | 0.0000 | 0.0339 | 0.0662 | 0.0000 | 0.0267 | 0.0000 | 0.0374 | 0.0356 | 0.0000 | 0.1288 | 0.0000 | 0.0060 | 0.2304 | 0.0435 | 0.2254 | 0.0128 | 0.0000 | 0.0851 | 0.0000 | 0.0000 | 0.0000 |
| MB-5013 | 0.1076 | 0.0000 | 0.0043 | 0.0000 | 0.0000 | 0.1260 | 0.0000 | 0.1857 | 0.0000 | 0.0000 | 0.0000 | 0.0808 | 0.0133 | 0.0183 | 0.0537 | 0.2837 | 0.0303 | 0.0000 | 0.0963 | 0.0000 | 0.0000 | 0.0000 |
| MB-4977 | 0.0000 | 0.0104 | 0.0148 | 0.0242 | 0.0000 | 0.0673 | 0.0000 | 0.0601 | 0.0388 | 0.0919 | 0.0000 | 0.0678 | 0.0000 | 0.1715 | 0.0988 | 0.2395 | 0.0000 | 0.0000 | 0.1149 | 0.0000 | 0.0000 | 0.0000 |
| MB-4967 | 0.0000 | 0.0065 | 0.0000 | 0.1196 | 0.0000 | 0.0000 | 0.0000 | 0.0665 | 0.0320 | 0.0930 | 0.0000 | 0.0365 | 0.0000 | 0.3087 | 0.0933 | 0.1398 | 0.0000 | 0.0000 | 0.1041 | 0.0000 | 0.0000 | 0.0000 |
| MB-4981 | 0.0272 | 0.0000 | 0.0000 | 0.1969 | 0.0000 | 0.0000 | 0.0000 | 0.0780 | 0.0000 | 0.1646 | 0.0000 | 0.0103 | 0.0211 | 0.0000 | 0.1525 | 0.1383 | 0.0235 | 0.0000 | 0.0760 | 0.1116 | 0.0000 | 0.0000 |
| MB-4003 | 0.0071 | 0.0000 | 0.0172 | 0.0682 | 0.0089 | 0.0000 | 0.0686 | 0.1154 | 0.1115 | 0.0000 | 0.0460 | 0.0589 | 0.0006 | 0.3384 | 0.0769 | 0.0127 | 0.0000 | 0.0000 | 0.0697 | 0.0000 | 0.0000 | 0.0000 |
| MB-4968 | 0.0295 | 0.0000 | 0.0288 | 0.0770 | 0.0000 | 0.1705 | 0.0000 | 0.0461 | 0.0000 | 0.0000 | 0.0000 | 0.0518 | 0.0355 | 0.0000 | 0.1178 | 0.1878 | 0.0362 | 0.0000 | 0.2190 | 0.0000 | 0.0000 | 0.0000 |
| MB-5052 | 0.0579 | 0.0000 | 0.0560 | 0.1119 | 0.0000 | 0.1558 | 0.0000 | 0.0465 | 0.0000 | 0.0778 | 0.0000 | 0.0449 | 0.0068 | 0.1641 | 0.1171 | 0.0899 | 0.0000 | 0.0000 | 0.0712 | 0.0000 | 0.0000 | 0.0000 |
| MB-5049 | 0.0641 | 0.0000 | 0.0128 | 0.0656 | 0.0000 | 0.1547 | 0.0000 | 0.0188 | 0.0233 | 0.0000 | 0.0000 | 0.0372 | 0.0038 | 0.0214 | 0.0596 | 0.3260 | 0.0282 | 0.0000 | 0.1808 | 0.0000 | 0.0000 | 0.0039 |
| MB-5041 | 0.0246 | 0.0000 | 0.0003 | 0.0899 | 0.0773 | 0.0243 | 0.0376 | 0.0600 | 0.0187 | 0.0787 | 0.0000 | 0.0376 | 0.0016 | 0.3047 | 0.1444 | 0.0377 | 0.0000 | 0.0000 | 0.0627 | 0.0000 | 0.0000 | 0.0000 |
| MB-5044 | 0.0610 | 0.0000 | 0.0123 | 0.0818 | 0.0000 | 0.0709 | 0.0000 | 0.0176 | 0.0776 | 0.0000 | 0.0000 | 0.0179 | 0.0000 | 0.1008 | 0.0355 | 0.2844 | 0.0166 | 0.0000 | 0.2236 | 0.0000 | 0.0000 | 0.0000 |
| MB-5072 | 0.0068 | 0.0001 | 0.0000 | 0.0375 | 0.0000 | 0.1366 | 0.0000 | 0.0169 | 0.0000 | 0.0543 | 0.0000 | 0.0557 | 0.0000 | 0.1499 | 0.0649 | 0.3180 | 0.0268 | 0.0000 | 0.1280 | 0.0000 | 0.0000 | 0.0044 |
| MB-4171 | 0.0000 | 0.0139 | 0.0691 | 0.1999 | 0.0268 | 0.0000 | 0.0000 | 0.1212 | 0.0713 | 0.0000 | 0.0000 | 0.0747 | 0.0313 | 0.1038 | 0.1024 | 0.1108 | 0.0000 | 0.0000 | 0.0748 | 0.0000 | 0.0000 | 0.0000 |
| MB-5053 | 0.0183 | 0.0216 | 0.0040 | 0.0723 | 0.0000 | 0.0324 | 0.0000 | 0.1149 | 0.0253 | 0.0259 | 0.0000 | 0.0805 | 0.0108 | 0.1360 | 0.0968 | 0.1935 | 0.0088 | 0.0000 | 0.1590 | 0.0000 | 0.0000 | 0.0000 |
| MB-5045 | 0.0000 | 0.0019 | 0.0192 | 0.1835 | 0.0000 | 0.0000 | 0.0342 | 0.1276 | 0.0087 | 0.0000 | 0.0000 | 0.1069 | 0.0043 | 0.1573 | 0.0911 | 0.1442 | 0.0205 | 0.0000 | 0.1007 | 0.0000 | 0.0000 | 0.0000 |
| MB-5116 | 0.0419 | 0.0000 | 0.0000 | 0.0553 | 0.0000 | 0.0812 | 0.0000 | 0.1042 | 0.0272 | 0.0000 | 0.0000 | 0.0190 | 0.0188 | 0.0790 | 0.1412 | 0.2683 | 0.0212 | 0.0000 | 0.1427 | 0.0000 | 0.0000 | 0.0000 |
| MB-5120 | 0.0000 | 0.0000 | 0.0087 | 0.0478 | 0.0000 | 0.0498 | 0.0494 | 0.0823 | 0.0448 | 0.0490 | 0.0176 | 0.0220 | 0.0000 | 0.3013 | 0.1661 | 0.0799 | 0.0000 | 0.0000 | 0.0815 | 0.0000 | 0.0000 | 0.0000 |
| MB-5074 | 0.0123 | 0.0003 | 0.0026 | 0.1350 | 0.0000 | 0.0754 | 0.0000 | 0.1039 | 0.0049 | 0.0562 | 0.0000 | 0.1022 | 0.0413 | 0.0000 | 0.1223 | 0.2030 | 0.0000 | 0.0000 | 0.1405 | 0.0000 | 0.0000 | 0.0000 |
| MB-5119 | 0.0351 | 0.0000 | 0.0009 | 0.0737 | 0.0077 | 0.1016 | 0.0000 | 0.0958 | 0.0496 | 0.0169 | 0.0000 | 0.0377 | 0.0595 | 0.1499 | 0.1016 | 0.1409 | 0.0068 | 0.0000 | 0.1223 | 0.0000 | 0.0000 | 0.0000 |
| MB-5114 | 0.0000 | 0.0512 | 0.0000 | 0.0962 | 0.0000 | 0.0944 | 0.0153 | 0.0870 | 0.0000 | 0.0685 | 0.0000 | 0.0754 | 0.0398 | 0.1136 | 0.1780 | 0.0968 | 0.0000 | 0.0000 | 0.0838 | 0.0000 | 0.0000 | 0.0000 |
| MB-4230 | 0.0000 | 0.0005 | 0.0053 | 0.1025 | 0.0000 | 0.1291 | 0.0000 | 0.1104 | 0.0321 | 0.0936 | 0.0000 | 0.0442 | 0.0214 | 0.0295 | 0.1091 | 0.2209 | 0.0029 | 0.0000 | 0.0987 | 0.0000 | 0.0000 | 0.0000 |
| MB-4154 | 0.0029 | 0.0000 | 0.0504 | 0.0374 | 0.0514 | 0.0182 | 0.0199 | 0.0622 | 0.0101 | 0.1347 | 0.0000 | 0.0161 | 0.0000 | 0.3625 | 0.1224 | 0.0642 | 0.0229 | 0.0000 | 0.0244 | 0.0000 | 0.0000 | 0.0000 |
| MB-5115 | 0.0291 | 0.0000 | 0.0257 | 0.0000 | 0.0424 | 0.0048 | 0.0000 | 0.0913 | 0.0333 | 0.0451 | 0.0286 | 0.0000 | 0.0248 | 0.2988 | 0.2218 | 0.1136 | 0.0000 | 0.0000 | 0.0409 | 0.0000 | 0.0000 | 0.0000 |
| MB-4737 | 0.0100 | 0.0004 | 0.0000 | 0.0037 | 0.0184 | 0.0707 | 0.0000 | 0.0000 | 0.0188 | 0.0843 | 0.0000 | 0.0507 | 0.0424 | 0.4453 | 0.0976 | 0.1130 | 0.0000 | 0.0000 | 0.0447 | 0.0000 | 0.0000 | 0.0000 |
| MB-4764 | 0.0409 | 0.0000 | 0.0077 | 0.0737 | 0.0131 | 0.1144 | 0.0000 | 0.0348 | 0.0000 | 0.0000 | 0.0000 | 0.0386 | 0.0193 | 0.2543 | 0.0975 | 0.1204 | 0.0083 | 0.0000 | 0.1770 | 0.0000 | 0.0000 | 0.0000 |
| MB-4735 | 0.0174 | 0.0000 | 0.0128 | 0.0000 | 0.0000 | 0.0542 | 0.0000 | 0.0300 | 0.0223 | 0.0000 | 0.0000 | 0.0208 | 0.0000 | 0.3127 | 0.0415 | 0.1949 | 0.0000 | 0.0000 | 0.2470 | 0.0464 | 0.0000 | 0.0000 |
| MB-4730 | 0.0000 | 0.0092 | 0.0000 | 0.1010 | 0.0000 | 0.0000 | 0.0214 | 0.1626 | 0.0585 | 0.0163 | 0.0000 | 0.0545 | 0.0280 | 0.0000 | 0.1254 | 0.3029 | 0.0135 | 0.0000 | 0.1067 | 0.0000 | 0.0000 | 0.0000 |
| MB-4733 | 0.1010 | 0.0000 | 0.0000 | 0.1531 | 0.0000 | 0.0133 | 0.0388 | 0.1025 | 0.0095 | 0.0000 | 0.0000 | 0.0699 | 0.0152 | 0.1467 | 0.1340 | 0.1335 | 0.0000 | 0.0000 | 0.0828 | 0.0000 | 0.0000 | 0.0000 |
| MB-4758 | 0.0277 | 0.0000 | 0.0076 | 0.0386 | 0.0000 | 0.0000 | 0.0000 | 0.0873 | 0.0099 | 0.0000 | 0.0287 | 0.0000 | 0.0000 | 0.4533 | 0.0131 | 0.2190 | 0.0000 | 0.0308 | 0.0000 | 0.0768 | 0.0000 | 0.0070 |
| MB-5033 | 0.0439 | 0.0000 | 0.0231 | 0.0868 | 0.0000 | 0.0000 | 0.0000 | 0.0874 | 0.0000 | 0.0000 | 0.0000 | 0.0377 | 0.0000 | 0.2980 | 0.0575 | 0.2270 | 0.0000 | 0.0000 | 0.1332 | 0.0000 | 0.0000 | 0.0053 |
| MB-4741 | 0.0057 | 0.0000 | 0.0191 | 0.0494 | 0.0776 | 0.0182 | 0.0000 | 0.1034 | 0.0269 | 0.0630 | 0.0000 | 0.0862 | 0.0130 | 0.0000 | 0.1131 | 0.0984 | 0.0324 | 0.0000 | 0.2934 | 0.0000 | 0.0000 | 0.0000 |
| MB-4732 | 0.0000 | 0.0000 | 0.0585 | 0.1525 | 0.0000 | 0.0000 | 0.0073 | 0.0831 | 0.0012 | 0.0120 | 0.0000 | 0.1021 | 0.0444 | 0.2105 | 0.1922 | 0.1115 | 0.0104 | 0.0029 | 0.0116 | 0.0000 | 0.0000 | 0.0000 |
| MB-5305 | 0.1217 | 0.0000 | 0.0116 | 0.0000 | 0.0019 | 0.0000 | 0.0000 | 0.0826 | 0.0316 | 0.0477 | 0.0192 | 0.0000 | 0.0000 | 0.3224 | 0.0171 | 0.3122 | 0.0000 | 0.0000 | 0.0000 | 0.0320 | 0.0000 | 0.0000 |
| MB-5256 | 0.0000 | 0.0198 | 0.0000 | 0.0861 | 0.0000 | 0.0580 | 0.0000 | 0.1428 | 0.0338 | 0.0813 | 0.0000 | 0.0879 | 0.0272 | 0.0825 | 0.1120 | 0.1430 | 0.0073 | 0.0000 | 0.1183 | 0.0000 | 0.0000 | 0.0000 |
| MB-5273 | 0.0000 | 0.0306 | 0.0000 | 0.0026 | 0.0000 | 0.0258 | 0.0000 | 0.0560 | 0.0376 | 0.0302 | 0.0363 | 0.0000 | 0.0000 | 0.3510 | 0.0240 | 0.2995 | 0.0000 | 0.0000 | 0.0751 | 0.0315 | 0.0000 | 0.0000 |
| MB-5236 | 0.1107 | 0.0000 | 0.0353 | 0.0877 | 0.2433 | 0.0000 | 0.0000 | 0.0813 | 0.0158 | 0.0000 | 0.0391 | 0.0199 | 0.0141 | 0.1648 | 0.0907 | 0.0866 | 0.0000 | 0.0000 | 0.0105 | 0.0000 | 0.0000 | 0.0000 |
| MB-5238 | 0.0253 | 0.0296 | 0.0000 | 0.0894 | 0.0366 | 0.2318 | 0.0000 | 0.0570 | 0.0000 | 0.0000 | 0.0433 | 0.0317 | 0.0455 | 0.0376 | 0.1349 | 0.1242 | 0.0028 | 0.0000 | 0.1104 | 0.0000 | 0.0000 | 0.0000 |
| MB-5233 | 0.0928 | 0.0000 | 0.0224 | 0.0000 | 0.0035 | 0.0000 | 0.0000 | 0.0230 | 0.0860 | 0.0684 | 0.0054 | 0.0000 | 0.0000 | 0.4955 | 0.0000 | 0.1087 | 0.0000 | 0.0649 | 0.0293 | 0.0000 | 0.0000 | 0.0000 |
| MB-5244 | 0.0664 | 0.0000 | 0.0093 | 0.0495 | 0.0000 | 0.0276 | 0.0000 | 0.0102 | 0.0590 | 0.0000 | 0.0000 | 0.0194 | 0.0073 | 0.2695 | 0.0695 | 0.3528 | 0.0000 | 0.0000 | 0.0596 | 0.0000 | 0.0000 | 0.0000 |
| MB-5253 | 0.0092 | 0.0234 | 0.0110 | 0.0888 | 0.0316 | 0.1478 | 0.0140 | 0.1140 | 0.0000 | 0.0417 | 0.0000 | 0.0971 | 0.0323 | 0.1049 | 0.0850 | 0.1279 | 0.0019 | 0.0118 | 0.0574 | 0.0000 | 0.0000 | 0.0000 |
| MB-5260 | 0.0094 | 0.0063 | 0.0000 | 0.0000 | 0.0000 | 0.1792 | 0.0000 | 0.0358 | 0.0000 | 0.0000 | 0.0000 | 0.0856 | 0.0000 | 0.1107 | 0.0187 | 0.1119 | 0.0000 | 0.0000 | 0.4423 | 0.0000 | 0.0000 | 0.0000 |
| MB-4998 | 0.0240 | 0.0000 | 0.0041 | 0.0515 | 0.0000 | 0.0261 | 0.0000 | 0.0352 | 0.0067 | 0.0316 | 0.0000 | 0.0608 | 0.0075 | 0.0540 | 0.0428 | 0.2858 | 0.0000 | 0.0000 | 0.3584 | 0.0115 | 0.0000 | 0.0000 |
| MB-4993 | 0.0128 | 0.0000 | 0.0203 | 0.0000 | 0.0000 | 0.0376 | 0.0000 | 0.0692 | 0.0236 | 0.0309 | 0.0086 | 0.0212 | 0.0000 | 0.4870 | 0.0746 | 0.1566 | 0.0000 | 0.0000 | 0.0577 | 0.0000 | 0.0000 | 0.0000 |
| MB-5001 | 0.0006 | 0.0000 | 0.0158 | 0.0668 | 0.0010 | 0.0837 | 0.0000 | 0.0621 | 0.0000 | 0.0000 | 0.0000 | 0.0435 | 0.0220 | 0.0000 | 0.0638 | 0.1594 | 0.0277 | 0.0000 | 0.4535 | 0.0000 | 0.0000 | 0.0000 |
| MB-5084 | 0.0350 | 0.0000 | 0.0000 | 0.0000 | 0.0051 | 0.1325 | 0.0000 | 0.1305 | 0.0000 | 0.2355 | 0.0000 | 0.0070 | 0.0153 | 0.0676 | 0.1087 | 0.1950 | 0.0212 | 0.0036 | 0.0430 | 0.0000 | 0.0000 | 0.0000 |
| MB-4969 | 0.0311 | 0.0000 | 0.0000 | 0.0355 | 0.0000 | 0.0869 | 0.0000 | 0.1091 | 0.0003 | 0.0000 | 0.0000 | 0.1093 | 0.0000 | 0.1690 | 0.1095 | 0.2272 | 0.0000 | 0.0000 | 0.1203 | 0.0000 | 0.0000 | 0.0019 |
| MB-4999 | 0.0000 | 0.0276 | 0.0000 | 0.0000 | 0.0000 | 0.0302 | 0.0000 | 0.1938 | 0.0552 | 0.0000 | 0.0000 | 0.1131 | 0.0425 | 0.1792 | 0.0364 | 0.1789 | 0.0404 | 0.0000 | 0.1027 | 0.0000 | 0.0000 | 0.0000 |
| MB-5011 | 0.0000 | 0.0453 | 0.0000 | 0.1072 | 0.0199 | 0.0409 | 0.0000 | 0.0822 | 0.0000 | 0.0022 | 0.0302 | 0.0246 | 0.0020 | 0.0177 | 0.0963 | 0.4169 | 0.0080 | 0.0265 | 0.0804 | 0.0000 | 0.0000 | 0.0000 |
| MB-4959 | 0.0397 | 0.0000 | 0.0002 | 0.1691 | 0.0000 | 0.0000 | 0.0000 | 0.0568 | 0.0495 | 0.0358 | 0.0000 | 0.0474 | 0.0190 | 0.2930 | 0.0865 | 0.0998 | 0.0000 | 0.0000 | 0.1032 | 0.0000 | 0.0000 | 0.0000 |
| MB-4599 | 0.0857 | 0.0000 | 0.0137 | 0.0423 | 0.0000 | 0.0000 | 0.0000 | 0.1716 | 0.0892 | 0.0000 | 0.0000 | 0.0919 | 0.0084 | 0.0809 | 0.0158 | 0.2828 | 0.0000 | 0.0000 | 0.1007 | 0.0000 | 0.0172 | 0.0000 |
| MB-4616 | 0.0867 | 0.0000 | 0.0286 | 0.0000 | 0.0000 | 0.1211 | 0.0000 | 0.0526 | 0.0154 | 0.0000 | 0.0000 | 0.0640 | 0.0006 | 0.1012 | 0.0099 | 0.1336 | 0.0000 | 0.0073 | 0.3792 | 0.0000 | 0.0000 | 0.0000 |
| MB-4623 | 0.0062 | 0.0258 | 0.0587 | 0.0084 | 0.0004 | 0.0122 | 0.0000 | 0.0833 | 0.0436 | 0.0644 | 0.0000 | 0.0451 | 0.0000 | 0.2720 | 0.1083 | 0.1728 | 0.0000 | 0.0000 | 0.0988 | 0.0000 | 0.0000 | 0.0000 |
| MB-4644 | 0.0336 | 0.0000 | 0.0000 | 0.0000 | 0.0487 | 0.0000 | 0.0000 | 0.0637 | 0.0997 | 0.0090 | 0.1418 | 0.0000 | 0.0000 | 0.4251 | 0.0543 | 0.0863 | 0.0000 | 0.0000 | 0.0000 | 0.0378 | 0.0000 | 0.0000 |
| MB-4869 | 0.0000 | 0.0291 | 0.0000 | 0.0173 | 0.0769 | 0.0000 | 0.0000 | 0.0431 | 0.0738 | 0.0126 | 0.0070 | 0.0155 | 0.0007 | 0.3813 | 0.0111 | 0.2192 | 0.0000 | 0.0000 | 0.0000 | 0.1124 | 0.0000 | 0.0000 |
| MB-4878 | 0.0198 | 0.0000 | 0.2302 | 0.0325 | 0.0000 | 0.0028 | 0.0000 | 0.0909 | 0.0260 | 0.0366 | 0.0120 | 0.0000 | 0.0000 | 0.3534 | 0.0841 | 0.0716 | 0.0000 | 0.0000 | 0.0353 | 0.0024 | 0.0000 | 0.0025 |
| MB-4851 | 0.0033 | 0.0000 | 0.0015 | 0.0981 | 0.0000 | 0.0000 | 0.0000 | 0.0984 | 0.0277 | 0.0110 | 0.0195 | 0.0097 | 0.0137 | 0.3138 | 0.0531 | 0.3160 | 0.0056 | 0.0000 | 0.0263 | 0.0023 | 0.0000 | 0.0000 |
| MB-4233 | 0.0061 | 0.0000 | 0.2666 | 0.0209 | 0.0380 | 0.0013 | 0.0000 | 0.0361 | 0.0327 | 0.0000 | 0.0000 | 0.0628 | 0.0000 | 0.1506 | 0.1136 | 0.0980 | 0.0180 | 0.0000 | 0.1430 | 0.0000 | 0.0000 | 0.0123 |
| MB-4937 | 0.0000 | 0.0000 | 0.0479 | 0.0180 | 0.0000 | 0.0394 | 0.0266 | 0.0945 | 0.0399 | 0.0241 | 0.0000 | 0.0515 | 0.0000 | 0.1796 | 0.1998 | 0.2271 | 0.0027 | 0.0000 | 0.0489 | 0.0000 | 0.0000 | 0.0000 |
| MB-4934 | 0.0090 | 0.0000 | 0.0155 | 0.0566 | 0.0000 | 0.0000 | 0.0000 | 0.0625 | 0.1248 | 0.0320 | 0.0000 | 0.0465 | 0.0540 | 0.2413 | 0.0772 | 0.1276 | 0.0000 | 0.0000 | 0.1530 | 0.0000 | 0.0000 | 0.0000 |
| MB-4899 | 0.0140 | 0.0458 | 0.0091 | 0.1119 | 0.0000 | 0.1349 | 0.0000 | 0.0747 | 0.0012 | 0.0000 | 0.0000 | 0.1008 | 0.0862 | 0.0023 | 0.0715 | 0.2196 | 0.0193 | 0.0000 | 0.1089 | 0.0000 | 0.0000 | 0.0000 |
| MB-4912 | 0.0082 | 0.0004 | 0.0071 | 0.0721 | 0.0000 | 0.0339 | 0.0000 | 0.0758 | 0.0758 | 0.0000 | 0.0000 | 0.0603 | 0.0233 | 0.1028 | 0.0324 | 0.3622 | 0.0495 | 0.0000 | 0.0880 | 0.0000 | 0.0000 | 0.0082 |
| MB-4935 | 0.0092 | 0.0000 | 0.0120 | 0.1150 | 0.0000 | 0.0000 | 0.0000 | 0.0923 | 0.1089 | 0.0000 | 0.0205 | 0.0872 | 0.0070 | 0.2302 | 0.1310 | 0.0840 | 0.0000 | 0.0263 | 0.0763 | 0.0000 | 0.0000 | 0.0000 |
| MB-4933 | 0.0224 | 0.0000 | 0.1094 | 0.0722 | 0.0000 | 0.0369 | 0.0000 | 0.0824 | 0.0262 | 0.1025 | 0.0000 | 0.1108 | 0.0000 | 0.1370 | 0.1091 | 0.0836 | 0.0000 | 0.0000 | 0.1076 | 0.0000 | 0.0000 | 0.0000 |
| MB-4900 | 0.0187 | 0.0000 | 0.0062 | 0.0270 | 0.0000 | 0.0620 | 0.0000 | 0.0096 | 0.0106 | 0.0000 | 0.0166 | 0.0000 | 0.0000 | 0.5021 | 0.0259 | 0.2880 | 0.0000 | 0.0000 | 0.0332 | 0.0000 | 0.0000 | 0.0000 |
| MB-4941 | 0.0392 | 0.0000 | 0.0731 | 0.0964 | 0.0038 | 0.0165 | 0.0000 | 0.0728 | 0.0722 | 0.0000 | 0.0000 | 0.0362 | 0.0000 | 0.2898 | 0.0486 | 0.1441 | 0.0000 | 0.0000 | 0.1005 | 0.0068 | 0.0000 | 0.0000 |
| MB-5221 | 0.0000 | 0.0128 | 0.0093 | 0.1248 | 0.0000 | 0.0000 | 0.0000 | 0.0477 | 0.0219 | 0.0000 | 0.0000 | 0.0802 | 0.0519 | 0.1745 | 0.0931 | 0.1927 | 0.0491 | 0.0000 | 0.1420 | 0.0000 | 0.0000 | 0.0000 |
| MB-5139 | 0.0000 | 0.0204 | 0.0153 | 0.0206 | 0.0766 | 0.0832 | 0.0000 | 0.1194 | 0.0000 | 0.0796 | 0.0000 | 0.0874 | 0.0136 | 0.1391 | 0.1530 | 0.1039 | 0.0000 | 0.0000 | 0.0879 | 0.0000 | 0.0000 | 0.0000 |
| MB-5222 | 0.0598 | 0.0000 | 0.1806 | 0.0000 | 0.0000 | 0.0634 | 0.0062 | 0.0709 | 0.0000 | 0.1333 | 0.0000 | 0.0399 | 0.0000 | 0.1928 | 0.1272 | 0.0950 | 0.0000 | 0.0000 | 0.0307 | 0.0000 | 0.0000 | 0.0000 |
| MB-5097 | 0.0000 | 0.0068 | 0.0116 | 0.1639 | 0.0000 | 0.0000 | 0.0437 | 0.0640 | 0.0234 | 0.0000 | 0.0000 | 0.0938 | 0.0353 | 0.1346 | 0.1036 | 0.1571 | 0.0133 | 0.0000 | 0.1487 | 0.0000 | 0.0000 | 0.0000 |
| MB-5338 | 0.0613 | 0.0000 | 0.0969 | 0.0760 | 0.0000 | 0.0000 | 0.0298 | 0.1144 | 0.0312 | 0.0108 | 0.0000 | 0.0655 | 0.0000 | 0.2256 | 0.1134 | 0.1290 | 0.0000 | 0.0000 | 0.0462 | 0.0000 | 0.0000 | 0.0000 |
| MB-5315 | 0.0768 | 0.0021 | 0.0000 | 0.0745 | 0.1193 | 0.0000 | 0.0409 | 0.0899 | 0.0485 | 0.0237 | 0.0000 | 0.0540 | 0.0389 | 0.1051 | 0.1643 | 0.0673 | 0.0345 | 0.0000 | 0.0601 | 0.0000 | 0.0000 | 0.0000 |
| MB-5195 | 0.0212 | 0.0000 | 0.0560 | 0.0495 | 0.0000 | 0.0670 | 0.0000 | 0.0681 | 0.0453 | 0.0872 | 0.0000 | 0.0454 | 0.0000 | 0.2938 | 0.0736 | 0.1409 | 0.0000 | 0.0000 | 0.0519 | 0.0000 | 0.0000 | 0.0000 |
| MB-5226 | 0.0385 | 0.0000 | 0.0071 | 0.0805 | 0.0000 | 0.0000 | 0.0000 | 0.0791 | 0.0184 | 0.0298 | 0.0000 | 0.0279 | 0.0000 | 0.3813 | 0.0495 | 0.2580 | 0.0000 | 0.0000 | 0.0285 | 0.0000 | 0.0000 | 0.0013 |
| MB-5232 | 0.0047 | 0.0000 | 0.0235 | 0.1150 | 0.0000 | 0.0776 | 0.0000 | 0.1371 | 0.0684 | 0.0647 | 0.0000 | 0.0251 | 0.0349 | 0.1749 | 0.1315 | 0.0999 | 0.0131 | 0.0000 | 0.0296 | 0.0000 | 0.0000 | 0.0000 |
| MB-5160 | 0.0000 | 0.0358 | 0.0000 | 0.1294 | 0.0000 | 0.0000 | 0.0000 | 0.0000 | 0.0000 | 0.0942 | 0.0000 | 0.0288 | 0.0345 | 0.0000 | 0.0786 | 0.4383 | 0.0165 | 0.0000 | 0.1393 | 0.0000 | 0.0044 | 0.0000 |
| MB-5126 | 0.0789 | 0.0000 | 0.0129 | 0.0612 | 0.0877 | 0.0443 | 0.0000 | 0.0471 | 0.0725 | 0.0568 | 0.0000 | 0.0620 | 0.0000 | 0.2096 | 0.1416 | 0.1009 | 0.0000 | 0.0000 | 0.0245 | 0.0000 | 0.0000 | 0.0000 |
| MB-5124 | 0.0000 | 0.0030 | 0.0000 | 0.1385 | 0.0000 | 0.0000 | 0.0000 | 0.0701 | 0.0000 | 0.0420 | 0.0000 | 0.0686 | 0.0000 | 0.2464 | 0.0838 | 0.1579 | 0.0000 | 0.0000 | 0.1898 | 0.0000 | 0.0000 | 0.0000 |
| MB-4855 | 0.0381 | 0.0000 | 0.0000 | 0.1694 | 0.0000 | 0.0000 | 0.0000 | 0.0843 | 0.0779 | 0.0000 | 0.0000 | 0.0514 | 0.0000 | 0.0901 | 0.0469 | 0.3953 | 0.0063 | 0.0000 | 0.0269 | 0.0000 | 0.0000 | 0.0133 |
| MB-5279 | 0.0000 | 0.1185 | 0.0000 | 0.0545 | 0.0523 | 0.0000 | 0.0000 | 0.0881 | 0.0785 | 0.1143 | 0.0000 | 0.0000 | 0.0291 | 0.0557 | 0.0582 | 0.2492 | 0.0432 | 0.0000 | 0.0000 | 0.0416 | 0.0169 | 0.0000 |
| MB-4173 | 0.0112 | 0.0041 | 0.0000 | 0.0529 | 0.0000 | 0.0356 | 0.0000 | 0.1024 | 0.0541 | 0.0000 | 0.0000 | 0.0516 | 0.0000 | 0.2496 | 0.0401 | 0.2819 | 0.0000 | 0.0000 | 0.1063 | 0.0000 | 0.0000 | 0.0102 |
| MB-5145 | 0.0207 | 0.1062 | 0.0091 | 0.0432 | 0.0622 | 0.0382 | 0.0000 | 0.1614 | 0.0000 | 0.0389 | 0.0000 | 0.0151 | 0.0220 | 0.2071 | 0.1076 | 0.1261 | 0.0000 | 0.0000 | 0.0424 | 0.0000 | 0.0000 | 0.0000 |
| MB-4944 | 0.0000 | 0.0535 | 0.0000 | 0.0000 | 0.0610 | 0.0923 | 0.0000 | 0.0000 | 0.0000 | 0.0000 | 0.0694 | 0.0000 | 0.0000 | 0.2073 | 0.0346 | 0.3473 | 0.0015 | 0.0014 | 0.0356 | 0.0674 | 0.0000 | 0.0287 |
| MB-4961 | 0.0000 | 0.0000 | 0.0412 | 0.1041 | 0.0000 | 0.1006 | 0.0000 | 0.0897 | 0.0564 | 0.0000 | 0.0000 | 0.0842 | 0.0226 | 0.0000 | 0.1043 | 0.2797 | 0.0076 | 0.0000 | 0.1096 | 0.0000 | 0.0000 | 0.0000 |
| MB-5121 | 0.0592 | 0.0000 | 0.0000 | 0.0177 | 0.0000 | 0.1756 | 0.0000 | 0.0438 | 0.0182 | 0.0000 | 0.0000 | 0.0000 | 0.0130 | 0.0000 | 0.0743 | 0.2383 | 0.0010 | 0.0000 | 0.3591 | 0.0000 | 0.0000 | 0.0000 |
| MB-4956 | 0.0174 | 0.0000 | 0.0103 | 0.1833 | 0.0000 | 0.0167 | 0.0000 | 0.0768 | 0.0294 | 0.0157 | 0.0000 | 0.0796 | 0.0291 | 0.1438 | 0.1463 | 0.1130 | 0.0099 | 0.0000 | 0.1286 | 0.0000 | 0.0000 | 0.0000 |
| MB-4886 | 0.0344 | 0.0000 | 0.0000 | 0.0519 | 0.0008 | 0.0000 | 0.0000 | 0.1053 | 0.0400 | 0.0450 | 0.0069 | 0.0190 | 0.0386 | 0.3179 | 0.1827 | 0.1103 | 0.0000 | 0.0000 | 0.0471 | 0.0000 | 0.0000 | 0.0000 |
| MB-4950 | 0.0000 | 0.0121 | 0.0754 | 0.0173 | 0.0000 | 0.0000 | 0.0000 | 0.1045 | 0.0806 | 0.0067 | 0.0196 | 0.0001 | 0.0000 | 0.3732 | 0.1203 | 0.1440 | 0.0000 | 0.0000 | 0.0461 | 0.0000 | 0.0000 | 0.0000 |
| MB-4965 | 0.0132 | 0.0000 | 0.0104 | 0.1089 | 0.0000 | 0.0000 | 0.0000 | 0.1193 | 0.0591 | 0.0009 | 0.0000 | 0.0449 | 0.0012 | 0.2693 | 0.1390 | 0.1698 | 0.0000 | 0.0003 | 0.0637 | 0.0000 | 0.0000 | 0.0000 |
| MB-4962 | 0.0075 | 0.0015 | 0.0000 | 0.1266 | 0.0000 | 0.0000 | 0.0000 | 0.1091 | 0.0389 | 0.0401 | 0.0000 | 0.0473 | 0.0000 | 0.2586 | 0.1010 | 0.1857 | 0.0000 | 0.0000 | 0.0837 | 0.0000 | 0.0000 | 0.0000 |
| MB-4952 | 0.0271 | 0.0000 | 0.1144 | 0.0778 | 0.0127 | 0.0268 | 0.0354 | 0.0231 | 0.0048 | 0.0319 | 0.0000 | 0.0963 | 0.0000 | 0.2369 | 0.1323 | 0.1162 | 0.0000 | 0.0000 | 0.0644 | 0.0000 | 0.0000 | 0.0000 |
| MB-5267 | 0.0590 | 0.0000 | 0.0268 | 0.0751 | 0.0075 | 0.2504 | 0.0000 | 0.0203 | 0.0000 | 0.0000 | 0.0000 | 0.0518 | 0.0721 | 0.0102 | 0.0857 | 0.1999 | 0.0066 | 0.0000 | 0.1346 | 0.0000 | 0.0000 | 0.0000 |
| MB-5266 | 0.0000 | 0.0000 | 0.0383 | 0.0767 | 0.0000 | 0.0000 | 0.1043 | 0.1507 | 0.0403 | 0.0840 | 0.0000 | 0.0474 | 0.0000 | 0.1288 | 0.1099 | 0.1185 | 0.0135 | 0.0000 | 0.0875 | 0.0000 | 0.0000 | 0.0000 |
| MB-5396 | 0.0000 | 0.0000 | 0.0000 | 0.0303 | 0.0000 | 0.0436 | 0.0000 | 0.1233 | 0.0325 | 0.0857 | 0.0000 | 0.0359 | 0.0173 | 0.3240 | 0.1075 | 0.1905 | 0.0000 | 0.0000 | 0.0094 | 0.0000 | 0.0000 | 0.0000 |
| MB-4938 | 0.0546 | 0.0000 | 0.0317 | 0.0000 | 0.0000 | 0.1010 | 0.0000 | 0.0545 | 0.0000 | 0.0889 | 0.0000 | 0.0000 | 0.0086 | 0.3368 | 0.0703 | 0.1348 | 0.0000 | 0.0000 | 0.0000 | 0.1187 | 0.0000 | 0.0000 |
| MB-5351 | 0.0457 | 0.0000 | 0.0754 | 0.0014 | 0.0061 | 0.1223 | 0.0000 | 0.1106 | 0.0122 | 0.1219 | 0.0000 | 0.0356 | 0.0227 | 0.1517 | 0.0729 | 0.1255 | 0.0593 | 0.0006 | 0.0362 | 0.0000 | 0.0000 | 0.0000 |
| MB-5347 | 0.0087 | 0.0192 | 0.0000 | 0.0433 | 0.0000 | 0.0509 | 0.0000 | 0.0515 | 0.0810 | 0.0000 | 0.1416 | 0.0000 | 0.0358 | 0.2443 | 0.0709 | 0.0424 | 0.0000 | 0.0237 | 0.1869 | 0.0000 | 0.0000 | 0.0000 |
| MB-5312 | 0.0520 | 0.0000 | 0.0380 | 0.0307 | 0.0004 | 0.1057 | 0.0192 | 0.0802 | 0.0054 | 0.0449 | 0.0000 | 0.0556 | 0.0221 | 0.1779 | 0.1922 | 0.0962 | 0.0061 | 0.0000 | 0.0734 | 0.0000 | 0.0000 | 0.0000 |
| MB-5311 | 0.0610 | 0.0000 | 0.0193 | 0.0000 | 0.0000 | 0.0000 | 0.0000 | 0.0403 | 0.0331 | 0.0996 | 0.0000 | 0.0000 | 0.0000 | 0.3324 | 0.0739 | 0.3047 | 0.0000 | 0.0000 | 0.0000 | 0.0356 | 0.0000 | 0.0000 |
| MB-5284 | 0.0284 | 0.0000 | 0.3477 | 0.0528 | 0.0000 | 0.0494 | 0.0000 | 0.1078 | 0.0054 | 0.0759 | 0.0000 | 0.0152 | 0.0159 | 0.0319 | 0.0398 | 0.1672 | 0.0000 | 0.0000 | 0.0519 | 0.0105 | 0.0000 | 0.0000 |
| MB-4148 | 0.0269 | 0.0000 | 0.0000 | 0.1399 | 0.0085 | 0.0000 | 0.0092 | 0.1069 | 0.0648 | 0.0866 | 0.0000 | 0.0392 | 0.0000 | 0.1434 | 0.1543 | 0.1508 | 0.0000 | 0.0000 | 0.0697 | 0.0000 | 0.0000 | 0.0000 |
| MB-5078 | 0.0000 | 0.0010 | 0.0604 | 0.0000 | 0.0000 | 0.1228 | 0.0000 | 0.1391 | 0.0372 | 0.0000 | 0.0747 | 0.0205 | 0.0097 | 0.2316 | 0.1282 | 0.1488 | 0.0000 | 0.0000 | 0.0259 | 0.0000 | 0.0000 | 0.0000 |
| MB-5088 | 0.0347 | 0.0000 | 0.0004 | 0.2132 | 0.0000 | 0.0000 | 0.0000 | 0.0544 | 0.0152 | 0.0658 | 0.0351 | 0.0000 | 0.0000 | 0.2247 | 0.0796 | 0.2117 | 0.0000 | 0.0000 | 0.0000 | 0.0652 | 0.0000 | 0.0000 |
| MB-5064 | 0.0006 | 0.0125 | 0.0000 | 0.0132 | 0.0768 | 0.0000 | 0.0000 | 0.0303 | 0.0000 | 0.0978 | 0.0000 | 0.0000 | 0.0004 | 0.3905 | 0.0343 | 0.2895 | 0.0209 | 0.0000 | 0.0000 | 0.0330 | 0.0000 | 0.0000 |
| MB-5107 | 0.0000 | 0.0009 | 0.0558 | 0.0158 | 0.0000 | 0.0003 | 0.0508 | 0.1900 | 0.0028 | 0.0199 | 0.0000 | 0.0725 | 0.0472 | 0.2322 | 0.0827 | 0.1207 | 0.0118 | 0.0000 | 0.0966 | 0.0000 | 0.0000 | 0.0000 |
| MB-5066 | 0.0132 | 0.0000 | 0.0067 | 0.0471 | 0.0110 | 0.0240 | 0.0000 | 0.0808 | 0.0559 | 0.0112 | 0.0000 | 0.0317 | 0.0588 | 0.1501 | 0.1112 | 0.2183 | 0.0240 | 0.0000 | 0.1561 | 0.0000 | 0.0000 | 0.0000 |
| MB-5068 | 0.1104 | 0.0000 | 0.0090 | 0.0456 | 0.0000 | 0.0000 | 0.0000 | 0.1608 | 0.0658 | 0.0000 | 0.0683 | 0.0000 | 0.0561 | 0.0000 | 0.0747 | 0.2616 | 0.0083 | 0.0000 | 0.1043 | 0.0352 | 0.0000 | 0.0000 |
| MB-5061 | 0.0212 | 0.0192 | 0.0000 | 0.0068 | 0.0345 | 0.0180 | 0.0000 | 0.0366 | 0.0562 | 0.0762 | 0.0000 | 0.0267 | 0.0000 | 0.3361 | 0.1161 | 0.1681 | 0.0278 | 0.0000 | 0.0564 | 0.0000 | 0.0000 | 0.0000 |
| MB-5070 | 0.0000 | 0.0000 | 0.3593 | 0.0110 | 0.0000 | 0.0969 | 0.0026 | 0.0657 | 0.0000 | 0.0466 | 0.0000 | 0.0354 | 0.0000 | 0.1124 | 0.1054 | 0.1423 | 0.0056 | 0.0000 | 0.0168 | 0.0000 | 0.0000 | 0.0000 |
| MB-5059 | 0.0000 | 0.0000 | 0.0000 | 0.1810 | 0.0000 | 0.0851 | 0.0000 | 0.0378 | 0.0000 | 0.0209 | 0.0000 | 0.0356 | 0.0107 | 0.2440 | 0.0664 | 0.2701 | 0.0000 | 0.0000 | 0.0483 | 0.0000 | 0.0000 | 0.0000 |
| MB-4266 | 0.0111 | 0.0000 | 0.0271 | 0.0801 | 0.0000 | 0.0000 | 0.0000 | 0.1128 | 0.0397 | 0.0397 | 0.0000 | 0.0966 | 0.0000 | 0.2878 | 0.0897 | 0.0768 | 0.0000 | 0.0000 | 0.1384 | 0.0000 | 0.0000 | 0.0000 |
| MB-4276 | 0.0085 | 0.0000 | 0.0012 | 0.1088 | 0.0000 | 0.0000 | 0.0000 | 0.0769 | 0.0519 | 0.0000 | 0.0234 | 0.0733 | 0.0203 | 0.2995 | 0.1070 | 0.1204 | 0.0049 | 0.0000 | 0.1039 | 0.0000 | 0.0000 | 0.0000 |
| MB-4771 | 0.0284 | 0.0000 | 0.1942 | 0.0344 | 0.0394 | 0.0828 | 0.0000 | 0.0135 | 0.0498 | 0.0875 | 0.0000 | 0.0144 | 0.0000 | 0.0889 | 0.1253 | 0.1771 | 0.0000 | 0.0000 | 0.0645 | 0.0000 | 0.0000 | 0.0000 |
| MB-4739 | 0.0195 | 0.0000 | 0.0099 | 0.0732 | 0.0000 | 0.0314 | 0.0000 | 0.0272 | 0.0312 | 0.0000 | 0.0055 | 0.0089 | 0.0386 | 0.3317 | 0.0906 | 0.1754 | 0.0081 | 0.0000 | 0.1490 | 0.0000 | 0.0000 | 0.0000 |
| MB-5331 | 0.0196 | 0.0000 | 0.0319 | 0.0000 | 0.0000 | 0.0656 | 0.0000 | 0.0699 | 0.0492 | 0.0316 | 0.0083 | 0.0000 | 0.0000 | 0.3552 | 0.1759 | 0.1566 | 0.0000 | 0.0000 | 0.0363 | 0.0000 | 0.0000 | 0.0000 |
| MB-4743 | 0.0214 | 0.0000 | 0.1384 | 0.1545 | 0.0177 | 0.1490 | 0.0000 | 0.0435 | 0.0000 | 0.0000 | 0.0000 | 0.0657 | 0.0101 | 0.0510 | 0.0608 | 0.0992 | 0.0009 | 0.0000 | 0.1879 | 0.0000 | 0.0000 | 0.0000 |
| MB-4785 | 0.0282 | 0.0000 | 0.0000 | 0.0000 | 0.0592 | 0.0000 | 0.0000 | 0.0552 | 0.0498 | 0.0000 | 0.0752 | 0.0000 | 0.0000 | 0.3891 | 0.0256 | 0.2264 | 0.0000 | 0.0000 | 0.0000 | 0.0913 | 0.0000 | 0.0000 |
| MB-4778 | 0.0000 | 0.0565 | 0.1477 | 0.0495 | 0.0000 | 0.0678 | 0.0000 | 0.0645 | 0.0200 | 0.0443 | 0.0000 | 0.0684 | 0.0000 | 0.1499 | 0.0546 | 0.1978 | 0.0045 | 0.0000 | 0.0745 | 0.0000 | 0.0000 | 0.0000 |
| MB-4763 | 0.0087 | 0.0000 | 0.1886 | 0.0000 | 0.0000 | 0.0928 | 0.0000 | 0.1039 | 0.0035 | 0.0173 | 0.0091 | 0.0349 | 0.0000 | 0.2435 | 0.1271 | 0.0999 | 0.0078 | 0.0000 | 0.0630 | 0.0000 | 0.0000 | 0.0000 |
| MB-4779 | 0.0314 | 0.0000 | 0.0000 | 0.0929 | 0.0000 | 0.0000 | 0.0000 | 0.0590 | 0.0136 | 0.0364 | 0.0000 | 0.0477 | 0.0041 | 0.3470 | 0.0736 | 0.1897 | 0.0000 | 0.0000 | 0.1045 | 0.0000 | 0.0000 | 0.0000 |
| MB-4849 | 0.0272 | 0.0000 | 0.0000 | 0.1187 | 0.0343 | 0.0000 | 0.0000 | 0.0085 | 0.0481 | 0.0153 | 0.0000 | 0.1224 | 0.0000 | 0.0182 | 0.0723 | 0.2915 | 0.0000 | 0.0000 | 0.2088 | 0.0000 | 0.0000 | 0.0348 |
| MB-4787 | 0.0081 | 0.0082 | 0.0000 | 0.0543 | 0.0000 | 0.0524 | 0.0000 | 0.0862 | 0.0680 | 0.0000 | 0.0000 | 0.1152 | 0.0834 | 0.2217 | 0.1260 | 0.0654 | 0.0218 | 0.0000 | 0.0892 | 0.0000 | 0.0000 | 0.0000 |
| MB-4767 | 0.0000 | 0.0299 | 0.0000 | 0.0625 | 0.0000 | 0.0472 | 0.0270 | 0.0721 | 0.0184 | 0.0922 | 0.0000 | 0.0584 | 0.0125 | 0.2894 | 0.0890 | 0.0676 | 0.0000 | 0.0000 | 0.1339 | 0.0000 | 0.0000 | 0.0000 |
| MB-4784 | 0.0000 | 0.0543 | 0.0000 | 0.0000 | 0.0259 | 0.1309 | 0.0000 | 0.0431 | 0.0387 | 0.0000 | 0.0932 | 0.0000 | 0.0409 | 0.2211 | 0.1543 | 0.1025 | 0.0381 | 0.0000 | 0.0000 | 0.0571 | 0.0000 | 0.0000 |
| MB-5043 | 0.0156 | 0.0000 | 0.0212 | 0.0796 | 0.0000 | 0.0000 | 0.0000 | 0.1071 | 0.0976 | 0.0859 | 0.0000 | 0.0420 | 0.0000 | 0.2916 | 0.0988 | 0.0788 | 0.0000 | 0.0000 | 0.0817 | 0.0000 | 0.0000 | 0.0000 |
| MB-4992 | 0.0000 | 0.0131 | 0.0000 | 0.1749 | 0.0000 | 0.1397 | 0.0000 | 0.0767 | 0.0000 | 0.0000 | 0.0000 | 0.0323 | 0.0044 | 0.0000 | 0.1970 | 0.2788 | 0.0040 | 0.0000 | 0.0792 | 0.0000 | 0.0000 | 0.0000 |
| MB-5020 | 0.0671 | 0.0000 | 0.0000 | 0.0000 | 0.0386 | 0.0000 | 0.0000 | 0.0346 | 0.0321 | 0.1364 | 0.0000 | 0.0000 | 0.0000 | 0.3233 | 0.0642 | 0.2283 | 0.0000 | 0.0000 | 0.0730 | 0.0024 | 0.0000 | 0.0000 |
| MB-5019 | 0.0168 | 0.0000 | 0.0090 | 0.0643 | 0.0000 | 0.0155 | 0.0000 | 0.1234 | 0.0357 | 0.0655 | 0.0000 | 0.0477 | 0.0507 | 0.2294 | 0.1159 | 0.1169 | 0.0000 | 0.0000 | 0.1093 | 0.0000 | 0.0000 | 0.0000 |
| MB-5015 | 0.1718 | 0.0000 | 0.0000 | 0.0604 | 0.0538 | 0.0000 | 0.0000 | 0.0308 | 0.0603 | 0.0000 | 0.0251 | 0.0110 | 0.0191 | 0.3206 | 0.0686 | 0.1014 | 0.0000 | 0.0000 | 0.0000 | 0.0770 | 0.0000 | 0.0000 |
| MB-5048 | 0.0298 | 0.0000 | 0.0000 | 0.0534 | 0.0000 | 0.0482 | 0.0000 | 0.0807 | 0.0586 | 0.0510 | 0.0000 | 0.0485 | 0.0047 | 0.2358 | 0.1447 | 0.0969 | 0.0185 | 0.0000 | 0.1290 | 0.0000 | 0.0000 | 0.0000 |
| MB-5035 | 0.0077 | 0.0000 | 0.0000 | 0.1866 | 0.0000 | 0.0000 | 0.0000 | 0.0767 | 0.0317 | 0.0302 | 0.0000 | 0.1126 | 0.0357 | 0.0748 | 0.1827 | 0.1336 | 0.0172 | 0.0000 | 0.1104 | 0.0000 | 0.0000 | 0.0000 |
| MB-5398 | 0.0000 | 0.0000 | 0.0379 | 0.0536 | 0.0000 | 0.0809 | 0.0000 | 0.1187 | 0.0129 | 0.0129 | 0.0000 | 0.0898 | 0.0067 | 0.0000 | 0.1515 | 0.2257 | 0.0457 | 0.0000 | 0.1637 | 0.0000 | 0.0000 | 0.0000 |
| MB-5377 | 0.0159 | 0.0000 | 0.0031 | 0.0381 | 0.0000 | 0.0262 | 0.0000 | 0.0768 | 0.0326 | 0.0346 | 0.0000 | 0.0021 | 0.0000 | 0.3626 | 0.0760 | 0.2634 | 0.0000 | 0.0000 | 0.0657 | 0.0000 | 0.0000 | 0.0027 |
| MB-5291 | 0.0349 | 0.0000 | 0.0090 | 0.0538 | 0.0000 | 0.0625 | 0.0000 | 0.0527 | 0.0240 | 0.0000 | 0.0000 | 0.0480 | 0.0181 | 0.0000 | 0.0375 | 0.3444 | 0.0000 | 0.0000 | 0.3151 | 0.0000 | 0.0000 | 0.0000 |
| MB-5403 | 0.0012 | 0.0000 | 0.0475 | 0.0783 | 0.0000 | 0.0420 | 0.0000 | 0.0747 | 0.0000 | 0.1131 | 0.0000 | 0.0842 | 0.0088 | 0.0173 | 0.1024 | 0.2578 | 0.0341 | 0.0000 | 0.1385 | 0.0000 | 0.0000 | 0.0000 |
| MB-5224 | 0.0220 | 0.0000 | 0.1407 | 0.1609 | 0.0000 | 0.0000 | 0.0000 | 0.0711 | 0.0426 | 0.0547 | 0.0000 | 0.0779 | 0.0000 | 0.0283 | 0.1367 | 0.1434 | 0.0150 | 0.0000 | 0.1066 | 0.0000 | 0.0000 | 0.0000 |
| MB-5369 | 0.0452 | 0.0000 | 0.0364 | 0.0000 | 0.0000 | 0.0359 | 0.0000 | 0.0606 | 0.0466 | 0.0395 | 0.0000 | 0.0000 | 0.0000 | 0.3462 | 0.0232 | 0.3353 | 0.0000 | 0.0000 | 0.0194 | 0.0000 | 0.0000 | 0.0115 |
| MB-5404 | 0.0000 | 0.0030 | 0.0097 | 0.0654 | 0.0000 | 0.1175 | 0.0000 | 0.0844 | 0.0319 | 0.0000 | 0.0000 | 0.0163 | 0.0000 | 0.0655 | 0.0321 | 0.4378 | 0.0636 | 0.0000 | 0.0202 | 0.0402 | 0.0000 | 0.0124 |
| MB-5397 | 0.0109 | 0.0000 | 0.0112 | 0.0160 | 0.0000 | 0.0294 | 0.0000 | 0.0352 | 0.0147 | 0.0000 | 0.0000 | 0.0494 | 0.0000 | 0.0790 | 0.0259 | 0.2070 | 0.0000 | 0.0079 | 0.5134 | 0.0000 | 0.0000 | 0.0000 |
| MB-5378 | 0.0178 | 0.0000 | 0.0038 | 0.0078 | 0.0000 | 0.0107 | 0.0000 | 0.0231 | 0.0722 | 0.0302 | 0.0000 | 0.0229 | 0.0000 | 0.4651 | 0.1315 | 0.1896 | 0.0000 | 0.0000 | 0.0000 | 0.0253 | 0.0000 | 0.0000 |
| MB-5388 | 0.0000 | 0.0000 | 0.0238 | 0.0292 | 0.0000 | 0.0299 | 0.0000 | 0.1425 | 0.0303 | 0.0846 | 0.0000 | 0.0832 | 0.0221 | 0.2560 | 0.1441 | 0.1123 | 0.0130 | 0.0000 | 0.0290 | 0.0000 | 0.0000 | 0.0000 |
| MB-5395 | 0.0311 | 0.0000 | 0.0461 | 0.0347 | 0.0000 | 0.0858 | 0.0000 | 0.1075 | 0.0000 | 0.0000 | 0.0000 | 0.0688 | 0.0087 | 0.0359 | 0.0250 | 0.0456 | 0.0050 | 0.0000 | 0.5058 | 0.0000 | 0.0000 | 0.0000 |
| MB-5365 | 0.0360 | 0.0000 | 0.0040 | 0.0848 | 0.0000 | 0.0671 | 0.0000 | 0.1002 | 0.0136 | 0.0000 | 0.0000 | 0.0624 | 0.0130 | 0.1052 | 0.1113 | 0.2599 | 0.0196 | 0.0000 | 0.1228 | 0.0000 | 0.0000 | 0.0000 |
| MB-5389 | 0.0000 | 0.0234 | 0.0000 | 0.0166 | 0.0576 | 0.0000 | 0.0144 | 0.0103 | 0.0050 | 0.0250 | 0.1393 | 0.0000 | 0.0000 | 0.1998 | 0.0385 | 0.4053 | 0.0048 | 0.0000 | 0.0599 | 0.0000 | 0.0000 | 0.0000 |
| MB-5393 | 0.0292 | 0.0000 | 0.0000 | 0.0000 | 0.1630 | 0.0000 | 0.0000 | 0.0014 | 0.0105 | 0.0072 | 0.0668 | 0.0000 | 0.0000 | 0.1509 | 0.0363 | 0.5221 | 0.0000 | 0.0000 | 0.0109 | 0.0000 | 0.0016 | 0.0000 |
| MB-5361 | 0.0000 | 0.0000 | 0.0399 | 0.1078 | 0.0000 | 0.0000 | 0.0000 | 0.0943 | 0.0283 | 0.0235 | 0.0000 | 0.0648 | 0.0162 | 0.3453 | 0.0717 | 0.1479 | 0.0000 | 0.0000 | 0.0586 | 0.0015 | 0.0000 | 0.0000 |
| MB-5392 | 0.0140 | 0.0000 | 0.0499 | 0.0548 | 0.0194 | 0.0975 | 0.0041 | 0.0274 | 0.0397 | 0.0297 | 0.0000 | 0.0551 | 0.0000 | 0.2026 | 0.2445 | 0.1030 | 0.0125 | 0.0000 | 0.0458 | 0.0000 | 0.0000 | 0.0000 |
| MB-5147 | 0.0176 | 0.0000 | 0.0098 | 0.0355 | 0.0000 | 0.0708 | 0.0000 | 0.0502 | 0.0356 | 0.0059 | 0.0000 | 0.0430 | 0.0000 | 0.2259 | 0.0525 | 0.4001 | 0.0000 | 0.0000 | 0.0522 | 0.0000 | 0.0000 | 0.0011 |
| MB-4140 | 0.0016 | 0.0000 | 0.0085 | 0.0741 | 0.0000 | 0.0000 | 0.0000 | 0.0686 | 0.0729 | 0.0128 | 0.0000 | 0.0365 | 0.0000 | 0.1973 | 0.0142 | 0.4337 | 0.0000 | 0.0000 | 0.0733 | 0.0000 | 0.0000 | 0.0065 |
| MB-5138 | 0.0563 | 0.0000 | 0.0771 | 0.0530 | 0.0455 | 0.0000 | 0.0296 | 0.0801 | 0.0023 | 0.0156 | 0.0000 | 0.0769 | 0.0000 | 0.1891 | 0.2108 | 0.0747 | 0.0000 | 0.0000 | 0.0890 | 0.0000 | 0.0000 | 0.0000 |
| MB-5123 | 0.0013 | 0.0060 | 0.0355 | 0.0878 | 0.0000 | 0.1297 | 0.0000 | 0.0631 | 0.0435 | 0.0000 | 0.0000 | 0.1072 | 0.0303 | 0.0103 | 0.1269 | 0.2228 | 0.0195 | 0.0000 | 0.1160 | 0.0000 | 0.0000 | 0.0000 |
| MB-5065 | 0.0305 | 0.0000 | 0.1018 | 0.0949 | 0.0000 | 0.0000 | 0.0128 | 0.0429 | 0.0000 | 0.1578 | 0.0000 | 0.0368 | 0.0000 | 0.2198 | 0.1526 | 0.1149 | 0.0000 | 0.0000 | 0.0352 | 0.0000 | 0.0000 | 0.0000 |
| MB-4127 | 0.0000 | 0.0000 | 0.0000 | 0.0000 | 0.0000 | 0.0518 | 0.1198 | 0.0366 | 0.0000 | 0.0000 | 0.0950 | 0.0000 | 0.0000 | 0.3537 | 0.0361 | 0.2240 | 0.0000 | 0.0000 | 0.0000 | 0.0831 | 0.0000 | 0.0000 |
| MB-5058 | 0.0191 | 0.0137 | 0.0000 | 0.0141 | 0.0000 | 0.0000 | 0.0000 | 0.1172 | 0.0271 | 0.1253 | 0.0000 | 0.0559 | 0.0000 | 0.2881 | 0.1196 | 0.1768 | 0.0106 | 0.0000 | 0.0325 | 0.0000 | 0.0000 | 0.0000 |
| MB-5050 | 0.0340 | 0.0000 | 0.0000 | 0.0000 | 0.0000 | 0.0549 | 0.0000 | 0.0595 | 0.0240 | 0.1041 | 0.0297 | 0.0000 | 0.0418 | 0.1340 | 0.0846 | 0.1242 | 0.0082 | 0.0000 | 0.3011 | 0.0000 | 0.0000 | 0.0000 |
| MB-5027 | 0.0064 | 0.0000 | 0.1315 | 0.0593 | 0.0000 | 0.1132 | 0.0000 | 0.0823 | 0.0000 | 0.0503 | 0.0000 | 0.0074 | 0.0182 | 0.3225 | 0.0623 | 0.1110 | 0.0000 | 0.0000 | 0.0354 | 0.0000 | 0.0000 | 0.0000 |
| MB-5060 | 0.0085 | 0.0000 | 0.0000 | 0.0000 | 0.0000 | 0.0000 | 0.0000 | 0.0665 | 0.0314 | 0.0320 | 0.0186 | 0.0000 | 0.0000 | 0.4033 | 0.0555 | 0.3573 | 0.0000 | 0.0000 | 0.0269 | 0.0000 | 0.0000 | 0.0000 |
| MB-5062 | 0.0179 | 0.0000 | 0.0982 | 0.0000 | 0.0000 | 0.0777 | 0.0000 | 0.0941 | 0.0187 | 0.0329 | 0.0154 | 0.0362 | 0.0417 | 0.2676 | 0.0924 | 0.1721 | 0.0000 | 0.0000 | 0.0351 | 0.0000 | 0.0000 | 0.0000 |
| MB-5383 | 0.0026 | 0.0127 | 0.0000 | 0.0040 | 0.0181 | 0.0000 | 0.0000 | 0.0294 | 0.0000 | 0.0223 | 0.0635 | 0.0000 | 0.0000 | 0.5115 | 0.0148 | 0.2627 | 0.0000 | 0.0000 | 0.0000 | 0.0583 | 0.0000 | 0.0000 |
| MB-5101 | 0.0109 | 0.0000 | 0.0241 | 0.1225 | 0.0000 | 0.0588 | 0.0143 | 0.0559 | 0.0314 | 0.0000 | 0.0000 | 0.0701 | 0.0370 | 0.0606 | 0.1817 | 0.1884 | 0.0000 | 0.0000 | 0.1443 | 0.0000 | 0.0000 | 0.0000 |
| MB-5258 | 0.0051 | 0.0000 | 0.0003 | 0.0294 | 0.0000 | 0.0733 | 0.0000 | 0.0154 | 0.0018 | 0.0000 | 0.0117 | 0.0085 | 0.0148 | 0.5665 | 0.1066 | 0.0968 | 0.0000 | 0.0000 | 0.0000 | 0.0698 | 0.0000 | 0.0000 |
| MB-5268 | 0.0000 | 0.0000 | 0.0227 | 0.0478 | 0.0000 | 0.1308 | 0.0000 | 0.1167 | 0.0052 | 0.0000 | 0.0000 | 0.0381 | 0.0401 | 0.2118 | 0.0857 | 0.1580 | 0.0064 | 0.0000 | 0.1368 | 0.0000 | 0.0000 | 0.0000 |
| MB-5261 | 0.0000 | 0.0000 | 0.0313 | 0.1159 | 0.0016 | 0.0000 | 0.0000 | 0.0643 | 0.0326 | 0.0435 | 0.0000 | 0.0298 | 0.0000 | 0.2876 | 0.0636 | 0.2657 | 0.0103 | 0.0000 | 0.0538 | 0.0000 | 0.0000 | 0.0000 |
| MB-5272 | 0.0576 | 0.0000 | 0.0327 | 0.0610 | 0.0600 | 0.0034 | 0.0149 | 0.0856 | 0.0034 | 0.1290 | 0.0000 | 0.0820 | 0.0143 | 0.1623 | 0.1177 | 0.0821 | 0.0000 | 0.0000 | 0.0940 | 0.0000 | 0.0000 | 0.0000 |
| MB-5264 | 0.0627 | 0.0000 | 0.0021 | 0.0744 | 0.0000 | 0.0400 | 0.0000 | 0.0721 | 0.0099 | 0.0699 | 0.0000 | 0.0000 | 0.0004 | 0.3083 | 0.0583 | 0.1967 | 0.0000 | 0.0000 | 0.1052 | 0.0000 | 0.0000 | 0.0000 |
| MB-5259 | 0.0191 | 0.0000 | 0.0000 | 0.0843 | 0.0000 | 0.1048 | 0.0036 | 0.0633 | 0.0293 | 0.0727 | 0.0000 | 0.0976 | 0.0291 | 0.1903 | 0.1519 | 0.0437 | 0.0000 | 0.0000 | 0.1103 | 0.0000 | 0.0000 | 0.0000 |
| MB-5306 | 0.0000 | 0.0000 | 0.0415 | 0.0125 | 0.0000 | 0.0037 | 0.0000 | 0.0906 | 0.0652 | 0.0863 | 0.0000 | 0.0605 | 0.0000 | 0.3080 | 0.0716 | 0.1499 | 0.0000 | 0.0000 | 0.1101 | 0.0000 | 0.0000 | 0.0000 |
| MB-5270 | 0.0494 | 0.0000 | 0.0127 | 0.0556 | 0.0000 | 0.0734 | 0.0075 | 0.0430 | 0.0249 | 0.0215 | 0.0000 | 0.0029 | 0.0000 | 0.1508 | 0.1475 | 0.2689 | 0.0000 | 0.0000 | 0.1420 | 0.0000 | 0.0000 | 0.0000 |
| MB-5411 | 0.0000 | 0.0000 | 0.0145 | 0.0606 | 0.0000 | 0.0477 | 0.1157 | 0.0897 | 0.0000 | 0.0000 | 0.0000 | 0.0347 | 0.0000 | 0.2341 | 0.0661 | 0.2228 | 0.0007 | 0.0000 | 0.1133 | 0.0000 | 0.0000 | 0.0000 |
| MB-5490 | 0.0741 | 0.0000 | 0.0161 | 0.0551 | 0.0000 | 0.0000 | 0.0000 | 0.0919 | 0.0243 | 0.0000 | 0.0026 | 0.0268 | 0.0000 | 0.3812 | 0.0109 | 0.2314 | 0.0000 | 0.0000 | 0.0856 | 0.0000 | 0.0000 | 0.0000 |
| MB-5421 | 0.0285 | 0.0000 | 0.0000 | 0.0000 | 0.0000 | 0.0838 | 0.0000 | 0.0892 | 0.0237 | 0.0348 | 0.0000 | 0.0208 | 0.0008 | 0.4665 | 0.0952 | 0.1030 | 0.0146 | 0.0046 | 0.0345 | 0.0000 | 0.0000 | 0.0000 |
| MB-5348 | 0.0040 | 0.0100 | 0.0578 | 0.0484 | 0.0083 | 0.0000 | 0.0000 | 0.1032 | 0.0358 | 0.0762 | 0.0000 | 0.1246 | 0.0000 | 0.2193 | 0.2112 | 0.0695 | 0.0000 | 0.0000 | 0.0315 | 0.0000 | 0.0000 | 0.0000 |
| MB-5429 | 0.0013 | 0.0000 | 0.0135 | 0.0000 | 0.0695 | 0.0000 | 0.0000 | 0.0426 | 0.0437 | 0.1128 | 0.0423 | 0.0000 | 0.0057 | 0.3506 | 0.0426 | 0.1720 | 0.0000 | 0.0000 | 0.0000 | 0.0964 | 0.0070 | 0.0000 |
| MB-5405 | 0.0337 | 0.0152 | 0.0000 | 0.1996 | 0.0707 | 0.0000 | 0.0000 | 0.0887 | 0.0485 | 0.1875 | 0.0000 | 0.1073 | 0.0000 | 0.0692 | 0.0693 | 0.0519 | 0.0000 | 0.0000 | 0.0584 | 0.0000 | 0.0000 | 0.0000 |
| MB-5414 | 0.0000 | 0.0094 | 0.0061 | 0.1241 | 0.0000 | 0.0000 | 0.0350 | 0.1057 | 0.0207 | 0.0134 | 0.0000 | 0.0719 | 0.0075 | 0.1809 | 0.1193 | 0.2461 | 0.0312 | 0.0000 | 0.0286 | 0.0000 | 0.0000 | 0.0000 |
| MB-5489 | 0.0118 | 0.0000 | 0.0392 | 0.0245 | 0.0000 | 0.2048 | 0.0000 | 0.0410 | 0.0000 | 0.0049 | 0.0000 | 0.0243 | 0.0520 | 0.1104 | 0.1017 | 0.1106 | 0.0112 | 0.0000 | 0.2638 | 0.0000 | 0.0000 | 0.0000 |
| MB-5417 | 0.0011 | 0.0000 | 0.0059 | 0.0784 | 0.0000 | 0.0074 | 0.0000 | 0.1206 | 0.0277 | 0.1149 | 0.0000 | 0.1018 | 0.0000 | 0.2252 | 0.1744 | 0.0883 | 0.0000 | 0.0000 | 0.0542 | 0.0000 | 0.0000 | 0.0000 |
| MB-5481 | 0.0449 | 0.0000 | 0.0216 | 0.0184 | 0.0000 | 0.0434 | 0.0000 | 0.0478 | 0.0557 | 0.0368 | 0.0000 | 0.0139 | 0.0000 | 0.4060 | 0.0517 | 0.2346 | 0.0000 | 0.0000 | 0.0177 | 0.0075 | 0.0000 | 0.0000 |
| MB-5409 | 0.0068 | 0.0000 | 0.0997 | 0.0413 | 0.0064 | 0.0244 | 0.0544 | 0.0767 | 0.0000 | 0.0723 | 0.0000 | 0.0450 | 0.0000 | 0.3040 | 0.1506 | 0.0545 | 0.0051 | 0.0000 | 0.0588 | 0.0000 | 0.0000 | 0.0000 |
| MB-5299 | 0.0243 | 0.0000 | 0.0404 | 0.0000 | 0.0088 | 0.0751 | 0.0000 | 0.1219 | 0.0438 | 0.0454 | 0.0000 | 0.0585 | 0.0000 | 0.2717 | 0.1324 | 0.1540 | 0.0000 | 0.0049 | 0.0187 | 0.0000 | 0.0000 | 0.0000 |
| MB-5408 | 0.0447 | 0.0000 | 0.0000 | 0.1502 | 0.0000 | 0.0007 | 0.0671 | 0.0595 | 0.0311 | 0.0310 | 0.0000 | 0.0747 | 0.0104 | 0.2032 | 0.0987 | 0.1282 | 0.0106 | 0.0000 | 0.0898 | 0.0000 | 0.0000 | 0.0000 |
| MB-5475 | 0.0030 | 0.0000 | 0.0273 | 0.0038 | 0.0000 | 0.0972 | 0.0000 | 0.0679 | 0.0343 | 0.0432 | 0.0000 | 0.0515 | 0.0000 | 0.0715 | 0.0662 | 0.4857 | 0.0057 | 0.0000 | 0.0000 | 0.0427 | 0.0000 | 0.0000 |
| MB-5505 | 0.0128 | 0.0054 | 0.0000 | 0.0000 | 0.0000 | 0.0190 | 0.0000 | 0.1126 | 0.0461 | 0.0938 | 0.0000 | 0.0000 | 0.0000 | 0.4143 | 0.1178 | 0.1555 | 0.0000 | 0.0000 | 0.0226 | 0.0000 | 0.0000 | 0.0000 |
| MB-5100 | 0.0135 | 0.0081 | 0.0000 | 0.0912 | 0.0894 | 0.0649 | 0.0308 | 0.0945 | 0.0045 | 0.0000 | 0.0000 | 0.0811 | 0.0145 | 0.0999 | 0.2437 | 0.0703 | 0.0000 | 0.0000 | 0.0935 | 0.0000 | 0.0000 | 0.0000 |
| MB-5502 | 0.0685 | 0.0000 | 0.0061 | 0.0197 | 0.0298 | 0.0257 | 0.0000 | 0.0491 | 0.0345 | 0.0533 | 0.0000 | 0.0269 | 0.0000 | 0.3378 | 0.0699 | 0.2123 | 0.0000 | 0.0179 | 0.0485 | 0.0000 | 0.0000 | 0.0000 |
| MB-5485 | 0.0000 | 0.0000 | 0.0075 | 0.1219 | 0.0000 | 0.0116 | 0.0136 | 0.2050 | 0.0000 | 0.0000 | 0.0000 | 0.0735 | 0.0000 | 0.1535 | 0.1423 | 0.1570 | 0.0053 | 0.0000 | 0.1087 | 0.0000 | 0.0000 | 0.0000 |
| MB-4293 | 0.0000 | 0.0487 | 0.0000 | 0.0197 | 0.0000 | 0.0269 | 0.0000 | 0.0755 | 0.0418 | 0.0000 | 0.0022 | 0.0369 | 0.0000 | 0.1603 | 0.0357 | 0.1766 | 0.0000 | 0.0000 | 0.3756 | 0.0000 | 0.0000 | 0.0000 |
| MB-4289 | 0.0000 | 0.0115 | 0.0240 | 0.0000 | 0.0473 | 0.0050 | 0.0000 | 0.0667 | 0.0393 | 0.0568 | 0.0000 | 0.0242 | 0.0000 | 0.4820 | 0.0374 | 0.1059 | 0.0000 | 0.0000 | 0.1000 | 0.0000 | 0.0000 | 0.0000 |
| MB-5293 | 0.0000 | 0.0000 | 0.0028 | 0.1573 | 0.0000 | 0.0000 | 0.0000 | 0.0769 | 0.0399 | 0.0785 | 0.0000 | 0.0503 | 0.0000 | 0.3331 | 0.0214 | 0.1070 | 0.0000 | 0.0000 | 0.1328 | 0.0000 | 0.0000 | 0.0000 |
| MB-5381 | 0.0000 | 0.0058 | 0.0577 | 0.0137 | 0.0000 | 0.0118 | 0.0096 | 0.1030 | 0.0951 | 0.0000 | 0.0229 | 0.0133 | 0.0051 | 0.2616 | 0.1618 | 0.1030 | 0.0368 | 0.0000 | 0.0988 | 0.0000 | 0.0000 | 0.0000 |
| MB-4834 | 0.0305 | 0.0000 | 0.0547 | 0.0000 | 0.0000 | 0.0267 | 0.0000 | 0.0789 | 0.0277 | 0.0808 | 0.0000 | 0.0313 | 0.0000 | 0.2675 | 0.0910 | 0.2049 | 0.0022 | 0.0000 | 0.1038 | 0.0000 | 0.0000 | 0.0000 |
| MB-5498 | 0.0000 | 0.0000 | 0.0000 | 0.0870 | 0.0000 | 0.0602 | 0.0000 | 0.0401 | 0.0015 | 0.0142 | 0.0000 | 0.0198 | 0.0000 | 0.3292 | 0.1833 | 0.1801 | 0.0000 | 0.0000 | 0.0846 | 0.0000 | 0.0000 | 0.0000 |
| MB-4749 | 0.0390 | 0.0000 | 0.0000 | 0.0251 | 0.0000 | 0.0677 | 0.0000 | 0.0700 | 0.0483 | 0.0325 | 0.0000 | 0.0405 | 0.0000 | 0.3192 | 0.1298 | 0.1499 | 0.0000 | 0.0000 | 0.0781 | 0.0000 | 0.0000 | 0.0000 |
| MB-4818 | 0.0000 | 0.0242 | 0.0000 | 0.0561 | 0.0000 | 0.0116 | 0.0000 | 0.0651 | 0.0432 | 0.0036 | 0.0014 | 0.0000 | 0.0000 | 0.3074 | 0.0463 | 0.4166 | 0.0000 | 0.0000 | 0.0244 | 0.0000 | 0.0000 | 0.0000 |
| MB-4805 | 0.0377 | 0.0000 | 0.0021 | 0.0000 | 0.0000 | 0.0000 | 0.0000 | 0.0642 | 0.0397 | 0.0000 | 0.0000 | 0.0294 | 0.0000 | 0.5240 | 0.0165 | 0.1211 | 0.0000 | 0.0000 | 0.1342 | 0.0312 | 0.0000 | 0.0000 |
| MB-4809 | 0.0067 | 0.0000 | 0.0876 | 0.0339 | 0.0847 | 0.0212 | 0.0286 | 0.0861 | 0.0000 | 0.0000 | 0.0338 | 0.0235 | 0.0465 | 0.3324 | 0.1153 | 0.0718 | 0.0000 | 0.0020 | 0.0259 | 0.0000 | 0.0000 | 0.0000 |
| MB-5418 | 0.0542 | 0.0000 | 0.0092 | 0.0648 | 0.0000 | 0.1283 | 0.0000 | 0.1031 | 0.0241 | 0.0000 | 0.0000 | 0.0477 | 0.0000 | 0.0451 | 0.0859 | 0.2519 | 0.0000 | 0.0000 | 0.1856 | 0.0000 | 0.0000 | 0.0000 |
| MB-5441 | 0.0000 | 0.0217 | 0.0311 | 0.1612 | 0.0000 | 0.0786 | 0.0000 | 0.1444 | 0.0958 | 0.0318 | 0.0000 | 0.0547 | 0.0164 | 0.0651 | 0.0857 | 0.1216 | 0.0051 | 0.0203 | 0.0664 | 0.0000 | 0.0000 | 0.0000 |
| MB-5424 | 0.0097 | 0.0000 | 0.0065 | 0.0259 | 0.0727 | 0.1728 | 0.0000 | 0.1115 | 0.0000 | 0.0048 | 0.0000 | 0.0851 | 0.0356 | 0.1468 | 0.1625 | 0.0623 | 0.0080 | 0.0000 | 0.0959 | 0.0000 | 0.0000 | 0.0000 |
| MB-5296 | 0.0125 | 0.0000 | 0.0088 | 0.1980 | 0.0245 | 0.0352 | 0.0208 | 0.0551 | 0.0180 | 0.0155 | 0.0000 | 0.0800 | 0.0157 | 0.2530 | 0.1467 | 0.0370 | 0.0080 | 0.0000 | 0.0713 | 0.0000 | 0.0000 | 0.0000 |
| MB-5446 | 0.0067 | 0.0000 | 0.0220 | 0.0000 | 0.0000 | 0.1492 | 0.0000 | 0.0475 | 0.0614 | 0.0000 | 0.0000 | 0.0522 | 0.0000 | 0.3359 | 0.1039 | 0.1637 | 0.0000 | 0.0000 | 0.0375 | 0.0200 | 0.0000 | 0.0000 |
| MB-5093 | 0.0119 | 0.0000 | 0.0124 | 0.0445 | 0.0023 | 0.0442 | 0.0000 | 0.0071 | 0.0182 | 0.0000 | 0.0000 | 0.0225 | 0.0000 | 0.4260 | 0.0246 | 0.3035 | 0.0000 | 0.0000 | 0.0730 | 0.0097 | 0.0000 | 0.0000 |
| MB-5350 | 0.0166 | 0.0049 | 0.0000 | 0.0616 | 0.0000 | 0.0799 | 0.0000 | 0.0552 | 0.0403 | 0.1066 | 0.0000 | 0.0441 | 0.0238 | 0.1729 | 0.1097 | 0.1644 | 0.0000 | 0.0000 | 0.0931 | 0.0272 | 0.0000 | 0.0000 |
| MB-5230 | 0.0000 | 0.0306 | 0.0000 | 0.1756 | 0.0000 | 0.0000 | 0.0000 | 0.0000 | 0.0040 | 0.1042 | 0.0000 | 0.1028 | 0.0286 | 0.0568 | 0.0988 | 0.0749 | 0.0204 | 0.0000 | 0.3033 | 0.0000 | 0.0000 | 0.0000 |
| MB-5206 | 0.0580 | 0.0000 | 0.0414 | 0.0949 | 0.0000 | 0.0042 | 0.0000 | 0.0379 | 0.0375 | 0.0000 | 0.0000 | 0.0000 | 0.0000 | 0.2313 | 0.0500 | 0.3392 | 0.0000 | 0.0000 | 0.0934 | 0.0000 | 0.0000 | 0.0122 |
| MB-4687 | 0.0135 | 0.0000 | 0.0068 | 0.2437 | 0.0891 | 0.0000 | 0.0000 | 0.0535 | 0.0000 | 0.1025 | 0.0000 | 0.0813 | 0.0000 | 0.1268 | 0.1086 | 0.1073 | 0.0000 | 0.0000 | 0.0653 | 0.0000 | 0.0016 | 0.0000 |
| MB-5433 | 0.0580 | 0.0000 | 0.0143 | 0.0095 | 0.0000 | 0.1565 | 0.0000 | 0.0383 | 0.0002 | 0.0000 | 0.0000 | 0.0570 | 0.0142 | 0.0586 | 0.0708 | 0.1512 | 0.0059 | 0.0000 | 0.3656 | 0.0000 | 0.0000 | 0.0000 |
| MB-5442 | 0.0138 | 0.0000 | 0.0000 | 0.0018 | 0.0000 | 0.0000 | 0.0562 | 0.1030 | 0.0376 | 0.0160 | 0.0518 | 0.0000 | 0.0089 | 0.3963 | 0.1157 | 0.1658 | 0.0000 | 0.0000 | 0.0331 | 0.0000 | 0.0000 | 0.0000 |
| MB-5427 | 0.1515 | 0.0000 | 0.0168 | 0.1637 | 0.0000 | 0.0000 | 0.0000 | 0.1268 | 0.0121 | 0.0000 | 0.0000 | 0.0047 | 0.0000 | 0.3721 | 0.0921 | 0.0513 | 0.0000 | 0.0000 | 0.0070 | 0.0000 | 0.0000 | 0.0019 |
| MB-5491 | 0.0275 | 0.0022 | 0.0000 | 0.0259 | 0.0117 | 0.0000 | 0.0000 | 0.1012 | 0.0634 | 0.0124 | 0.0000 | 0.0250 | 0.0246 | 0.4269 | 0.0430 | 0.1105 | 0.0000 | 0.0000 | 0.0000 | 0.1255 | 0.0000 | 0.0000 |
| MB-5493 | 0.0756 | 0.0000 | 0.0218 | 0.0452 | 0.0000 | 0.0835 | 0.0000 | 0.1014 | 0.0000 | 0.0000 | 0.0000 | 0.0693 | 0.0506 | 0.0184 | 0.0599 | 0.1987 | 0.0000 | 0.0456 | 0.2298 | 0.0000 | 0.0000 | 0.0000 |
| MB-5451 | 0.0000 | 0.0000 | 0.0254 | 0.0239 | 0.0000 | 0.1753 | 0.0000 | 0.0971 | 0.0000 | 0.0464 | 0.0000 | 0.0454 | 0.0000 | 0.1524 | 0.1132 | 0.2067 | 0.0228 | 0.0000 | 0.0915 | 0.0000 | 0.0000 | 0.0000 |
| MB-5454 | 0.0009 | 0.0000 | 0.0627 | 0.1669 | 0.0420 | 0.0692 | 0.0199 | 0.0326 | 0.0000 | 0.0843 | 0.0000 | 0.0948 | 0.0194 | 0.1007 | 0.1471 | 0.0284 | 0.0236 | 0.0000 | 0.1074 | 0.0000 | 0.0000 | 0.0000 |
| MB-5360 | 0.0513 | 0.0000 | 0.0000 | 0.0460 | 0.0703 | 0.0000 | 0.0000 | 0.0394 | 0.0546 | 0.0387 | 0.0425 | 0.0000 | 0.0423 | 0.0406 | 0.0471 | 0.4341 | 0.0000 | 0.0230 | 0.0012 | 0.0691 | 0.0000 | 0.0000 |
| MB-5229 | 0.0187 | 0.0000 | 0.0132 | 0.0767 | 0.0222 | 0.0754 | 0.0185 | 0.0562 | 0.0000 | 0.0234 | 0.0000 | 0.0442 | 0.0201 | 0.1654 | 0.1994 | 0.1274 | 0.0000 | 0.0000 | 0.1392 | 0.0000 | 0.0000 | 0.0000 |
| MB-4827 | 0.0410 | 0.0000 | 0.0000 | 0.0472 | 0.0000 | 0.0200 | 0.0000 | 0.0811 | 0.0039 | 0.0545 | 0.0000 | 0.0226 | 0.0159 | 0.2309 | 0.0459 | 0.2651 | 0.0000 | 0.0000 | 0.1719 | 0.0000 | 0.0000 | 0.0000 |
| MB-5455 | 0.0466 | 0.0000 | 0.0000 | 0.0328 | 0.1348 | 0.0000 | 0.0543 | 0.0774 | 0.0000 | 0.0836 | 0.0000 | 0.0638 | 0.0011 | 0.1362 | 0.1880 | 0.0745 | 0.0000 | 0.0000 | 0.1069 | 0.0000 | 0.0000 | 0.0000 |
| MB-5370 | 0.0000 | 0.0914 | 0.0000 | 0.0080 | 0.0891 | 0.0000 | 0.0000 | 0.0162 | 0.0394 | 0.0718 | 0.0317 | 0.0000 | 0.0000 | 0.3501 | 0.0118 | 0.2392 | 0.0000 | 0.0000 | 0.0363 | 0.0149 | 0.0000 | 0.0000 |
| MB-5290 | 0.0000 | 0.0294 | 0.0004 | 0.0252 | 0.0202 | 0.0843 | 0.0000 | 0.0000 | 0.1514 | 0.0000 | 0.0000 | 0.0689 | 0.0263 | 0.1671 | 0.0323 | 0.0852 | 0.0228 | 0.0064 | 0.2801 | 0.0000 | 0.0000 | 0.0000 |
| MB-5366 | 0.0000 | 0.0045 | 0.0199 | 0.0720 | 0.0000 | 0.0000 | 0.0000 | 0.1001 | 0.0528 | 0.0714 | 0.0324 | 0.0000 | 0.0000 | 0.4100 | 0.0479 | 0.1800 | 0.0000 | 0.0000 | 0.0038 | 0.0052 | 0.0000 | 0.0000 |
| MB-5382 | 0.0297 | 0.0000 | 0.0361 | 0.0753 | 0.0000 | 0.0348 | 0.0000 | 0.0876 | 0.0579 | 0.0330 | 0.0000 | 0.0946 | 0.0312 | 0.1725 | 0.0602 | 0.1464 | 0.0000 | 0.0000 | 0.1408 | 0.0000 | 0.0000 | 0.0000 |
| MB-5402 | 0.0302 | 0.0000 | 0.0000 | 0.2742 | 0.0000 | 0.0000 | 0.0000 | 0.0797 | 0.0177 | 0.0170 | 0.0000 | 0.0621 | 0.0143 | 0.0545 | 0.0989 | 0.2368 | 0.0474 | 0.0000 | 0.0606 | 0.0066 | 0.0000 | 0.0000 |
| MB-5288 | 0.0692 | 0.0000 | 0.0420 | 0.0956 | 0.0056 | 0.0000 | 0.0000 | 0.0535 | 0.0487 | 0.0888 | 0.0000 | 0.0932 | 0.0000 | 0.2113 | 0.1432 | 0.0381 | 0.0049 | 0.0000 | 0.1059 | 0.0000 | 0.0000 | 0.0000 |
| MB-5384 | 0.1012 | 0.0000 | 0.0000 | 0.0000 | 0.0202 | 0.0736 | 0.0000 | 0.0000 | 0.0246 | 0.0075 | 0.0566 | 0.0000 | 0.0000 | 0.3544 | 0.1323 | 0.1714 | 0.0000 | 0.0000 | 0.0133 | 0.0384 | 0.0000 | 0.0064 |
| MB-5358 | 0.1168 | 0.0000 | 0.0205 | 0.0407 | 0.0000 | 0.1054 | 0.0000 | 0.0581 | 0.0156 | 0.0000 | 0.0000 | 0.0804 | 0.0211 | 0.0000 | 0.2291 | 0.1729 | 0.0198 | 0.0000 | 0.1170 | 0.0000 | 0.0000 | 0.0027 |
| MB-4357 | 0.0844 | 0.0000 | 0.1457 | 0.2367 | 0.0492 | 0.0271 | 0.0000 | 0.0612 | 0.0638 | 0.0000 | 0.0000 | 0.0799 | 0.0000 | 0.0861 | 0.0663 | 0.0157 | 0.0108 | 0.0000 | 0.0732 | 0.0000 | 0.0000 | 0.0000 |
| MB-4991 | 0.0189 | 0.0000 | 0.0047 | 0.0125 | 0.0000 | 0.0521 | 0.0000 | 0.0478 | 0.0427 | 0.0797 | 0.0000 | 0.0177 | 0.0000 | 0.4445 | 0.0709 | 0.1404 | 0.0000 | 0.0000 | 0.0682 | 0.0000 | 0.0000 | 0.0000 |
| MB-5401 | 0.0146 | 0.0000 | 0.0000 | 0.0270 | 0.0214 | 0.0466 | 0.0000 | 0.0000 | 0.0714 | 0.0000 | 0.1070 | 0.0000 | 0.0000 | 0.2285 | 0.1422 | 0.1805 | 0.0000 | 0.0000 | 0.1505 | 0.0103 | 0.0000 | 0.0000 |
| MB-5390 | 0.0450 | 0.0000 | 0.0039 | 0.0478 | 0.0000 | 0.0092 | 0.0213 | 0.0775 | 0.0066 | 0.0000 | 0.0314 | 0.0330 | 0.0852 | 0.0606 | 0.1051 | 0.1632 | 0.0000 | 0.2692 | 0.0410 | 0.0000 | 0.0000 | 0.0000 |
| MB-5412 | 0.0083 | 0.0000 | 0.0140 | 0.0895 | 0.0000 | 0.1285 | 0.0000 | 0.0194 | 0.0191 | 0.0332 | 0.0000 | 0.0012 | 0.0000 | 0.3412 | 0.0679 | 0.1773 | 0.0033 | 0.0000 | 0.0970 | 0.0000 | 0.0000 | 0.0000 |
| MB-5193 | 0.0558 | 0.0000 | 0.0012 | 0.0397 | 0.0000 | 0.0196 | 0.0000 | 0.0508 | 0.0176 | 0.0000 | 0.0000 | 0.0400 | 0.0413 | 0.2024 | 0.0112 | 0.4618 | 0.0000 | 0.0000 | 0.0000 | 0.0537 | 0.0000 | 0.0051 |
| MB-5182 | 0.0257 | 0.0000 | 0.0435 | 0.0219 | 0.0000 | 0.1518 | 0.0000 | 0.0533 | 0.0342 | 0.0000 | 0.0301 | 0.0284 | 0.0203 | 0.1352 | 0.0911 | 0.2074 | 0.0000 | 0.0000 | 0.1569 | 0.0000 | 0.0000 | 0.0000 |
| MB-5157 | 0.0097 | 0.0000 | 0.0000 | 0.2080 | 0.0000 | 0.0000 | 0.0054 | 0.1119 | 0.0545 | 0.0771 | 0.0000 | 0.0382 | 0.0000 | 0.2045 | 0.1450 | 0.0867 | 0.0000 | 0.0000 | 0.0590 | 0.0000 | 0.0000 | 0.0000 |
| MB-5205 | 0.0130 | 0.0000 | 0.0000 | 0.0782 | 0.0000 | 0.0000 | 0.0450 | 0.1596 | 0.0449 | 0.0000 | 0.0166 | 0.0863 | 0.0026 | 0.1909 | 0.1690 | 0.1291 | 0.0185 | 0.0062 | 0.0402 | 0.0000 | 0.0000 | 0.0000 |
| MB-5161 | 0.0045 | 0.0000 | 0.2140 | 0.0075 | 0.0000 | 0.0794 | 0.0000 | 0.0873 | 0.0000 | 0.0913 | 0.0000 | 0.0371 | 0.0437 | 0.0844 | 0.0597 | 0.1307 | 0.0209 | 0.0000 | 0.1237 | 0.0159 | 0.0000 | 0.0000 |
| MB-5292 | 0.0093 | 0.0000 | 0.0044 | 0.0000 | 0.0000 | 0.0000 | 0.0000 | 0.0594 | 0.0747 | 0.0344 | 0.0042 | 0.0000 | 0.0000 | 0.3906 | 0.0788 | 0.2756 | 0.0000 | 0.0000 | 0.0686 | 0.0000 | 0.0000 | 0.0000 |
| MB-5211 | 0.0159 | 0.0000 | 0.0094 | 0.0216 | 0.0000 | 0.0296 | 0.0018 | 0.0769 | 0.0594 | 0.0052 | 0.0000 | 0.0412 | 0.0000 | 0.2278 | 0.0680 | 0.2551 | 0.0033 | 0.0000 | 0.1848 | 0.0000 | 0.0000 | 0.0000 |
| MB-5218 | 0.0190 | 0.0000 | 0.0000 | 0.0411 | 0.0000 | 0.1222 | 0.0000 | 0.0455 | 0.0236 | 0.0350 | 0.0000 | 0.0410 | 0.1061 | 0.2103 | 0.1343 | 0.1151 | 0.0154 | 0.0000 | 0.0912 | 0.0000 | 0.0000 | 0.0000 |
| MB-5209 | 0.0000 | 0.0129 | 0.1856 | 0.1809 | 0.0000 | 0.0000 | 0.0844 | 0.0980 | 0.0657 | 0.0291 | 0.0000 | 0.0659 | 0.0129 | 0.1981 | 0.0572 | 0.0055 | 0.0000 | 0.0000 | 0.0032 | 0.0006 | 0.0000 | 0.0000 |
| MB-5227 | 0.0269 | 0.0000 | 0.0082 | 0.0891 | 0.0000 | 0.0391 | 0.0000 | 0.0412 | 0.0260 | 0.0000 | 0.0000 | 0.0366 | 0.0653 | 0.2679 | 0.1291 | 0.1334 | 0.0130 | 0.0000 | 0.1241 | 0.0000 | 0.0000 | 0.0000 |
| MB-5213 | 0.0086 | 0.0000 | 0.0785 | 0.0000 | 0.0000 | 0.1312 | 0.0000 | 0.0739 | 0.0002 | 0.0532 | 0.0000 | 0.0885 | 0.0000 | 0.2030 | 0.1585 | 0.1036 | 0.0538 | 0.0000 | 0.0469 | 0.0000 | 0.0000 | 0.0000 |
| MB-5166 | 0.0000 | 0.0232 | 0.0563 | 0.1872 | 0.0000 | 0.0100 | 0.0000 | 0.0977 | 0.0000 | 0.0145 | 0.0000 | 0.0691 | 0.0000 | 0.1993 | 0.0951 | 0.1861 | 0.0098 | 0.0000 | 0.0517 | 0.0000 | 0.0000 | 0.0000 |
| MB-5163 | 0.0000 | 0.0000 | 0.0000 | 0.0257 | 0.0000 | 0.0000 | 0.0000 | 0.0476 | 0.0662 | 0.0495 | 0.0000 | 0.0588 | 0.0246 | 0.2565 | 0.0272 | 0.4028 | 0.0000 | 0.0000 | 0.0412 | 0.0000 | 0.0000 | 0.0000 |
| MB-5188 | 0.0202 | 0.0000 | 0.0000 | 0.0616 | 0.0452 | 0.0578 | 0.0178 | 0.1004 | 0.0000 | 0.0011 | 0.0000 | 0.0484 | 0.0797 | 0.0980 | 0.1828 | 0.1832 | 0.0892 | 0.0000 | 0.0144 | 0.0000 | 0.0000 | 0.0000 |
| MB-5143 | 0.0247 | 0.0000 | 0.0000 | 0.0558 | 0.0201 | 0.1010 | 0.0000 | 0.0779 | 0.0271 | 0.0000 | 0.0000 | 0.0776 | 0.0526 | 0.1392 | 0.0816 | 0.2236 | 0.0000 | 0.0000 | 0.1188 | 0.0000 | 0.0000 | 0.0000 |
| MB-5329 | 0.0000 | 0.0000 | 0.0059 | 0.1118 | 0.0000 | 0.0113 | 0.0000 | 0.0919 | 0.0129 | 0.0000 | 0.0003 | 0.0254 | 0.0570 | 0.2635 | 0.1393 | 0.1401 | 0.0038 | 0.0000 | 0.1368 | 0.0000 | 0.0000 | 0.0000 |
| MB-5322 | 0.0000 | 0.0156 | 0.0000 | 0.0346 | 0.0565 | 0.0000 | 0.0000 | 0.1115 | 0.0414 | 0.0654 | 0.0000 | 0.0936 | 0.0368 | 0.0453 | 0.1237 | 0.1881 | 0.0071 | 0.0000 | 0.1804 | 0.0000 | 0.0000 | 0.0000 |
| MB-5310 | 0.0000 | 0.0000 | 0.0532 | 0.0990 | 0.0092 | 0.0304 | 0.0000 | 0.0668 | 0.0199 | 0.0000 | 0.0000 | 0.0560 | 0.1606 | 0.0527 | 0.1204 | 0.2096 | 0.0048 | 0.0000 | 0.1176 | 0.0000 | 0.0000 | 0.0000 |
| MB-4000 | 0.0000 | 0.0184 | 0.0606 | 0.0493 | 0.0290 | 0.0000 | 0.0000 | 0.0656 | 0.0222 | 0.0361 | 0.0000 | 0.0393 | 0.0038 | 0.2127 | 0.1073 | 0.2472 | 0.0000 | 0.0000 | 0.1084 | 0.0000 | 0.0000 | 0.0000 |
| MB-5240 | 0.0517 | 0.0000 | 0.0158 | 0.1409 | 0.0000 | 0.0773 | 0.0000 | 0.0429 | 0.0000 | 0.0000 | 0.0000 | 0.0628 | 0.0443 | 0.0374 | 0.0713 | 0.2204 | 0.0501 | 0.0000 | 0.1613 | 0.0216 | 0.0000 | 0.0023 |
| MB-5228 | 0.0000 | 0.0000 | 0.0031 | 0.1030 | 0.0000 | 0.0371 | 0.0000 | 0.0807 | 0.0620 | 0.0343 | 0.0000 | 0.0405 | 0.0177 | 0.3239 | 0.0775 | 0.1944 | 0.0048 | 0.0000 | 0.0211 | 0.0000 | 0.0000 | 0.0000 |
| MB-5117 | 0.0533 | 0.0000 | 0.0227 | 0.1299 | 0.0000 | 0.0773 | 0.0000 | 0.0840 | 0.0066 | 0.0234 | 0.0000 | 0.0422 | 0.0159 | 0.1889 | 0.1060 | 0.1811 | 0.0000 | 0.0000 | 0.0686 | 0.0000 | 0.0000 | 0.0000 |
| MB-5176 | 0.0000 | 0.0000 | 0.0716 | 0.2035 | 0.0189 | 0.0000 | 0.0580 | 0.0435 | 0.0000 | 0.0098 | 0.0000 | 0.1069 | 0.0258 | 0.0655 | 0.0954 | 0.1622 | 0.0393 | 0.0000 | 0.0995 | 0.0000 | 0.0000 | 0.0000 |
| MB-5215 | 0.0000 | 0.0549 | 0.0000 | 0.0840 | 0.0000 | 0.0217 | 0.0000 | 0.1570 | 0.1149 | 0.0000 | 0.0062 | 0.0347 | 0.0000 | 0.2091 | 0.0154 | 0.0992 | 0.0000 | 0.0000 | 0.1760 | 0.0000 | 0.0000 | 0.0269 |
| MB-5223 | 0.0421 | 0.0000 | 0.0027 | 0.1639 | 0.0000 | 0.0882 | 0.0405 | 0.0574 | 0.0000 | 0.0445 | 0.0000 | 0.0220 | 0.0479 | 0.1136 | 0.1451 | 0.1207 | 0.0000 | 0.0000 | 0.1115 | 0.0000 | 0.0000 | 0.0000 |
| MB-5179 | 0.0012 | 0.0000 | 0.0324 | 0.0171 | 0.0000 | 0.1092 | 0.0000 | 0.0566 | 0.0000 | 0.0541 | 0.0000 | 0.0366 | 0.0378 | 0.0421 | 0.2151 | 0.1285 | 0.0383 | 0.0000 | 0.2311 | 0.0000 | 0.0000 | 0.0000 |
| MB-5330 | 0.0157 | 0.0000 | 0.0009 | 0.0969 | 0.0000 | 0.0000 | 0.0000 | 0.0698 | 0.0258 | 0.0454 | 0.0000 | 0.0421 | 0.0000 | 0.3539 | 0.0380 | 0.2481 | 0.0000 | 0.0000 | 0.0497 | 0.0138 | 0.0000 | 0.0000 |
| MB-5144 | 0.0000 | 0.0270 | 0.0000 | 0.0000 | 0.0000 | 0.0837 | 0.0000 | 0.0954 | 0.0059 | 0.0142 | 0.0000 | 0.0284 | 0.0000 | 0.3057 | 0.1700 | 0.1302 | 0.0000 | 0.0000 | 0.1396 | 0.0000 | 0.0000 | 0.0000 |
| MB-5196 | 0.0006 | 0.0000 | 0.0113 | 0.0350 | 0.0000 | 0.0147 | 0.0000 | 0.0608 | 0.0388 | 0.0273 | 0.0000 | 0.0519 | 0.0000 | 0.2799 | 0.0631 | 0.1894 | 0.0002 | 0.0000 | 0.2269 | 0.0000 | 0.0000 | 0.0000 |
| MB-5200 | 0.0242 | 0.0000 | 0.0032 | 0.0844 | 0.0000 | 0.0000 | 0.0000 | 0.0700 | 0.0537 | 0.0691 | 0.0000 | 0.0082 | 0.0000 | 0.4092 | 0.0362 | 0.2036 | 0.0026 | 0.0000 | 0.0316 | 0.0000 | 0.0000 | 0.0039 |
| MB-5583 | 0.0118 | 0.0000 | 0.1022 | 0.0695 | 0.0000 | 0.1261 | 0.0000 | 0.0613 | 0.0039 | 0.0153 | 0.0000 | 0.0540 | 0.0053 | 0.1284 | 0.1115 | 0.1897 | 0.0000 | 0.0000 | 0.1212 | 0.0000 | 0.0000 | 0.0000 |
| MB-5601 | 0.0000 | 0.0000 | 0.0132 | 0.0344 | 0.0000 | 0.0936 | 0.0000 | 0.0567 | 0.0208 | 0.1008 | 0.0000 | 0.1160 | 0.0194 | 0.1267 | 0.1384 | 0.2007 | 0.0000 | 0.0000 | 0.0792 | 0.0000 | 0.0000 | 0.0000 |
| MB-5632 | 0.0173 | 0.0038 | 0.0000 | 0.0000 | 0.0000 | 0.0000 | 0.0000 | 0.0648 | 0.0227 | 0.1211 | 0.0000 | 0.0293 | 0.0000 | 0.4529 | 0.0469 | 0.1961 | 0.0000 | 0.0000 | 0.0377 | 0.0075 | 0.0000 | 0.0000 |
| MB-5616 | 0.0000 | 0.0042 | 0.1373 | 0.0954 | 0.0332 | 0.0188 | 0.0000 | 0.1297 | 0.0000 | 0.0000 | 0.0000 | 0.1384 | 0.0323 | 0.1564 | 0.1477 | 0.0755 | 0.0000 | 0.0077 | 0.0233 | 0.0000 | 0.0000 | 0.0000 |
| MB-5604 | 0.0417 | 0.0000 | 0.0000 | 0.0000 | 0.0512 | 0.0679 | 0.0000 | 0.0188 | 0.0000 | 0.1045 | 0.0000 | 0.0000 | 0.0192 | 0.0573 | 0.0467 | 0.2913 | 0.0000 | 0.0000 | 0.2802 | 0.0213 | 0.0000 | 0.0000 |
| MB-5589 | 0.0154 | 0.0000 | 0.0086 | 0.0515 | 0.0000 | 0.1627 | 0.0000 | 0.0000 | 0.0000 | 0.0000 | 0.0000 | 0.0610 | 0.0202 | 0.1051 | 0.0356 | 0.2202 | 0.0000 | 0.0000 | 0.3196 | 0.0000 | 0.0000 | 0.0000 |
| MB-5582 | 0.0145 | 0.0000 | 0.0209 | 0.0651 | 0.0091 | 0.0722 | 0.0000 | 0.0555 | 0.0280 | 0.0392 | 0.0000 | 0.0394 | 0.0000 | 0.3580 | 0.0528 | 0.1350 | 0.0305 | 0.0000 | 0.0796 | 0.0000 | 0.0000 | 0.0000 |
| MB-5571 | 0.0423 | 0.0000 | 0.0218 | 0.0365 | 0.0000 | 0.1018 | 0.0000 | 0.0648 | 0.0000 | 0.0000 | 0.0136 | 0.0029 | 0.0000 | 0.1620 | 0.0755 | 0.2308 | 0.0042 | 0.0000 | 0.2340 | 0.0000 | 0.0000 | 0.0101 |
| MB-5596 | 0.0273 | 0.0000 | 0.2015 | 0.0847 | 0.0000 | 0.0900 | 0.0000 | 0.0108 | 0.0013 | 0.0124 | 0.0000 | 0.0380 | 0.0045 | 0.0000 | 0.0700 | 0.3701 | 0.0196 | 0.0000 | 0.0174 | 0.0476 | 0.0000 | 0.0049 |
| MB-5590 | 0.0000 | 0.0097 | 0.0633 | 0.0534 | 0.0000 | 0.0748 | 0.0000 | 0.0631 | 0.0581 | 0.0883 | 0.0000 | 0.0119 | 0.0000 | 0.2317 | 0.0903 | 0.2276 | 0.0000 | 0.0000 | 0.0279 | 0.0000 | 0.0000 | 0.0000 |
| MB-5599 | 0.0051 | 0.0120 | 0.0922 | 0.0302 | 0.0000 | 0.0992 | 0.0000 | 0.0638 | 0.0854 | 0.0000 | 0.0000 | 0.0454 | 0.0272 | 0.1684 | 0.0456 | 0.1476 | 0.0000 | 0.0000 | 0.1163 | 0.0615 | 0.0000 | 0.0000 |
| MB-5629 | 0.0026 | 0.0028 | 0.0000 | 0.1082 | 0.0064 | 0.0000 | 0.0000 | 0.0831 | 0.0228 | 0.0000 | 0.0036 | 0.0498 | 0.0000 | 0.1797 | 0.1690 | 0.1398 | 0.0043 | 0.0000 | 0.2279 | 0.0000 | 0.0000 | 0.0000 |
| MB-5579 | 0.0062 | 0.0000 | 0.1285 | 0.0327 | 0.0421 | 0.0048 | 0.0029 | 0.0423 | 0.0227 | 0.1099 | 0.0000 | 0.0556 | 0.0000 | 0.1918 | 0.0870 | 0.1604 | 0.0119 | 0.0000 | 0.1011 | 0.0000 | 0.0000 | 0.0000 |
| MB-5623 | 0.0000 | 0.0022 | 0.0094 | 0.1018 | 0.0000 | 0.0363 | 0.0239 | 0.0790 | 0.0258 | 0.0000 | 0.0200 | 0.0306 | 0.0175 | 0.1286 | 0.1034 | 0.0758 | 0.0251 | 0.0000 | 0.3206 | 0.0000 | 0.0000 | 0.0000 |
| MB-5654 | 0.1771 | 0.0000 | 0.0000 | 0.1033 | 0.0000 | 0.0000 | 0.0000 | 0.0758 | 0.0650 | 0.0628 | 0.0000 | 0.0306 | 0.0000 | 0.1126 | 0.0452 | 0.2127 | 0.0000 | 0.0000 | 0.0392 | 0.0757 | 0.0000 | 0.0000 |
| MB-5617 | 0.0000 | 0.0000 | 0.0298 | 0.0366 | 0.0194 | 0.0831 | 0.0000 | 0.1155 | 0.0000 | 0.0005 | 0.0314 | 0.0880 | 0.0801 | 0.0367 | 0.1638 | 0.1524 | 0.0207 | 0.0000 | 0.1420 | 0.0000 | 0.0000 | 0.0000 |
| MB-5638 | 0.0000 | 0.0089 | 0.0180 | 0.0135 | 0.0000 | 0.0110 | 0.0255 | 0.0980 | 0.0480 | 0.0000 | 0.0095 | 0.0205 | 0.0516 | 0.3014 | 0.0731 | 0.2583 | 0.0000 | 0.0000 | 0.0627 | 0.0000 | 0.0000 | 0.0000 |
| MB-5635 | 0.0098 | 0.0000 | 0.0211 | 0.0000 | 0.0000 | 0.0000 | 0.0000 | 0.0377 | 0.0170 | 0.0665 | 0.0000 | 0.0102 | 0.0000 | 0.4742 | 0.0667 | 0.2053 | 0.0000 | 0.0000 | 0.0801 | 0.0000 | 0.0113 | 0.0000 |
| MB-5642 | 0.0609 | 0.0000 | 0.0698 | 0.0000 | 0.0012 | 0.0532 | 0.0000 | 0.0512 | 0.0553 | 0.0292 | 0.0000 | 0.0000 | 0.0061 | 0.1922 | 0.0940 | 0.2966 | 0.0037 | 0.0000 | 0.0000 | 0.0821 | 0.0045 | 0.0000 |
| MB-5647 | 0.1255 | 0.0519 | 0.0000 | 0.1303 | 0.0000 | 0.0000 | 0.0000 | 0.0694 | 0.0267 | 0.0000 | 0.0000 | 0.0603 | 0.0042 | 0.2170 | 0.0665 | 0.0983 | 0.0000 | 0.0000 | 0.1500 | 0.0000 | 0.0000 | 0.0000 |
| MB-4018 | 0.0000 | 0.0767 | 0.0000 | 0.2352 | 0.0000 | 0.0000 | 0.0000 | 0.0000 | 0.0370 | 0.0566 | 0.0000 | 0.0982 | 0.0187 | 0.0567 | 0.1116 | 0.1160 | 0.0316 | 0.0000 | 0.1617 | 0.0000 | 0.0000 | 0.0000 |
| MB-5584 | 0.0166 | 0.0000 | 0.0054 | 0.1124 | 0.0000 | 0.0000 | 0.0849 | 0.0993 | 0.0000 | 0.0385 | 0.0000 | 0.0980 | 0.0000 | 0.1424 | 0.1823 | 0.1433 | 0.0134 | 0.0000 | 0.0605 | 0.0003 | 0.0000 | 0.0027 |
| MB-5626 | 0.0453 | 0.0000 | 0.0000 | 0.0333 | 0.0000 | 0.1891 | 0.0000 | 0.0092 | 0.0358 | 0.0264 | 0.0000 | 0.0404 | 0.1232 | 0.0116 | 0.1114 | 0.2466 | 0.0165 | 0.0118 | 0.0937 | 0.0058 | 0.0000 | 0.0000 |
| MB-5602 | 0.0000 | 0.0396 | 0.0425 | 0.0690 | 0.0644 | 0.0000 | 0.0000 | 0.0050 | 0.0312 | 0.0930 | 0.0000 | 0.0967 | 0.0024 | 0.1792 | 0.1537 | 0.1135 | 0.0311 | 0.0000 | 0.0788 | 0.0000 | 0.0000 | 0.0000 |
| MB-5653 | 0.0088 | 0.0000 | 0.0416 | 0.0000 | 0.0000 | 0.0521 | 0.0000 | 0.0619 | 0.0240 | 0.0416 | 0.0260 | 0.0000 | 0.0000 | 0.4297 | 0.0207 | 0.2429 | 0.0000 | 0.0394 | 0.0000 | 0.0114 | 0.0000 | 0.0000 |
| MB-5576 | 0.0252 | 0.0000 | 0.0435 | 0.0421 | 0.0000 | 0.0723 | 0.0000 | 0.0599 | 0.0697 | 0.0000 | 0.0000 | 0.0215 | 0.0338 | 0.1549 | 0.3072 | 0.0899 | 0.0036 | 0.0000 | 0.0764 | 0.0000 | 0.0000 | 0.0000 |
| MB-5575 | 0.0000 | 0.0104 | 0.0123 | 0.0000 | 0.0000 | 0.1046 | 0.0000 | 0.0727 | 0.0339 | 0.0531 | 0.0000 | 0.0000 | 0.0000 | 0.1549 | 0.0822 | 0.2328 | 0.0066 | 0.0000 | 0.2165 | 0.0199 | 0.0000 | 0.0000 |
| MB-5646 | 0.0542 | 0.0000 | 0.0137 | 0.0000 | 0.0000 | 0.0779 | 0.0000 | 0.0469 | 0.1062 | 0.0249 | 0.0213 | 0.0000 | 0.0000 | 0.3094 | 0.1669 | 0.1466 | 0.0000 | 0.0000 | 0.0319 | 0.0000 | 0.0000 | 0.0000 |
| MB-5634 | 0.0060 | 0.0000 | 0.2359 | 0.0294 | 0.0085 | 0.0272 | 0.0068 | 0.0395 | 0.0238 | 0.0111 | 0.0000 | 0.0680 | 0.0048 | 0.3234 | 0.1136 | 0.0520 | 0.0000 | 0.0000 | 0.0498 | 0.0000 | 0.0000 | 0.0000 |
| MB-5591 | 0.0021 | 0.0000 | 0.0080 | 0.0081 | 0.0000 | 0.0000 | 0.0000 | 0.0972 | 0.0000 | 0.0845 | 0.0000 | 0.0022 | 0.0007 | 0.3580 | 0.1403 | 0.2383 | 0.0000 | 0.0000 | 0.0321 | 0.0286 | 0.0000 | 0.0000 |
| MB-5567 | 0.0070 | 0.0000 | 0.0097 | 0.0414 | 0.0000 | 0.0963 | 0.0000 | 0.0992 | 0.0388 | 0.0124 | 0.0079 | 0.0237 | 0.0123 | 0.3291 | 0.0623 | 0.2181 | 0.0000 | 0.0000 | 0.0354 | 0.0065 | 0.0000 | 0.0000 |
| MB-5651 | 0.0096 | 0.0000 | 0.1042 | 0.0927 | 0.0000 | 0.0208 | 0.0000 | 0.0833 | 0.0480 | 0.0623 | 0.0000 | 0.0589 | 0.0000 | 0.1914 | 0.1554 | 0.1238 | 0.0000 | 0.0123 | 0.0372 | 0.0000 | 0.0000 | 0.0000 |
| MB-5603 | 0.0157 | 0.0000 | 0.0679 | 0.0503 | 0.0000 | 0.0861 | 0.0149 | 0.0757 | 0.0460 | 0.0547 | 0.0000 | 0.0094 | 0.0212 | 0.2199 | 0.1009 | 0.1095 | 0.0395 | 0.0000 | 0.0883 | 0.0000 | 0.0000 | 0.0000 |
| MB-5622 | 0.0364 | 0.0046 | 0.0000 | 0.0000 | 0.0412 | 0.0814 | 0.0000 | 0.0673 | 0.0408 | 0.0926 | 0.0000 | 0.0052 | 0.0000 | 0.2767 | 0.1016 | 0.1137 | 0.0000 | 0.0000 | 0.0634 | 0.0752 | 0.0000 | 0.0000 |
| MB-5597 | 0.0152 | 0.0000 | 0.0000 | 0.0000 | 0.0000 | 0.0593 | 0.0000 | 0.0851 | 0.0663 | 0.0470 | 0.0000 | 0.0713 | 0.0047 | 0.0000 | 0.1081 | 0.3194 | 0.0031 | 0.0000 | 0.2182 | 0.0000 | 0.0000 | 0.0022 |
| MB-5613 | 0.0287 | 0.0000 | 0.0050 | 0.0000 | 0.0000 | 0.0317 | 0.0000 | 0.0547 | 0.0332 | 0.0063 | 0.0050 | 0.0084 | 0.0000 | 0.3756 | 0.0310 | 0.2613 | 0.0000 | 0.0000 | 0.0000 | 0.1590 | 0.0000 | 0.0000 |
| MB-5580 | 0.0000 | 0.0210 | 0.0690 | 0.1362 | 0.0000 | 0.0776 | 0.0000 | 0.0137 | 0.0741 | 0.0242 | 0.0000 | 0.0625 | 0.0569 | 0.0000 | 0.0705 | 0.2739 | 0.0108 | 0.0000 | 0.1072 | 0.0000 | 0.0000 | 0.0024 |
| MB-5645 | 0.0196 | 0.0000 | 0.0660 | 0.0516 | 0.0000 | 0.0592 | 0.0000 | 0.0533 | 0.0160 | 0.0540 | 0.0000 | 0.0702 | 0.0525 | 0.2271 | 0.1337 | 0.1305 | 0.0000 | 0.0000 | 0.0663 | 0.0000 | 0.0000 | 0.0000 |
| MB-5548 | 0.0411 | 0.0000 | 0.0002 | 0.0167 | 0.0970 | 0.0235 | 0.0000 | 0.0728 | 0.0000 | 0.0458 | 0.0000 | 0.0444 | 0.0237 | 0.1238 | 0.2461 | 0.2013 | 0.0110 | 0.0000 | 0.0526 | 0.0000 | 0.0000 | 0.0000 |
| MB-5526 | 0.0723 | 0.0000 | 0.0998 | 0.0230 | 0.0000 | 0.1276 | 0.0264 | 0.0929 | 0.0000 | 0.0768 | 0.0000 | 0.0539 | 0.0000 | 0.1586 | 0.1494 | 0.0596 | 0.0338 | 0.0000 | 0.0260 | 0.0000 | 0.0000 | 0.0000 |
| MB-5531 | 0.0021 | 0.0080 | 0.0512 | 0.0471 | 0.0084 | 0.0723 | 0.0034 | 0.0412 | 0.0192 | 0.0094 | 0.0151 | 0.0001 | 0.0196 | 0.3583 | 0.1816 | 0.1336 | 0.0114 | 0.0000 | 0.0180 | 0.0000 | 0.0000 | 0.0000 |
| MB-5468 | 0.0000 | 0.0094 | 0.0425 | 0.0380 | 0.0071 | 0.0000 | 0.0837 | 0.1177 | 0.0016 | 0.0253 | 0.0000 | 0.0499 | 0.0232 | 0.3434 | 0.0722 | 0.1272 | 0.0000 | 0.0145 | 0.0442 | 0.0000 | 0.0000 | 0.0000 |
| MB-5474 | 0.0518 | 0.0000 | 0.1378 | 0.0807 | 0.0094 | 0.1136 | 0.0382 | 0.0391 | 0.0000 | 0.0540 | 0.0000 | 0.0310 | 0.0000 | 0.1497 | 0.1416 | 0.0934 | 0.0000 | 0.0000 | 0.0598 | 0.0000 | 0.0000 | 0.0000 |
| MB-5514 | 0.0863 | 0.0000 | 0.0000 | 0.0436 | 0.0000 | 0.1451 | 0.0000 | 0.0652 | 0.0000 | 0.0000 | 0.0000 | 0.1032 | 0.0261 | 0.0000 | 0.0424 | 0.2793 | 0.0129 | 0.0000 | 0.1958 | 0.0000 | 0.0000 | 0.0000 |
| MB-5510 | 0.0161 | 0.0000 | 0.0005 | 0.0007 | 0.0000 | 0.0000 | 0.0000 | 0.0814 | 0.0317 | 0.0000 | 0.0112 | 0.0100 | 0.0000 | 0.2684 | 0.0191 | 0.4720 | 0.0024 | 0.0000 | 0.0582 | 0.0000 | 0.0000 | 0.0283 |
| MB-5473 | 0.0359 | 0.0000 | 0.0112 | 0.0625 | 0.0000 | 0.0558 | 0.0000 | 0.0470 | 0.0208 | 0.0544 | 0.0000 | 0.0684 | 0.0000 | 0.1450 | 0.0588 | 0.2896 | 0.0000 | 0.0151 | 0.1356 | 0.0000 | 0.0000 | 0.0000 |
| MB-5484 | 0.0323 | 0.0000 | 0.0000 | 0.0757 | 0.0000 | 0.0627 | 0.0000 | 0.2064 | 0.0000 | 0.0067 | 0.0000 | 0.0366 | 0.0000 | 0.0000 | 0.0800 | 0.1926 | 0.0000 | 0.0000 | 0.2423 | 0.0454 | 0.0000 | 0.0194 |
| MB-5432 | 0.0093 | 0.0000 | 0.0287 | 0.0437 | 0.0173 | 0.0000 | 0.0063 | 0.1480 | 0.0305 | 0.0000 | 0.0000 | 0.0368 | 0.0134 | 0.2546 | 0.1042 | 0.1258 | 0.0000 | 0.0000 | 0.1816 | 0.0000 | 0.0000 | 0.0000 |
| MB-5525 | 0.0507 | 0.0000 | 0.0162 | 0.0415 | 0.0000 | 0.0904 | 0.0000 | 0.0618 | 0.0043 | 0.0000 | 0.0011 | 0.0466 | 0.0000 | 0.0000 | 0.0477 | 0.4119 | 0.0295 | 0.0000 | 0.1757 | 0.0000 | 0.0000 | 0.0226 |
| MB-5562 | 0.0379 | 0.0000 | 0.0051 | 0.0564 | 0.0000 | 0.1056 | 0.0000 | 0.0590 | 0.0045 | 0.0000 | 0.0000 | 0.0244 | 0.0467 | 0.0000 | 0.0439 | 0.4059 | 0.0297 | 0.0000 | 0.1775 | 0.0000 | 0.0000 | 0.0035 |
| MB-5497 | 0.0112 | 0.0000 | 0.0220 | 0.0232 | 0.0000 | 0.1130 | 0.0000 | 0.0498 | 0.0000 | 0.0936 | 0.0000 | 0.0212 | 0.0210 | 0.0944 | 0.1077 | 0.1731 | 0.0070 | 0.0000 | 0.2629 | 0.0000 | 0.0000 | 0.0000 |
| MB-5551 | 0.0686 | 0.0000 | 0.0157 | 0.0000 | 0.0000 | 0.0000 | 0.0030 | 0.0906 | 0.0404 | 0.0259 | 0.0375 | 0.0000 | 0.0000 | 0.4950 | 0.1372 | 0.0732 | 0.0000 | 0.0000 | 0.0129 | 0.0000 | 0.0000 | 0.0000 |
| MB-5300 | 0.0477 | 0.0000 | 0.0033 | 0.0396 | 0.0000 | 0.0329 | 0.0000 | 0.0751 | 0.0000 | 0.0445 | 0.0000 | 0.0390 | 0.0000 | 0.1938 | 0.0882 | 0.4161 | 0.0010 | 0.0000 | 0.0000 | 0.0188 | 0.0000 | 0.0000 |
| MB-5560 | 0.0248 | 0.0000 | 0.0000 | 0.0544 | 0.0000 | 0.0525 | 0.0124 | 0.1130 | 0.0000 | 0.0000 | 0.0000 | 0.0383 | 0.1198 | 0.2336 | 0.0669 | 0.2044 | 0.0000 | 0.0172 | 0.0628 | 0.0000 | 0.0000 | 0.0000 |
| MB-5550 | 0.0441 | 0.0165 | 0.0000 | 0.0598 | 0.0000 | 0.0729 | 0.0000 | 0.2092 | 0.0000 | 0.0152 | 0.0000 | 0.0796 | 0.0389 | 0.0662 | 0.1534 | 0.1404 | 0.0159 | 0.0000 | 0.0881 | 0.0000 | 0.0000 | 0.0000 |
| MB-5520 | 0.0319 | 0.0000 | 0.0482 | 0.0514 | 0.0000 | 0.1267 | 0.0000 | 0.0169 | 0.0175 | 0.0061 | 0.0075 | 0.0731 | 0.0802 | 0.1464 | 0.0906 | 0.1780 | 0.0000 | 0.0000 | 0.1255 | 0.0000 | 0.0000 | 0.0000 |
| MB-5529 | 0.0236 | 0.0000 | 0.0175 | 0.0981 | 0.0451 | 0.0977 | 0.0000 | 0.0372 | 0.0166 | 0.0066 | 0.0000 | 0.0831 | 0.1034 | 0.0260 | 0.0991 | 0.2339 | 0.0213 | 0.0000 | 0.0908 | 0.0000 | 0.0000 | 0.0000 |
| MB-5554 | 0.0072 | 0.0000 | 0.1088 | 0.0141 | 0.0000 | 0.1890 | 0.0000 | 0.0467 | 0.0143 | 0.0705 | 0.0000 | 0.0739 | 0.0000 | 0.0000 | 0.0814 | 0.2228 | 0.0067 | 0.0000 | 0.1647 | 0.0000 | 0.0000 | 0.0000 |
| MB-4421 | 0.0272 | 0.0000 | 0.0062 | 0.0000 | 0.2388 | 0.0000 | 0.0000 | 0.0464 | 0.1219 | 0.0533 | 0.0271 | 0.0090 | 0.0000 | 0.3842 | 0.0094 | 0.0631 | 0.0000 | 0.0000 | 0.0134 | 0.0000 | 0.0000 | 0.0000 |
| MB-4408 | 0.0186 | 0.0000 | 0.0528 | 0.1170 | 0.0000 | 0.0000 | 0.0220 | 0.1536 | 0.1202 | 0.0015 | 0.0121 | 0.0343 | 0.0000 | 0.2786 | 0.1047 | 0.0410 | 0.0000 | 0.0000 | 0.0438 | 0.0000 | 0.0000 | 0.0000 |
| MB-4146 | 0.0844 | 0.0000 | 0.0048 | 0.1646 | 0.1672 | 0.0000 | 0.0110 | 0.0914 | 0.1079 | 0.0000 | 0.0683 | 0.0168 | 0.0101 | 0.1360 | 0.0869 | 0.0000 | 0.0002 | 0.0000 | 0.0503 | 0.0000 | 0.0000 | 0.0000 |
| MB-5534 | 0.0507 | 0.0000 | 0.0000 | 0.0720 | 0.0000 | 0.0671 | 0.0294 | 0.1120 | 0.0000 | 0.1825 | 0.0000 | 0.1103 | 0.0150 | 0.1168 | 0.0900 | 0.0876 | 0.0000 | 0.0000 | 0.0667 | 0.0000 | 0.0000 | 0.0000 |
| MB-5521 | 0.0430 | 0.0000 | 0.0000 | 0.1948 | 0.0000 | 0.0000 | 0.0474 | 0.0833 | 0.0000 | 0.0850 | 0.0000 | 0.1096 | 0.0129 | 0.1212 | 0.1018 | 0.0967 | 0.0000 | 0.0000 | 0.1043 | 0.0000 | 0.0000 | 0.0000 |
| MB-5482 | 0.0118 | 0.0000 | 0.0268 | 0.0000 | 0.0380 | 0.1933 | 0.0000 | 0.0695 | 0.0000 | 0.0000 | 0.0657 | 0.0000 | 0.0874 | 0.1387 | 0.1510 | 0.1080 | 0.0325 | 0.0220 | 0.0552 | 0.0000 | 0.0000 | 0.0000 |
| MB-5483 | 0.0215 | 0.0233 | 0.0404 | 0.0362 | 0.0000 | 0.0137 | 0.0000 | 0.0457 | 0.0440 | 0.0137 | 0.0000 | 0.0365 | 0.0090 | 0.3103 | 0.0692 | 0.2203 | 0.0000 | 0.0000 | 0.1161 | 0.0000 | 0.0000 | 0.0000 |
| MB-5532 | 0.0447 | 0.0000 | 0.0246 | 0.0129 | 0.0741 | 0.0000 | 0.0000 | 0.0039 | 0.0157 | 0.0809 | 0.0075 | 0.0000 | 0.0000 | 0.3768 | 0.0254 | 0.2034 | 0.0000 | 0.0000 | 0.0738 | 0.0562 | 0.0000 | 0.0000 |
| MB-5556 | 0.0108 | 0.0000 | 0.0000 | 0.0236 | 0.0000 | 0.0057 | 0.0173 | 0.0592 | 0.0179 | 0.0000 | 0.0000 | 0.0869 | 0.0862 | 0.1921 | 0.1938 | 0.1212 | 0.0000 | 0.0000 | 0.1853 | 0.0000 | 0.0000 | 0.0000 |
| MB-5518 | 0.0173 | 0.0000 | 0.0140 | 0.0543 | 0.0031 | 0.0000 | 0.0014 | 0.1099 | 0.0142 | 0.0906 | 0.0000 | 0.0349 | 0.0000 | 0.3610 | 0.1250 | 0.1215 | 0.0056 | 0.0000 | 0.0471 | 0.0000 | 0.0000 | 0.0000 |
| MB-5486 | 0.0156 | 0.0000 | 0.0028 | 0.1852 | 0.0000 | 0.0256 | 0.0000 | 0.0811 | 0.0104 | 0.0442 | 0.0000 | 0.0635 | 0.0000 | 0.1431 | 0.1390 | 0.1533 | 0.0009 | 0.0000 | 0.1354 | 0.0000 | 0.0000 | 0.0000 |
| MB-5511 | 0.0000 | 0.0000 | 0.0474 | 0.0000 | 0.0000 | 0.2508 | 0.0000 | 0.0555 | 0.0362 | 0.0792 | 0.0000 | 0.0765 | 0.0043 | 0.0647 | 0.1121 | 0.1765 | 0.0229 | 0.0000 | 0.0739 | 0.0000 | 0.0000 | 0.0000 |
| MB-5422 | 0.0405 | 0.0000 | 0.0298 | 0.0637 | 0.0000 | 0.0244 | 0.0000 | 0.0676 | 0.0224 | 0.0044 | 0.0000 | 0.0255 | 0.0000 | 0.3501 | 0.1168 | 0.0952 | 0.0000 | 0.0000 | 0.1597 | 0.0000 | 0.0000 | 0.0000 |
| MB-5535 | 0.0136 | 0.0060 | 0.0000 | 0.0325 | 0.0000 | 0.0967 | 0.0000 | 0.1144 | 0.0000 | 0.0543 | 0.0000 | 0.0564 | 0.0000 | 0.1997 | 0.1868 | 0.1073 | 0.0000 | 0.0000 | 0.1322 | 0.0000 | 0.0000 | 0.0000 |
| MB-5477 | 0.0000 | 0.0000 | 0.0038 | 0.0153 | 0.0000 | 0.0388 | 0.0000 | 0.0814 | 0.0088 | 0.0179 | 0.0000 | 0.0249 | 0.0000 | 0.3154 | 0.0604 | 0.3601 | 0.0052 | 0.0000 | 0.0679 | 0.0000 | 0.0000 | 0.0000 |
| MB-5472 | 0.0200 | 0.0169 | 0.0000 | 0.0000 | 0.0215 | 0.0000 | 0.0000 | 0.0838 | 0.0576 | 0.1528 | 0.0000 | 0.0000 | 0.0390 | 0.1127 | 0.0437 | 0.1738 | 0.0363 | 0.0678 | 0.0843 | 0.0899 | 0.0000 | 0.0000 |
| MB-5540 | 0.0129 | 0.0000 | 0.1486 | 0.1219 | 0.0086 | 0.0000 | 0.0675 | 0.0975 | 0.0173 | 0.0000 | 0.0000 | 0.0598 | 0.0112 | 0.1124 | 0.1008 | 0.1668 | 0.0136 | 0.0093 | 0.0492 | 0.0000 | 0.0000 | 0.0027 |
| MB-5184 | 0.0427 | 0.0000 | 0.0009 | 0.0735 | 0.0000 | 0.1517 | 0.0000 | 0.0658 | 0.0000 | 0.0000 | 0.0000 | 0.0698 | 0.0796 | 0.0000 | 0.0250 | 0.1373 | 0.0122 | 0.0008 | 0.3408 | 0.0000 | 0.0000 | 0.0000 |
| MB-5294 | 0.0545 | 0.0000 | 0.0301 | 0.0635 | 0.0303 | 0.1562 | 0.0185 | 0.1183 | 0.0000 | 0.0000 | 0.0411 | 0.0105 | 0.0631 | 0.1679 | 0.1225 | 0.0703 | 0.0000 | 0.0000 | 0.0531 | 0.0000 | 0.0000 | 0.0000 |
| MB-5565 | 0.0000 | 0.0096 | 0.0181 | 0.0000 | 0.0177 | 0.1743 | 0.0343 | 0.0670 | 0.0000 | 0.0200 | 0.0000 | 0.1079 | 0.0000 | 0.1660 | 0.1519 | 0.1637 | 0.0019 | 0.0000 | 0.0677 | 0.0000 | 0.0000 | 0.0000 |
| MB-5040 | 0.0026 | 0.0000 | 0.0321 | 0.0987 | 0.0000 | 0.0000 | 0.0000 | 0.0787 | 0.0371 | 0.0402 | 0.0000 | 0.0566 | 0.0000 | 0.2663 | 0.0629 | 0.2143 | 0.0000 | 0.0000 | 0.1033 | 0.0000 | 0.0000 | 0.0072 |
| MB-4801 | 0.0753 | 0.0000 | 0.0000 | 0.0000 | 0.0826 | 0.0000 | 0.0000 | 0.0095 | 0.1149 | 0.0514 | 0.0777 | 0.0000 | 0.0000 | 0.1602 | 0.0466 | 0.1459 | 0.0000 | 0.0000 | 0.2169 | 0.0000 | 0.0191 | 0.0000 |
| MB-5499 | 0.0333 | 0.0000 | 0.0515 | 0.0542 | 0.0000 | 0.1600 | 0.0000 | 0.0218 | 0.0177 | 0.0324 | 0.0000 | 0.0759 | 0.0509 | 0.0094 | 0.0987 | 0.1365 | 0.0457 | 0.0000 | 0.2119 | 0.0000 | 0.0000 | 0.0000 |
| MB-5459 | 0.0886 | 0.0000 | 0.0000 | 0.0092 | 0.0000 | 0.0000 | 0.0000 | 0.0595 | 0.0096 | 0.0000 | 0.0456 | 0.0000 | 0.0000 | 0.3940 | 0.0387 | 0.1707 | 0.0000 | 0.0000 | 0.1634 | 0.0156 | 0.0000 | 0.0051 |
| MB-5243 | 0.0098 | 0.0000 | 0.0000 | 0.0432 | 0.0000 | 0.0390 | 0.0000 | 0.0650 | 0.0025 | 0.0000 | 0.0000 | 0.0432 | 0.0020 | 0.1534 | 0.1237 | 0.1027 | 0.0044 | 0.0000 | 0.4112 | 0.0000 | 0.0000 | 0.0000 |
| MB-4120 | 0.0727 | 0.0000 | 0.0000 | 0.0000 | 0.0884 | 0.0000 | 0.0000 | 0.0034 | 0.0229 | 0.1131 | 0.0198 | 0.0000 | 0.0000 | 0.3787 | 0.1254 | 0.0000 | 0.0217 | 0.0000 | 0.1540 | 0.0000 | 0.0000 | 0.0000 |
| MB-4966 | 0.0564 | 0.0000 | 0.0345 | 0.0728 | 0.0000 | 0.0155 | 0.0000 | 0.0840 | 0.0124 | 0.1195 | 0.0000 | 0.0726 | 0.0000 | 0.0690 | 0.1064 | 0.2061 | 0.0039 | 0.0000 | 0.1469 | 0.0000 | 0.0000 | 0.0000 |
| MB-5457 | 0.0000 | 0.0000 | 0.0066 | 0.0864 | 0.0000 | 0.1270 | 0.0000 | 0.0763 | 0.0113 | 0.0956 | 0.0000 | 0.0455 | 0.0000 | 0.1806 | 0.1320 | 0.0968 | 0.0239 | 0.0000 | 0.1180 | 0.0000 | 0.0000 | 0.0000 |
| MB-4011 | 0.0740 | 0.0000 | 0.0101 | 0.0756 | 0.0425 | 0.0079 | 0.0299 | 0.0293 | 0.0235 | 0.0186 | 0.0502 | 0.0000 | 0.0000 | 0.2551 | 0.1384 | 0.1008 | 0.0069 | 0.0000 | 0.1372 | 0.0000 | 0.0000 | 0.0000 |
| MB-5478 | 0.0388 | 0.0000 | 0.0000 | 0.0658 | 0.0529 | 0.1247 | 0.0000 | 0.0474 | 0.0000 | 0.1007 | 0.0000 | 0.1252 | 0.0193 | 0.0713 | 0.1535 | 0.0815 | 0.0055 | 0.0000 | 0.1134 | 0.0000 | 0.0000 | 0.0000 |
| MB-4822 | 0.0605 | 0.0000 | 0.0033 | 0.1549 | 0.0381 | 0.0265 | 0.0082 | 0.0680 | 0.0040 | 0.0000 | 0.0000 | 0.0279 | 0.0468 | 0.2611 | 0.1471 | 0.0778 | 0.0000 | 0.0000 | 0.0759 | 0.0000 | 0.0000 | 0.0000 |
| MB-4017 | 0.1087 | 0.0000 | 0.0537 | 0.0384 | 0.0000 | 0.1161 | 0.0000 | 0.0754 | 0.0453 | 0.0401 | 0.0000 | 0.0723 | 0.0000 | 0.1609 | 0.0747 | 0.1005 | 0.0000 | 0.0000 | 0.1138 | 0.0000 | 0.0000 | 0.0000 |
| MB-5018 | 0.0239 | 0.0000 | 0.0117 | 0.0732 | 0.0000 | 0.0803 | 0.0000 | 0.0748 | 0.0000 | 0.0668 | 0.0000 | 0.0635 | 0.0228 | 0.0323 | 0.0820 | 0.2842 | 0.0000 | 0.0000 | 0.1845 | 0.0000 | 0.0000 | 0.0000 |
| MB-4791 | 0.0290 | 0.0000 | 0.0450 | 0.0528 | 0.0000 | 0.0069 | 0.0000 | 0.1006 | 0.0298 | 0.2123 | 0.0000 | 0.0877 | 0.0000 | 0.1325 | 0.1082 | 0.1274 | 0.0000 | 0.0000 | 0.0678 | 0.0000 | 0.0000 | 0.0000 |
| MB-5118 | 0.0570 | 0.0000 | 0.0412 | 0.0458 | 0.0000 | 0.1125 | 0.0000 | 0.0880 | 0.0288 | 0.0000 | 0.0084 | 0.0823 | 0.0243 | 0.0705 | 0.1446 | 0.1903 | 0.0174 | 0.0000 | 0.0887 | 0.0000 | 0.0000 | 0.0000 |
| MB-4806 | 0.0173 | 0.0000 | 0.0200 | 0.0418 | 0.0000 | 0.0853 | 0.0000 | 0.0393 | 0.0009 | 0.0000 | 0.0000 | 0.0618 | 0.0029 | 0.0370 | 0.0231 | 0.1010 | 0.0000 | 0.0125 | 0.5572 | 0.0000 | 0.0000 | 0.0000 |
| MB-4970 | 0.0433 | 0.0000 | 0.0121 | 0.0000 | 0.0865 | 0.0586 | 0.0000 | 0.0555 | 0.0000 | 0.0834 | 0.0000 | 0.0764 | 0.0397 | 0.2197 | 0.0537 | 0.1721 | 0.0099 | 0.0000 | 0.0890 | 0.0000 | 0.0000 | 0.0000 |
| MB-2964 | 0.0075 | 0.0000 | 0.1057 | 0.0211 | 0.0000 | 0.0529 | 0.0000 | 0.0578 | 0.0266 | 0.0310 | 0.0000 | 0.0398 | 0.0252 | 0.1964 | 0.1150 | 0.1101 | 0.0175 | 0.0000 | 0.1933 | 0.0000 | 0.0000 | 0.0000 |
| MB-2963 | 0.0043 | 0.0000 | 0.0079 | 0.0112 | 0.0293 | 0.0406 | 0.0000 | 0.0464 | 0.0069 | 0.0526 | 0.0000 | 0.0322 | 0.0143 | 0.2695 | 0.0472 | 0.2145 | 0.0044 | 0.0000 | 0.2188 | 0.0000 | 0.0000 | 0.0000 |
| MB-2957 | 0.0432 | 0.0000 | 0.0832 | 0.0236 | 0.0000 | 0.0339 | 0.0076 | 0.1008 | 0.0000 | 0.0575 | 0.0000 | 0.0161 | 0.0320 | 0.2153 | 0.1746 | 0.1780 | 0.0000 | 0.0000 | 0.0343 | 0.0000 | 0.0000 | 0.0000 |
| MB-2954 | 0.0213 | 0.0276 | 0.0000 | 0.1006 | 0.0000 | 0.0000 | 0.0000 | 0.0121 | 0.0120 | 0.0000 | 0.0190 | 0.0000 | 0.0561 | 0.1620 | 0.0007 | 0.4169 | 0.0196 | 0.0105 | 0.1284 | 0.0000 | 0.0131 | 0.0000 |
| MB-2916 | 0.0215 | 0.0000 | 0.0179 | 0.0562 | 0.0000 | 0.2667 | 0.0000 | 0.0253 | 0.0000 | 0.0000 | 0.0000 | 0.0366 | 0.0704 | 0.0361 | 0.1155 | 0.0982 | 0.0298 | 0.0000 | 0.2259 | 0.0000 | 0.0000 | 0.0000 |
| MB-2725 | 0.0080 | 0.0000 | 0.0951 | 0.0205 | 0.0000 | 0.0879 | 0.0000 | 0.0272 | 0.0128 | 0.0356 | 0.0000 | 0.0350 | 0.0397 | 0.1068 | 0.0317 | 0.1402 | 0.0000 | 0.0000 | 0.3595 | 0.0000 | 0.0000 | 0.0000 |
[truncated: 217,245 more chars]
